# IMPRESSION generation 2 – accurate, fast and generalised neural network model for predicting NMR parameters in place of DFT.†

**DOI:** 10.1039/d4sc07858f

**Published:** 2025-03-31

**Authors:** Calvin Yiu, Ben Honoré, Will Gerrard, Jose Napolitano-Farina, Dave Russell, Iuni Margaret Laura Trist, Ruth Dooley, Craig P. Butts

**Affiliations:** a School of Chemistry, University of Bristol UK Craig.Butts@Bristol.ac.uk; b Genentech Inc. USA; c Evotec (UK) Ltd Milton Park Abingdon UK

## Abstract

Predicting 3D-aware Nuclear Magnetic Resonance (NMR) properties is critical for determining the 3D structure and dynamics, both stereochemical and conformational, of molecules in solution. Existing tools for such predictions are limited, being either relatively slow quantum chemical methods such as Density Functional Theory (DFT), or niche parameterised empirical or machine learning methods that only predict a single parameter type, often across only a limited chemical space. We present here IMPRESSION-Generation 2 (G2), a transformer-based neural network which can be used as a much faster alternative to high level DFT calculations in computational workflows of multiple classes of NMR parameter simultaneously, with time-savings of several orders of magnitude. IMPRESSION-G2 is the first system that simultaneously predicts all NMR chemical shifts, as well as scalar couplings for ^1^H, ^13^C, ^15^N and ^19^F nuclei up to 4 bonds apart, in a single prediction event starting from a 3D molecular structure. Rapid NMR predictions take <50 ms to predict on average ∼5000 chemical shifts and scalar couplings per molecule, which is approximately 10^6^-times faster than DFT-based NMR predictions starting from a 3D structure. When combined with fast GFN2-xTB geometry optimisations to generate the 3D input structures themselves in just a few seconds, a complete workflow for NMR predictions on a new molecule is 10^3^–10^4^ times faster than a wholly DFT-based workflow for this. The accuracy of this multi-parameter predictor in reproducing DFT-quality results for a wide chemical space of organic molecules up to ∼1000 g mol^−1^ containing C, H, N, O, F, Si, P, S, Cl, Br exceeds that of existing state-of-the-art empirical or machine learning systems (∼0.07 ppm for ^1^H chemical shifts, ∼0.8 ppm for ^13^C chemical shifts, <0.15 Hz for ^3^*J*_HH_ scalar coupling constants) and, critically, it also demonstrates generalisability when tested against molecules from sources that are completely independent of its own training data. When compared to experimental NMR data for ∼5000 compounds, IMPRESSION-G2 gives results in minutes on a standard laptop which are almost indistinguishable from DFT results that took days on a large scale High Performance Computing system. This accuracy and speed of IMPRESSION-G2 coupled to GFN-xTB shows that it can be used to simply replace DFT for predicting 3D-aware NMR parameters inside the wide chemical space of its training data.

## Abstract 2

Predicting 3D-aware Nuclear Magnetic Resonance (NMR) properties is critical for determining the 3D structure and dynamics, both stereochemical and conformational, of molecules in solution.

## Graphical abstract

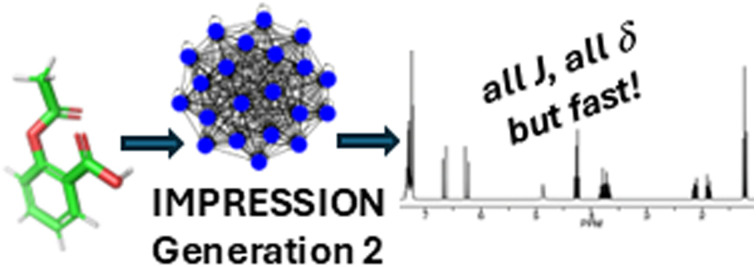

## Introduction

When establishing the structure or dynamics of a molecule in solution, NMR spectroscopy is arguably the most powerful tool in a chemist's arsenal. It provides atomic level detail on both connectivity of atoms and their local 3D structure. Modern 3-dimensional molecular structure elucidation by NMR generally uses quantitative fitting between experimental values and predicted/computed values for one or more proposed chemical structures.^1,2^ This approach has been enhanced by development of statistical tools, such as DP4/DP5,^3,4^ to better discriminate correct from incorrect proposed structures in such comparisons. In order to make any comparison efficiently however, the NMR prediction methods must be both fast and accurate, especially where multiple potential candidate structures are to be tested, and cover all of the NMR parameters to be compared.

Traditional rapid empirical methods for predicting NMR chemical shifts are limited mostly to 2-dimensional structures and cannot readily deal with 3-dimensional conformational or stereochemical analysis. For example, the additivity rules of Pretsch^5^ and HOSE-codes^6^ are inherently ‘flat’, with some modifications to treating for 3-dimensionality by *e.g.* flat-but-stereochemically-aware HOSE codes^7^ or conformational ensemble models for experimental systems.^8–10^ The most accurate tool for fully 3D-aware NMR predictions are quantum chemical calculations, typically based on Density Functional Theory (DFT).^11–14^ The best DFT methods can reproduce experiment to within 1–2% of the appropriate range of parameter values *i.e.* 0.2–0.3/2–4 ppm^15,16^ on ranges of ∼10/∼200 ppm for ^1^H and ^13^C chemical shifts respectively, across a very wide range of chemical structure space. However accurate DFT is very slow, especially when calculating for multiple molecules and/or conformers – full workflows typically take hours to days of CPU time for NMR predictions for each 3D geometry of a molecule of moderate size (say 30–40 non-H atoms). Naturally, if multiple conformers or isomers must be considered then the computation time can become days to months of computation for a single study, which rapidly becomes impractical.

Accurate prediction of scalar coupling constants are more directly linked to 3-dimensional structure than chemical shifts, through their high dependency on the dihedral angles of intervening bonds between the coupled nuclei. Generic Karplus-style empirical relationships, such as that from Haasnoot *et al.*,^17^ provide a partial solution for specific coupling types, *e.g.* 3-bond ^1^H–^1^H and ^1^H–^13^C, but they lose accuracy for even moderately complex structures, for example where heteroatoms introduce stereoelectronic effects. While bespoke versions of these can be optimised to deal with specific sub-structures, such as often used with carbohydrates,^18^ they are conversely not generalisable to molecules outside that specific chemical space. Finally, many NMR parameters which have great potential value in molecular structure elucidation, for example ^15^N chemical shifts and 1-bond ^1^H–^13^C scalar coupling constants, ^1^*J*_CH_, are much more rarely used in quantitative comparison simply because there are not reliable, fast and accurate predictive methods for them, but one must ask the question – what if there were?

Machine learning systems, trained on DFT-computed NMR parameters for 3D molecular structures, offer a solution to all of these issues. They are much faster to run than DFT NMR predictions, executing in seconds rather than hours or days. Machines for 2D-based (no conformation or stereochemistry) predictions for ^1^H and ^13^C chemical shifts exist and are typically trained on many thousands of literature experimental chemical shift data.^19–22^ These experimental chemical shift datasets are of variable quality due to limitations in measurement accuracy and errors of reporting by researchers. Training such machines for prediction of scalar couplings, on the other hand, is generally not even possible because large, accurate and validated experimental databases simply do not exist with the associated 3D molecular structures that are critical to scalar coupling constants (*e.g.*^3^*J*_HH/CH_ values). On the other hand, large datasets of both DFT-computed chemical shifts and scalar couplings can be generated accurately, fully validated and ensure a direct match of those parameters to a single 3D structure. Also, datasets of DFT-generated structures can readily be made more diverse, as they are not limited only to chemical structures similar to previously experimentally studied molecules. The only downsides are then how accurately the machine reproduces the DFT result and how accurate the DFT method is in reproducing experiment. Paruzzo *et al.* first reported this approach for machine learning-based prediction of DFT-like solid-state NMR chemical shifts with ShiftML based on a kernel-ridge regression approach.^23,24^ Soon after, we demonstrated a similar architecture for solution-state NMR predictions with the first generation of our IMPRESSION model^25^ which could generate predictions comparable to DFT with mean absolute deviations of 0.23 ppm (*δ*^1^H) and 2.45 ppm (*δ*^13^C) for chemical shifts, as well as predicting ^1^*J*_CH_ (MAD = 0.87 Hz). IMPRESSION was trained on 882 chemical structures, covering the same relatively limited chemical space as ShiftML (C,H,N,O,F only) and was limited in training dataset size by the kernel ridge regression architecture and resulting memory-demands of its molecular representation. CASCADE from Guan *et al.*^26^ later reported two separate message passing neural networks that provide ^1^H or ^13^C chemical shift predictions respectively. Both CASCADE machines were trained on ∼8000 DFT-derived molecular structures (DFT8K) and provided accuracies approaching 0.10 ppm (*δ*^1^H) and 1.26 ppm (*δ*^13^C) against an internal hold-out of structures from that same training data, with testing outcomes against external datasets not reported.

Herein we introduce our second generation system with a transformer-based neural network architecture, IMPRESSION-G2. This simultaneously predicts all defined types of scalar coupling constants and chemical shifts with DFT-like accuracy but much higher computational efficiency. Its performance is assessed against both computed and experimental external test sets to ensure generalisability, and we demonstrate that it can effectively replace DFT in such workflows, while providing orders of magnitude in time-savings.

## Results and discussion

### Datasets and methods

**Fig. 1:**
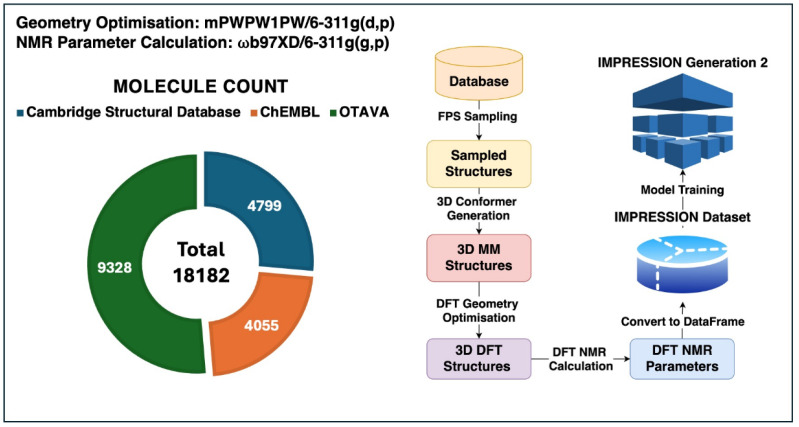
Workflow for training IMPRESSION-Generation 2 including DFT methodology and training and testing dataset sources. Full details can be found in the ESI.†

The IMPRESSION-G2 system is based on a graph transformer network,^27^ inspired by our community-search project for NMR prediction,^28^ that simultaneously predicts a variety of NMR parameters as accurately, or better, than existing machine learning systems. The transformer architecture, using attention mechanisms, enables IMPRESSION-G2 to optimise the transfer of information between all NMR parameters during training and inference, *e.g.* chemical shift information from one nucleus will inform the scalar coupling constants to, and between, other nuclei and *vice versa*. To leverage this capability, the NMR parameters used cover chemical shifts for all spin-active elements in the training set, as well as all ^1^H, ^13^C, ^15^N and ^19^F scalar couplings for nuclei up to 4 bonds apart. Together, these NMR parameters represent those most commonly used in the structure elucidation of organic molecules by NMR spectroscopy in solution. The training data were derived from DFT calculations (Fig. 1), collating a database of 18 182 molecules from three sources: the Cambridge Structural Database^29^ (4799 molecules) which comprises >1 M molecules for which X-ray diffractometry crystal structures have been reported; ChEMBL^30,31^ (4055 molecules) which is a manually curated database of ∼2.4 M bioactive molecules with drug-like properties; and the OTAVA chemicals diversity library^32^ (9328 molecules) which contains ∼10k diversity-selected drug-like molecules. These diverse data sources were selected to improve the generalisability of the trained machine when tested against externally sourced molecules. Further details of the structures and methods used to select for, and subsequently refine, the diversity of chemical space covered by the training dataset can be found in the ESI.†

NMR parameters for each training molecule were predicted with DFT from a single 3D structure, using mPW1PW91/6-311g(d,p) for geometry optimisation, and ωB97XD/6-311g(d,p) for NMR predictions,^33–37^ providing 739 913 chemical shift environments (330 411 *δ*^1^H; 306 458 δ^13^C) and 5 696 784 scalar coupling constants (including 307 270 ^1^*J*_CH_; 486 884 ^2^*J*_CH_; 672 433 ^3^*J*_CH_; 705 737 ^4^*J*_CH_; 134 051 ^2^*J*_HH_; 217 940 ^3^*J*_HH_; 333 010 ^4^*J*_HH_) with the latter divided into their labelled sets, ^*n*^*J*_XY_, depending on the number of bonds (*n*) between the coupled pairs of nuclei (X and Y). Details of the DFT workflows and neural network architecture are available in the ESI.†

### Performance *vs.* DFT

Using the training set, totalling 16 304 molecules after a 10% hold-out of molecules for testing were removed, IMPRESSION-G2 achieved excellent performance in reproducing DFT-quality predictions for both ^1^H and ^13^C chemical shifts (Mean Absolute Deviation (MAD) = 0.07 ppm ^1^H, 0.76 ppm ^13^C; Table 1, entry 1) against this 10% internal holdout. The key driver for training machine learning systems for NMR predictions is time-saving over DFT alternatives. IMPRESSION-G2 takes only ∼50 milliseconds per molecular structure to predict all NMR parameters with the accuracy described. This is ∼10^6^ times faster than the hours to days required per molecular structure for the corresponding DFT-based calculation of chemical shift and coupling constants. The time-saving benefits will be especially marked when making predictions for large numbers of compounds or flexible molecules with multiple 3D structures *i.e.* conformers, that would be extremely challenging using DFT-based NMR prediction. It should also be noted that DFT predictions of couplings constants take considerably longer than those for chemical shifts alone, which is a significant barrier to using DFT for that purpose.

**Table 1:** NMR prediction accuracy against DFT (ωB97XD/6-311g(d,p)) for key ^1^H and ^13^C chemical shifts and scalar coupling constants, highlighting performance based on machine learning system and training/testing dataset

Entry	Predictor	Training dataset	Testing dataset	3D geometry	*δ* ^1^H/ppm	*δ* ^13^C/ppm	^3^ *J*_HH_/Hz	^2^ *J*_HH_/Hz	^3^ *J*_CH_/Hz	^2^ *J*_CH_/Hz	^1^ *J*_CH_/Hz
1	IMPRESSION-G2	IG2	Internal	DFT	0.07	0.76	0.12	0.13	0.15	0.15	0.36
2	IMPRESSION-G2	IG2	CSD-500	DFT	0.09	0.97	0.14	0.14	0.19	0.18	0.44
3	IMPRESSION-G2	IG2	DFT8K^a^	DFT	0.09	1.27	0.14	0.17	0.20	0.20	0.54
4	IMPRESSION-G2	IG2	CSD-500	GFN2-xTB	0.13	1.18	0.31	0.36	0.29	0.25	0.68
5	IMPRESSION-G2	IG2	DFT8K^a^	GFN2-xTB	0.13	1.46	0.31	0.33	0.32	0.27	0.74
6	IMPRESSION^25^	IG1	CSD-500	DFT	0.23	2.45	—	—	—	—	0.87
7	CASCADE^26^	DFT8K^b^	Internal	DFT	0.10	1.26	—	—	—	—	—

a Test result against all molecules in DFT8K, recalculated using the same DFT method (ωB97xd/6-311g(d,p)) used for IMPRESSION-G2.

b Testing result reported by Guan *et al.*,^26^ with both training and testing sets calculated using the mPW1PW91/6-311+G(d,p) DFT method.

Beyond simple time-saving, the ^1^H and ^13^C chemical shift performance of IMPRESSION-G2 against DFT on an internal holdout improves on the current gold standard CASCADE predictor performance (Table 1, entry 7; MAD = 0.10 ppm ^1^H, 1.26 ppm ^13^C). We note that this improvement in performance exceeds what is expected solely on the basis of the slightly (∼2×) larger training dataset used for IMPRESSION-G2, as a 10-fold increase in training size is generally required to deliver a 2-fold improvement in accuracy. This suggests that the transformer architecture of IMPRESSION-G2, with attention passed between NMR parameters, also offers some benefits to accuracy during training.

The key test of any machine learning system is how it performs in generalisation tasks *i.e.* predictions on external sets of molecules that are entirely independent of those from which it was trained. Here we first compared against the relatively forgiving CSD-500 testing set used for the original IMPRESSION report (410 chemical structures comprising C,H,N,O reported by Paruzzo *et al.* for ShiftML,^23^ comprising 8475 *δ*^1^H; 7523 *δ*^13^C environments). IMPRESSION-G2 again provided excellent performance (MAD = 0.09 ppm ^1^H, 0.97 ppm ^13^C; Table 1, entry 2) that is ∼2.5-times better than the original IMPRESSION^25^ using the same test (Table 1, entry 6). We also tested IMPRESSION-G2 against CASCADE's more challenging DFT8K dataset of molecules,^26^ which contains a greater diversity of elements than CSD-500 and is sourced from a database (NMRShiftDB) that is entirely independent of those used to curate our training set. Excellent performance was again observed (MAD = 0.09 ppm ^1^H, 1.27 ppm ^13^C; Table 1, entry 3) suggesting the IMPRESSION-G2 model is indeed generalisable across a wide chemical space for ^1^H and ^13^C chemical shift prediction in molecules containing C, H, N, O, F, Si, P, S, Cl, Br, including independent data sources.

Accurate chemical shift predictions for ^15^N and ^19^F are also made simultaneously by IMPRESSION-G2 and predicted well (MAD = 2.26 ppm ^15^N, 2.60 ppm ^19^F). These accuracies of <3 ppm are comparable to the best reported DFT methods for ^19^F,^38,39^ and a substantial improvement for ^15^N over the original kernel ridge-based IMPRESSION model (MAD = 6.20 ppm).^40^

It is important to note that while predicting dozens of chemical shifts per molecule, IMPRESSION is simultaneously predicting hundreds to thousands of coupling constants for each molecule, providing extremely efficient computation. The accuracy of these scalar coupling constants predictions is also very high (Table 1). Multiple-bond ^1^H–^1^H and ^1^H–^13^C coupling constants were predicted with high accuracy against the internal hold-out (MAD ≤ 0.15 Hz; Table 1, entry 1) and both CSD500 and DFT8K external testing sets (MAD < 0.2 Hz, Table 1, entries 2 and 3). Similarly, one-bond ^1^H–^13^C scalar coupling accuracy (MAD ∼ 0.5 Hz; Table 1, entries 2 and 3) were nearly twice as accurate as those from the original IMPRESSION system (Table 1, entry 6).^40^ IMPRESSION-G2 thus represents a step-change in NMR parameter prediction as currently DFT is the only generalisable tool to predict scalar coupling constants across 1–4 bonds and IMPRESSION-G2 is the first system capable of reproducing such DFT calculations but much more rapidly than DFT can achieve.

It should be noted that the main time-limiting feature for IMPRESSION-G2 workflows is how long it takes to generate the 3D structures prior to NMR prediction. The accuracies described above were achieved by starting from DFT-based 3D molecular geometries *i.e.* the overall workflow to achieve this accuracy still required a slow (minute to hours) DFT geometry optimisation prior to running IMPRESSION-G2. Gratifyingly, IMPRESSION-G2 predictions are still accurate when executed on 3D molecular structures derived from much more rapid GFN2-xTB optimisations.^41^ This was tested against both CSD-500 and DFT8K (Table 1, entries 4 and 5 compared to entries 2 and 3) and these calculations took only a few seconds per molecule to deliver the combined 3D geometry optimisation and IMPRESSION-G2 NMR prediction, *i.e.* ∼10^4^ times faster than a full DFT workflow.

### Performance *vs.* experiment

**Fig. 2:**
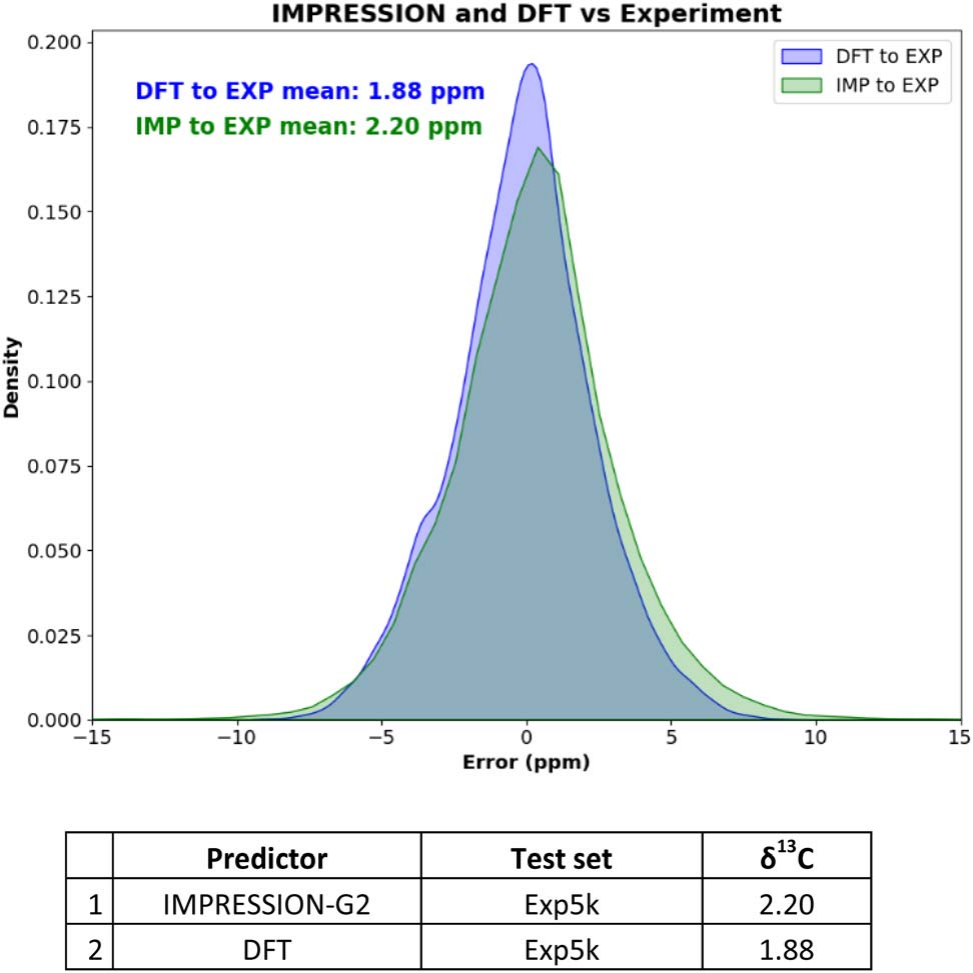
Table of mean absolute deviations and overlay of error distributions for DFT ωB97XD/6-311g(d,p) and IMPRESSION-Generation 2 *vs.* Exp5K, as well as DFT ωB97XD/6-311g(d,p) *vs.* IMPRESSION.

Naturally the ultimate test of an NMR prediction system is against experiment. The ability of IMPRESSION-G2 to reproduce experimental data was first explored using ^13^C chemical shift data from the ‘Exp5K’^26^ subset of DFT8K. The Exp5K *δ*^13^C experimental values, derived from literature data in the NMRShiftDB contain a diverse range of chemical structures covering both flexible and rigid molecules. The EXP5K dataset was validated by Guan *et al.*^26^ against DFT prediction to minimise assignment/interpretation errors in the experimental dataset. Gratifyingly IMPRESSION-G2 offered excellent accuracy (MAD = 2.20 ppm; Fig. 2, table, entry 1) which is comparable to the performance of the ωB97XD/6-311g(d,p) DFT method itself on these same molecules (MAD = 1.88 ppm; Fig. 2 table, entry 2). It should be noted that the absolute accuracy of both DFT and IMPRESSION comparisons to experimental data is always limited by how accurately one reproduces the conformational landscape of flexible molecules. Our goal is to show the comparability of DFT and IMPRESSION here, so we used identical single geometries as input for each compound for both DFT and IMPRESSION NMR predictions, full details can be found in Section 4.2 of the ESI.†

The overlay of error distributions in Fig. 2 for the IMPRESSION-G2 and DFT methods further demonstrates the comparability of these two approaches across the ∼5k molecules. However the DFT predictions (DFT geometry optimisations and NMR predictions) took days even on a large parallelised high-performance computing cluster, while IMPRESSION-G2 (GFN2-xTB geometry optimisations and NMR predictions) completed the whole dataset in <10 minutes on a standard laptop. While each individual IMPRESSION-G2 prediction is different to what DFT would predict, the ensemble of predictions from IMPRESSION (green) is nearly indistinguishable from DFT (blue). This strongly supports our conclusion that IMPRESSION-G2 can be used as a drop-in replacement for DFT when predicting NMR parameters for any molecules inside the chemical space (C, H, N, O, F, Si, P, S, Cl, Br) for which it has been trained.

Finally we explored what improvements IMPRESSION-G2 provides in the critical application of 3D-structure molecular determination. This is demonstrated here through the enhanced 3D-structure discrimination offered by predicted coupling constants with IMPRESSION-G2, as opposed to just chemical shifts, in the diastereomeric determination of strychnine. Strychnine is a well-studied example, has a nearly perfectly ‘rigid’ structure,^42^ and has rigorously validated and tested NMR assignments of both chemical shifts and couplings across multiple literature reports. Consequently it allows us to test IMPRESSION-G2 while avoiding complications arising from errors in conformer population averaging and the misassignments of experimental NMR data that abound in literature reports.

**Fig. 3:**
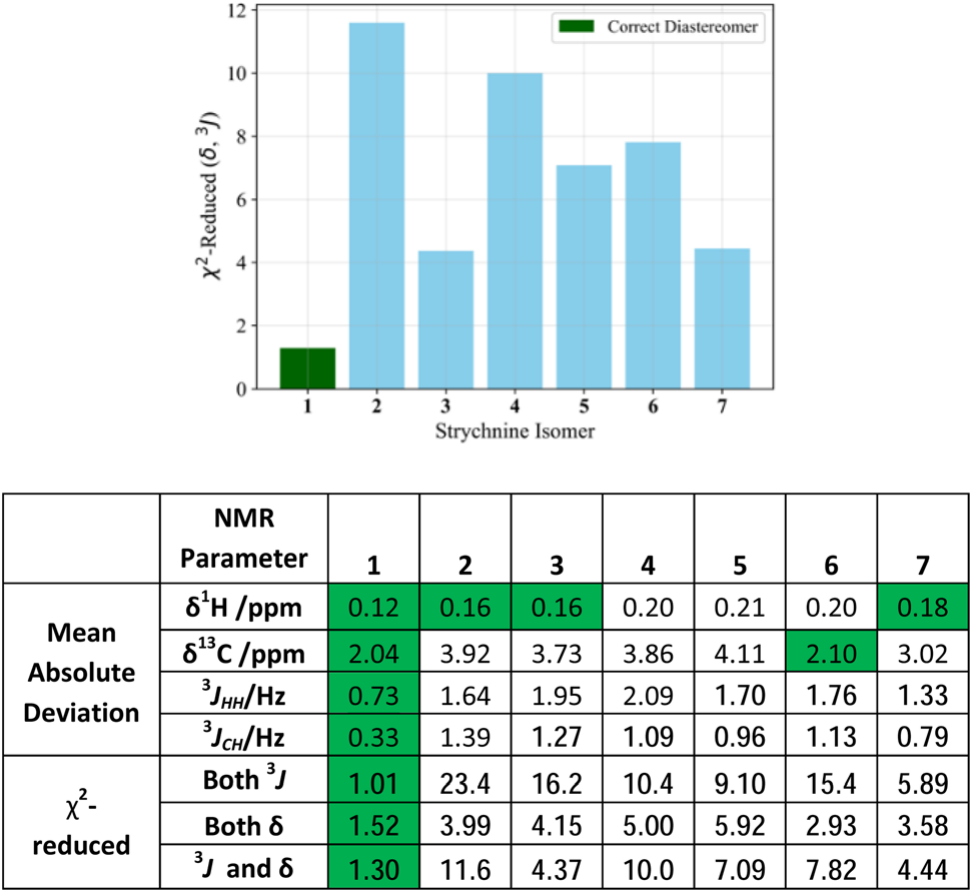
Statistical measures of IMPRESSION-G2 predictions of chemical shifts and scalar couplings for strychnine diastereomers, compared to those reported for the natural product. Entries highlighted in green are those within 50% of the best value for each analysis.

The MAD between IMPRESSION-G2 predictions and experimental values for ^1^H- and ^13^C-based NMR parameters for diastereomers of strychnine, 1–7, that were found here to be stable by computation are shown in the table in Fig. 3. In every case, the correct diastereomer, 1, has the best fit, however if one only considers chemical shift then there is a lack of certainty, with other plausible fits also having low average deviations close to the performance limits of IMPRESSION-G2 (highlighted in green where MAD <0.2 ppm for ^1^H and <3 ppm for ^13^C). Using predicted coupling constants provides much more effective discrimination of diastereomers, with both ^1^H–^1^H and ^1^H–^13^C scalar couplings suggesting only diastereomer 1 as a plausible solution. An alternative analysis using the more discriminating *χ*^2^-reduced statistic, reinforces this finding. *χ*^2^-Reduced should provide values close to 1 for good fits and ideally values >2 for incorrect structures (see ESI† for details). Here, the *χ*^2^-reduced achieved with the combined ^1^H–^1^H and ^1^H–^13^C coupling constants offers a very clear fit for 1 (*χ*^2^-reduced = 1.01) with the next best option being 7, which can be definitively excluded based on a six-fold higher *χ*^2^-reduced of 5.89. By contrast, the differentiation if only considering chemical shifts is much less, with less than 2-fold discrimination between diastereomers 1 and 6 (1.52 and 2.93). Unsurprisingly, the combination of *J* and *δ* together also provides a clear discrimination between the correct structure, 1, and all other options and this is clearly illustrated in Fig. 3, top.

## Conclusions

In summary, IMPRESSION-G2 predicts multiple NMR parameters simultaneously and accurately with a single model. When combined with the computational efficiency of GFN2-xTB geometry optimisation this system offers comparable accuracy, but with orders of magnitude improvement in computational efficiency and time-savings, compared to DFT.

In common with other machine learning systems for NMR prediction, it achieves the highest accuracy when tested against internal hold-outs from its own training dataset of molecules, but crucially IMPRESSION-G2 also provides excellent accuracy for molecules within its chemical space (C, H, N, O, F, Si, P, S, Cl, Br; *M*_R_ < 1000 g mol^−1^) that are sourced entirely independently of its training data. IMPRESSION-G2 reproduces experimental data with error distributions that are comparable to those achievable by DFT, and can provide similarly excellent diastereomeric discrimination to DFT, but in seconds rather than hours. Consequently we believe IMPRESSION-G2 is the first plausible machine learning replacement for DFT for the prediction of 3D-sensitive NMR parameters, with time-savings that make it possible to predict millions of parameters for thousands of structures in minutes.

## Data availability

The data presented in this work is available at: https://github.com/orgs/Buttsgroup/repositories.

## Author contributions

C. Y. led the architecture and code improvements, generated, curated and enhanced the bulk of the training sets used for IMPRESSION-G2, and conducted the testing against computational and experimental datasets. B. H. integrated GFN-xTB and conducted the diastereomer analysis. W. G. developed the original code for IMPRESSION-G2, adapted from ref. 28. J. N.-F., D. R., I. T., R. D. supported development of the ideas used in this program, co-supervised C. Y. and B. H. C. B. designed, developed and leads the IMPRESSION research programme and supervised C. Y., B. H. and W. G. The manuscript was co-written by C. B. C. Y. and B. H. with edits and comments from other authors.

## Conflicts of interest

There are no conflicts to declare.

## Supplementary Material

SC-OLF-D4SC07858F-s001
