# Supplementary material for: IMPRESSION generation 2 – accurate, fast and generalised neural network model for predicting NMR parameters in place of DFT
Source: Chem Sci. 2025 Mar 31;16(19):8377–82. doi: 10.1039/d4sc07858f (PMC11983320; doi:10.1039/d4sc07858f)
Supplement: SC-016-D4SC07858F-s001 [file SC-016-D4SC07858F-s001.pdf]

Supplementary Information (SI) for Chemical Science.
This journal is © The Royal Society of Chemistry 2025

Supplementary Information for IMPRESSION Generation 2 – Accurate, fast and generalised neural network model for predicting NMR parameters in place of DFT.

Calvin Yiu, Ben Honoré, Will Gerrard, Jose Napolitano-Farina, Ruth Dooley and Craig P. Butts

Table of Contents

STATEMENT ON SOFTWARE AVAILABILITY....3

S1 METHODS....3

S1.1 Graph Transformer Network....3
S1.1.1 Graphs....3
S1.1.2 Multi-headed[1, 2] attention....3
S1.1.3 Lamb[5] Optimiser....5
S1.2 IMPRESSION Generation 2 (IMP-G2)....5
S1.2.1 Data preparation....5
S1.2.2 IMP-G2 – Architecture....6
S1.2.3 IMP-G2 - Hyperparameters....7
S1.3 Training and testing data....8
S1.3.1 Diversity sampling....8
S1.3.2 DFT NMR parameter calculation workflow....8
S1.3.3 Chemical Space covered by Training Dataset....10

S2 IMPRESSION-G2 PREDICTION AGAINST DFT CALCULATED VALUES....15

S2.1 Chemical Shifts....15
S2.1.1 δ^1H....15
S2.1.2 $\delta^{13}C$....18
S2.1.3 $\delta^{15}N$....21
S2.1.4 $\delta^{19}F$....24
S2.2 Scalar couplings....26
S2.2.1 $^1J_{CH}$....26
S2.2.2 $^2J_{CH}$....29
S2.2.3 $^3J_{CH}$....32
S2.2.4 $^4J_{CH}$....35
S2.2.5 $^2J_{HH}$....38
S2.2.6 $^3J_{HH}$....41
S2.2.7 $^4J_{HH}$....44
S2.2.8 $^1J_{CC}$....47
S2.2.9 $^2J_{CC}$....50
S2.2.10 $^3J_{CC}$....53
S2.2.11 $^4J_{CC}$....56
S2.2.12 $^1J_{NH}$....59
S2.2.13 $^2J_{NH}$....62
S2.2.14 $^3J_{NH}$....65
S2.2.15 $^4J_{NH}$....68
S2.2.16 $^1J_{NC}$....71
S2.2.17 $^2J_{NC}$....74
S2.2.18 $^3J_{NC}$....77
S2.2.19 $^4J_{NC}$....80
S2.2.24 $^2J_{FH}$....83

S2.2.25 $^3J_{FH}$ *85*
S2.2.26 $^4J_{FH}$ *87*
S2.2.27 $^1J_{FC}$ *89*
S2.2.28 $^2J_{FC}$ *91*
S2.2.29 $^3J_{FC}$ *93*
S2.2.30 $^4J_{FC}$ *95*
S2.2.31 $^2J_{FN}$ *97*
S2.2.32 $^3J_{FN}$ *99*
S2.2.33 $^4J_{FN}$ *101*
S2.2.34 $^2J_{FF}$ *103*
S2.2.35 $^3J_{FF}$ *105*
S2.2.36 $^4J_{FF}$ *107*

S3 COMPARISON OF IMPRESSION GENERATION 2 ERRORS BETWEEN VARIOUS DFT METHODS ON CHESHIRE TEST SET **109**

S4 EXPERIMENTAL COMPARISON **110**

S4.1 STRYCHNINE 110
S4.2 EXP5K 112
S4.3 CHESHIRE TEST SET IMPRESSION/DFT TO EXPERIMENT COMPARISON 113

***2-CYANOPROPANE, T-BUTYLACETYLENE REMOVED FROM CHESHIRE TEST SET AS DFT CALCULATIONS FAILED DURING TESTING.S5. SAMPLED STRUCTURES** **113**

S5.1 CSD STRUCTURES 113
S5.2 CHEMBL STRUCTURES 136
S5.3 OTAVA STRUCTURES 158

S5 REFERENCE **212**

Statement on Software and Data availability

The IMPRESSION software is temporarily available at https://www.impression-nmr.com/ but may have limited capacity or be inaccessible while the authors finalise this webtool for use. Once finalised, this Statement will be updated to identify avenues to accessing the software for academic and non-academic purposes.

The data presented in this work is available at: https://github.com/orgs/Buttsgroup/repositories

S1 Methods

S1.1 Graph Transformer Network

Graph Transformer Network[1] (GTN) is the implementation of the transformer[2] architecture into graphs data, enabling the use of the attention mechanism to evaluate interactions between nodes of different labelled data type. In the context of NMR parameter prediction, this allows the weighting of relationships between chemical shifts and coupling constants between different environments (e.g. the local environment of a $\delta^{13}C$ shift in an environment based on neighbouring atoms and atom-pair interaction over a given distance in space). This implementation of the GTN is an adapted version of the solution presented in the CHAMMPS Kaggle competition[3].

S1.1.1 Graphs

A natural representation of a given molecule is a chemical graph, this data can then be directly converted to an input computational graph. This can be represented by the molecules as a graph (G) with atoms as a set of nodes (V) feature vectors and bonds as a set of edges (E), typically in the form of an adjacency matrix but not always, which corresponds to:

$$G = (V,E)$$

However, since IMP-G2 target values contain coupling constants for distances of up to six-bond, we generate edges (E) for all atoms within 6-bond hops away, this results in a partially connected graph.

In IMP-G2, the node only contains information about the atom type, both as a numeric and character representation, with the attached label being the associated chemical shift. The edge contains information about the atom-pair interaction which are: shortest number of direct bonds away (up to 6), and the associated coupling constant label (e.g. '3JCH'), the attached label to the edges contains the coupling constant for this specific interaction. Finally, instead of

an adjacency matrix, we use a distance matrix for edges recording absolute distance in Angstroms for atoms within six bonds of one another, and zero for distances beyond that.

S1.1.2 Multi-headed[1, 2] attention

Multi-headed attention[2] is the fundamental feature which allows the transformer to resolve complex relationships. Functionally, it is the aggregation of multiple attention heads into a single final feature vector, each single attention head (k) focuses on information from a different target label.

For each head (k) we have the following steps:

1. Linear Transformation

   $h_i^k = W^k h_i$

   Where for node (i), with the feature vector (h) containing the node information, is multiplied by a weight matrix (W) associated with this attention head. The weight matrix (W) is a parameter adjusted through training. Resulting in the updated node feature vector of h_i^k.

2. Attention coefficient, e_{ij}

   $$e_{ij}^k = LeakyReLU(a^k[h_i^k||h_j^k])$$

   The attention coefficient (e_{ij}) is a measure of the contributions for a given neighbour (j) with respect to the focus of the attention head (i.e. how much this interaction is weighted when considering the chemical environment of a given atom.) for the node of interest (i). Using the transformed feature vectors, the features are concatenated together and then multiplied by a learnable parameter vector (a^k), specific to the attention head (k). The calculated value is then activated with LeakyReLU[4] to return the same value if it's positive or reduced negative values by scaling if it's negative.

3. Normalised attention coefficient α_{ij}:

   $$\alpha_{ij} = \frac{exp(e_{ij})}{\sum_{k \epsilon N(i)} exp(e_{ik})}$$

   The normalised attention coefficient for a given atom-pair interaction is a value between 0 and 1 directly correlating the weighted contribution of said interaction with a higher value being of greater importance. This is calculated by taking the exponent of the target attention coefficient of interest and dividing it by the sum of all attention coefficient present for atom (i) against the nodes in set k. The exponential is included to

exemplify higher calculated attention coefficients. As this is a division against a set of given data, the summation of all normalised attention coefficients α_{ij}, equated to one for a given atom.

$$\sum_{j \in N(i)} \alpha_{ij} = 1$$

4. Aggregation and activation:

$$\sum_{j \in N(i)} \alpha_{ij} W h_j$$

The updated feature vector, $h_i^{'}$, after a pass through the attention head is calculated by summing all the feature neighbouring node vectors multiplied by their respective attention coefficients, where the weight is the attention coefficient calculated.

$$h_i^{k'} = \sigma\left(\sum_{j \in N(i)} \alpha_{ij} W h_j\right)$$

This is then passed to an activation function σ, to introduce non-linearity so inherent features can be derived in back-propagation. The updated feature vector is now $h_i^{k'}$ for node i after passing through attention head k.

This process is applied for each attention head present in multi-headed attention. The final feature vector is measured through either the concatenation or averaging of the values for each given attention head. For IMP-G2 we use averaging:

$$h_i^{'} = \frac{1}{K} \sum_{k=1}^{K} h_i^{k'}$$

Where k is a given attention head, and K is all attention head present. The final feature, $h_i^{'}$ vectors contain the total sum of interaction accounting for all attention heads in representing the available features. For IMP-G2 with NMR parameter prediction, this equates to an attention head looking at each possible interaction from all given NMR parameters. Enabling the weighted contribution of each connected atom and edge interaction to evaluate a given NMR parameter's environment.

S1.1.3 Lamb[5] Optimiser

We used the Lamb optimiser for the training optimisation for IMP-G2. This allows the optimiser to assign different learning rates per layer, providing improved stability and adaptive updates per parameter to prevent drastic changes in step sizes. In general, it has been shown to improve scalability of both batch sizes and model layer depth.

S1.2 IMPRESSION Generation 2 (IMP-G2)

S1.2.1 Data preparation

Prior to training the molecules are converted to graphs using DGL with atoms to nodes, and edges representing through space interaction, we only generate edges for nodes within 6 bonds of one another. We store the atom type as an integer value (H = 1, C = 6,…), with DFT calculated chemical shifts under 'Shift' as the label for ground truth.
For edges we features we store, the 'nmr_type' after it has been mapped based on the desired coupling constant to be trained on as this directly relates to the number of attention heads required; Distances as 'dist', the absolute distance from one atom to another in Angstroms; Path length as 'path_len' corresponding the number of bond hops away one atom is from another; finally a 'coupling' label to store coupling constant as the ground truth for loss calculation.

All chemical shifts and coupling are normalised with respect to each other to values between 0 and 1. Values are subsequently descaled after predictions.

S1.2.2 IMP-G2 – Architecture

IMP-G2 is an adapted version of the GTN using attention to weight the contribution of each NMR parameter with respect to the predicted chemical shift or coupling constant. Here we outline the architecture, comprising layers, hyperparameters, and data generation pipeline.

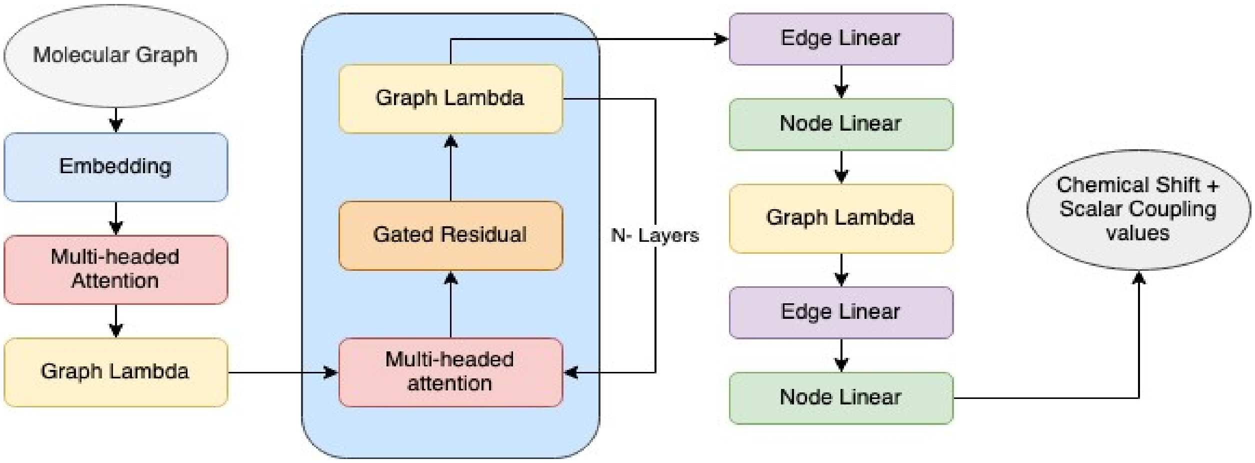

Figure S1. Flowchart of layers within the IMPRESSION-G2 model.

IMPRESSION Generation 2 (Figure S1) contains the following layers:

1. **Embedding**
   The embedding layer is effectively tokenisation applied to the Graph Transformer Network, enabling the projection of node and edge features into a matrix representation of a predetermined dimension, desired predictions are mapped onto a specific row corresponding to a target flag (NMR parameter). Node embedding

dimensions are 61 x 48, edge embeddings are 1000 x 48. This produces the same dimensionality for all graphs, standardising the input format.

2. **Multi-headed attention**
   Implementation of multiheaded attention (Section S1.1.2) layer with an attention head corresponding to each NMR parameters present in the graph. In IMP-G2 we utilise 42 attention heads.
3. **Graph Lambda**
   Layer updating node and edge features with reshaped outputs from previous attention weighted interaction and/or gated residual layers to fit the layer dimensions.
4. **Gated Residual[6]**
   Enables data to flowthrough prior to multi-headed attention layers for the model to evaluate the effect of attention on the updated representation and correlation to NMR parameters in this instance.
5. **Attention Block**
   The repeating block of IMP-G2 where the effects of attention layers are culminated. It consists of the multi-headed attention layer, gated residual layer, and a graph lambda layer.
6. **Node and Edge Linear**
   Linear layers of neurons to process the latent embeddings updated by multiheaded attention to the final output values of chemicals shifts (node layers) and coupling constants (edge layers). The first set of node and edge layers takes in input dimensions of 48 x 42 (embedding dimension by attention heads) to a 512 x 1 dimension, the second layer of linear layers takes the 512 x 1 into 42 x 1 for final output with each of those 42 values corresponding to the specific NMR parameters as mapped by the attention heads from the initial target labels.

We use L1 regularisation/loss (Mean absolute error) as the metric for determining loss during training.

S1.2.3 IMP-G2 - Hyperparameters

The following are the fundamental tune-able hyper-parameters for the model:

- Training epochs: **2000**
- Number of attention heads: **42**
- Number of layers (attention block): **4**
- Effective batch size: **16** (batch size, **2** * gradient accumulation, **8**)
- Targetflags (Predictable NMR parameters): [**'HCS', 'CCS', 'NCS', 'FCS', '1JCH', '1JCC', '1JNH', '1JNC', '1JFC', '2JCH', '2JHH', '2JCC', '2JNH', '2JNC', '2JFH', '2JFC', '2JFN', '2JFF', '3JCH', '3JHH', '3JCC', '3JNH', '3JNC', '3JFH', '3JFC', '3JFN', '3JFF', '4JCH', '4JHH', '4JCC', '4JNH', '4JNC', '4JFH', '4JFC', '4JFN', '4JFF'**]

Training epochs are how many full passes of training data the model sees; attention heads correspond to the number of heads in multi-headed attention (Section S1.1.2); layers are the repeating block as described in S1.2.2; effective batch size is the number of graphs (molecules)

the model sees at a time before updating weights and also how many graphs the models sees at one time during prediction – this is calculated through the batch size multiplied by the gradient accumulation value; targetflags are the labels of the NMR parameters to be predicted, the architecture of the model means that the number of attention heads must be greater than the number of targetflags as each labels corresponding to a prediction typed are mapped onto individual attention heads.

S1.3 Training and testing data

S1.3.1 Diversity sampling

All molecules we use in our training data were adaptively sampled from large databases. These consist of Cambridge Structural Database (CSD), ChEMBL, and OTAVA.

S1.3.2 DFT NMR parameter calculation workflow

In order to train and test IMPRESSION, we developed a dataset of NMR parameters computed using DFT in the Gaussian16 software package.[7] We conducted a literature review and independent benchmarking of a wide range of functionals and basis sets and found that there is no best single method that is *both* optimally accurate and optimally computationally efficient for predicting *both* chemical shifts and scalar coupling constants for *both* ^{1}H and ^{13}C nuclei. However there are many combinations which are good or near-best in several of those respects and alternative DFT methods would have provided very similar results (see S3.3 for evidence of this point),so the exact choice of which DFT method to choose for the IMPRESSION-G2 training set is not considered especially limiting unless a far superior DFT methodology is forthcoming, at which point retraining IMPRESSION-G2 using such training data would be sensible.

From this benchmarking we selected mPW1PW91/6-311g(d,p) for optimisation and ωb97xd/6-311g(d,p)[8–12] for computing both chemical shifts and scalar coupling constants as these were amongst the most computationally efficient methods that gave accurate results for both ^{1}H and ^{13}C shifts and couplings.

S1.3.2.2 GFN-xTB[9]

The semi empirical quantum chemical method GFN2-xTB[15] was used as another method of geometry optimisation. In each case, the Approximate Normal Coordinate Rational Function Optimizer was used (ANCOPT) with default parameters.

- Optimisation level: **normal**
- Maximum Optimisation Cycles: **200**
- ANC Micro Cycles: **20**

- RF Solver: **Davidson**
- Energy Convergence: **0.5x10-5 Eh**
- Gradient Convergence: **0.1x10-2 Eh/α**
- Maximum RF Displacement: **1.0**
- HLow Frequency Cut Off: **0.1x10-1**
- HMax Frequency Cut Off: **5.0**
- S6 in Model Hessian: **20.0**

S1.3.2.3 DFT Geometry Optimisation

Following initial geometry optimisation (Molecular Mechanics Section S1.3.2.1 for the generation of our training molecules) as highlighted in the main article, we carried out DFT geometry optimisation using Gaussian16 to get refined input coordinates. Here are the starting block and route line parameters used for all DFT geometry calculations for the training/testing data we generated:

```
%Chk={molecule_name}_OPT.chk
%NoSave
%mem=26GB
%NProcShared=8
# opt=tight freq mPW1PW/6-311g(d,p) integral=ultrafine MaxDisk=50GB
```

S1.3.2.4 DFT NMR Tensor and Scalar Coupling Constant Calculation

The optimised geometries are then extracted from the Gaussian16 output log files and used as input coordinates for the DFT NMR Tensor and Coupling Constant calculations. The NMR parameters were calculated using gauge independent atomic orbitals with uncontracted basis sets to improve descriptions of the core orbitals[30] and calculation of all components of the scalar couplings (Fermi contact, spin dipole, diamagnetic spin orbit, paramagnetic spin orbit). Here are the following block and route line parameters used for all DFT NMR parameters calculation for the training/testing data we generated:

```
%Chk={molecule_name}_NMR.chk
%NoSave
%mem=26GB
%NProcShared=8
#T nmr(giao,spinspin,mixed) wB97XD/6-311g(d,p) maxdisk=50GB
```

S1.3.2.5 Chemical Shift Scaling of DFT Tensors

As DFT calculates tensors, the values need to be linearly scaled to their chemical shift counterpart. This is done through comparison of DFT tensors against experimentally measured

chemical shifts and calculating the slope (m) and linear correction (c) typical of the y = mx+c equation of a linear line, where x is the tensor and y is the chemical shift.

$$\delta_{EXP} = \frac{\sigma_{DFT} - c}{m}$$

For ^{1}H and ^{13}C, we used the CHESHIRE reference set to calculate our scaling factors which were.

- ^{1}H = -1.0594, 32.2293 for m, c
- ^{13}C = -1.0207, 187.4436 for m, c

^{15}N, ^{19}F, and ^{31}P were gathered from literature with matching functional group and basis set[16, 17].

- ^{15}N = -1.0139, -148.67 for m, c
- ^{19}F = -1.0940, 173.02 for m, c
- ^{31}P = -1.2777, 307.74 for m, c

S1.3.3 Chemical Space covered by Training Dataset

The IMPRESSION-G2 training dataset covers 18182 organic molecules listed in Section S4. These molecules have masses primarily in the range of 50-1000 g/mol, contain C, H, N, O, F, Si, P, S, Cl, Br, and comprises 739,913 chemical shift environments (330,411 $\delta^{1}H$; 306,458 ^{13}C) and 5,696,784 scalar coupling constants (including 307,270 $^{1}J_{CH}$; 486,884 $^{2}J_{CH}$; 672,433 $^{3}J_{CH}$; 705,737 $^{4}J_{CH}$; 134,051 $^{2}J_{HH}$; 217,940 $^{3}J_{HH}$; 333,010 $^{4}J_{HH}$) in addition to corresponding NMR parameters for all other NMR-active nuclei in these molecules. While there is no entirely robust way to ensure diversity in a molecular dataset, various subsets of the data were selected using different diversity sampling methods, including Tanimoto analyses, cross-validation and by including the CHEMBL diversity-sampled dataset. The resulting structural and chemical space can be approximated by visualizing the mass, chemical shift, hybridization and scalar coupling constant distributions of the molecules, as seen here:

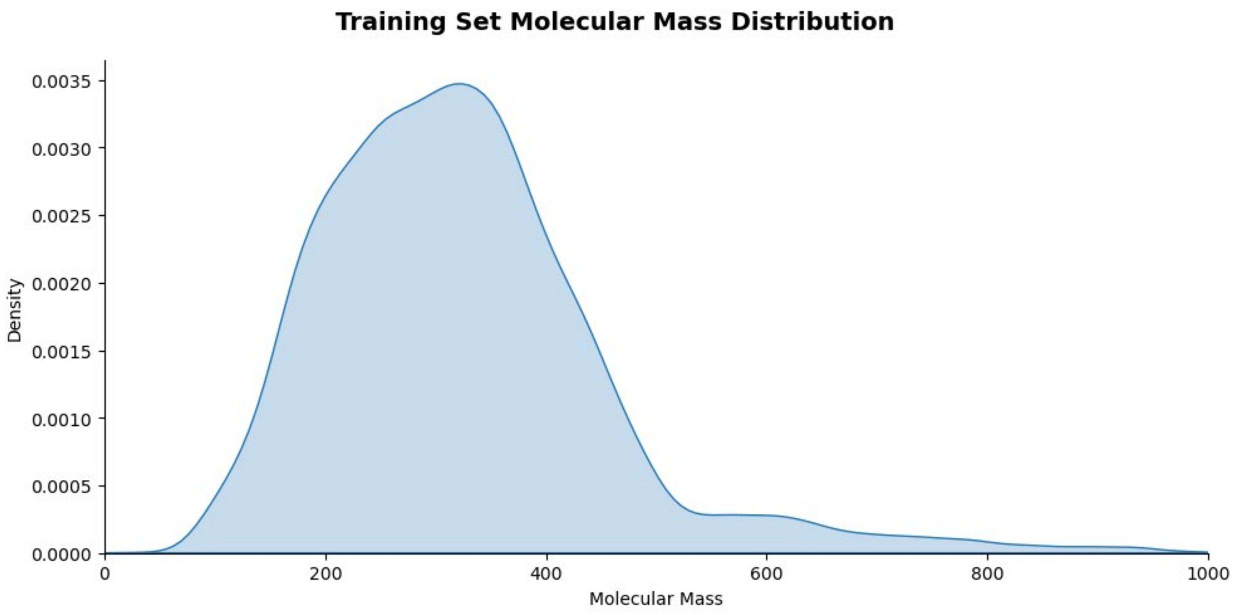
Training Set Molecular Mass Distribution
Density
0.0035
0.0030
0.0025
0.0020
0.0015
0.0010
0.0005
0.0000
0
200
400
600
800
1000
Molecular Mass

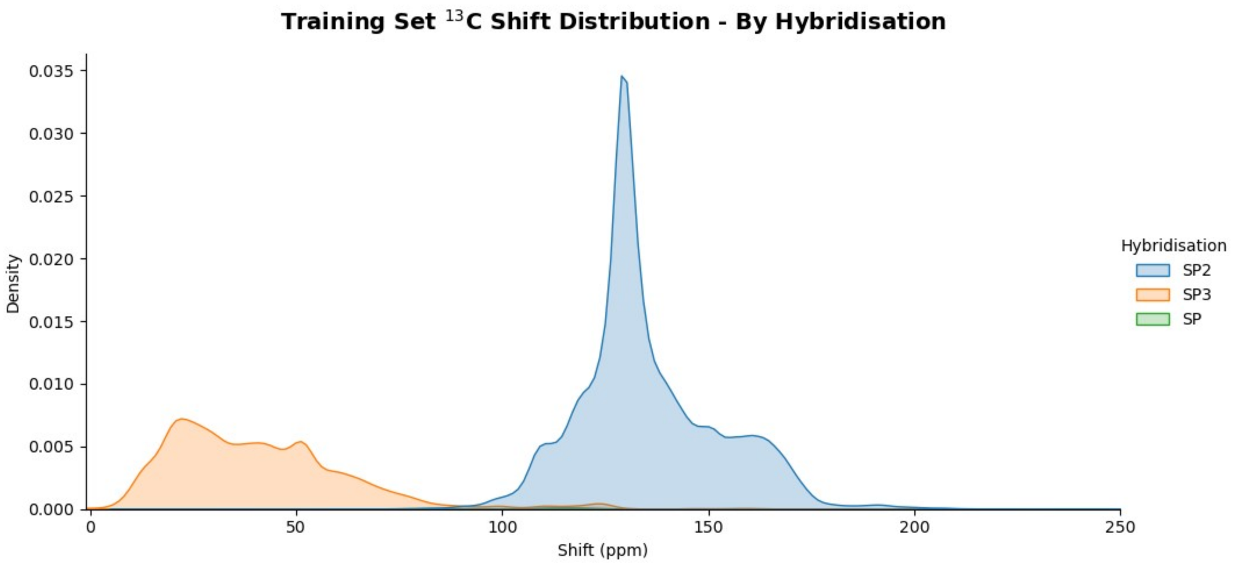
Training Set ^{13}C Shift Distribution - By Hybridisation
Density
0.035
0.030
0.025
0.020
0.015
0.010
0.005
0.000
0
50
100
150
200
250
Shift (ppm)
Hybridisation
SP2
SP3
SP

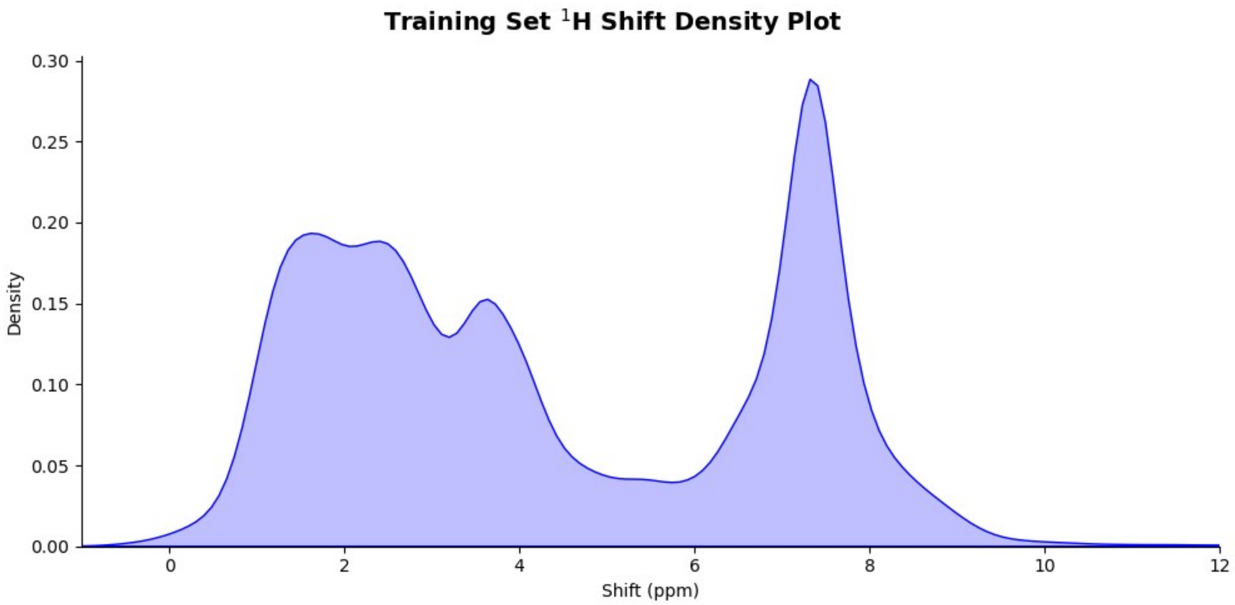
Training Set ^{1}H Shift Density Plot
Density
0.00
0.05
0.10
0.15
0.20
0.25
0.30
Shift (ppm)
0
2
4
6
8
10
12

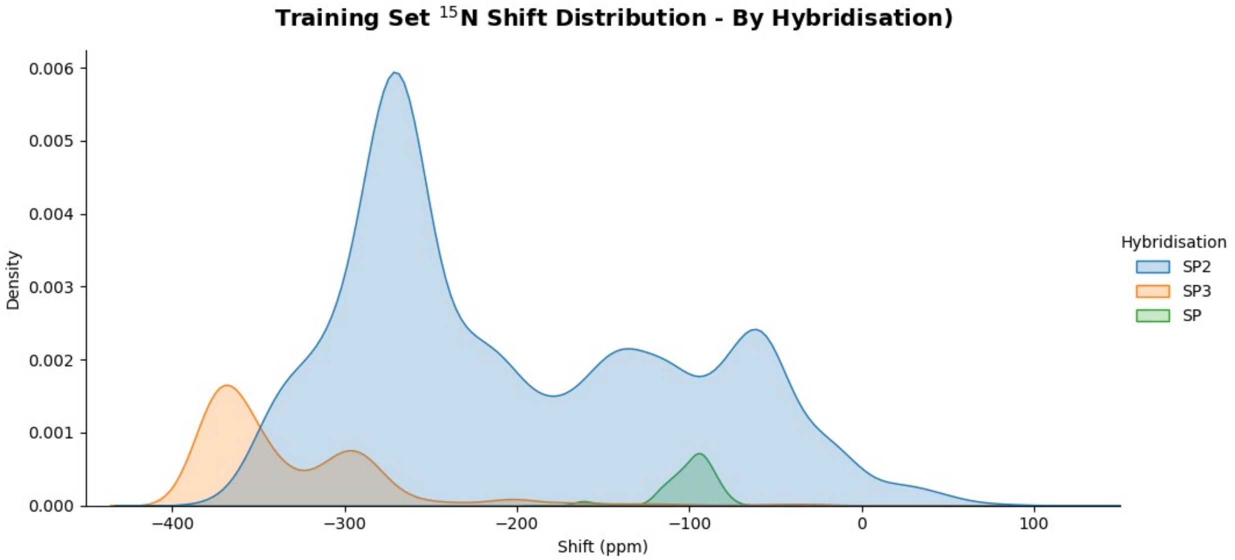
Training Set ^{15}N Shift Distribution - By Hybridisation)
Density
0.000
0.001
0.002
0.003
0.004
0.005
0.006
Shift (ppm)
−400
−300
−200
−100
0
100
Hybridisation
SP2
SP3
SP

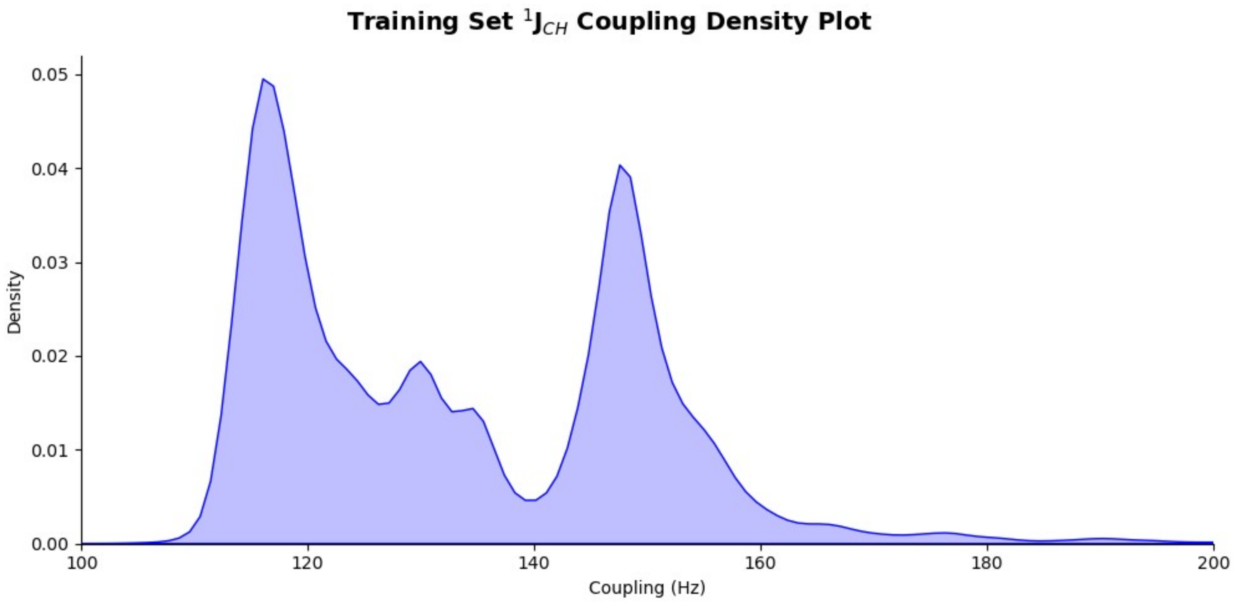
Training Set $^{1}J_{CH}$ Coupling Density Plot
Density
0.05
0.04
0.03
0.02
0.01
0.00
100
120
140
160
180
200
Coupling (Hz)

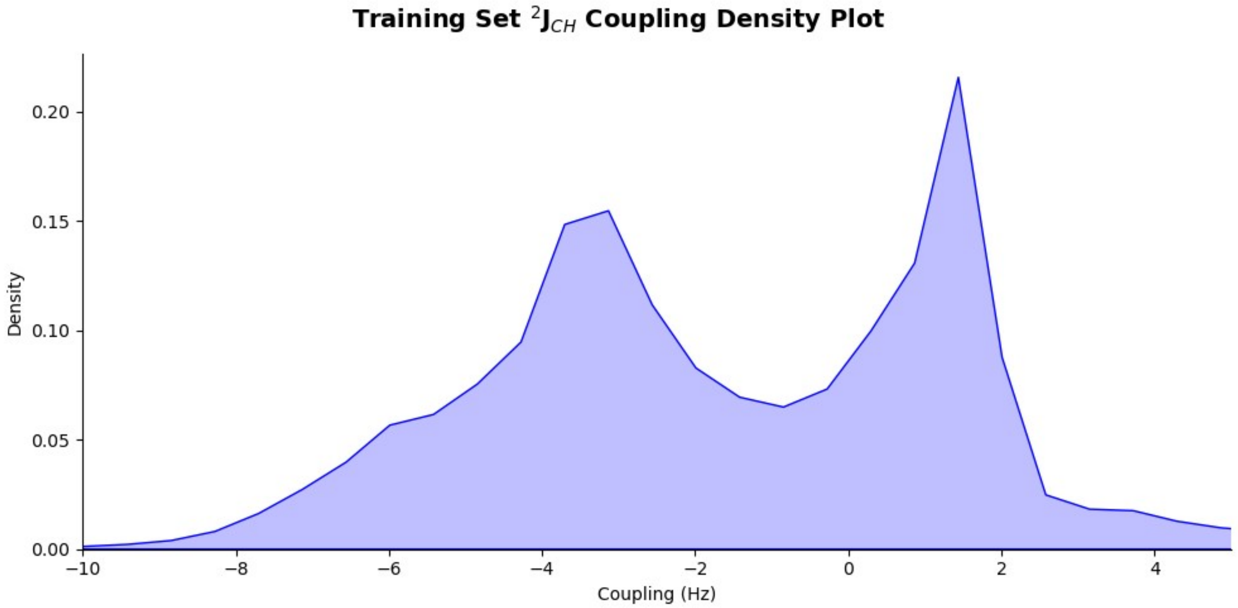
Training Set $^{2}J_{CH}$ Coupling Density Plot
Density
0.20
0.15
0.10
0.05
0.00
−10
−8
−6
−4
−2
0
2
4
Coupling (Hz)

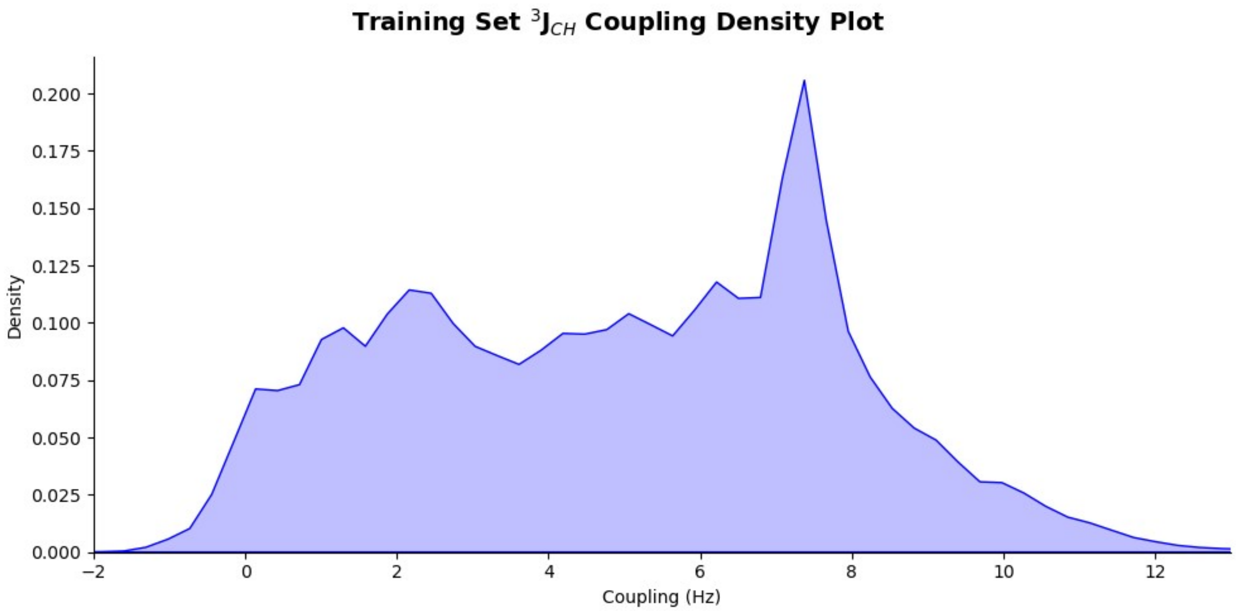

Training Set $^3J_{CH}$ Coupling Density Plot
Density
0.200
0.175
0.150
0.125
0.100
0.075
0.050
0.025
0.000
−2
0
2
4
6
8
10
12
Coupling (Hz)

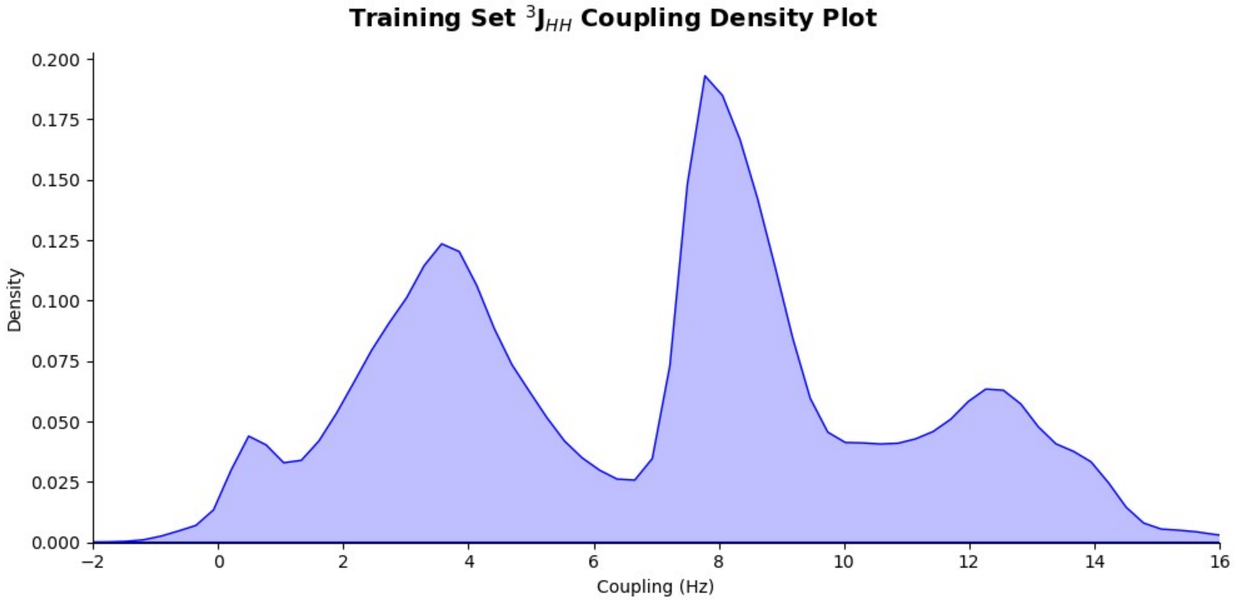

Training Set $^3J_{HH}$ Coupling Density Plot
Density
0.200
0.175
0.150
0.125
0.100
0.075
0.050
0.025
0.000
−2
0
2
4
6
8
10
12
14
16
Coupling (Hz)

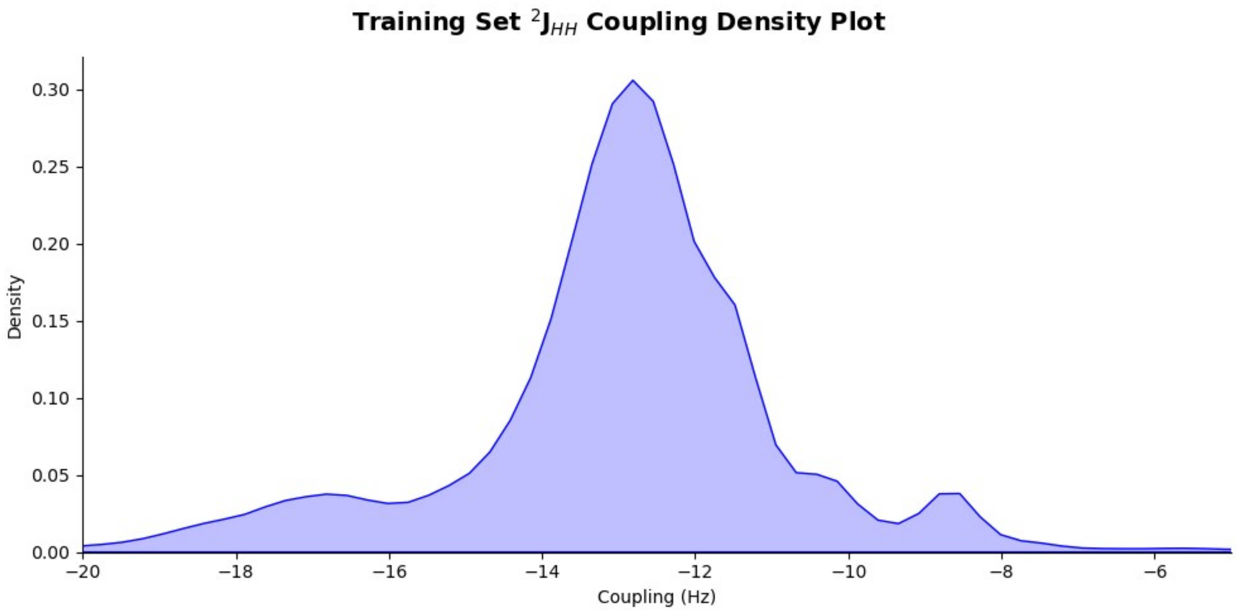
Training Set $^{2}J_{HH}$ Coupling Density Plot
Density
0.30
0.25
0.20
0.15
0.10
0.05
0.00
−20
−18
−16
−14
−12
−10
−8
−6
Coupling (Hz)

S2 IMPRESSION-G2 prediction against DFT calculated values

Here we present all of IMP-G2 predictions against DFT calculated values, where Chemical Tensors have been scaled to Chemical Shifts, and Scalar Couplings are taken as is from: the holdout set -10% of withheld structures from the total number of molecules in the training set, Data3 – Testing set from the original IMPRESSION[18] paper, DFT8K_bg – DFT calculated values from DFT8K[19] structures, recalculated using our DFT functional and basis set.

S2.1 Chemical Shifts

S2.1.1 δ^{1}H

Holdout

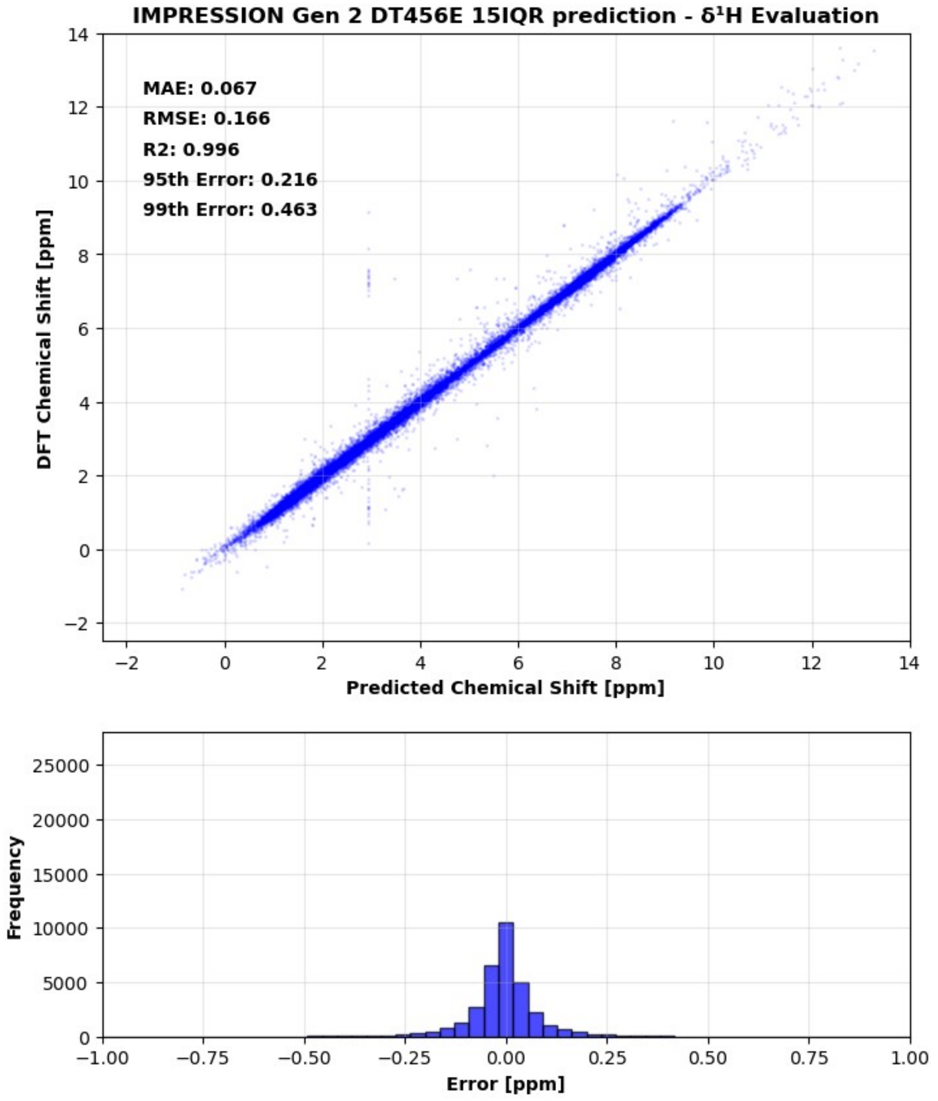

Dataset 3

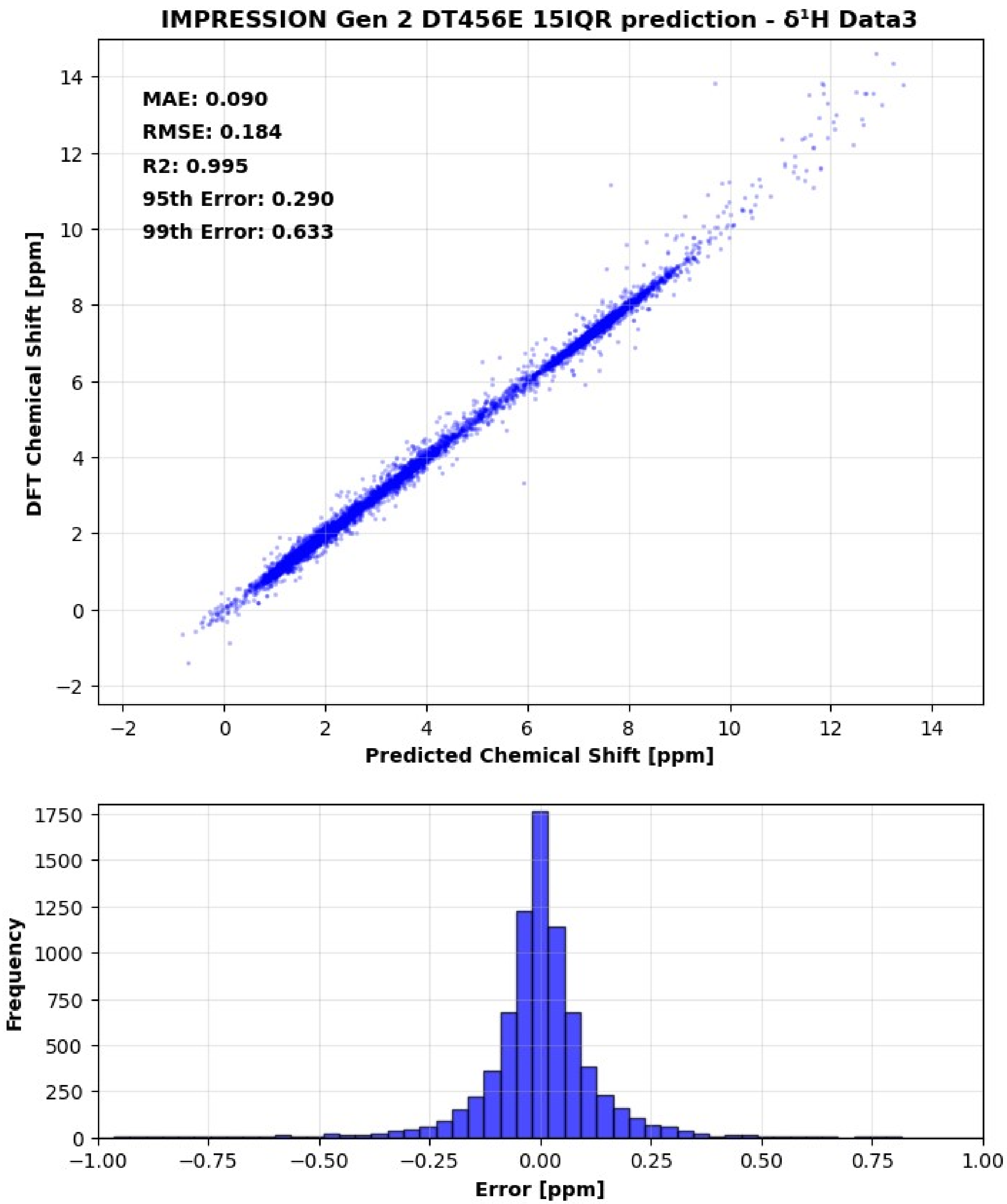
IMPRESSION Gen 2 DT456E 15IQR prediction - δ1H Data3
MAE: 0.090
RMSE: 0.184
R2: 0.995
95th Error: 0.290
99th Error: 0.633
DFT Chemical Shift [ppm]
Predicted Chemical Shift [ppm]
Frequency
Error [ppm]

DFT8K_bg

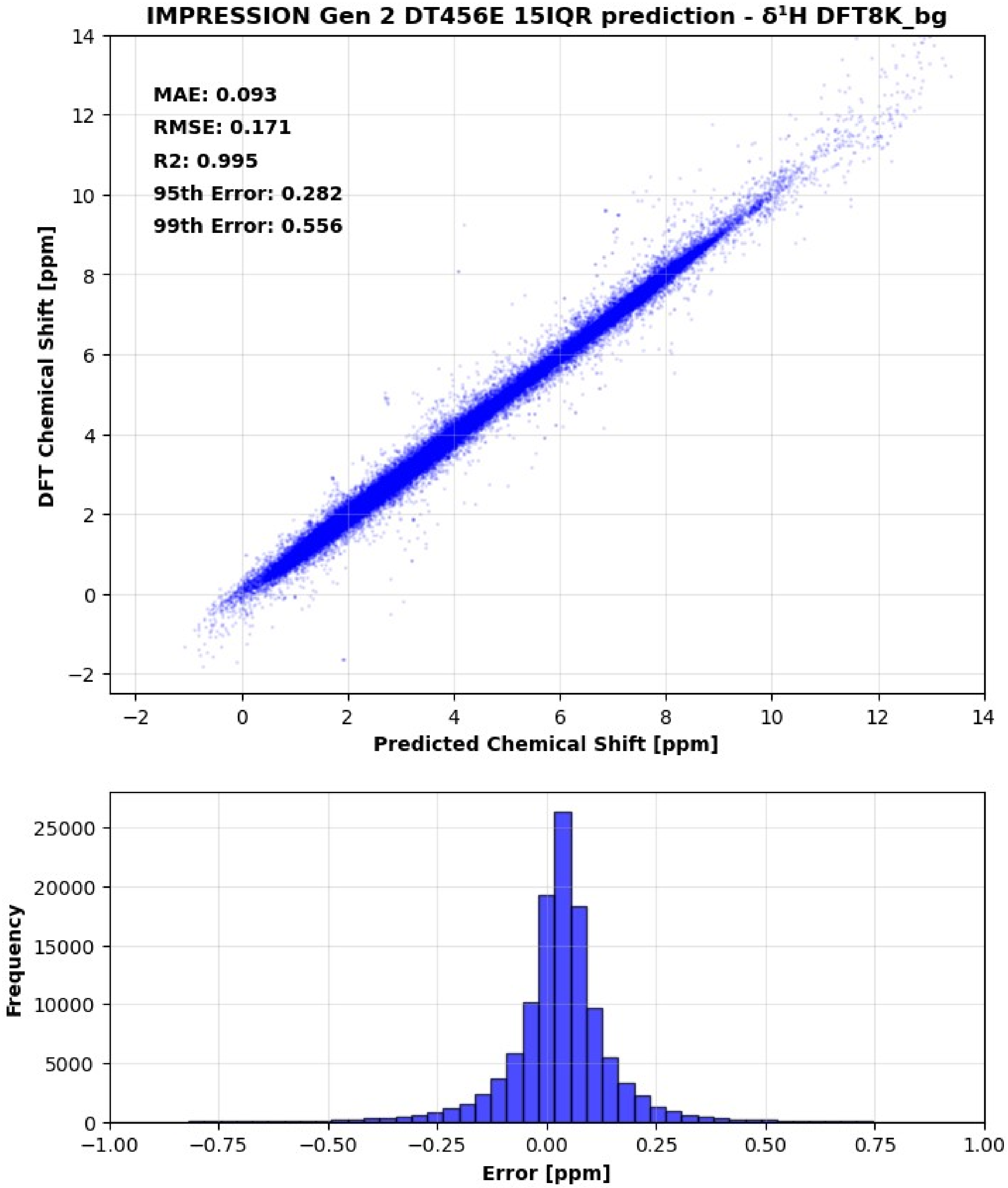

IMPRESSION Gen 2 DT456E 15IQR prediction - δ¹H DFT8K_bg
MAE: 0.093
RMSE: 0.171
R2: 0.995
95th Error: 0.282
99th Error: 0.556
DFT Chemical Shift [ppm]
Predicted Chemical Shift [ppm]
Frequency
Error [ppm]

S2.1.2 δ^{13}C

Holdout

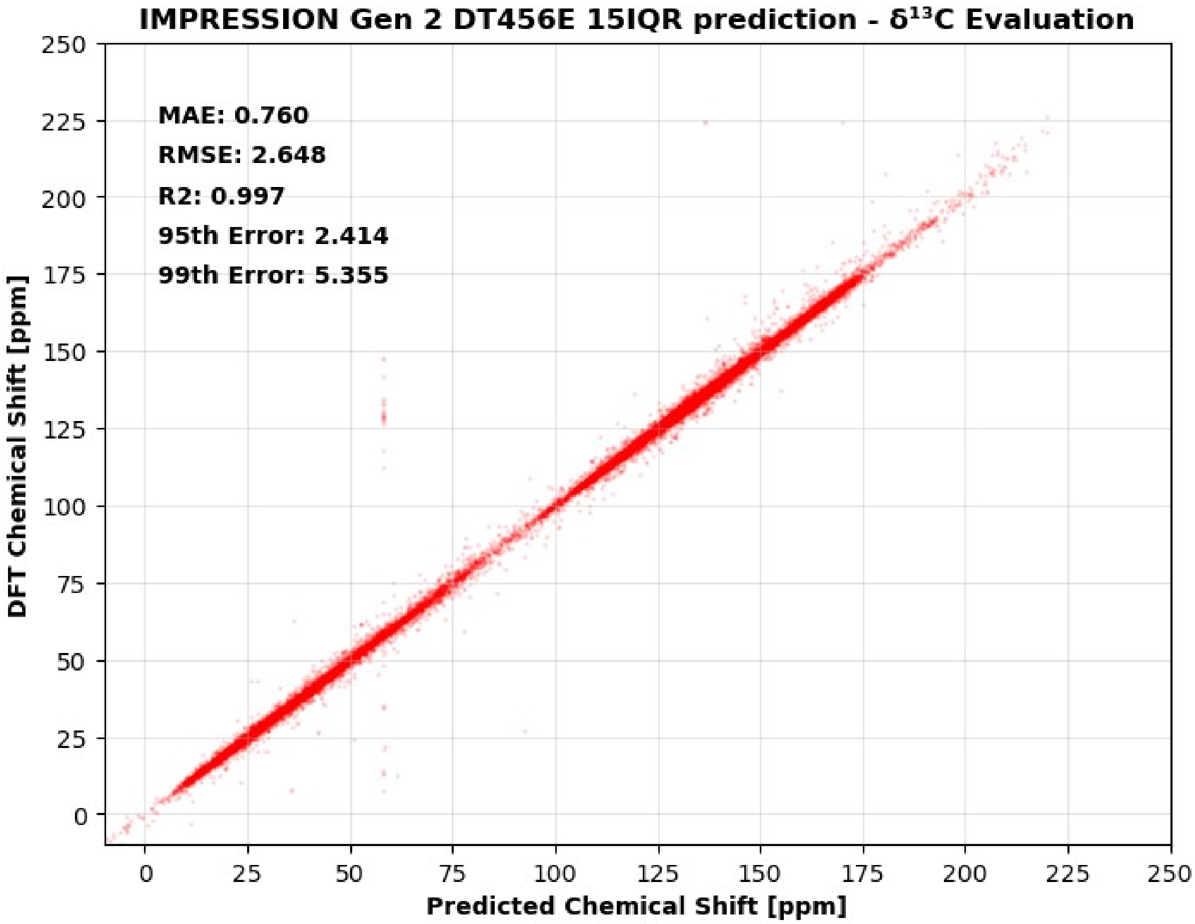

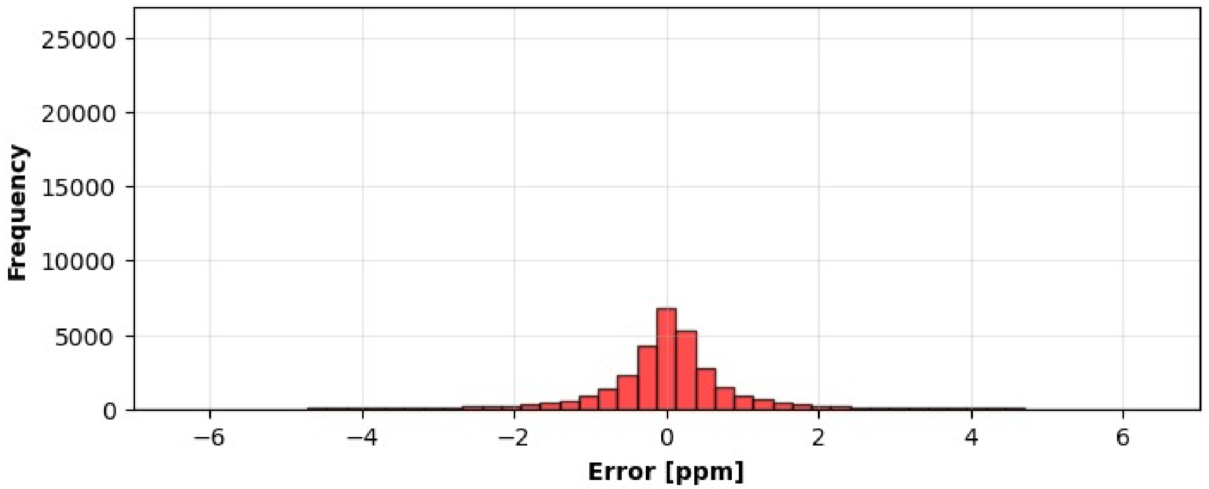

Data3

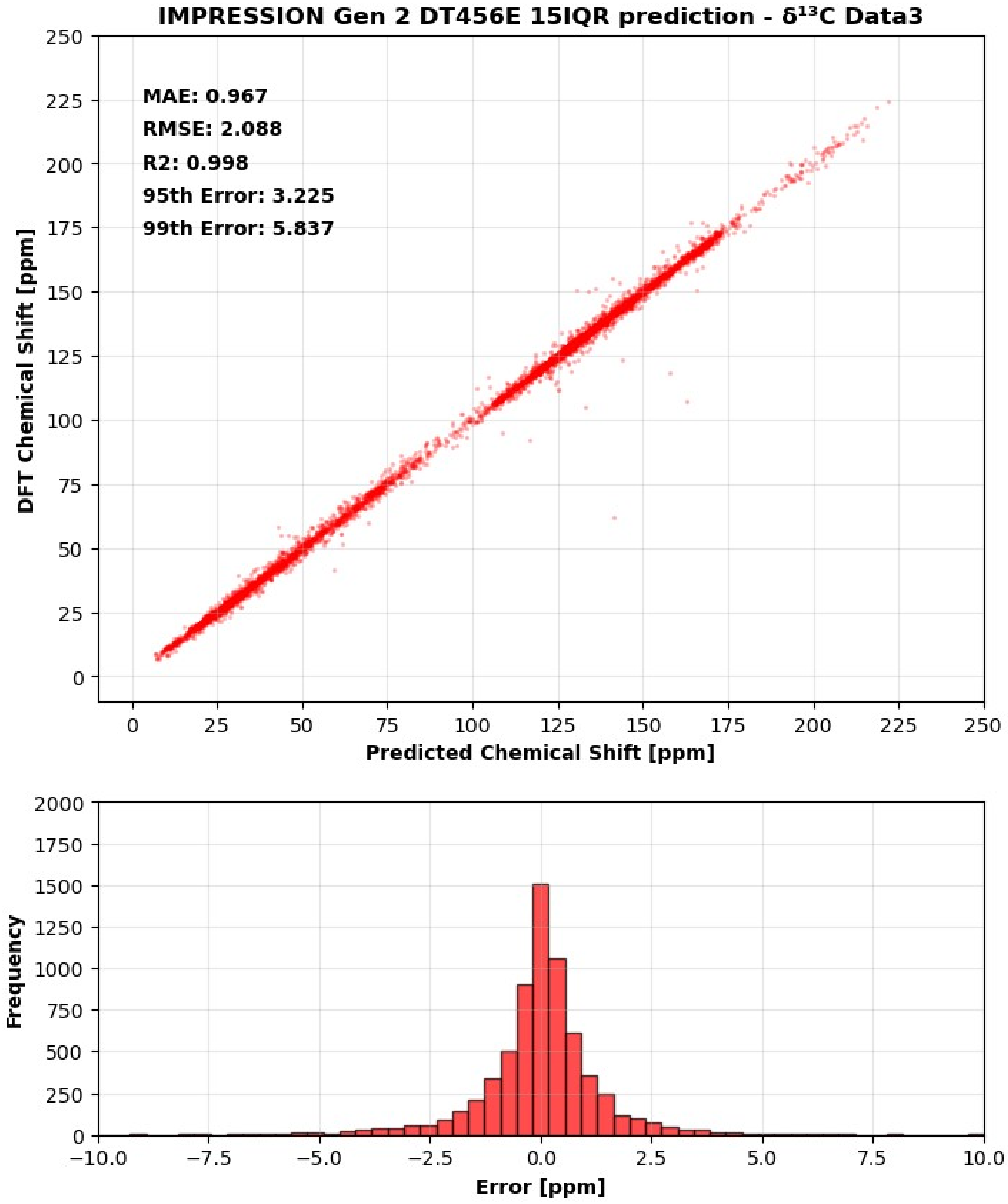
IMPRESSION Gen 2 DT456E 15IQR prediction - δ13C Data3
MAE: 0.967
RMSE: 2.088
R2: 0.998
95th Error: 3.225
99th Error: 5.837
DFT Chemical Shift [ppm]
Predicted Chemical Shift [ppm]
Frequency
Error [ppm]

DFT8K_bg

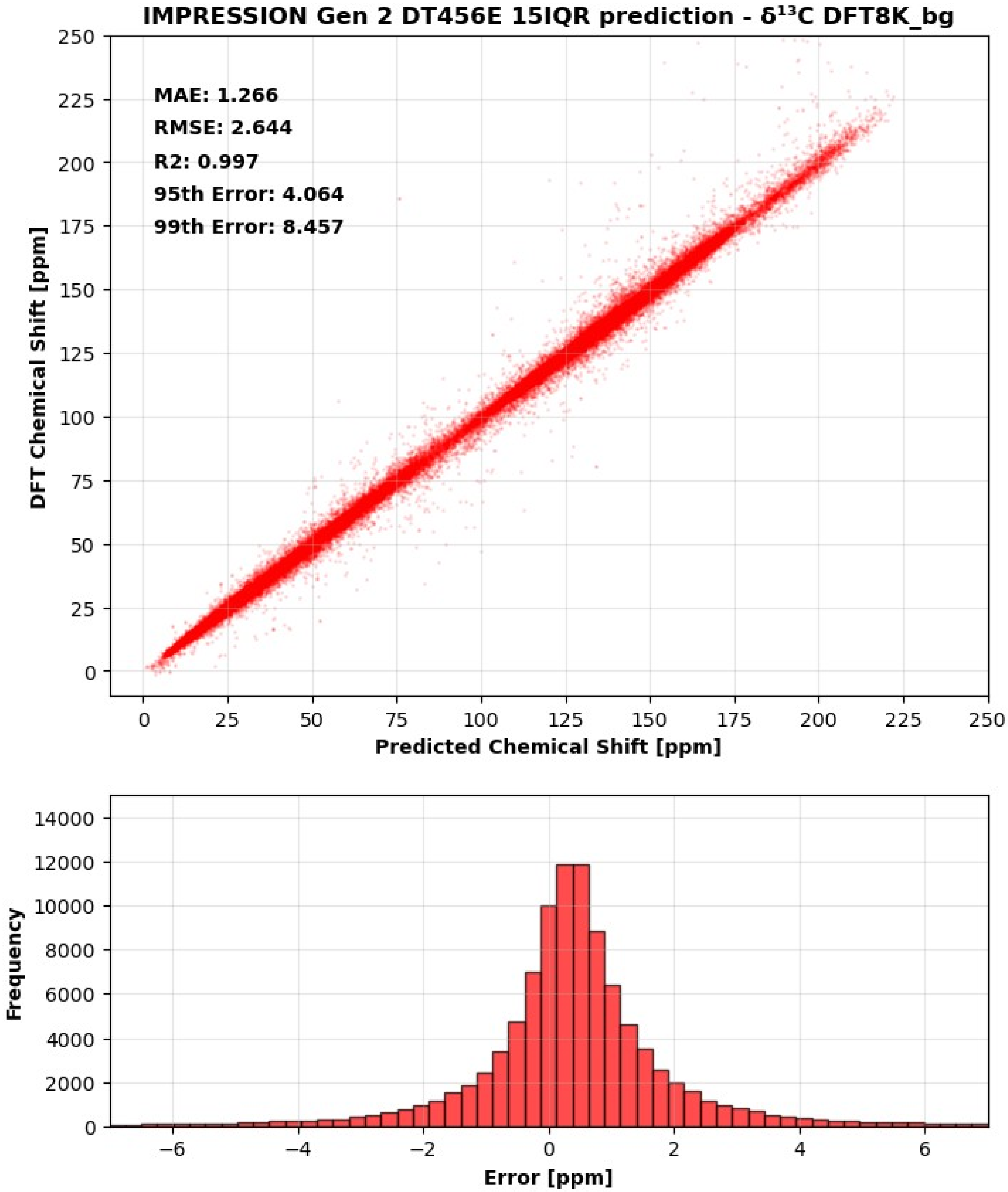

IMPRESSION Gen 2 DT456E 15IQR prediction - δ13C DFT8K_bg
MAE: 1.266
RMSE: 2.644
R2: 0.997
95th Error: 4.064
99th Error: 8.457
DFT Chemical Shift [ppm]
Predicted Chemical Shift [ppm]
Frequency
Error [ppm]

S2.1.3 $\delta^{15}N$

Holdout

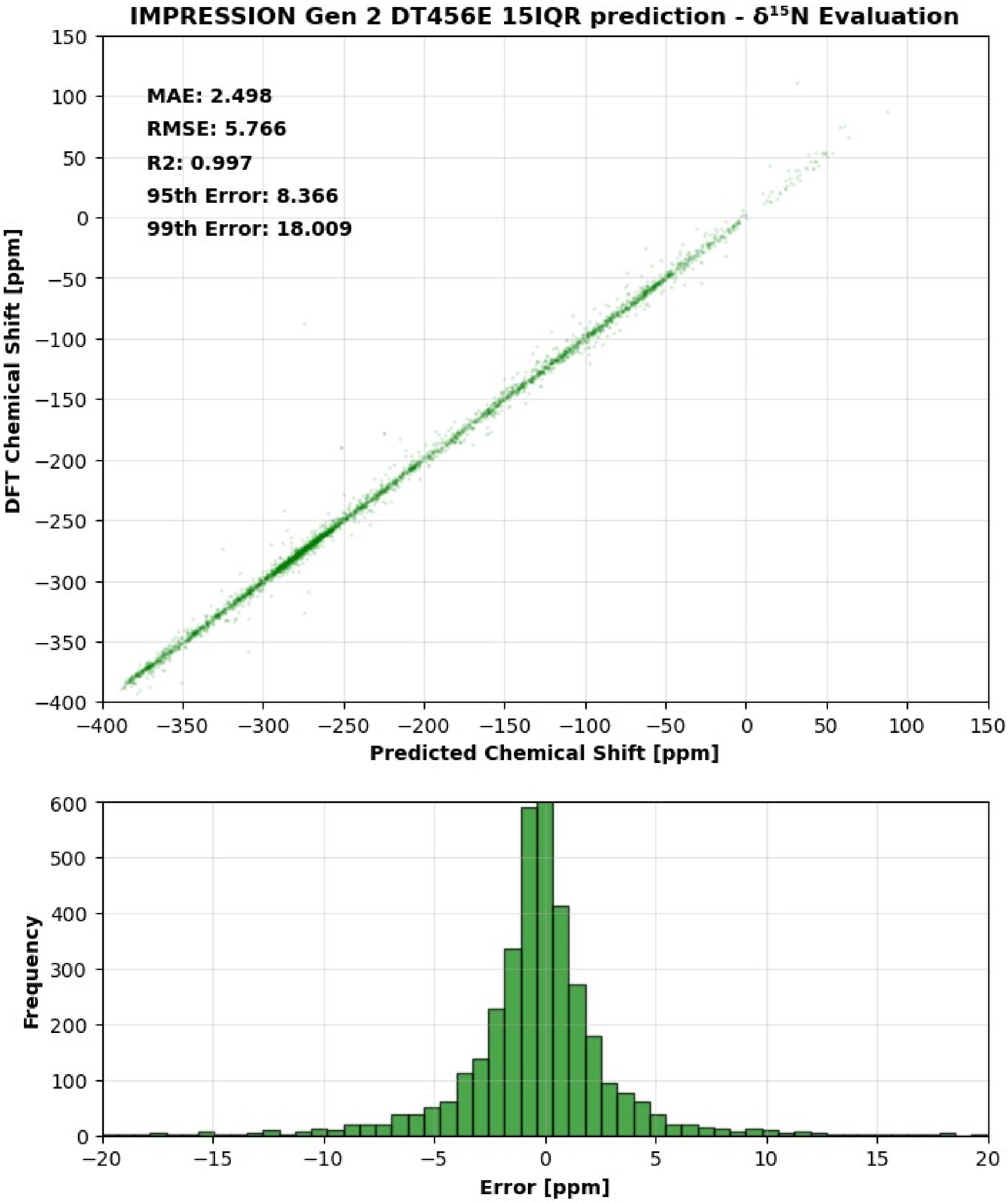

Data3

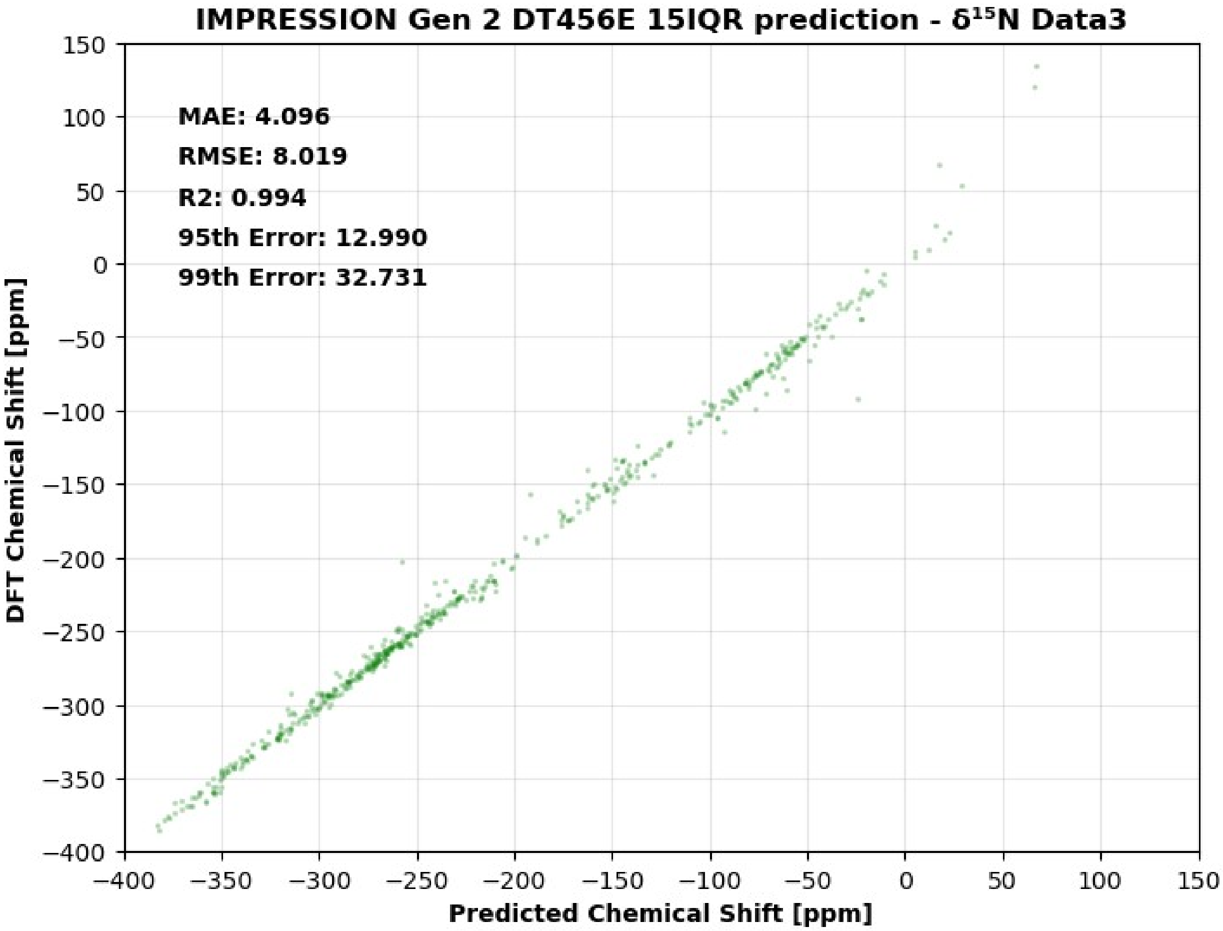

IMPRESSION Gen 2 DT456E 15IQR prediction - δ15N Data3
MAE: 4.096
RMSE: 8.019
R2: 0.994
95th Error: 12.990
99th Error: 32.731
DFT Chemical Shift [ppm]
Predicted Chemical Shift [ppm]

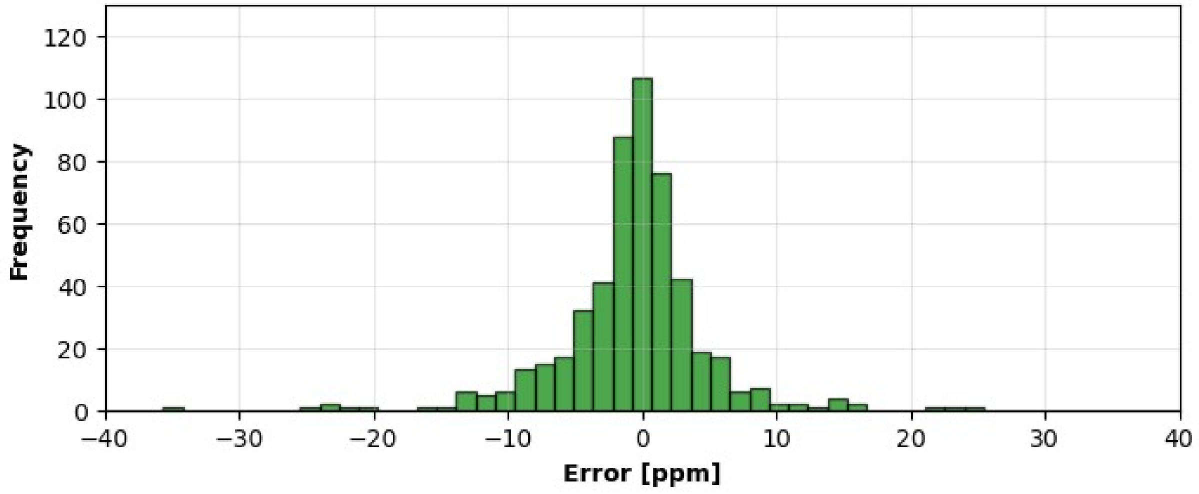

Frequency
Error [ppm]

DFT8K_bg

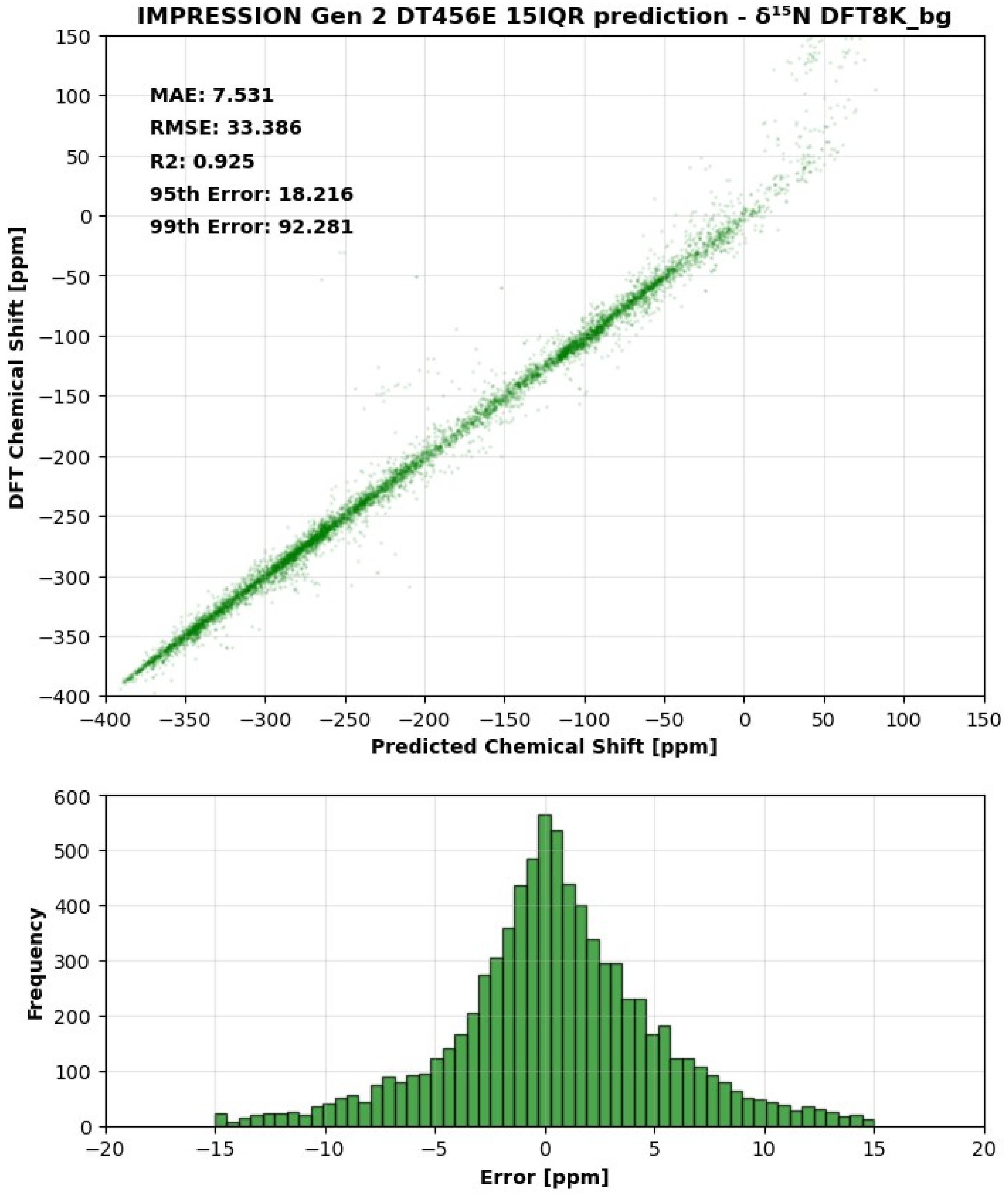
IMPRESSION Gen 2 DT456E 15IQR prediction - δ15N DFT8K_bg
MAE: 7.531
RMSE: 33.386
R2: 0.925
95th Error: 18.216
99th Error: 92.281
DFT Chemical Shift [ppm]
Predicted Chemical Shift [ppm]
Frequency
Error [ppm]

S2.1.4 δ^{19}F

Holdout

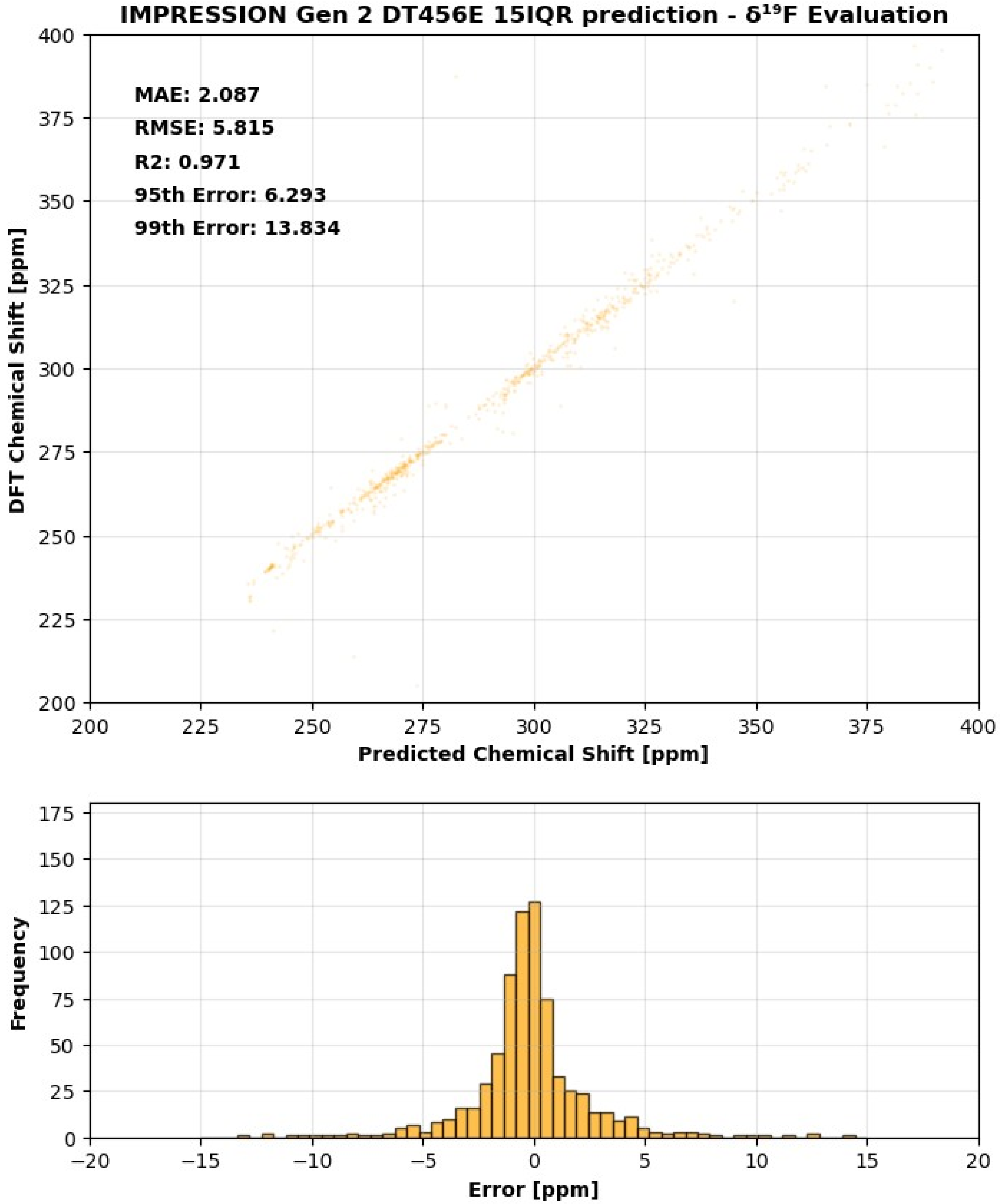

DFT8K_bg

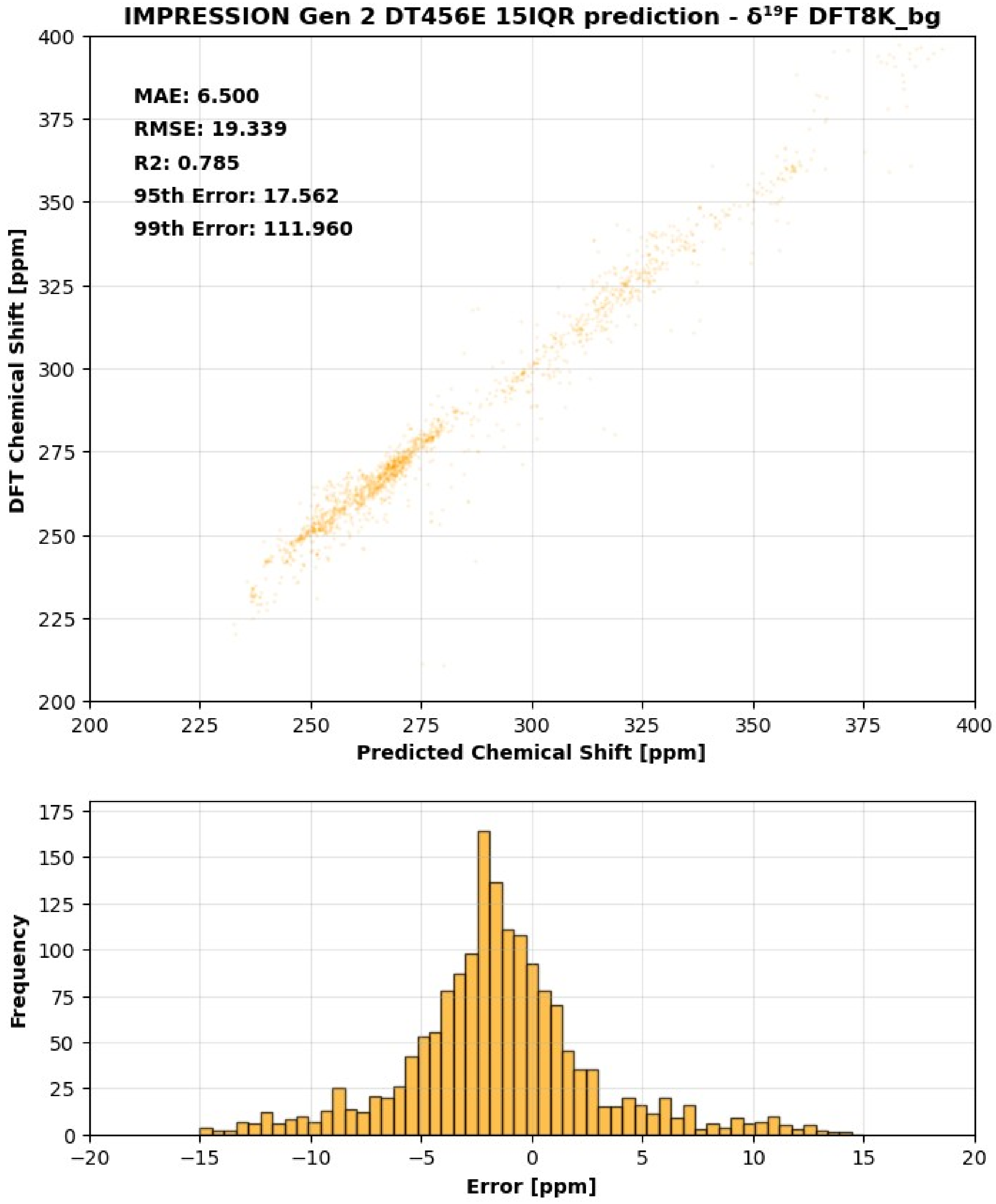
IMPRESSION Gen 2 DT456E 15IQR prediction - δ19F DFT8K_bg
MAE: 6.500
RMSE: 19.339
R2: 0.785
95th Error: 17.562
99th Error: 111.960
DFT Chemical Shift [ppm]
Predicted Chemical Shift [ppm]
Frequency
Error [ppm]

S2.2 Scalar couplings

S2.2.1 $^{1}J_{CH}$

Holdout

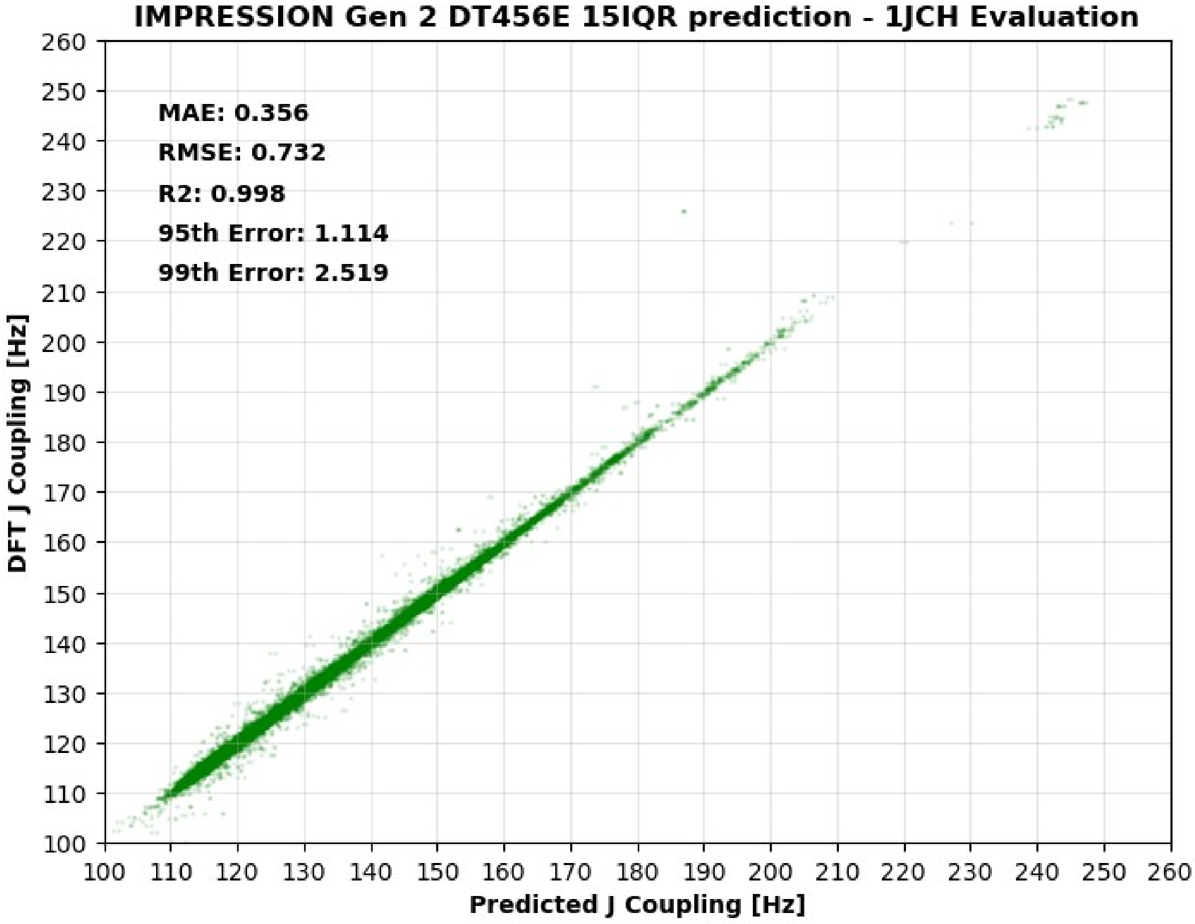

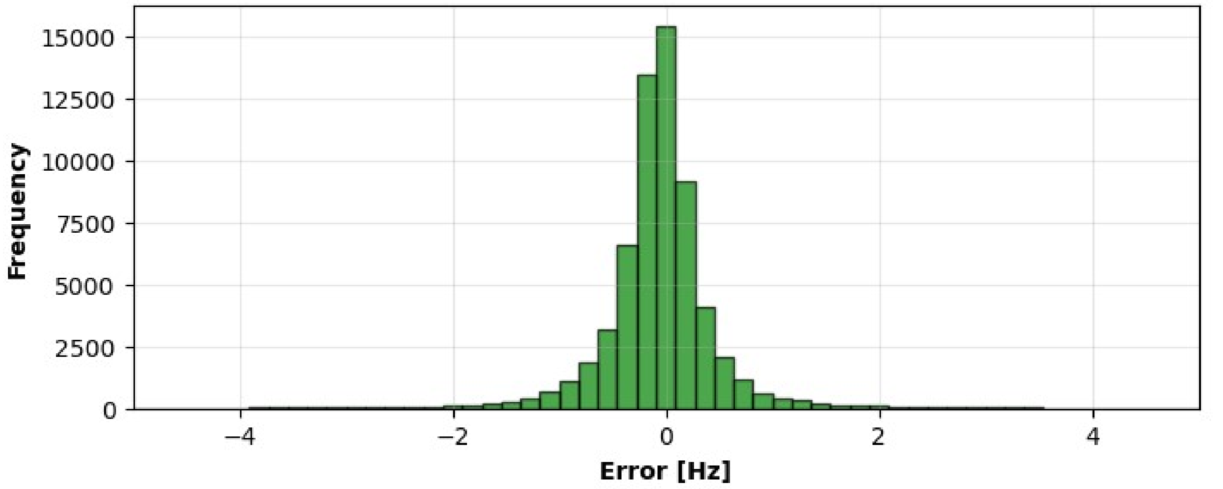

Data3

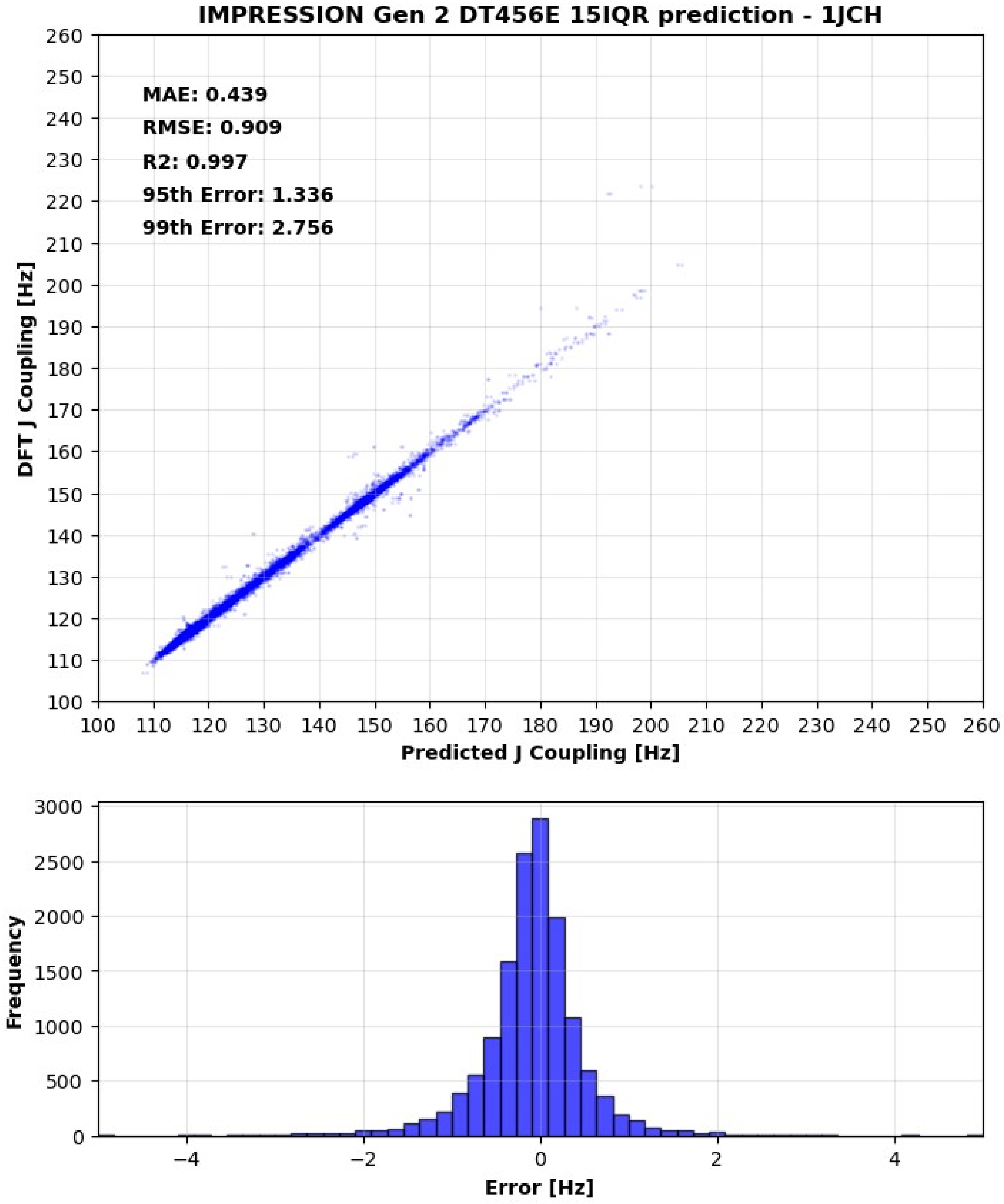
IMPRESSION Gen 2 DT456E 15IQR prediction - 1JCH
MAE: 0.439
RMSE: 0.909
R2: 0.997
95th Error: 1.336
99th Error: 2.756
DFT J Coupling [Hz]
Predicted J Coupling [Hz]
Frequency
Error [Hz]

DFT8K_bg

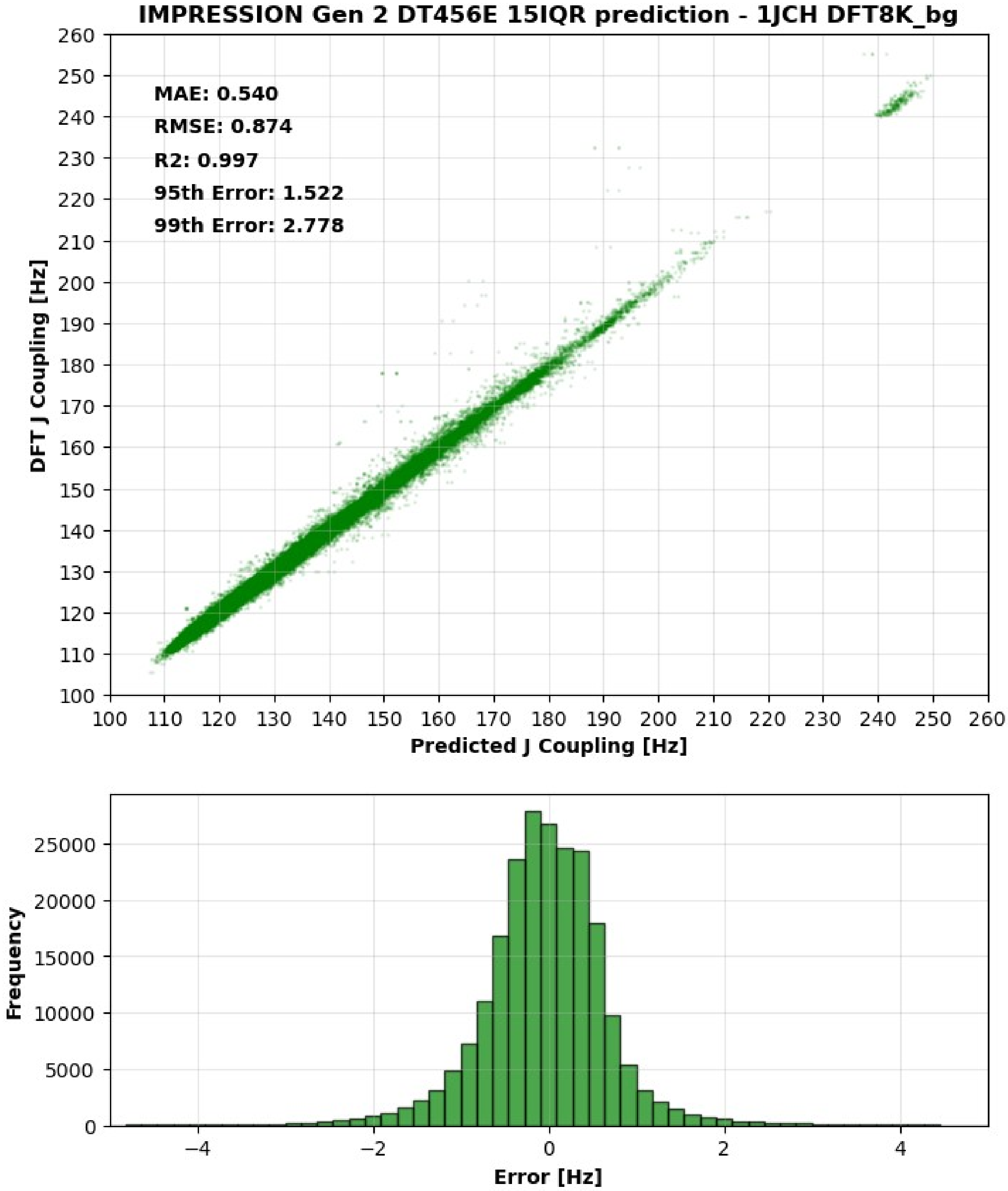

IMPRESSION Gen 2 DT456E 15IQR prediction - 1JCH DFT8K_bg
MAE: 0.540
RMSE: 0.874
R2: 0.997
95th Error: 1.522
99th Error: 2.778
DFT J Coupling [Hz]
Predicted J Coupling [Hz]
Frequency
Error [Hz]

S2.2.2 $^2J_{CH}$

Holdout

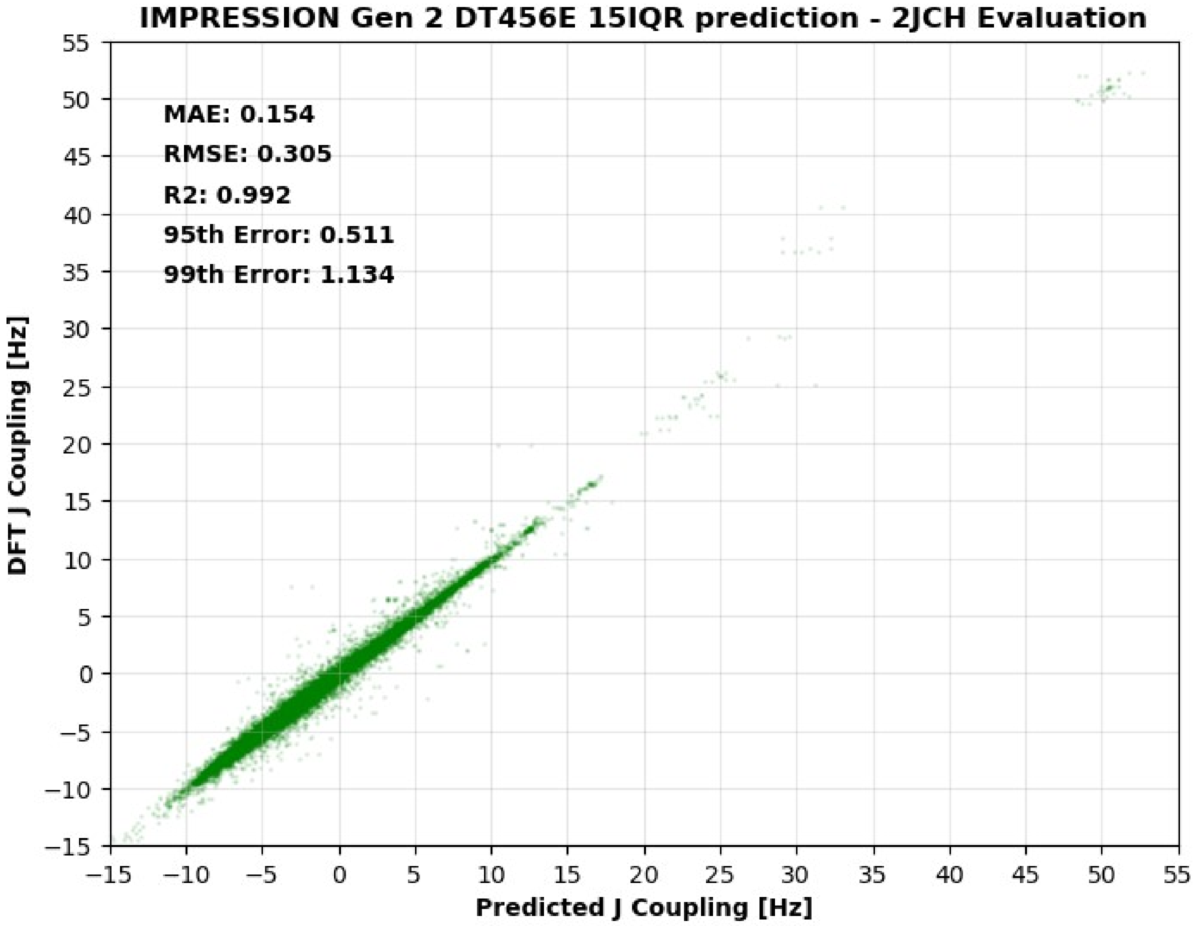

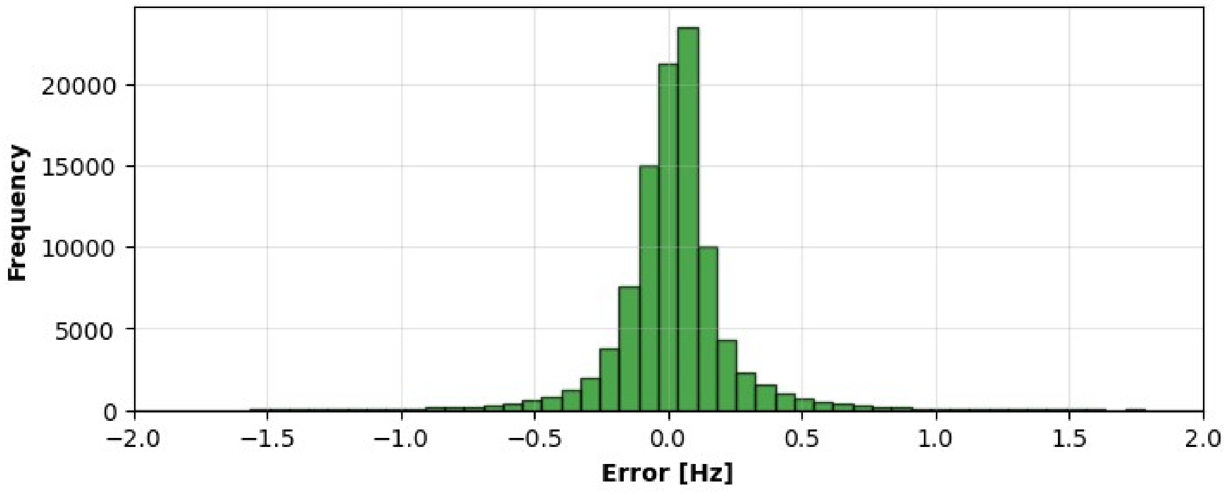

Data3

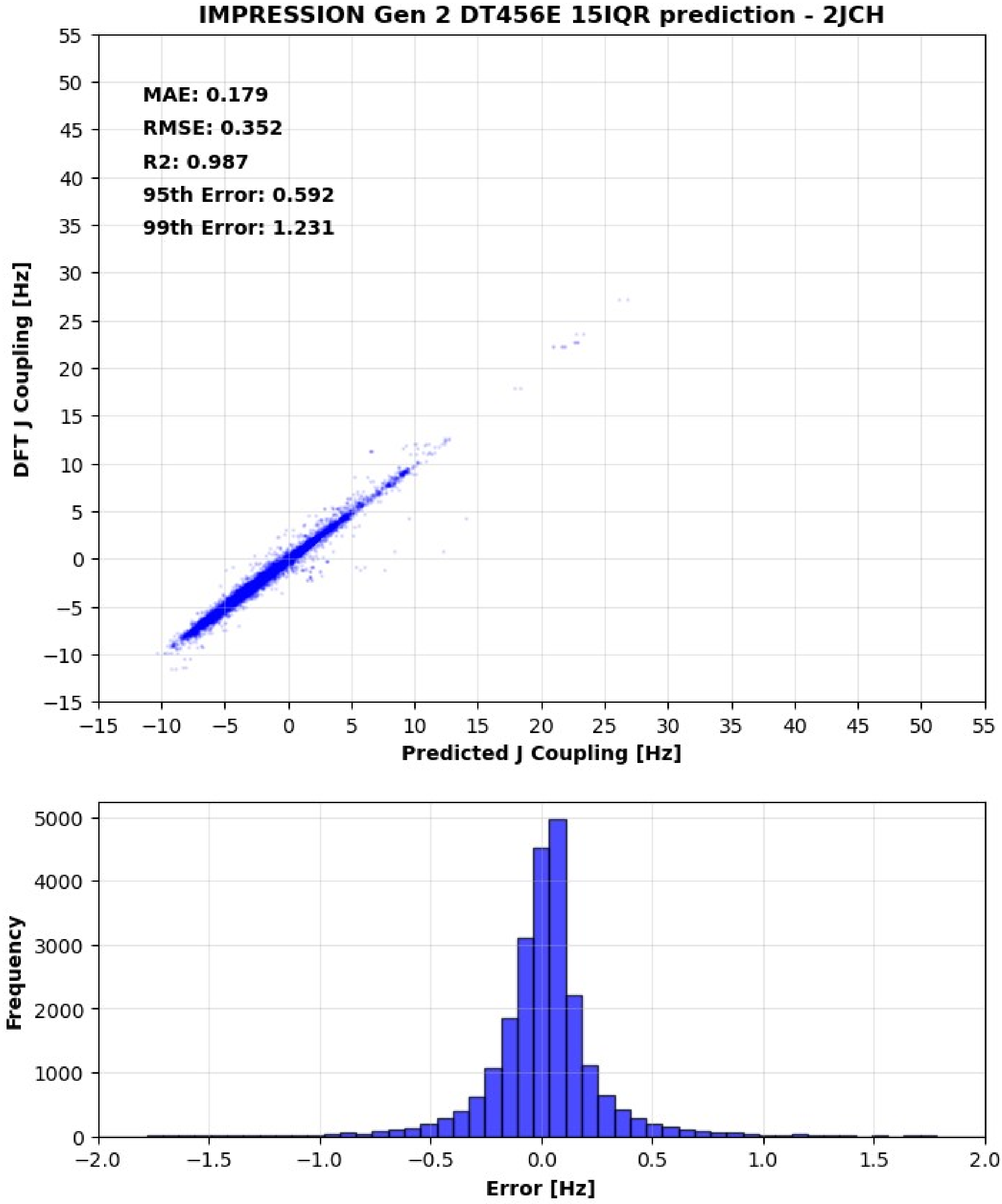
IMPRESSION Gen 2 DT456E 15IQR prediction - 2JCH
MAE: 0.179
RMSE: 0.352
R2: 0.987
95th Error: 0.592
99th Error: 1.231
DFT J Coupling [Hz]
Predicted J Coupling [Hz]
55
50
45
40
35
30
25
20
15
10
5
0
−5
−10
−15
Frequency
Error [Hz]
5000
4000
3000
2000
1000
−2.0
−1.5
−1.0
−0.5
0.0
0.5
1.0
1.5
2.0

DFT8K_bg

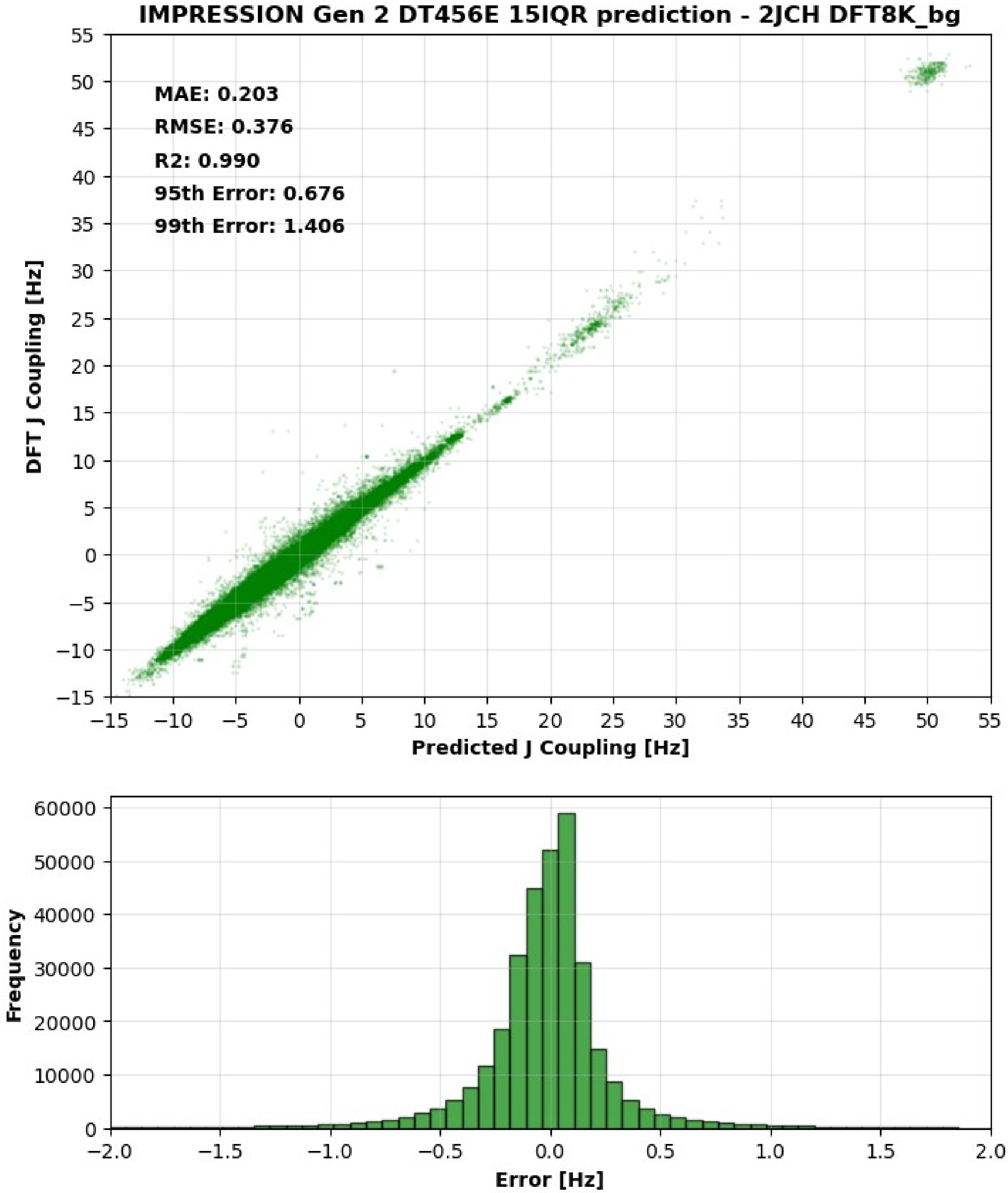
IMPRESSION Gen 2 DT456E 15IQR prediction - 2JCH DFT8K_bg
MAE: 0.203
RMSE: 0.376
R2: 0.990
95th Error: 0.676
99th Error: 1.406
DFT J Coupling [Hz]
Predicted J Coupling [Hz]
Frequency
Error [Hz]

S2.2.3 $^3J_{CH}$

Holdout

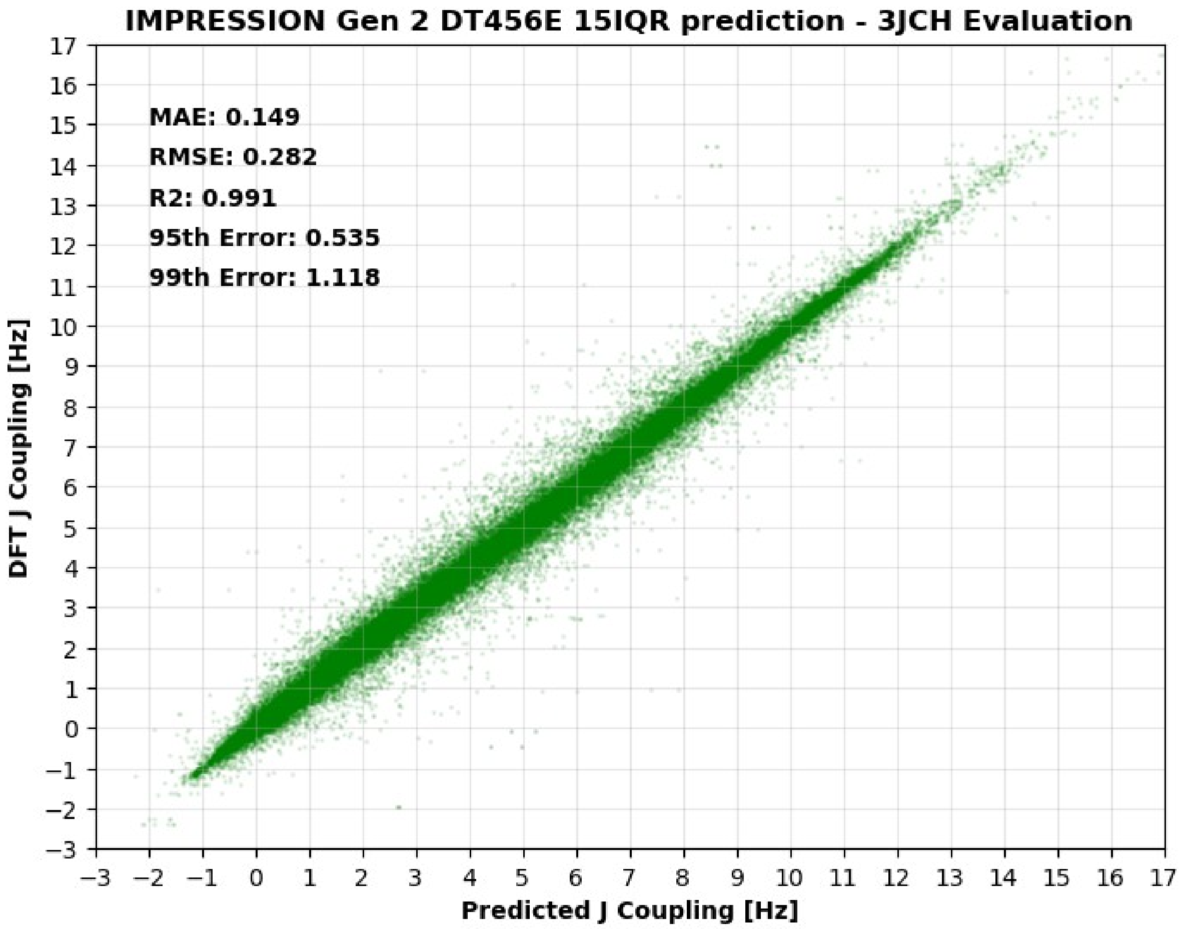

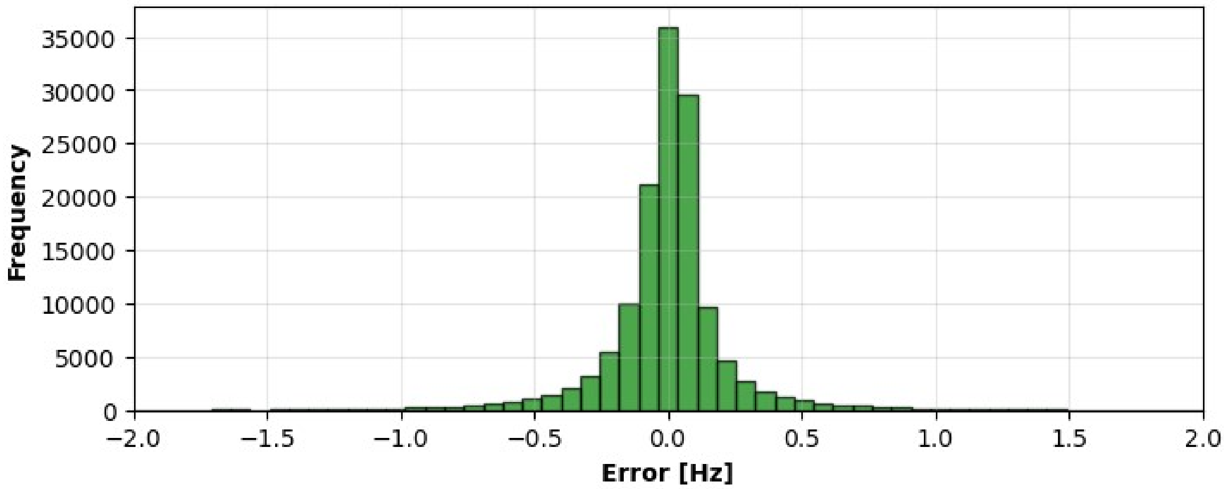

Data3

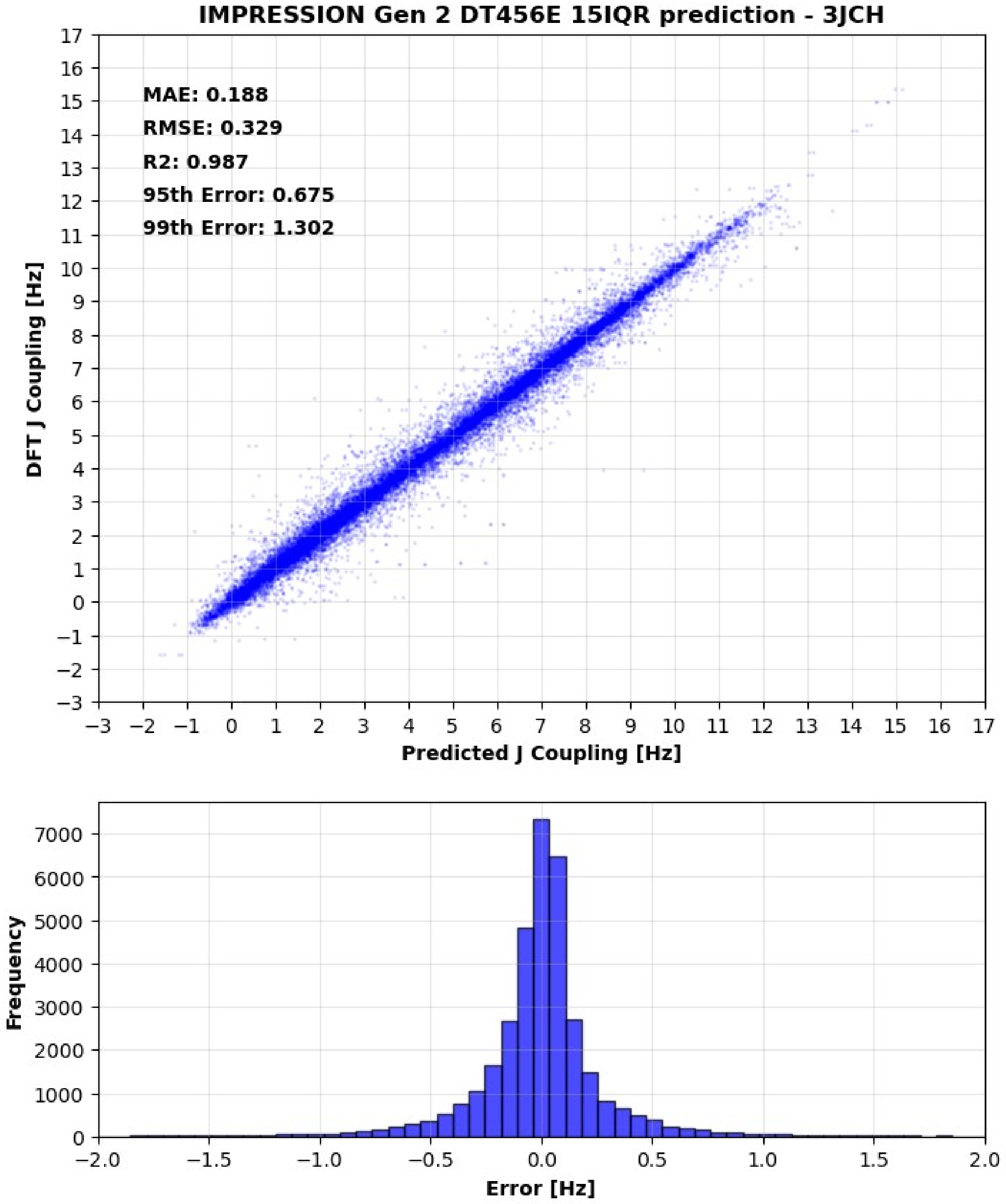
IMPRESSION Gen 2 DT456E 15IQR prediction - 3JCH
MAE: 0.188
RMSE: 0.329
R2: 0.987
95th Error: 0.675
99th Error: 1.302
DFT J Coupling [Hz]
Predicted J Coupling [Hz]
−3
−2
−1
0
1
2
3
4
5
6
7
8
9
10
11
12
13
14
15
16
17
Frequency
Error [Hz]
0
1000
2000
3000
4000
5000
6000
7000
−2.0
−1.5
−1.0
−0.5
0.0
0.5
1.0
1.5
2.0

DFT8K_bg

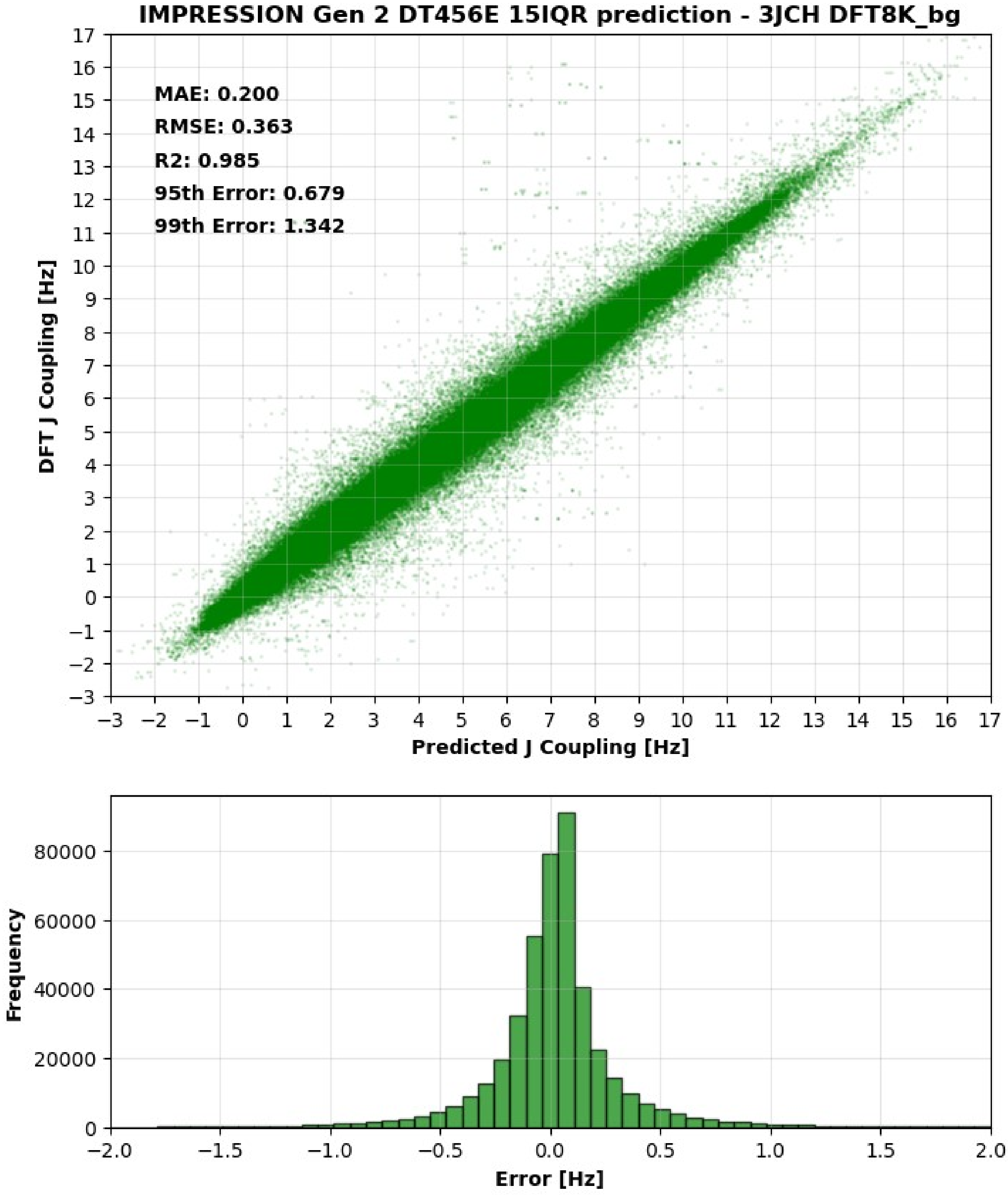

IMPRESSION Gen 2 DT456E 15IQR prediction - 3JCH DFT8K_bg
MAE: 0.200
RMSE: 0.363
R2: 0.985
95th Error: 0.679
99th Error: 1.342
DFT J Coupling [Hz]
Predicted J Coupling [Hz]
Frequency
Error [Hz]

S2.2.4 $^4J_{CH}$

Holdout

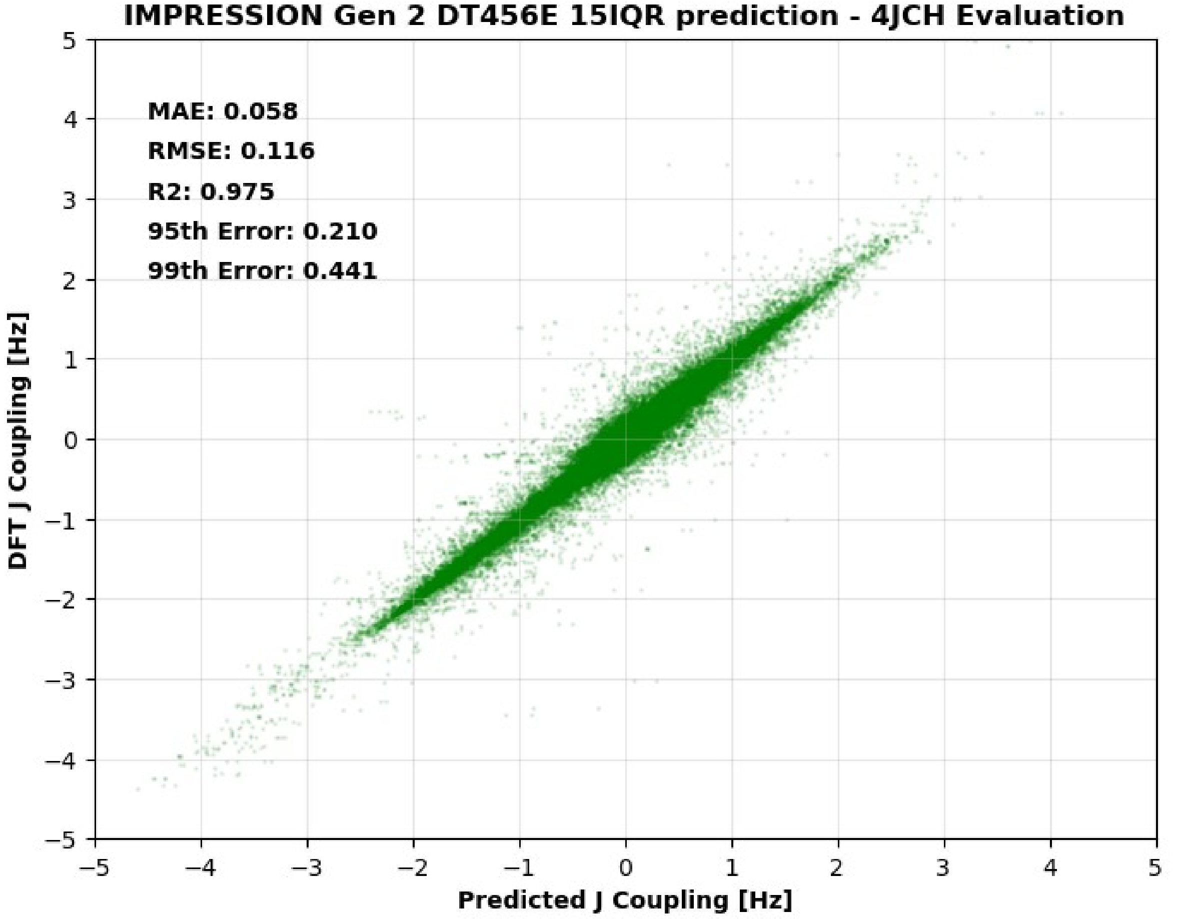

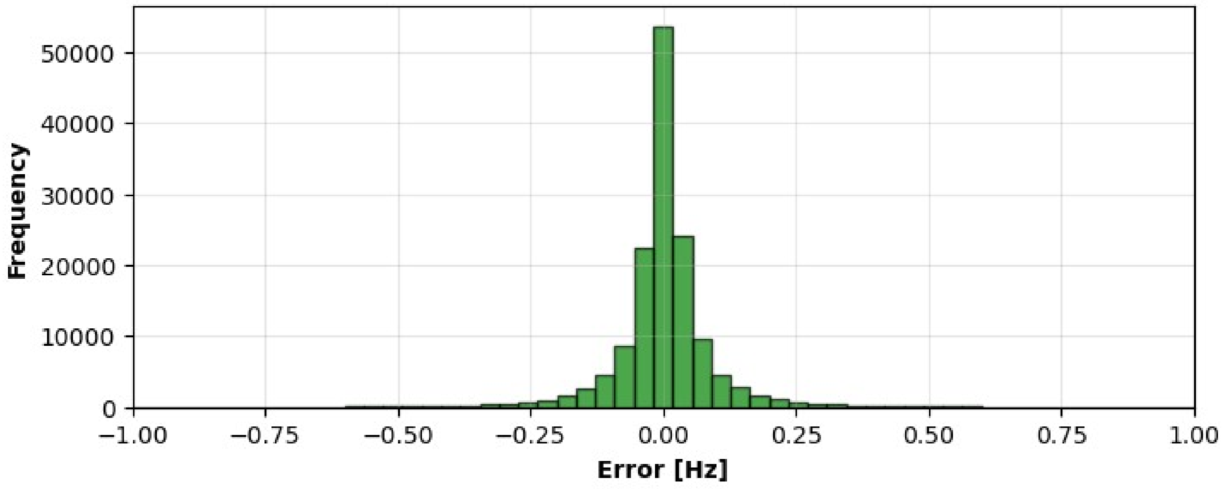

Data3

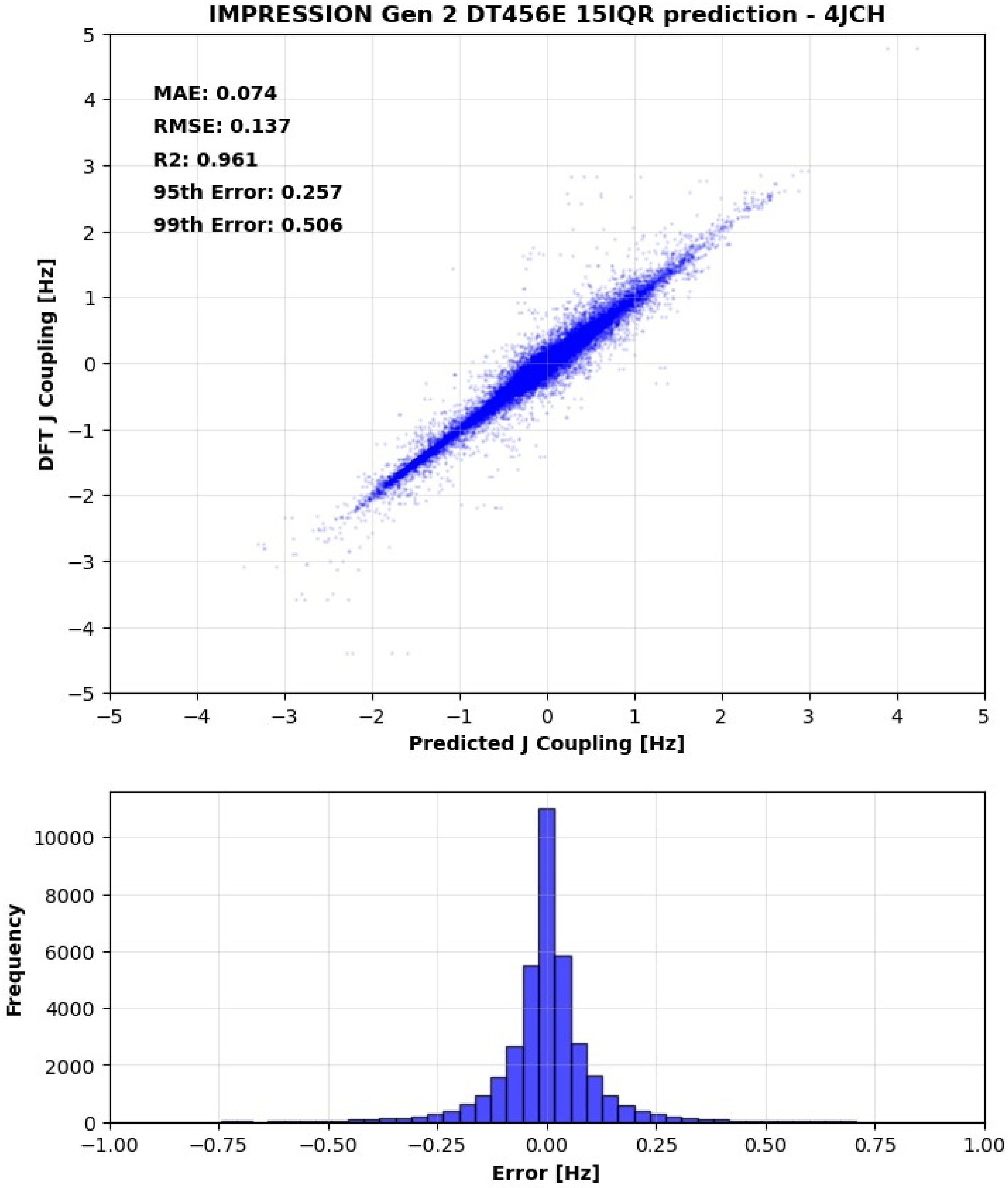
IMPRESSION Gen 2 DT456E 15IQR prediction - 4JCH
MAE: 0.074
RMSE: 0.137
R2: 0.961
95th Error: 0.257
99th Error: 0.506
DFT J Coupling [Hz]
Predicted J Coupling [Hz]
Frequency
Error [Hz]

DFT8K_bg

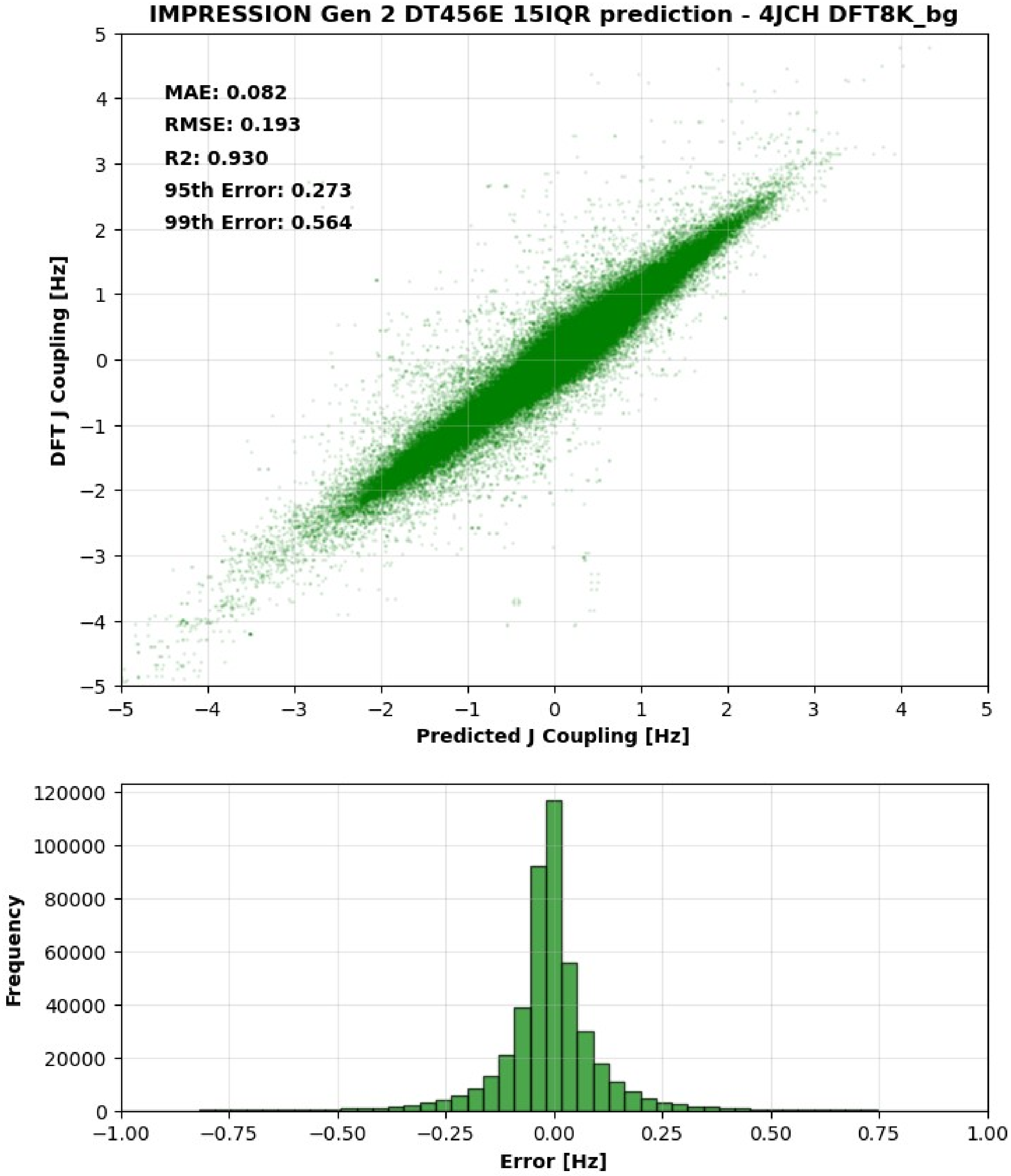

IMPRESSION Gen 2 DT456E 15IQR prediction - 4JCH DFT8K_bg
MAE: 0.082
RMSE: 0.193
R2: 0.930
95th Error: 0.273
99th Error: 0.564
DFT J Coupling [Hz]
Predicted J Coupling [Hz]
Frequency
Error [Hz]

S2.2.5 $^2J_{HH}$

Holdout

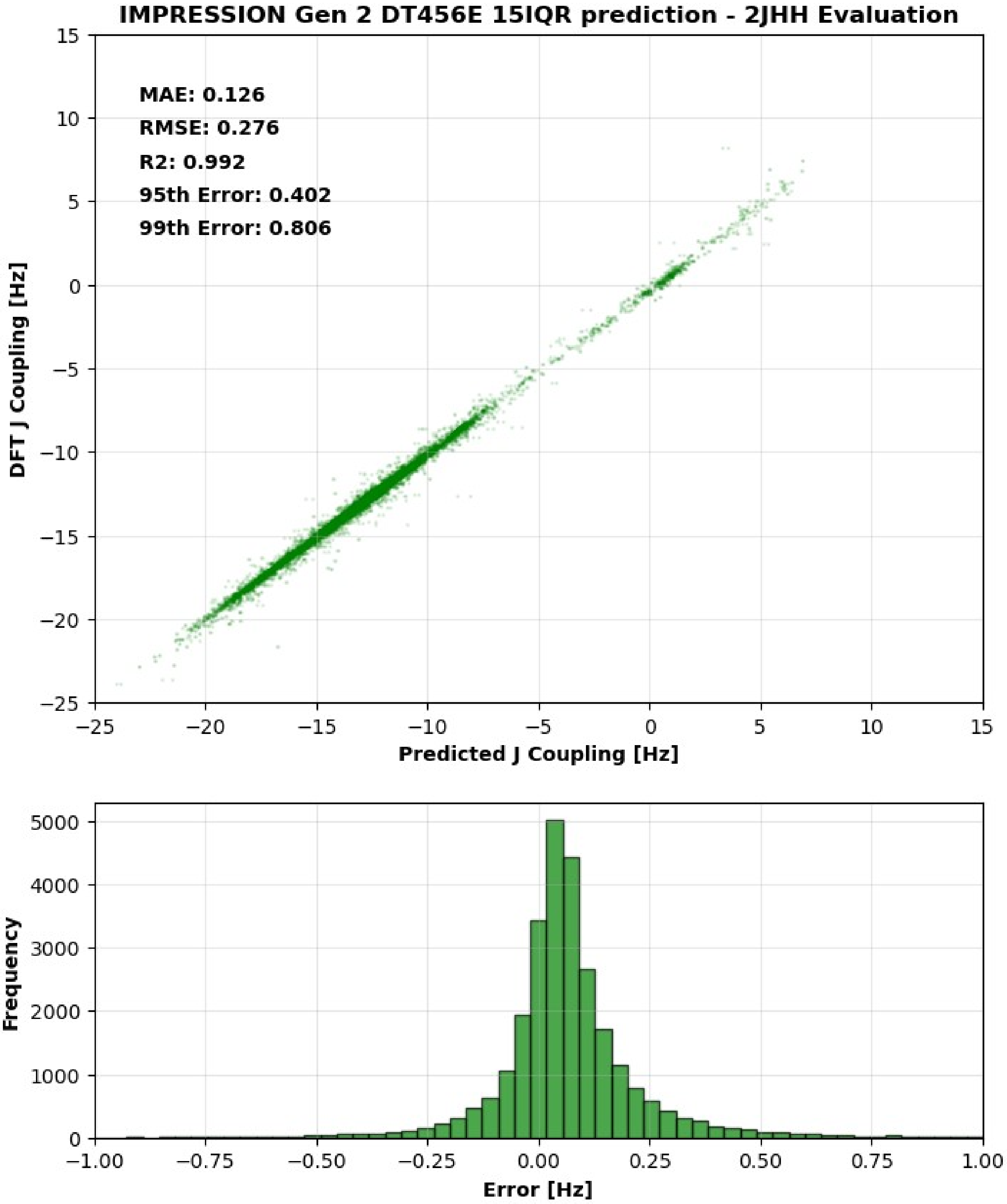

Data3

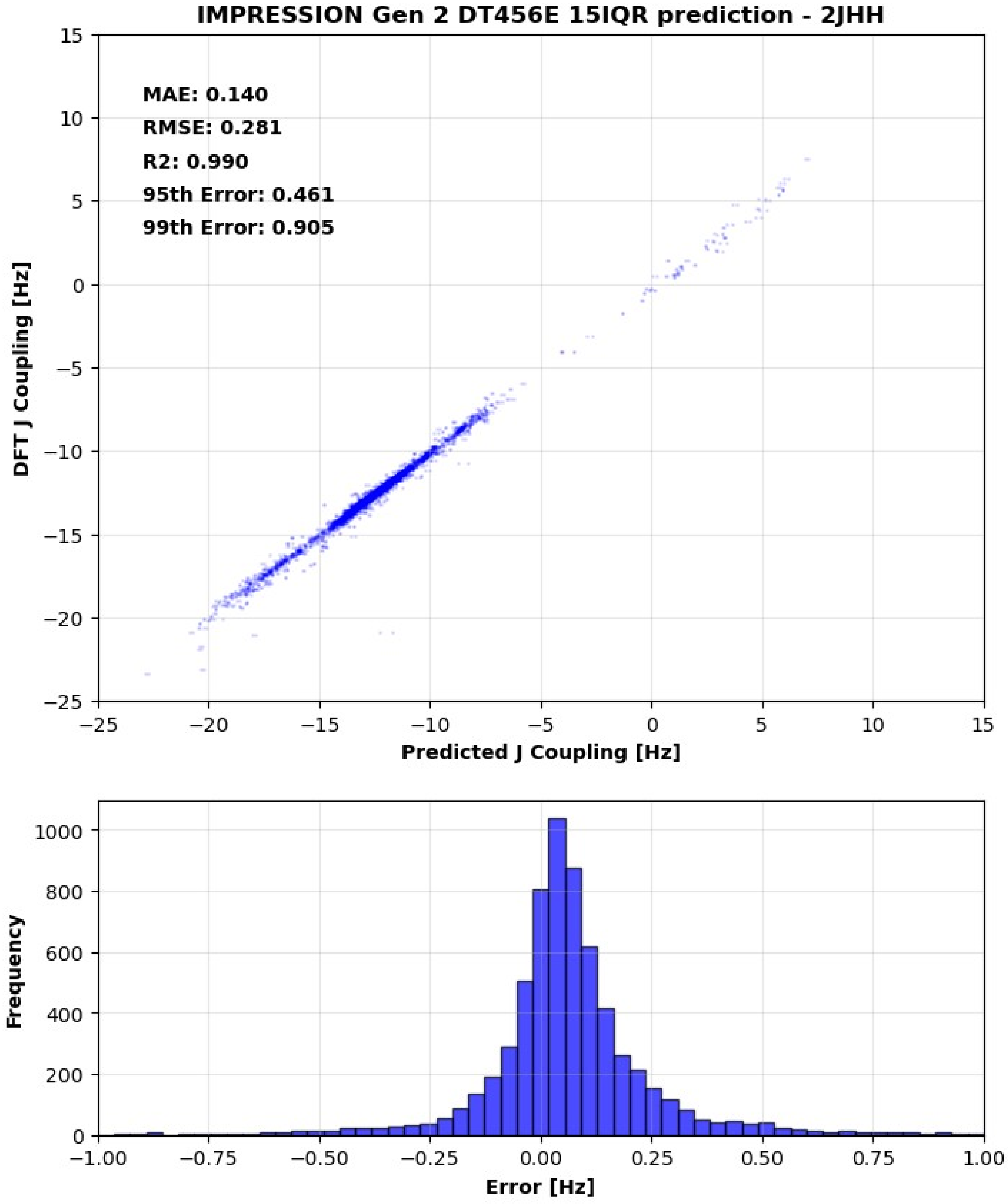
IMPRESSION Gen 2 DT456E 15IQR prediction - 2JHH
MAE: 0.140
RMSE: 0.281
R2: 0.990
95th Error: 0.461
99th Error: 0.905
DFT J Coupling [Hz]
Predicted J Coupling [Hz]
Frequency
Error [Hz]

DFT8K_bg

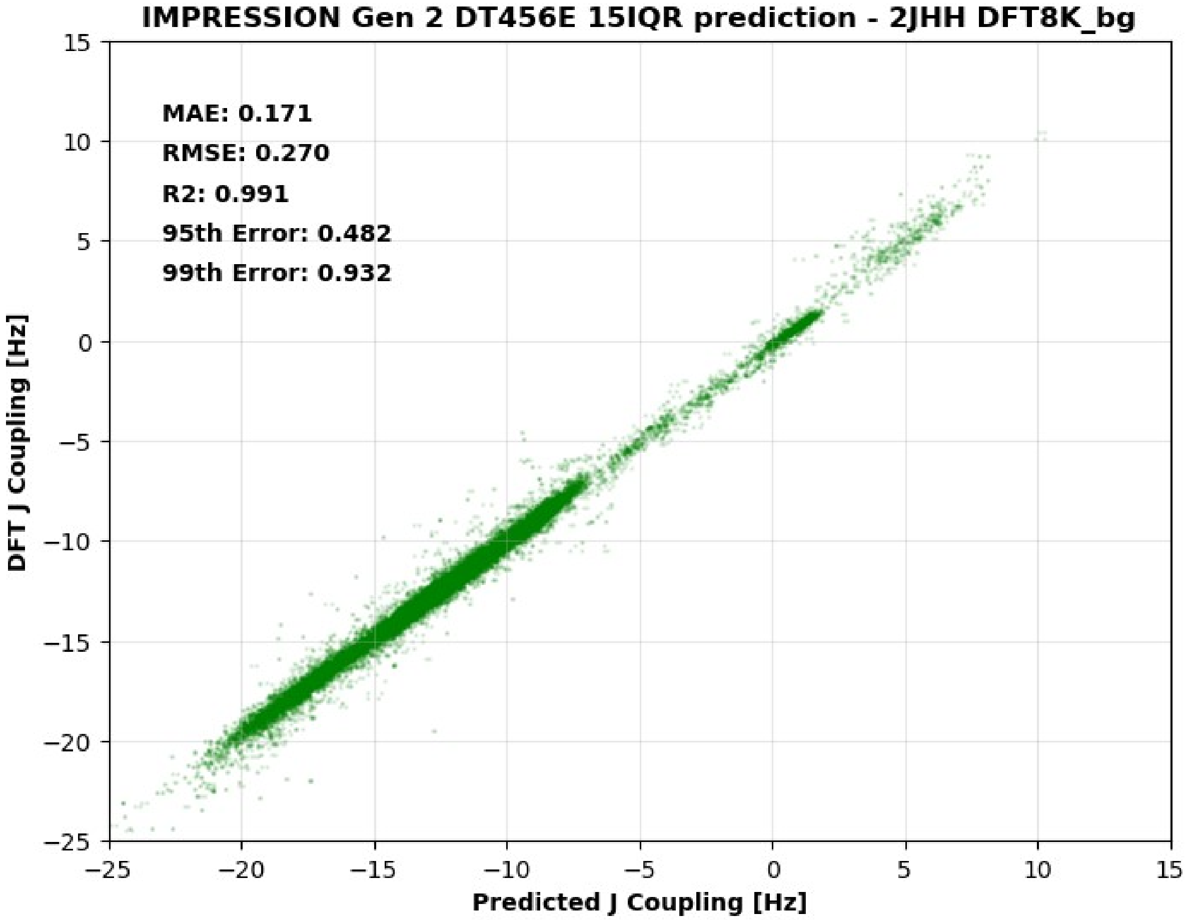
IMPRESSION Gen 2 DT456E 15IQR prediction - 2JHH DFT8K_bg
MAE: 0.171
RMSE: 0.270
R2: 0.991
95th Error: 0.482
99th Error: 0.932
DFT J Coupling [Hz]
Predicted J Coupling [Hz]
15
10
5
0
−5
−10
−15
−20
−25
−25
−20
−15
−10
−5
0
5
10
15

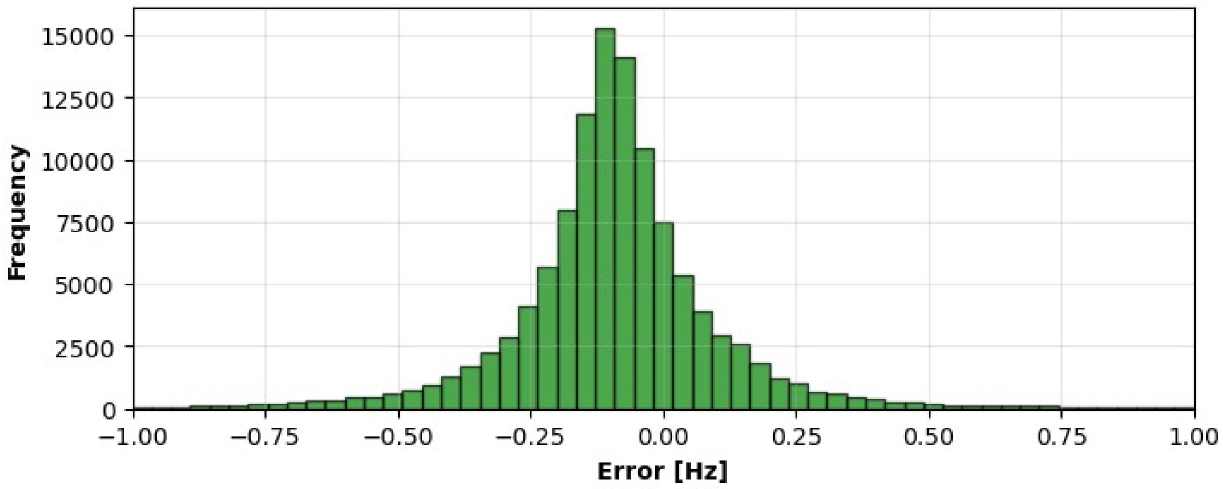
Frequency
Error [Hz]
15000
12500
10000
7500
5000
2500
0
−1.00
−0.75
−0.50
−0.25
0.00
0.25
0.50
0.75
1.00

S2.2.6 $^3J_{HH}$

Holdout

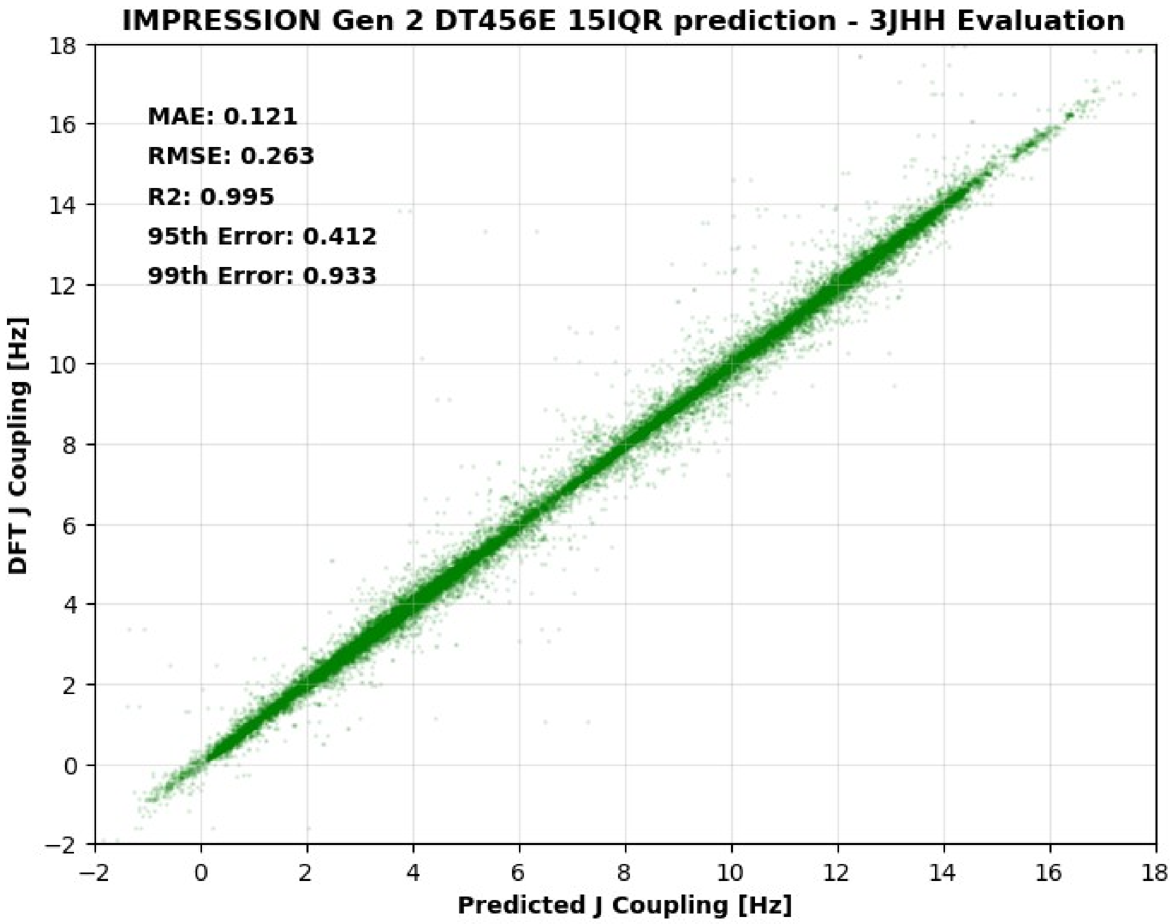

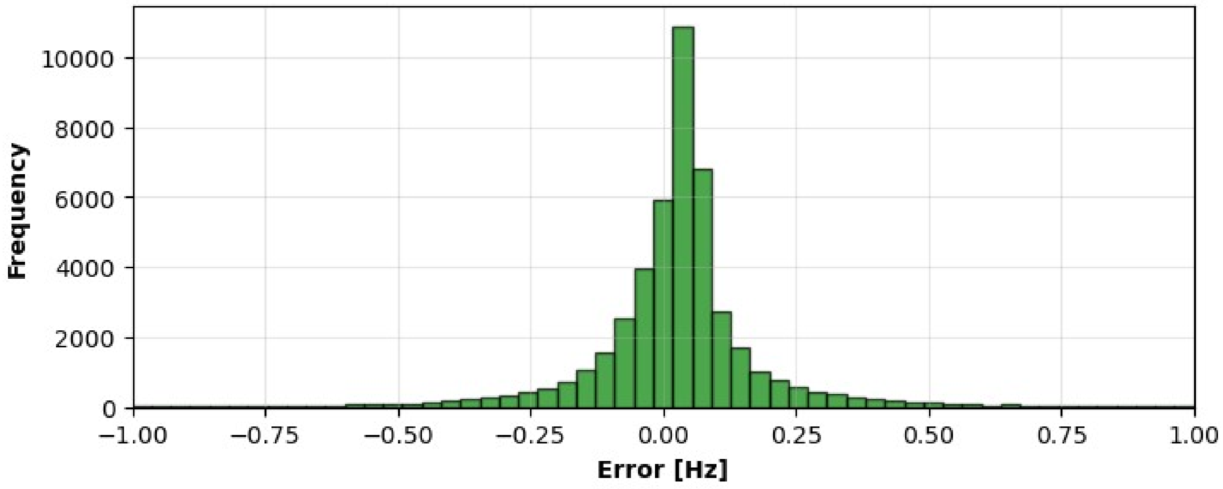

Data3

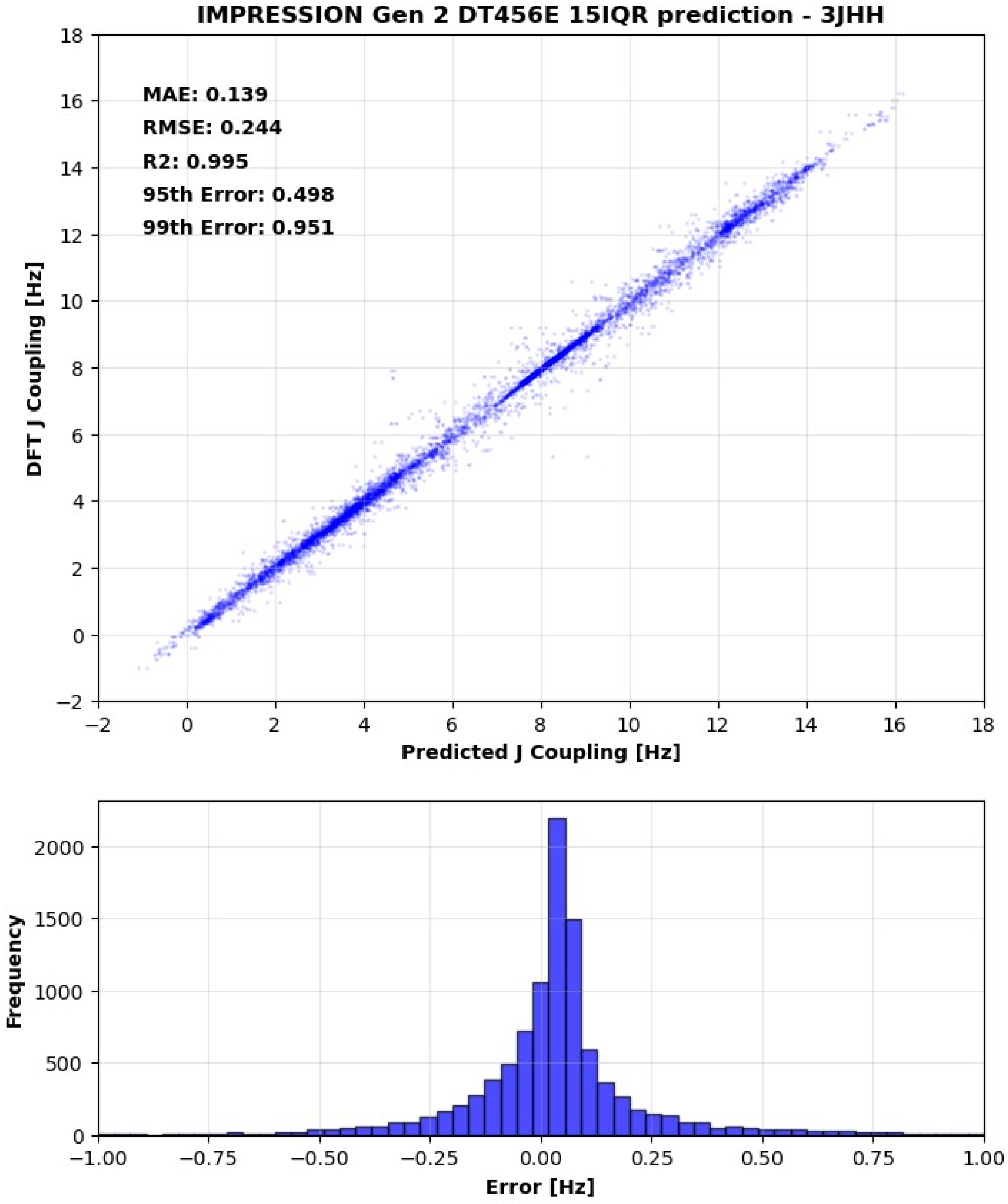

IMPRESSION Gen 2 DT456E 15IQR prediction - 3JHH
MAE: 0.139
RMSE: 0.244
R2: 0.995
95th Error: 0.498
99th Error: 0.951
DFT J Coupling [Hz]
Predicted J Coupling [Hz]
Frequency
Error [Hz]

DFT8K_bg

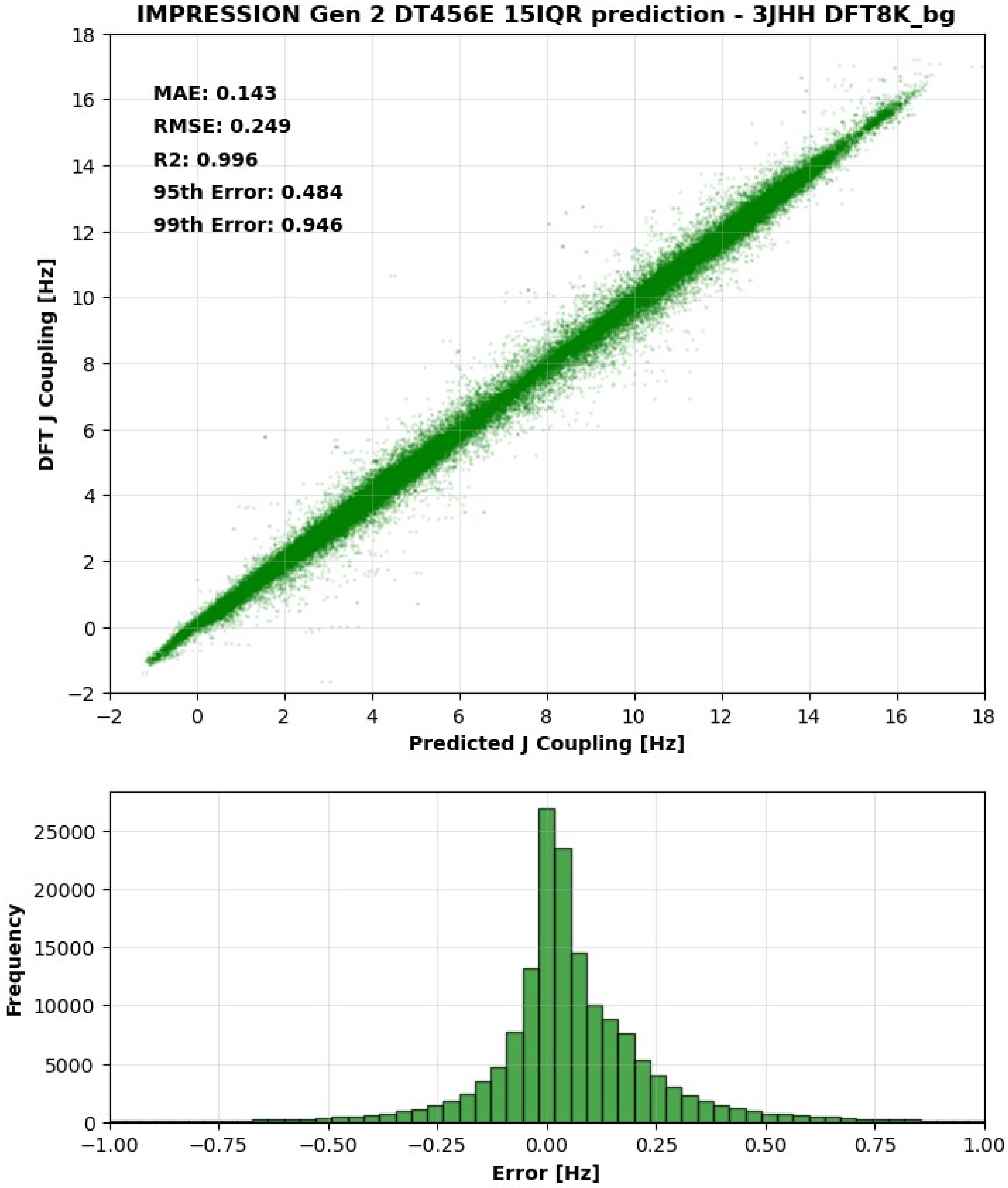
IMPRESSION Gen 2 DT456E 15IQR prediction - 3JHH DFT8K_bg
MAE: 0.143
RMSE: 0.249
R2: 0.996
95th Error: 0.484
99th Error: 0.946
DFT J Coupling [Hz]
Predicted J Coupling [Hz]
Frequency
Error [Hz]

S2.2.7 $^4J_{HH}$

Holdout

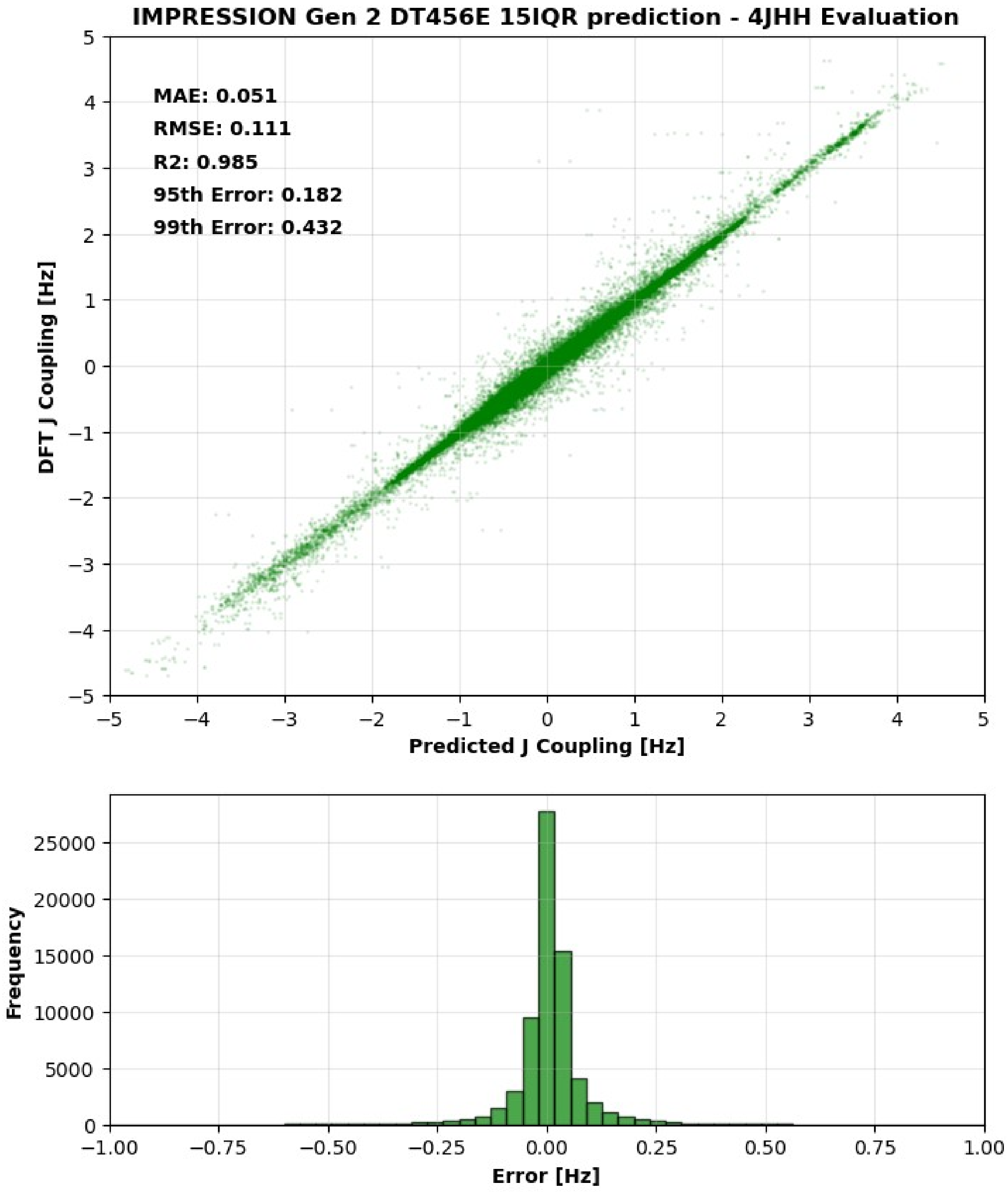

Data3

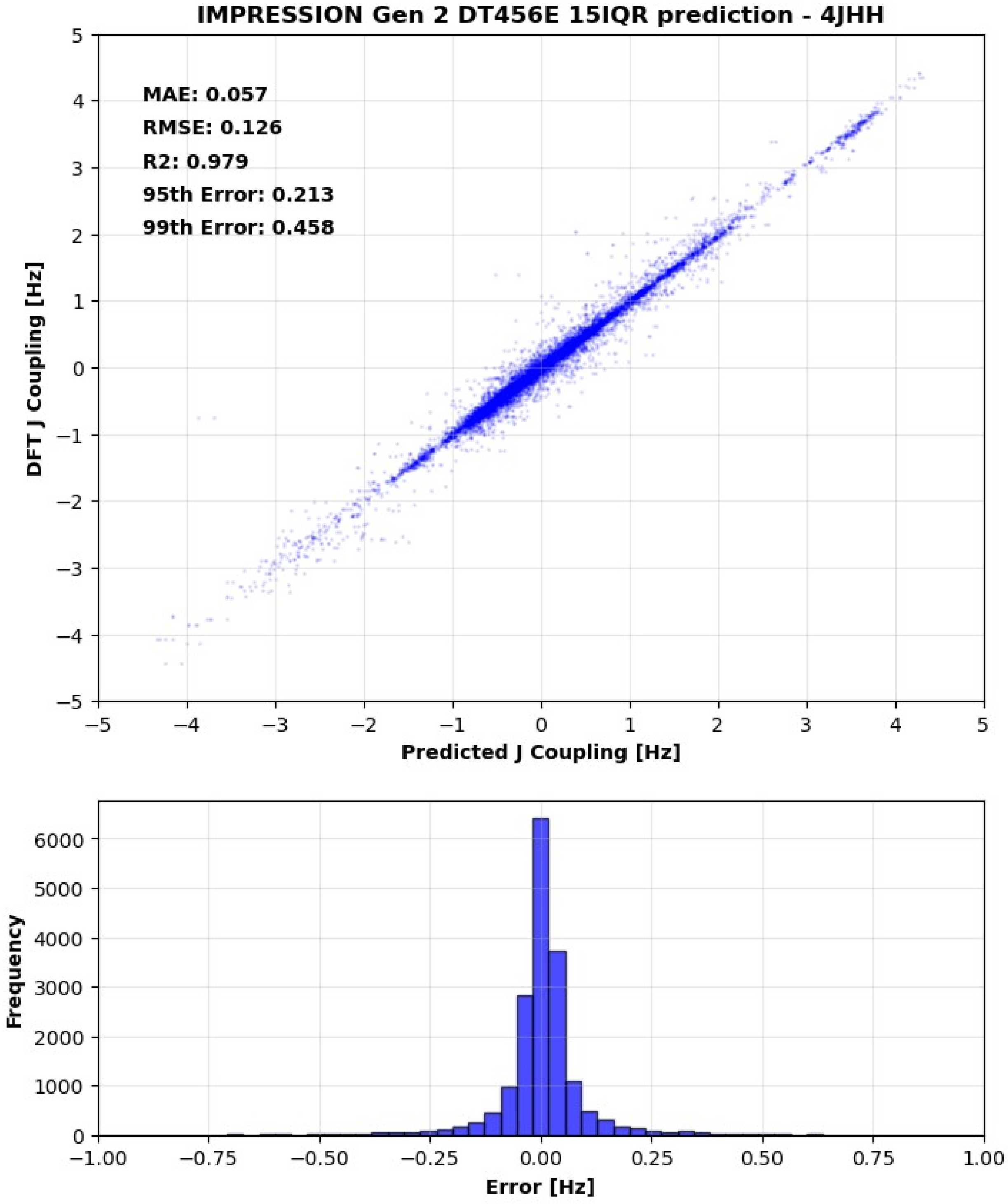

IMPRESSION Gen 2 DT456E 15IQR prediction - 4JHH
MAE: 0.057
RMSE: 0.126
R2: 0.979
95th Error: 0.213
99th Error: 0.458
DFT J Coupling [Hz]
Predicted J Coupling [Hz]
Frequency
Error [Hz]

DFT8K_bg

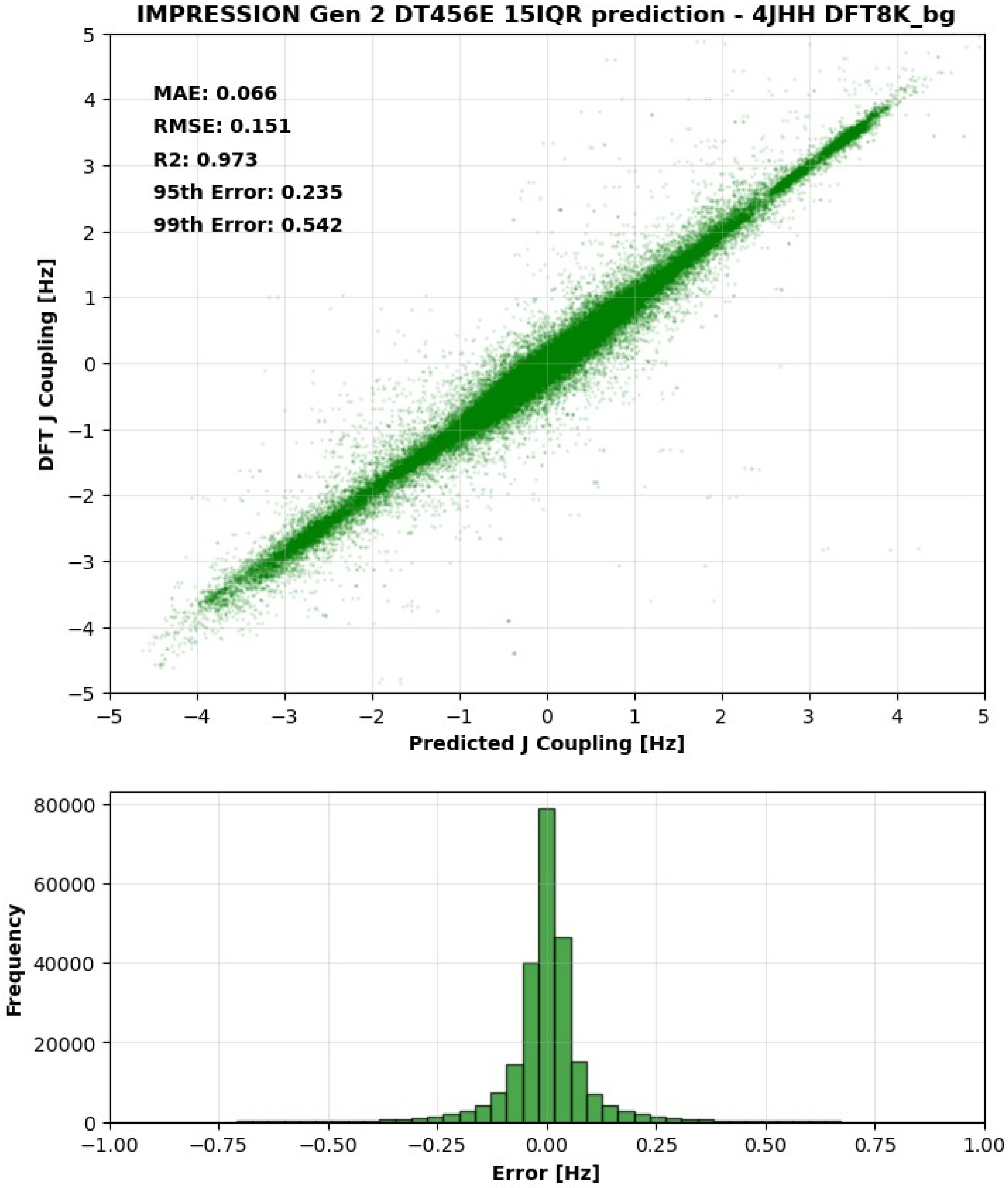

IMPRESSION Gen 2 DT456E 15IQR prediction - 4JHH DFT8K_bg
MAE: 0.066
RMSE: 0.151
R2: 0.973
95th Error: 0.235
99th Error: 0.542
DFT J Coupling [Hz]
Predicted J Coupling [Hz]
Frequency
Error [Hz]

S2.2.8 $^1J_{CC}$

Holdout

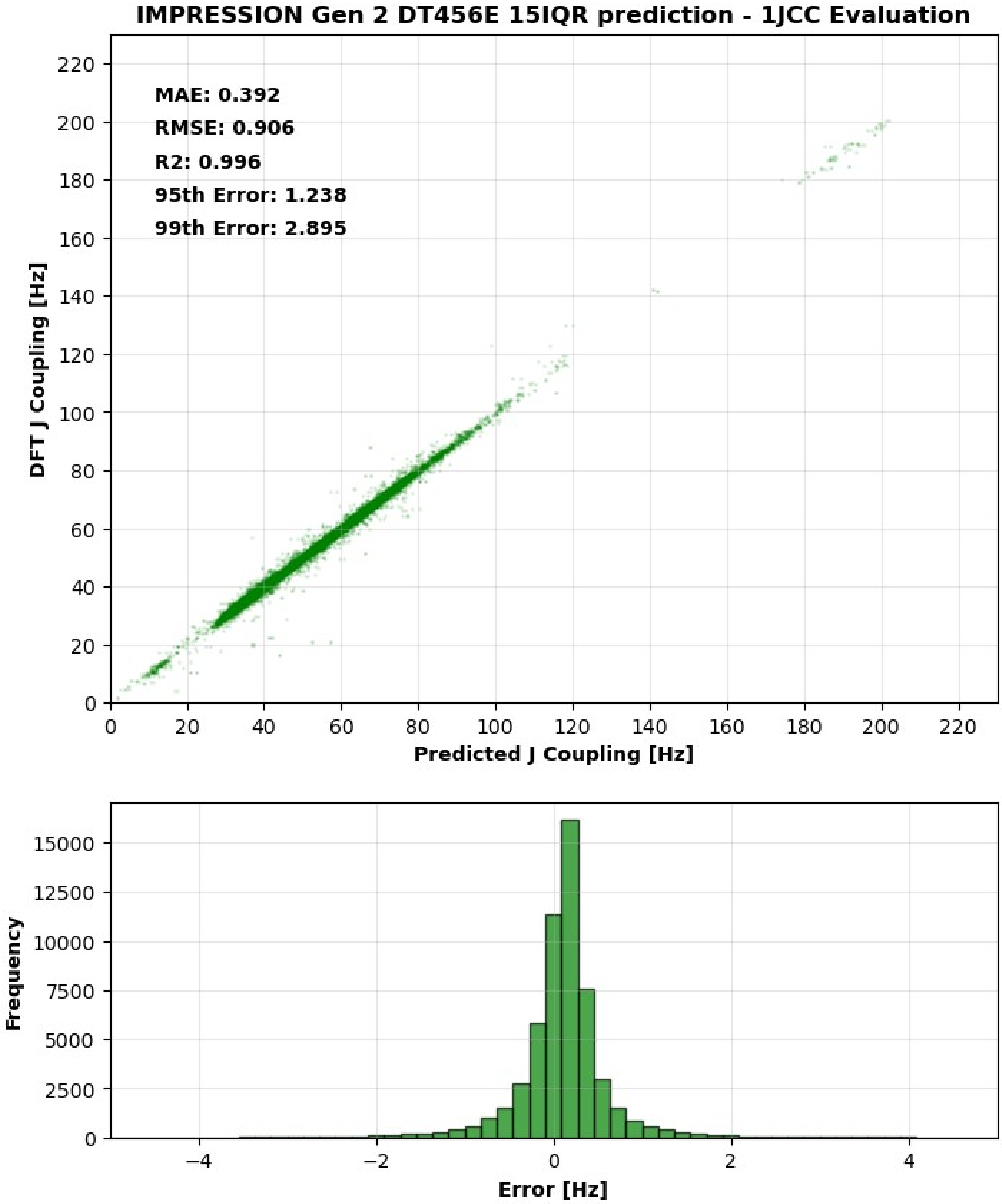

Data3

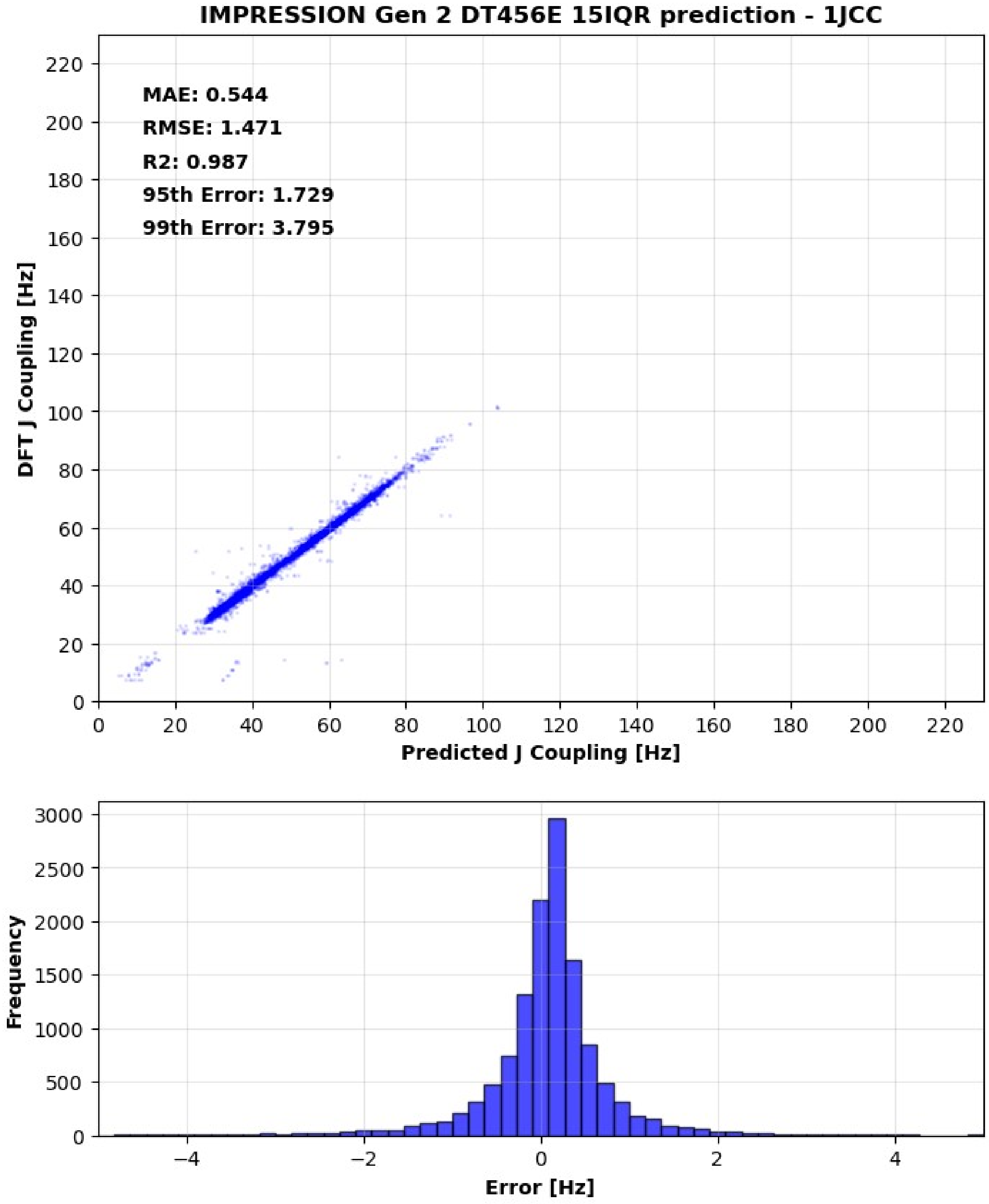
IMPRESSION Gen 2 DT456E 15IQR prediction - 1JCC
MAE: 0.544
RMSE: 1.471
R2: 0.987
95th Error: 1.729
99th Error: 3.795
DFT J Coupling [Hz]
Predicted J Coupling [Hz]
0
20
40
60
80
100
120
140
160
180
200
220
Frequency
Error [Hz]
0
500
1000
1500
2000
2500
3000
−4
−2
0
2
4

DFT8K_bg

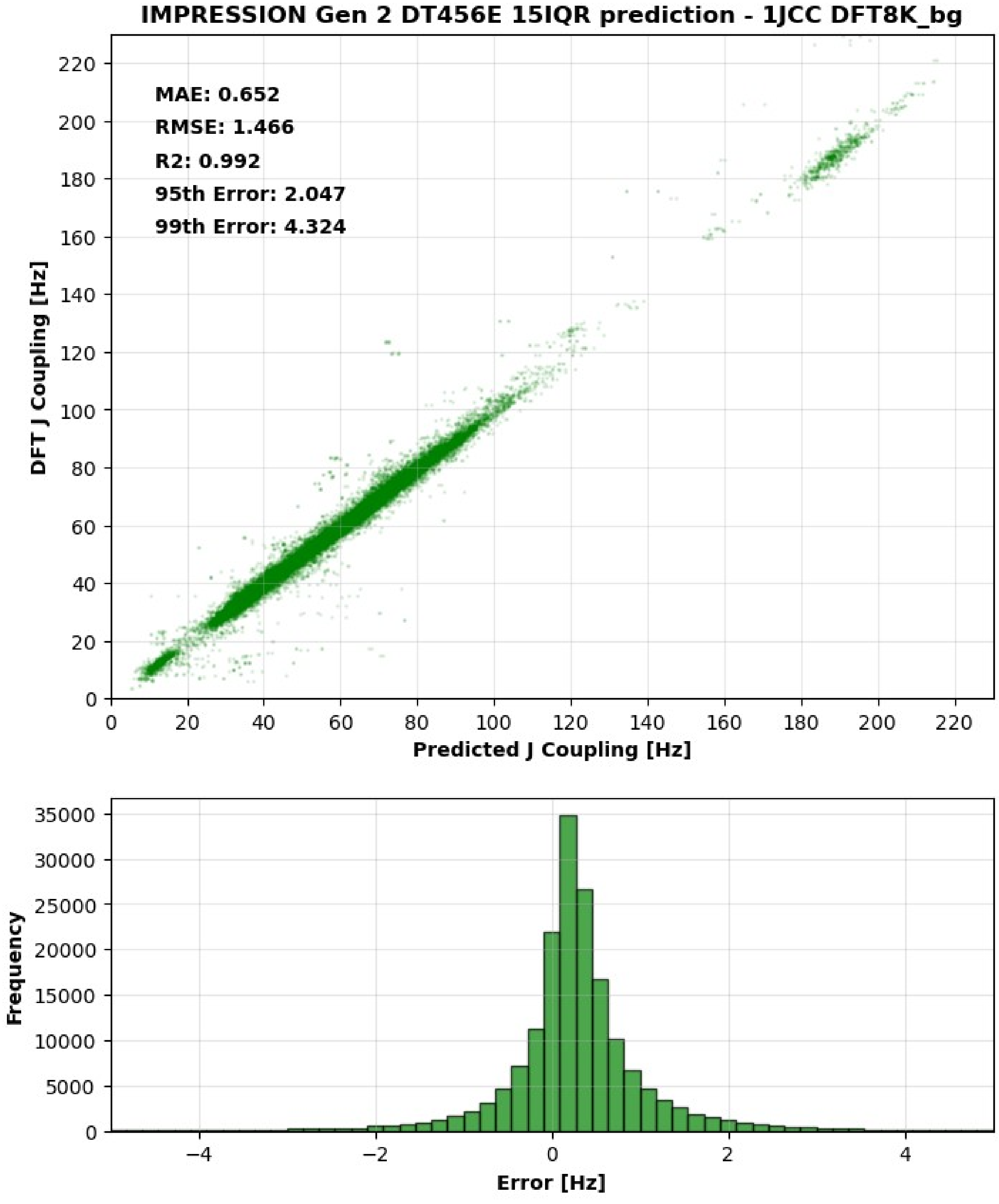

IMPRESSION Gen 2 DT456E 15IQR prediction - 1JCC DFT8K_bg
MAE: 0.652
RMSE: 1.466
R2: 0.992
95th Error: 2.047
99th Error: 4.324
DFT J Coupling [Hz]
Predicted J Coupling [Hz]
Frequency
Error [Hz]

S2.2.9 $^2J_{CC}$

Holdout

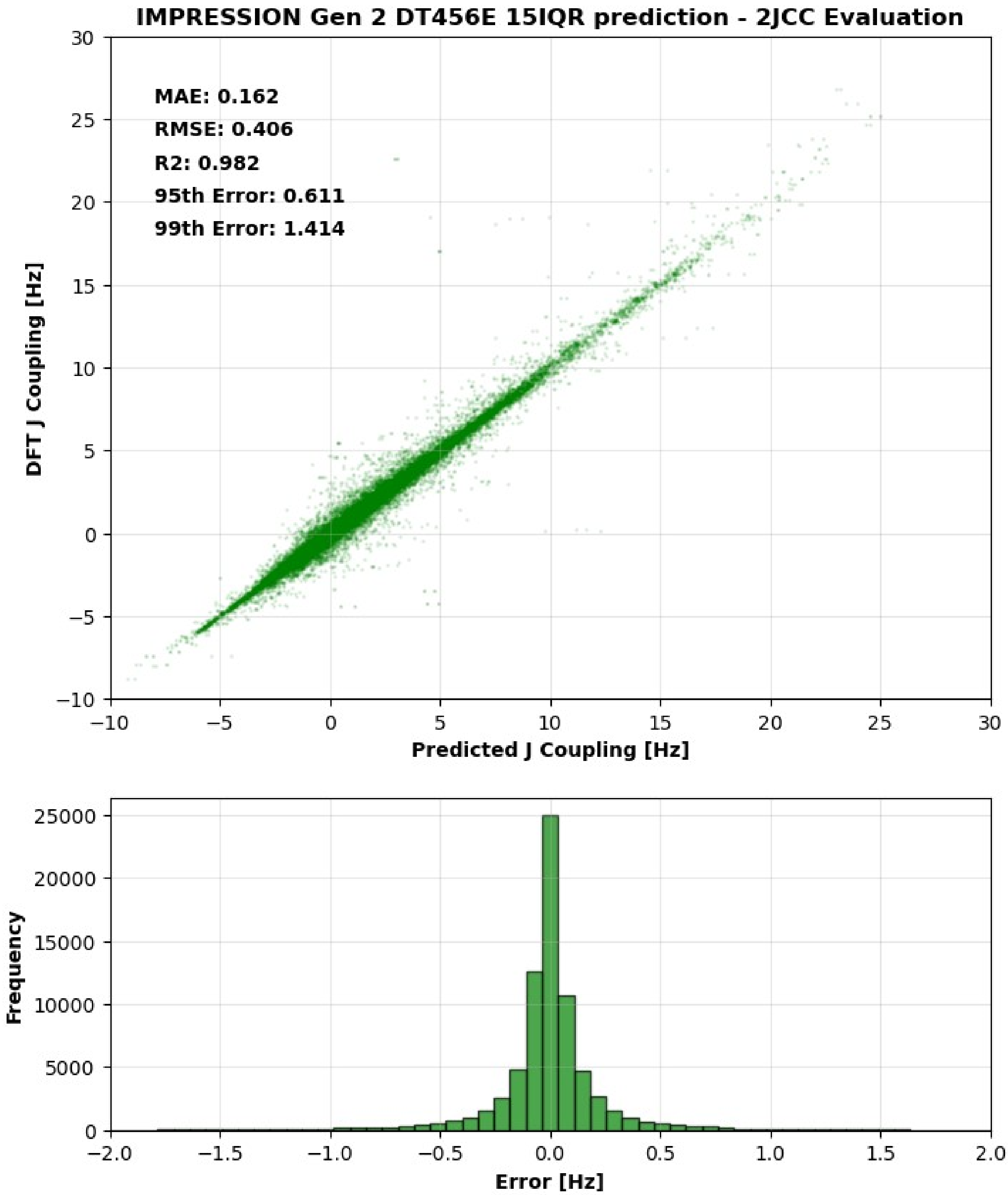

Data3

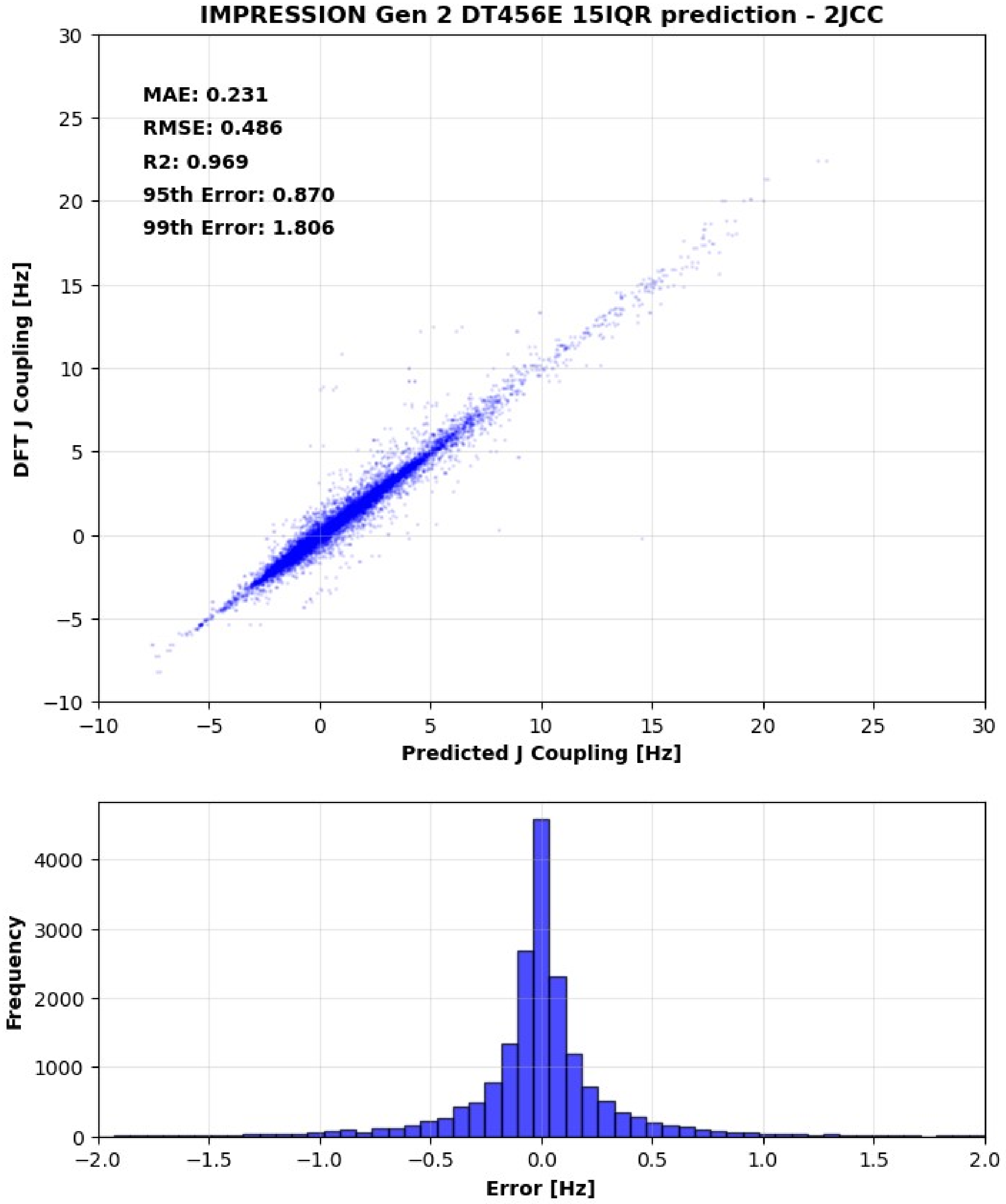
IMPRESSION Gen 2 DT456E 15IQR prediction - 2JCC
MAE: 0.231
RMSE: 0.486
R2: 0.969
95th Error: 0.870
99th Error: 1.806
DFT J Coupling [Hz]
Predicted J Coupling [Hz]
Frequency
Error [Hz]

DFT8K_bg

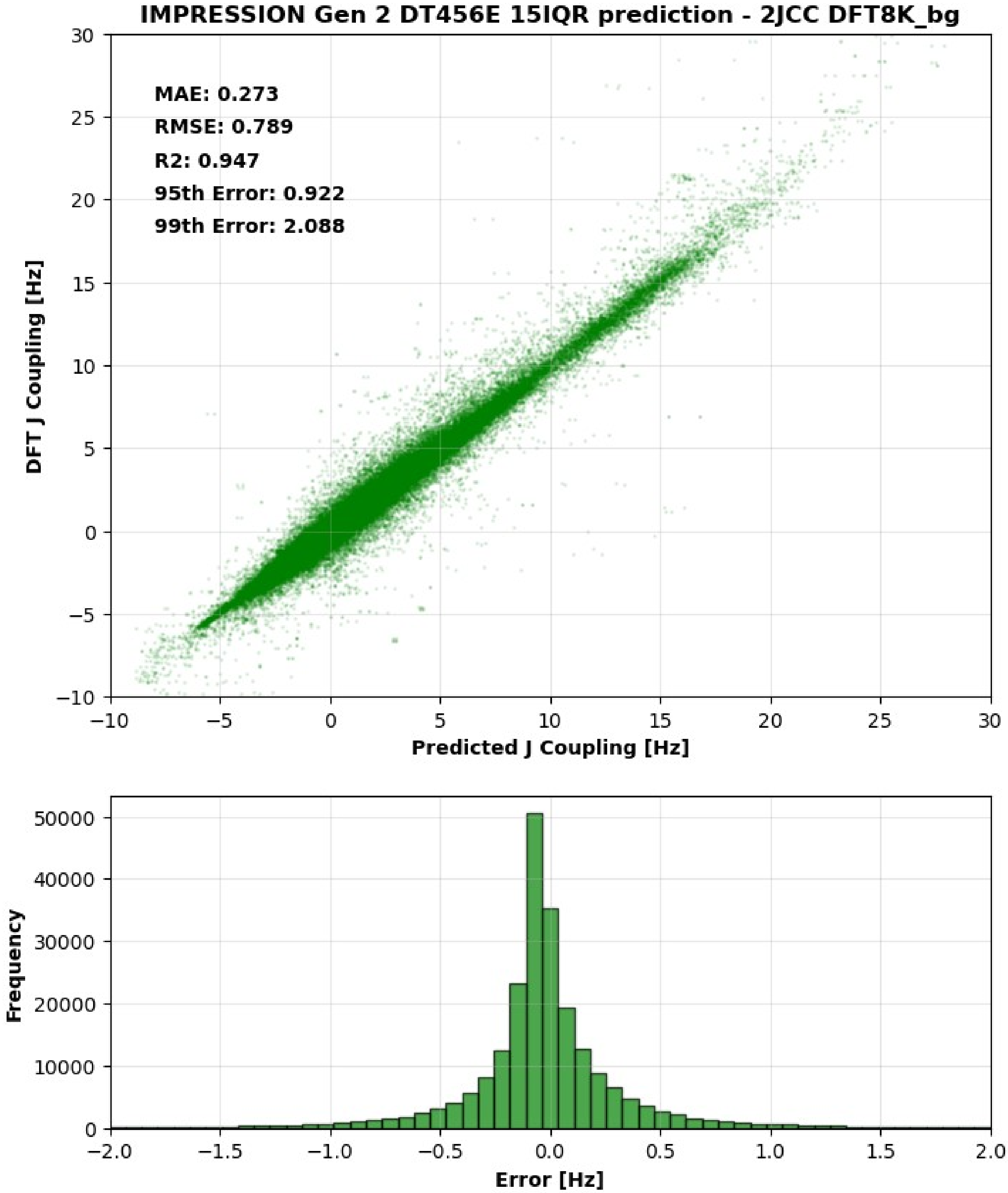

IMPRESSION Gen 2 DT456E 15IQR prediction - 2JCC DFT8K_bg
MAE: 0.273
RMSE: 0.789
R2: 0.947
95th Error: 0.922
99th Error: 2.088
DFT J Coupling [Hz]
Predicted J Coupling [Hz]
Frequency
Error [Hz]

S2.2.10 $^3J_{CC}$

Holdout

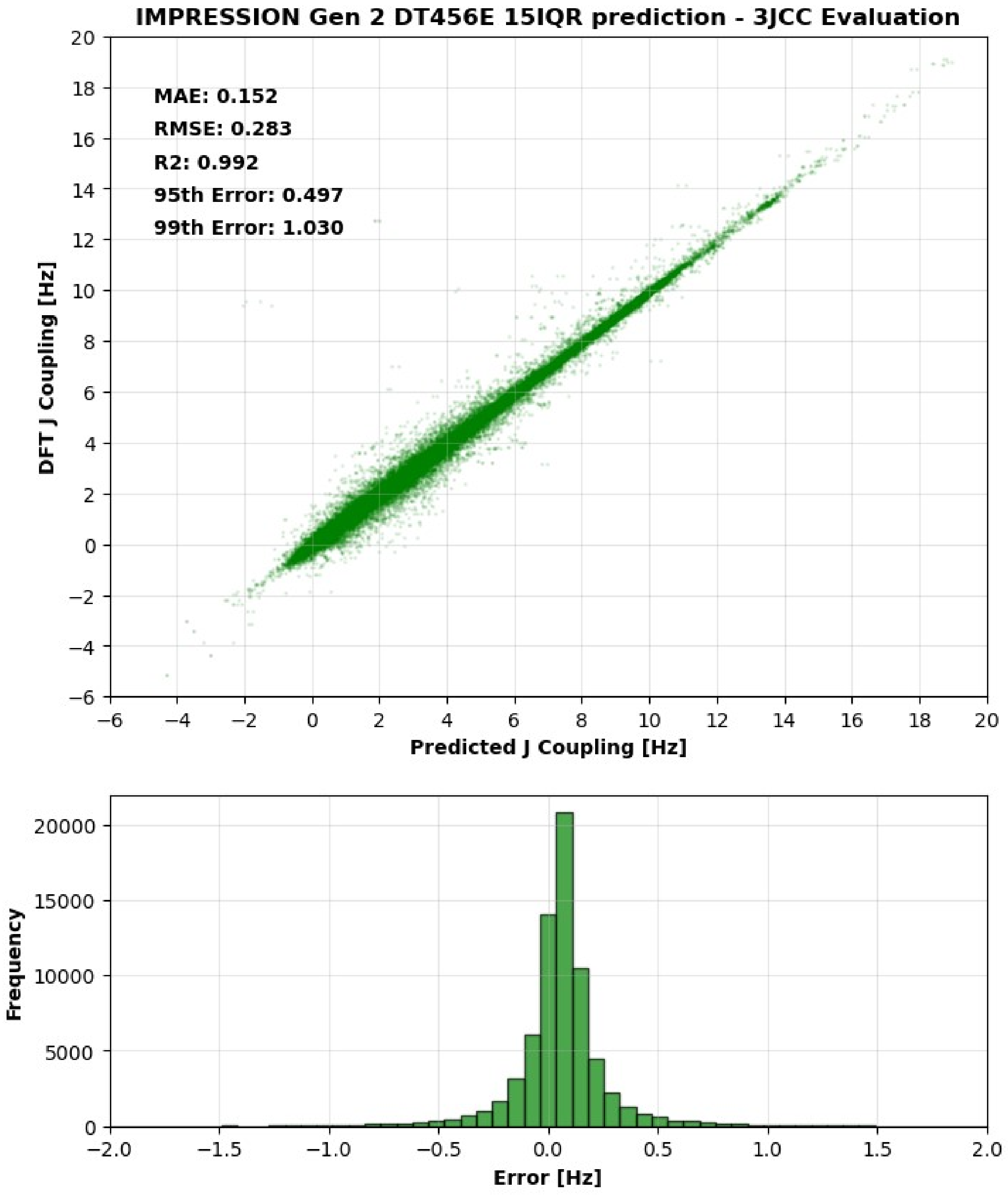

Data3

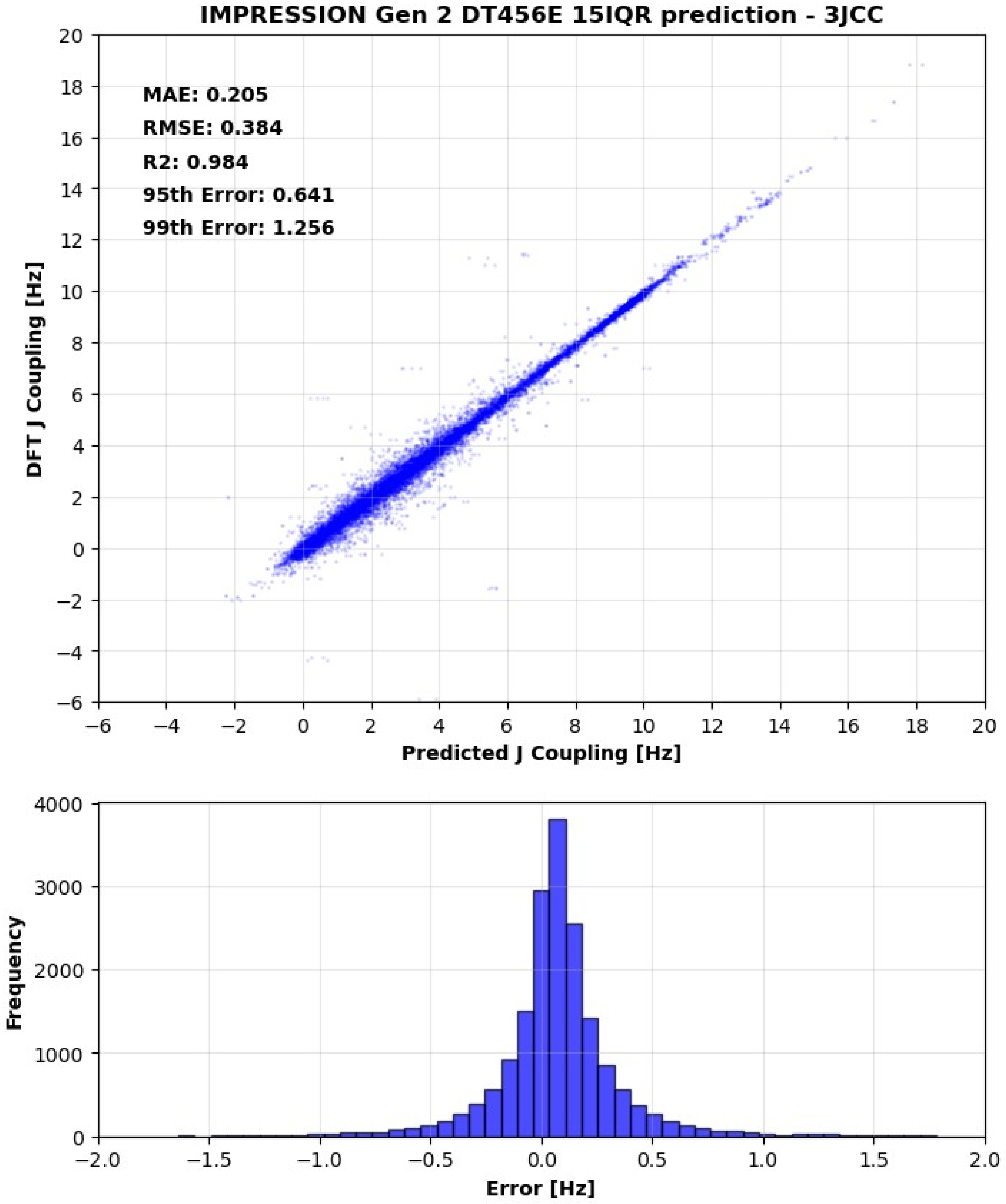

IMPRESSION Gen 2 DT456E 15IQR prediction - 3JCC
MAE: 0.205
RMSE: 0.384
R2: 0.984
95th Error: 0.641
99th Error: 1.256
DFT J Coupling [Hz]
Predicted J Coupling [Hz]
Frequency
Error [Hz]

DFT8K_bg

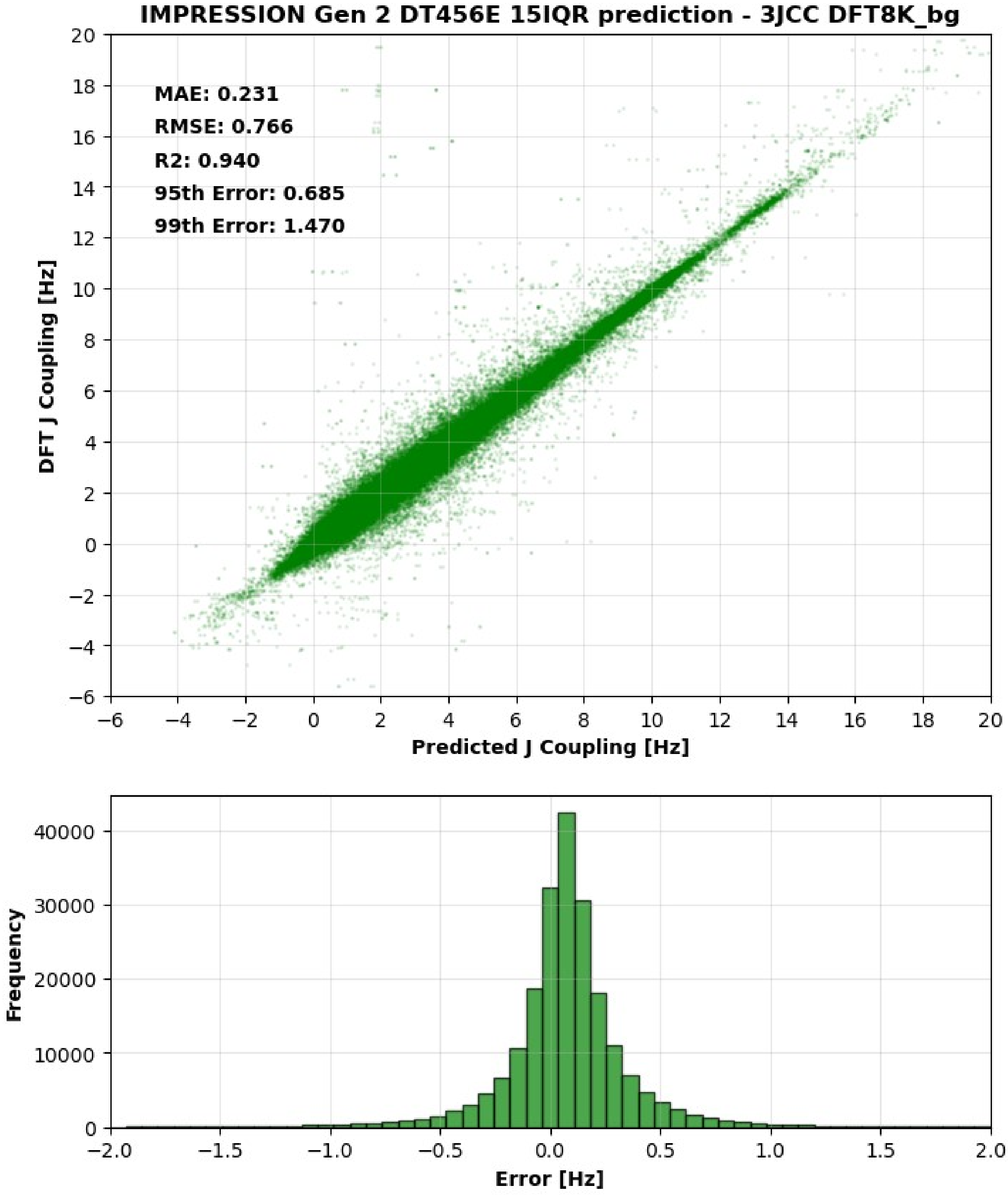
IMPRESSION Gen 2 DT456E 15IQR prediction - 3JCC DFT8K_bg
MAE: 0.231
RMSE: 0.766
R2: 0.940
95th Error: 0.685
99th Error: 1.470
DFT J Coupling [Hz]
Predicted J Coupling [Hz]
Frequency
Error [Hz]

S2.2.11 $^4J_{CC}$

Holdout

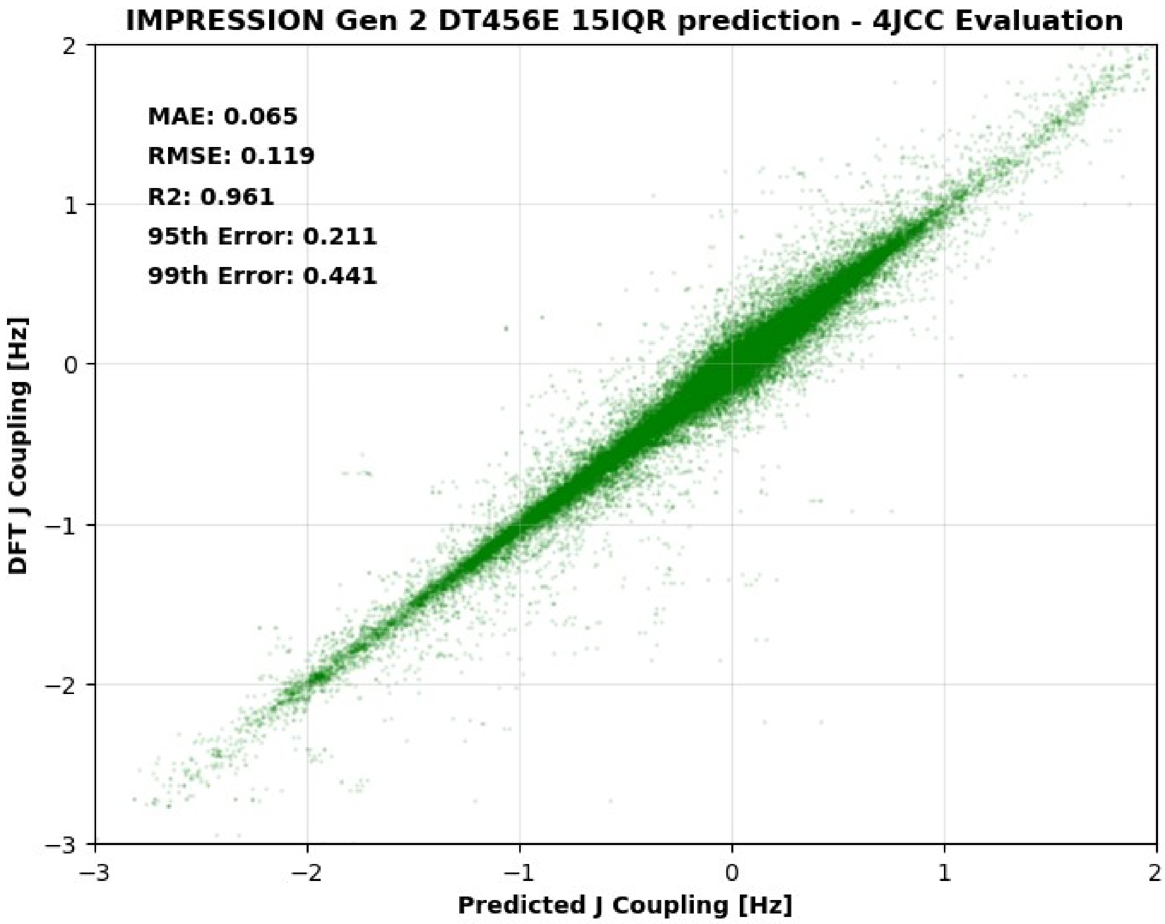

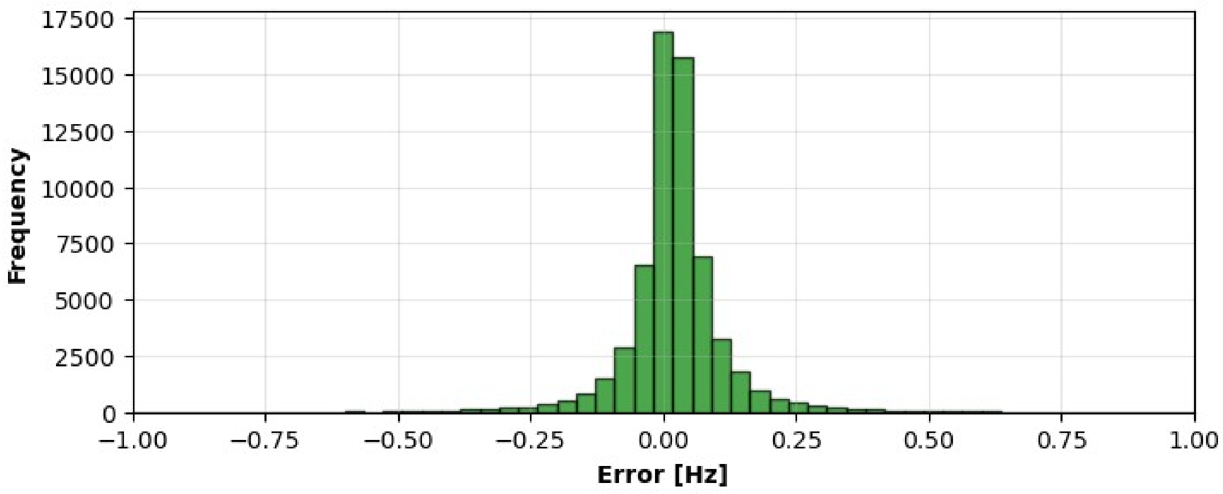

Data3

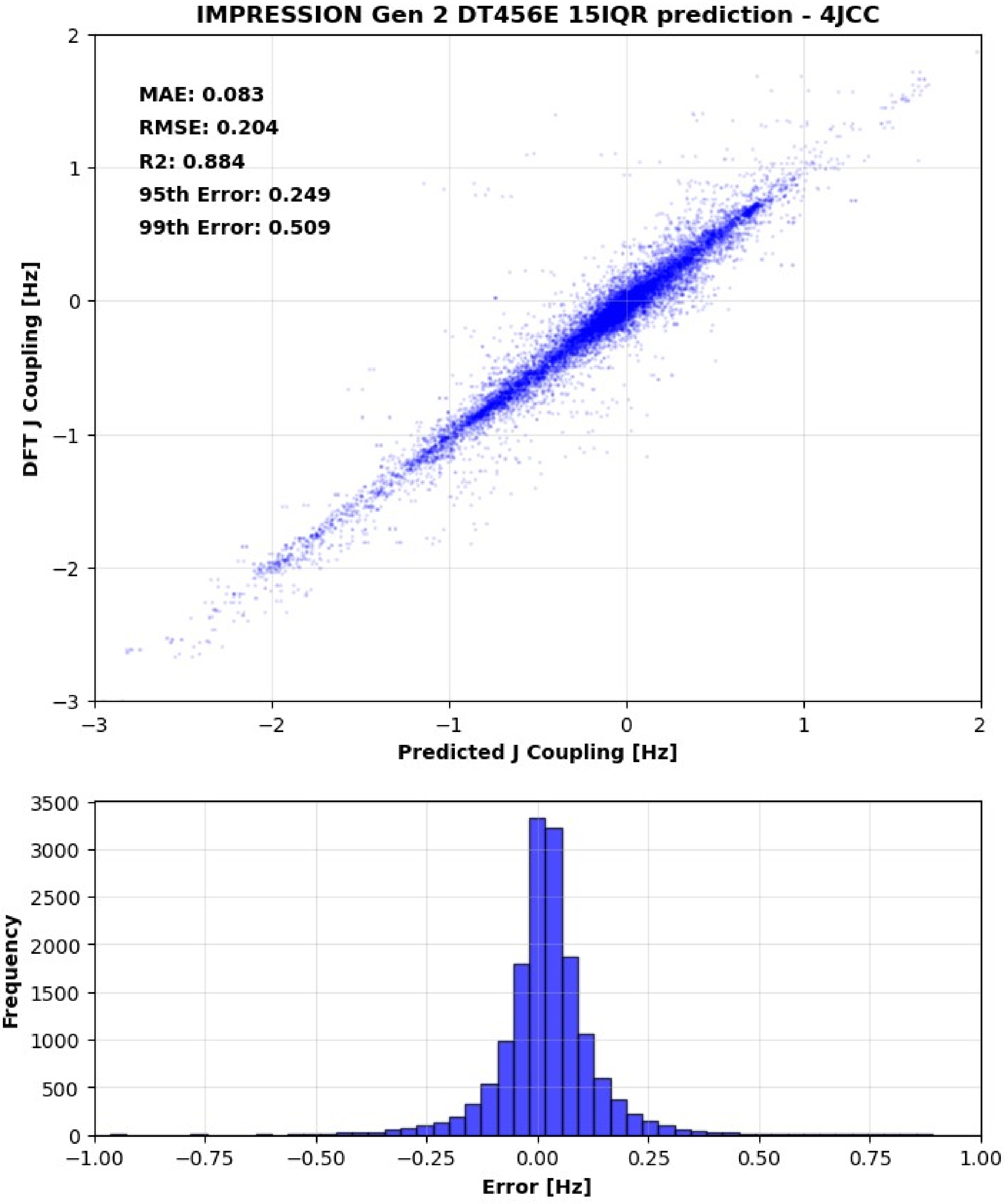
IMPRESSION Gen 2 DT456E 15IQR prediction - 4JCC
MAE: 0.083
RMSE: 0.204
R2: 0.884
95th Error: 0.249
99th Error: 0.509
DFT J Coupling [Hz]
Predicted J Coupling [Hz]
Frequency
Error [Hz]

DFT8K_bg

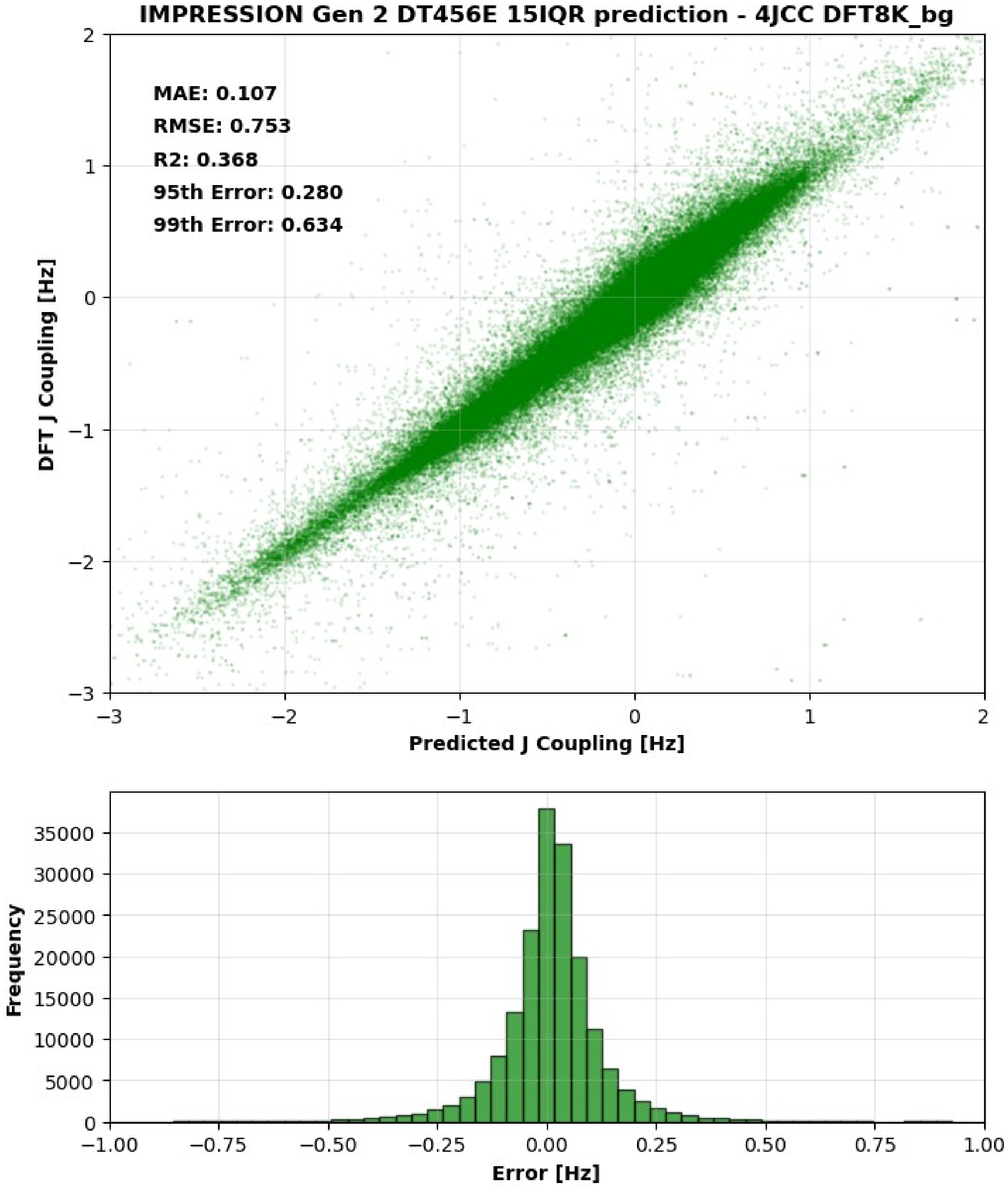

IMPRESSION Gen 2 DT456E 15IQR prediction - 4JCC DFT8K_bg
MAE: 0.107
RMSE: 0.753
R2: 0.368
95th Error: 0.280
99th Error: 0.634
DFT J Coupling [Hz]
Predicted J Coupling [Hz]
Frequency
Error [Hz]

S2.2.12 $^{1}J_{NH}$

Holdout

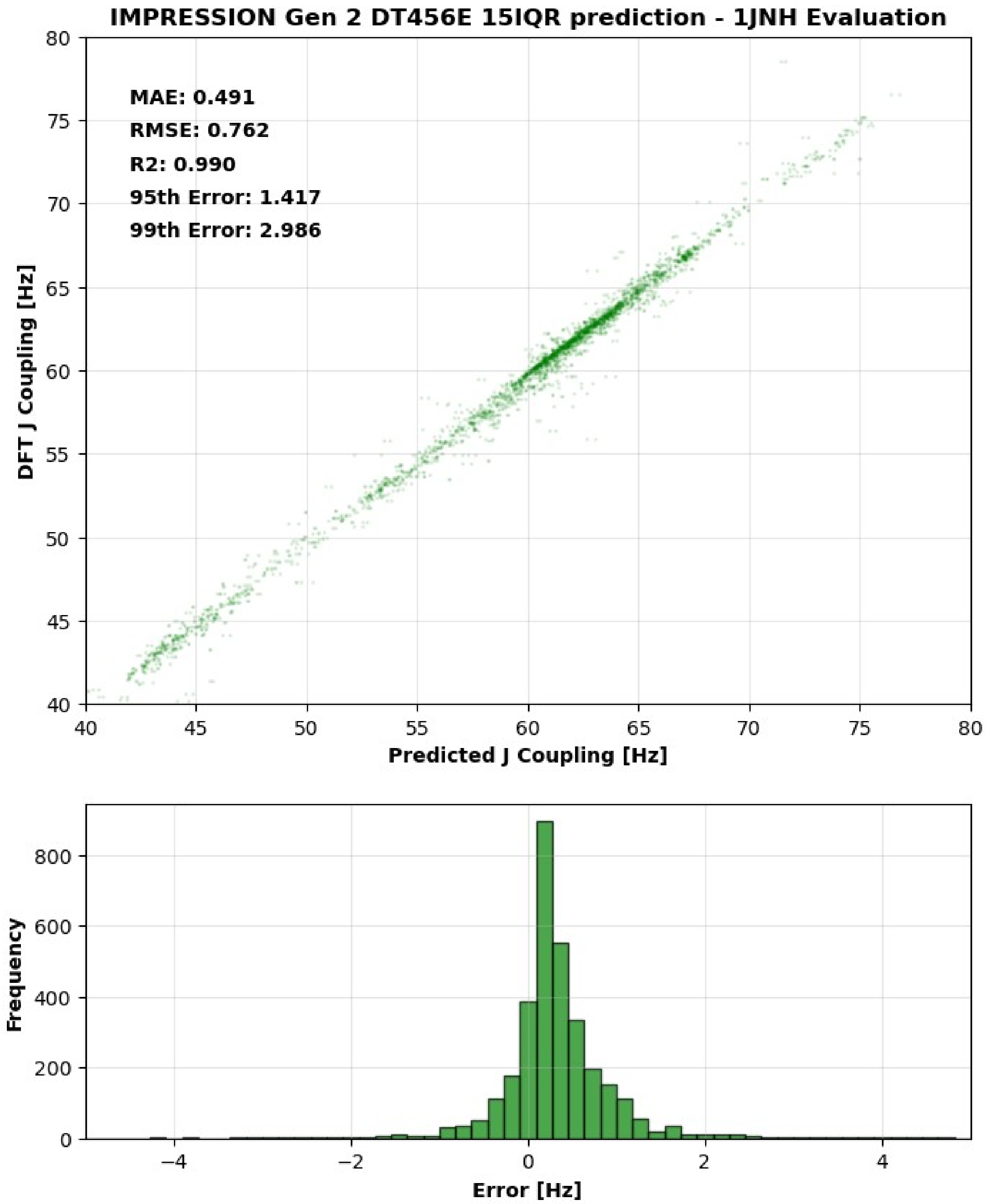

Data3

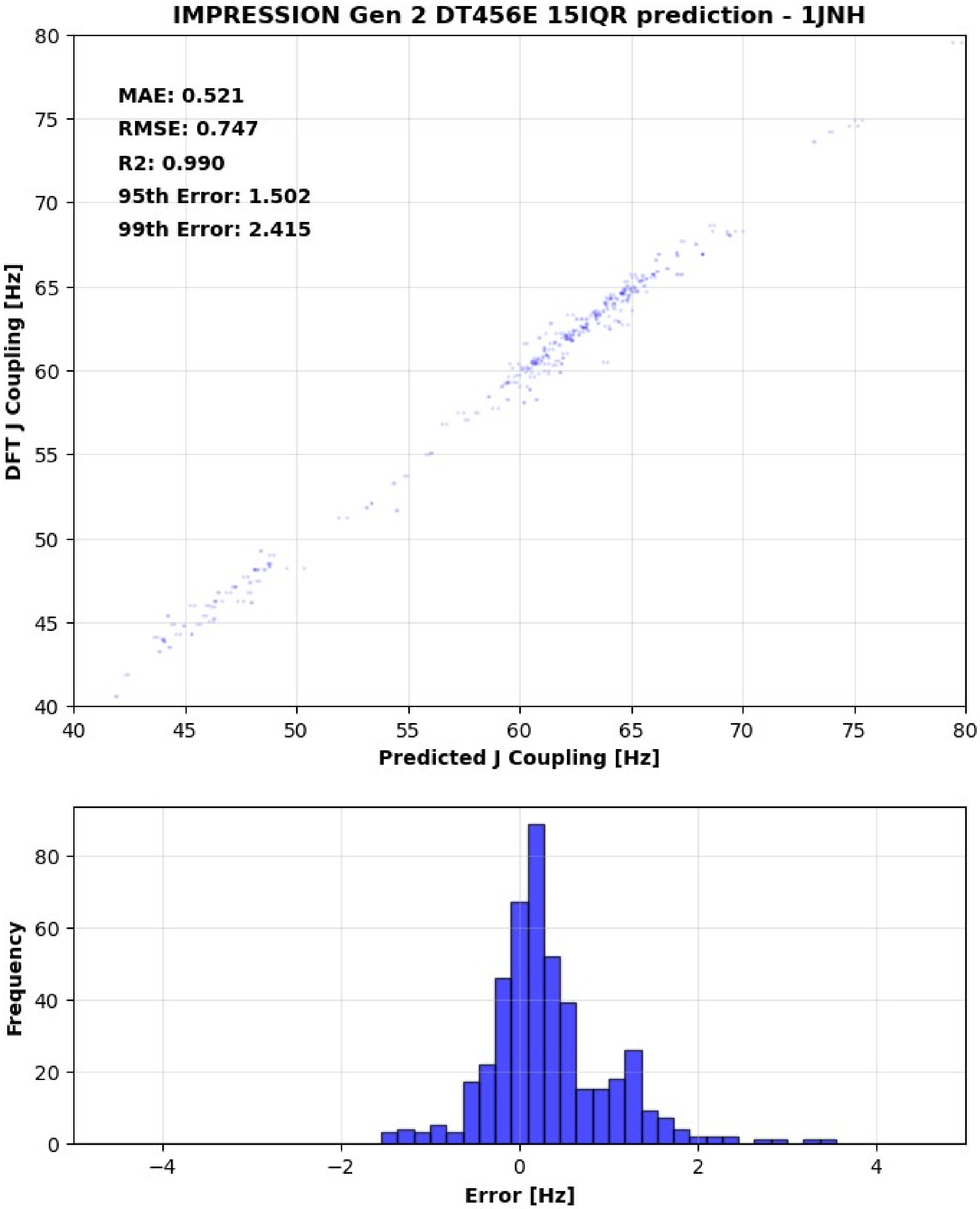
IMPRESSION Gen 2 DT456E 15IQR prediction - 1JNH
MAE: 0.521
RMSE: 0.747
R2: 0.990
95th Error: 1.502
99th Error: 2.415
DFT J Coupling [Hz]
Predicted J Coupling [Hz]
Frequency
Error [Hz]

DFT8K_bg

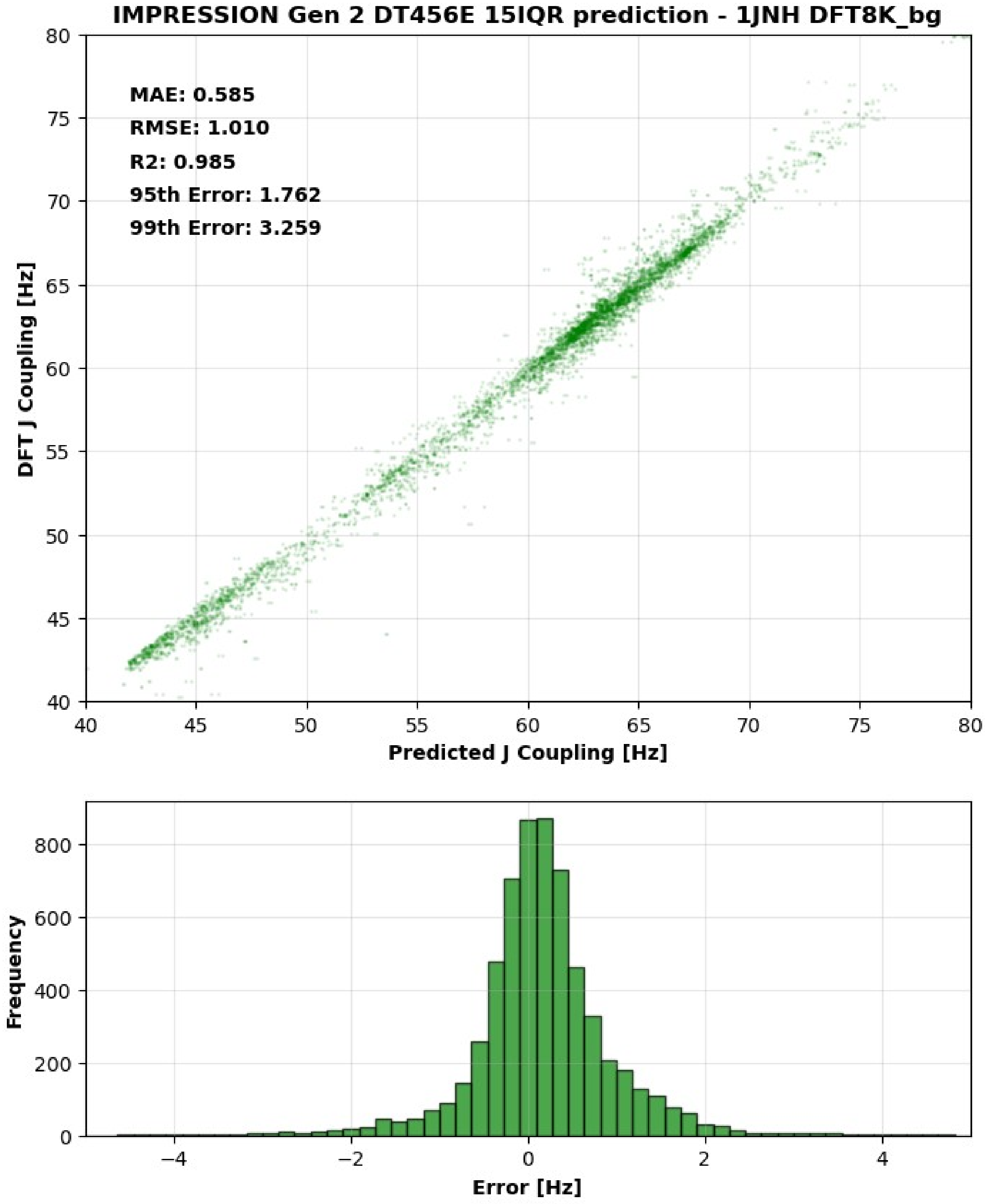
IMPRESSION Gen 2 DT456E 15IQR prediction - 1JNH DFT8K_bg
MAE: 0.585
RMSE: 1.010
R2: 0.985
95th Error: 1.762
99th Error: 3.259
DFT J Coupling [Hz]
Predicted J Coupling [Hz]
Frequency
Error [Hz]

S2.2.13 $^2J_{NH}$

Holdout

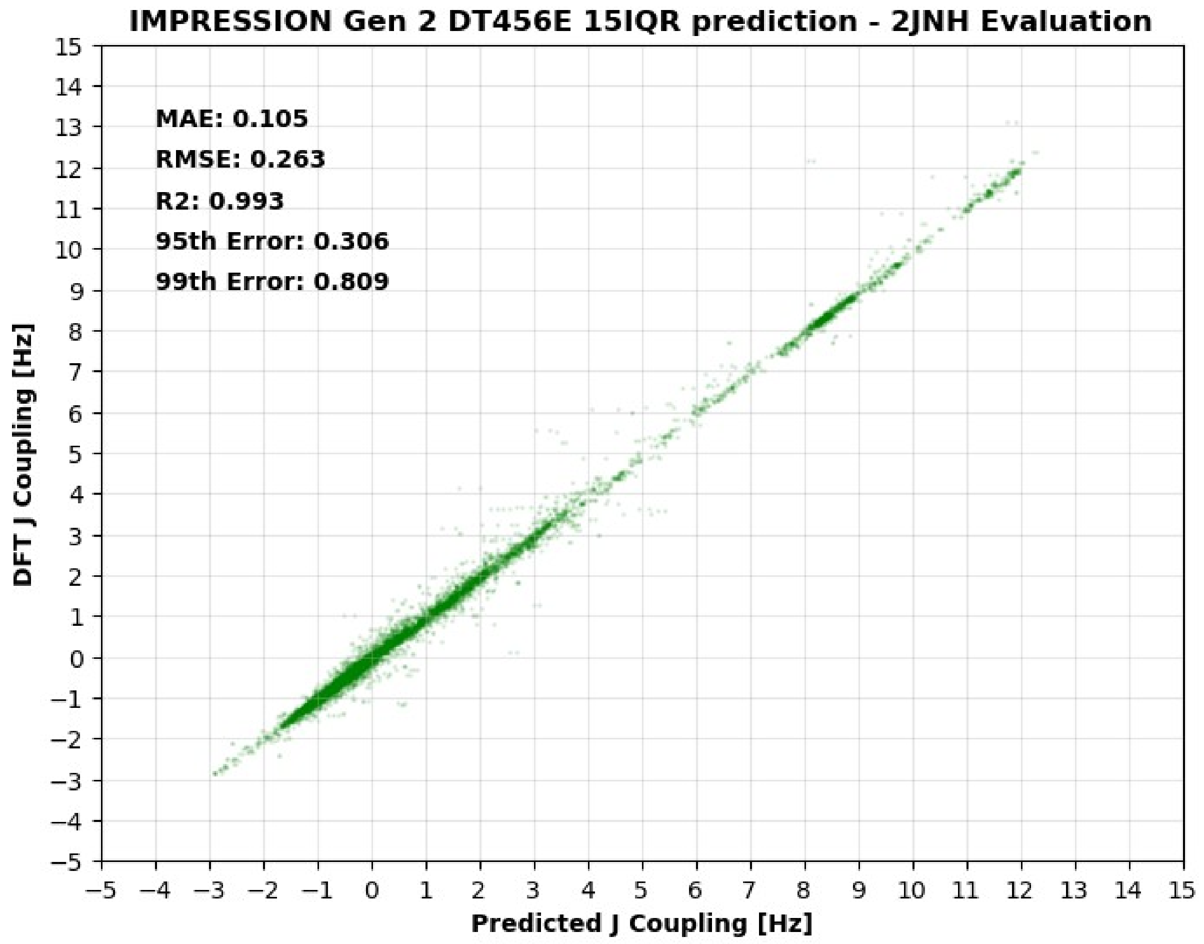

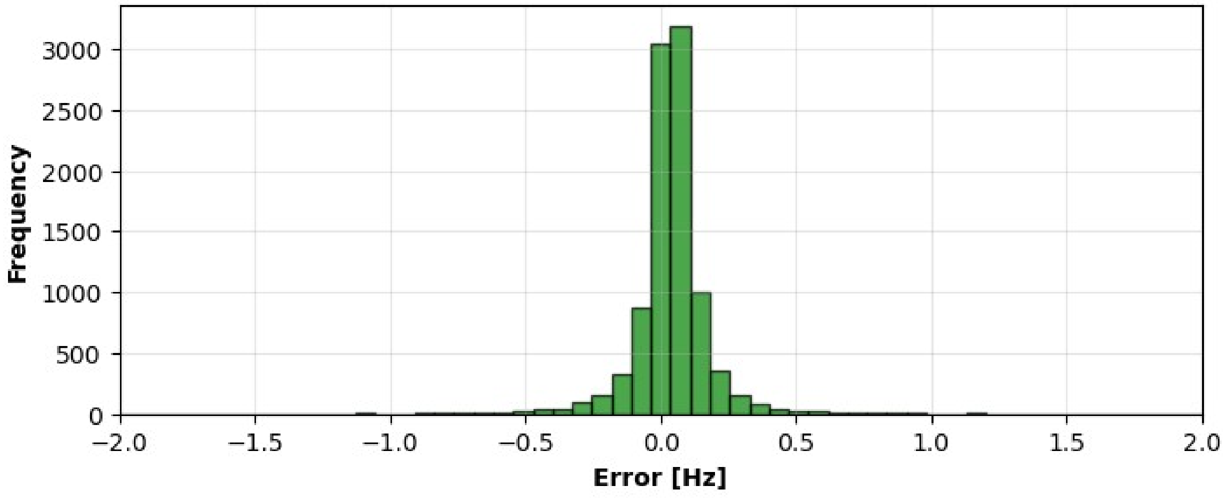

Data3

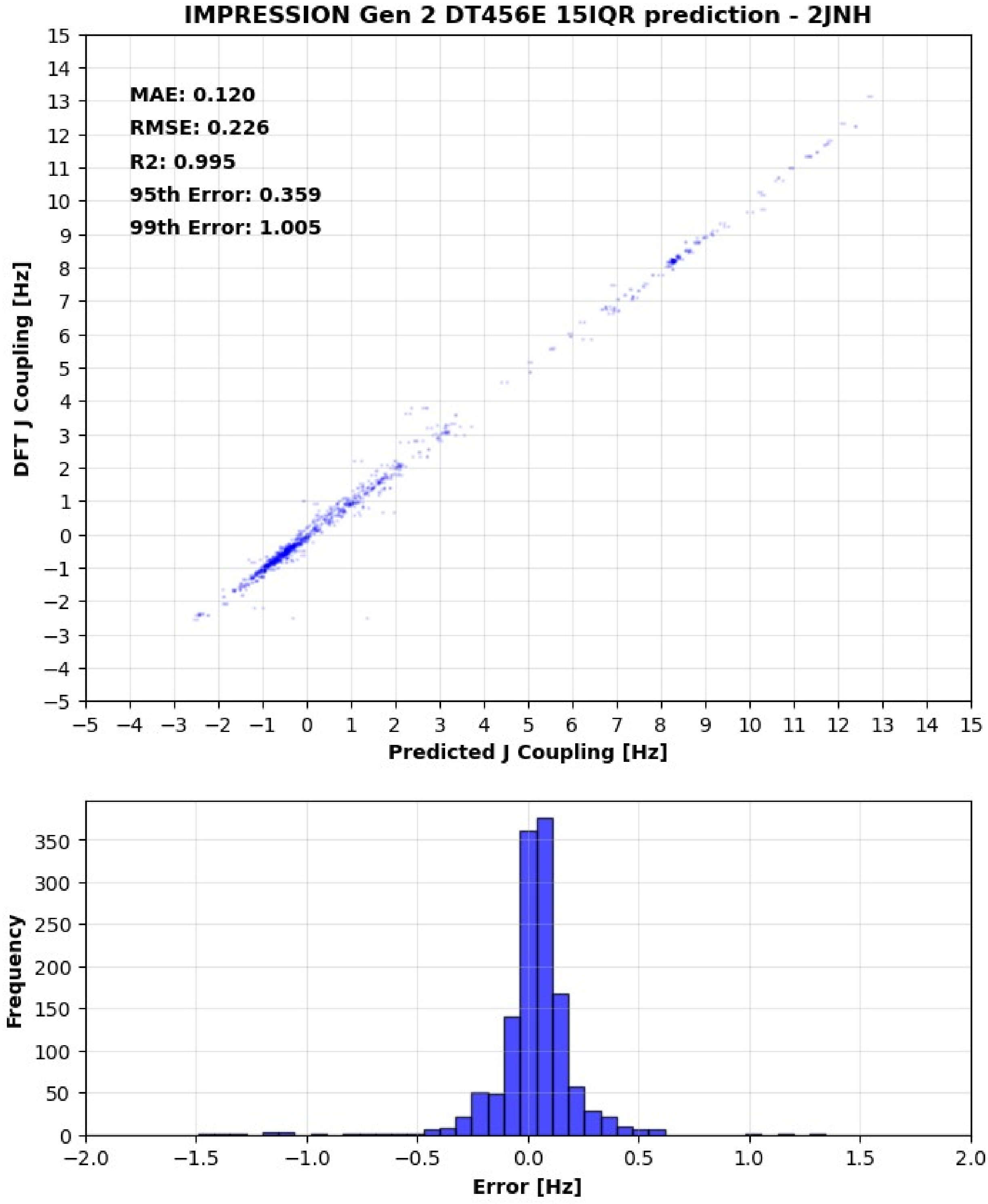
IMPRESSION Gen 2 DT456E 15IQR prediction - 2JNH
MAE: 0.120
RMSE: 0.226
R2: 0.995
95th Error: 0.359
99th Error: 1.005
DFT J Coupling [Hz]
Predicted J Coupling [Hz]
Frequency
Error [Hz]

DFT8K_bg

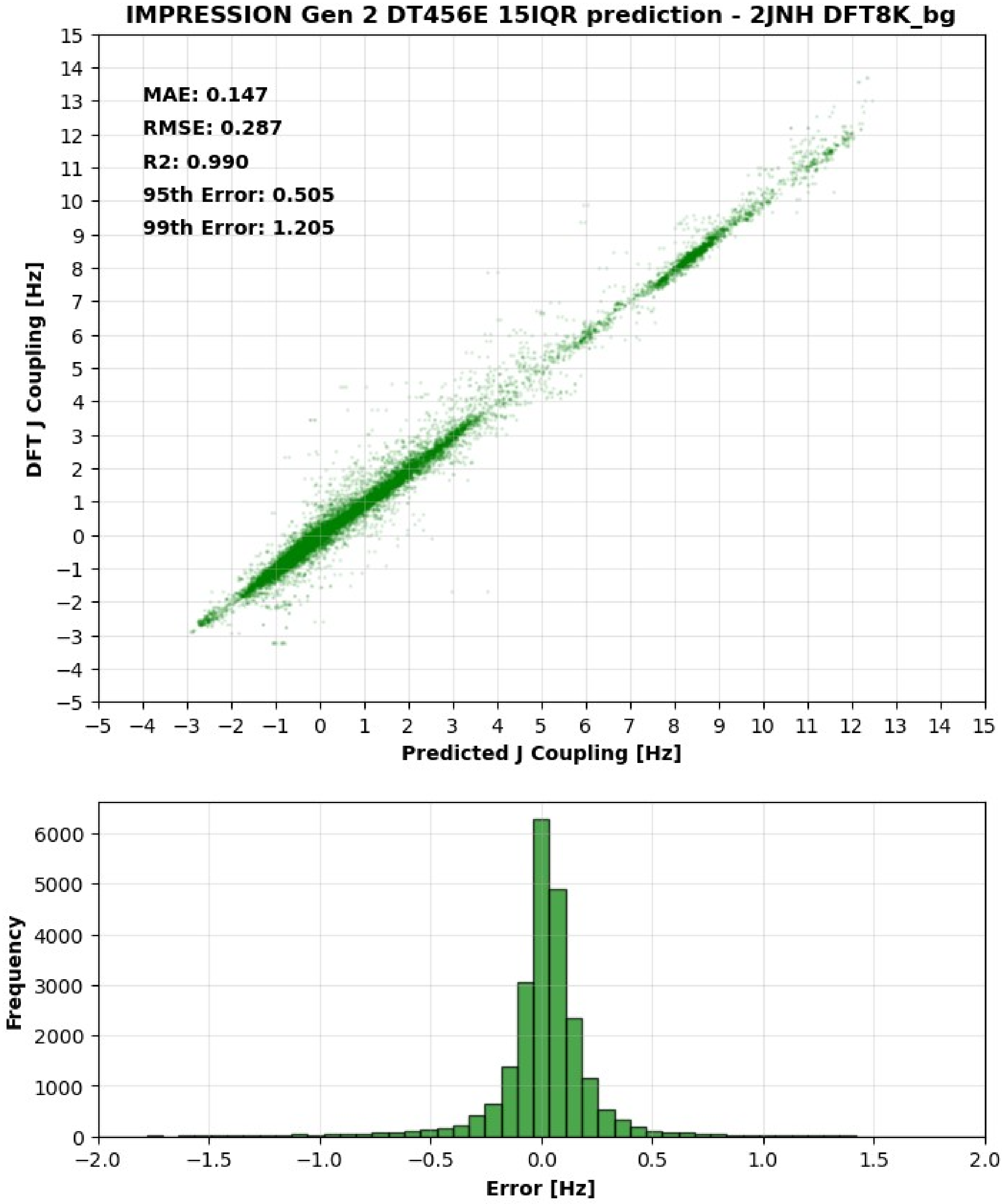

IMPRESSION Gen 2 DT456E 15IQR prediction - 2JNH DFT8K_bg
MAE: 0.147
RMSE: 0.287
R2: 0.990
95th Error: 0.505
99th Error: 1.205
DFT J Coupling [Hz]
Predicted J Coupling [Hz]
Frequency
Error [Hz]

S2.2.14 $^3J_{NH}$

Holdout

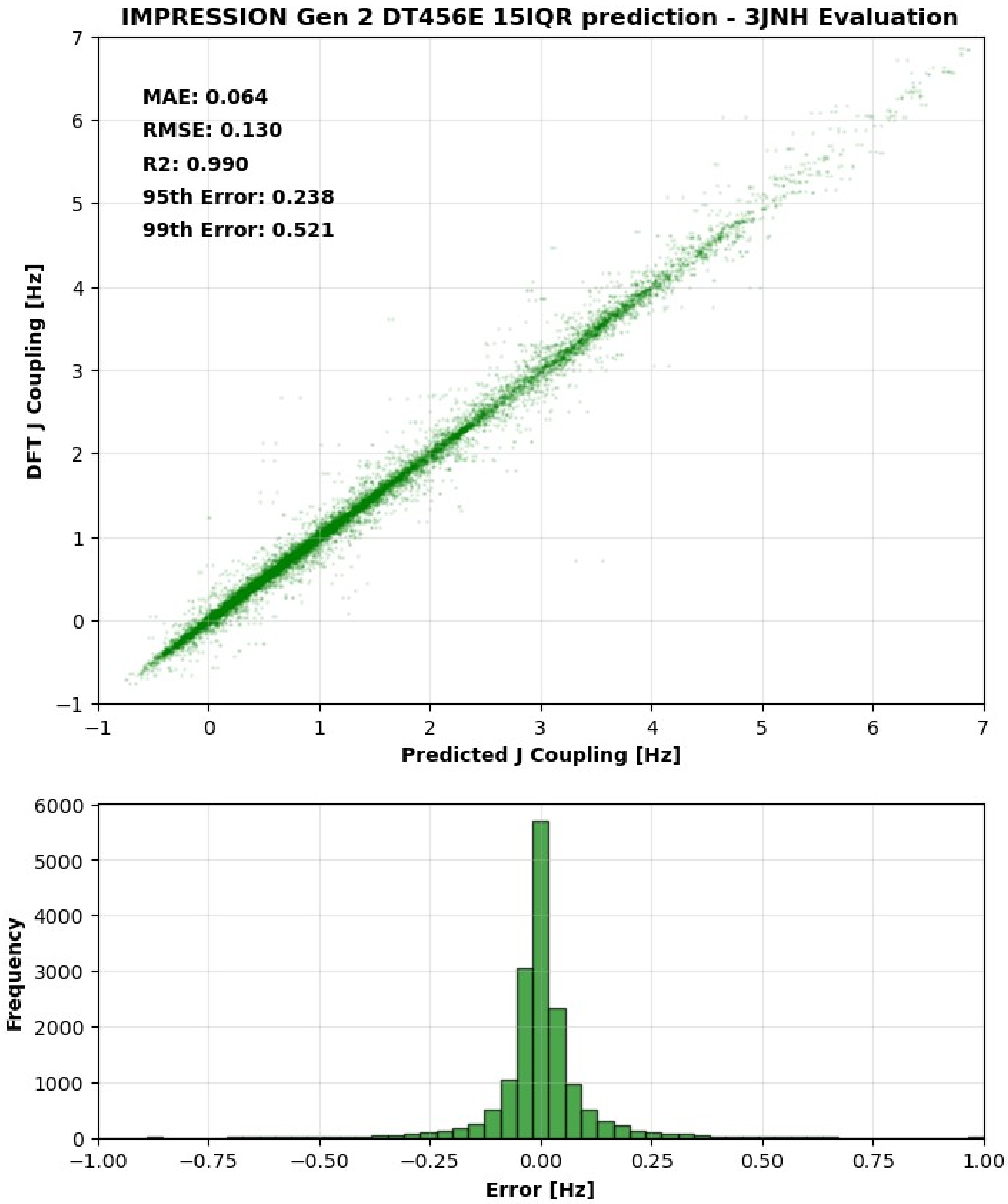

Data3

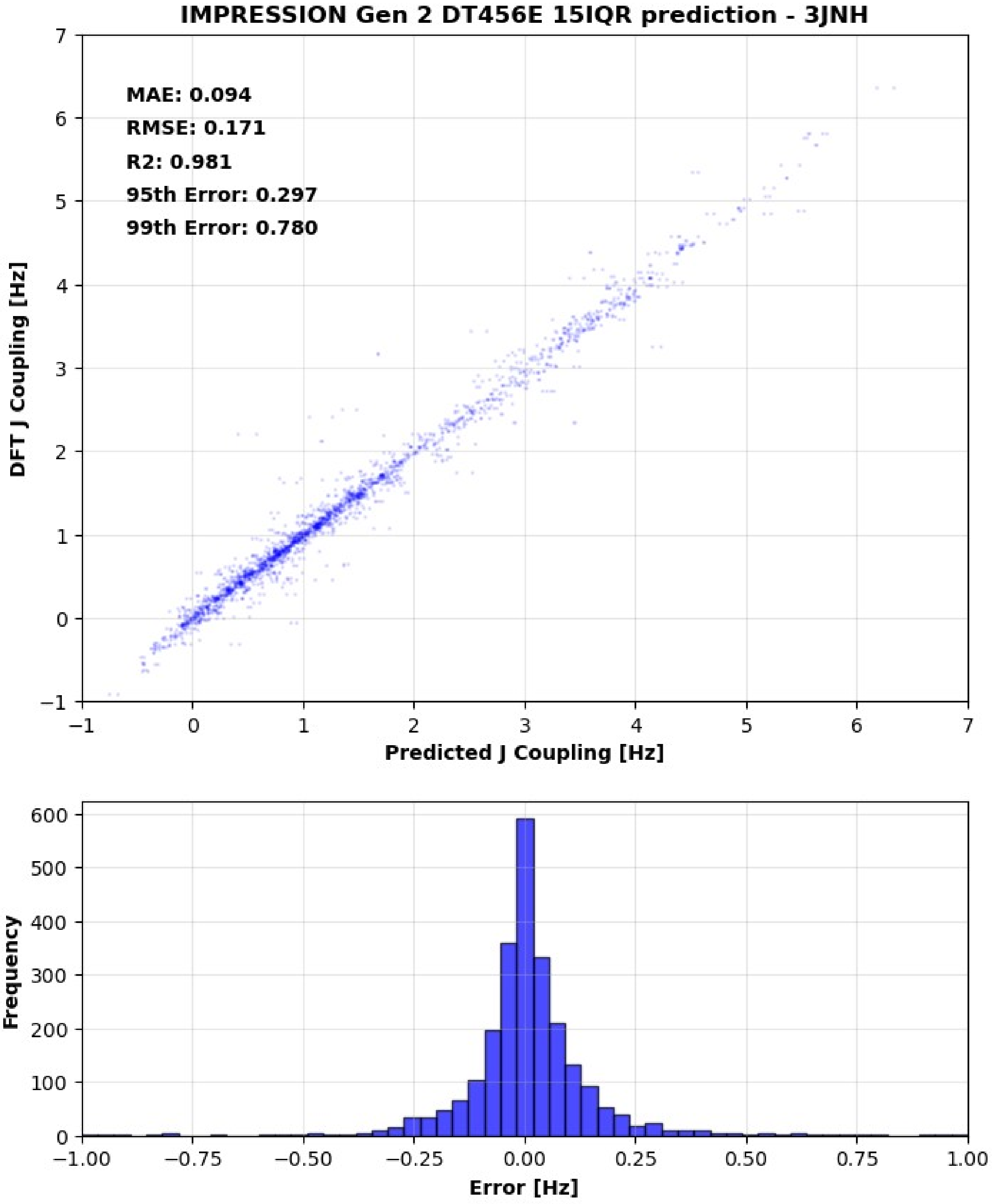
IMPRESSION Gen 2 DT456E 15IQR prediction - 3JNH
MAE: 0.094
RMSE: 0.171
R2: 0.981
95th Error: 0.297
99th Error: 0.780
DFT J Coupling [Hz]
Predicted J Coupling [Hz]
Frequency
Error [Hz]

DFT8K_bg

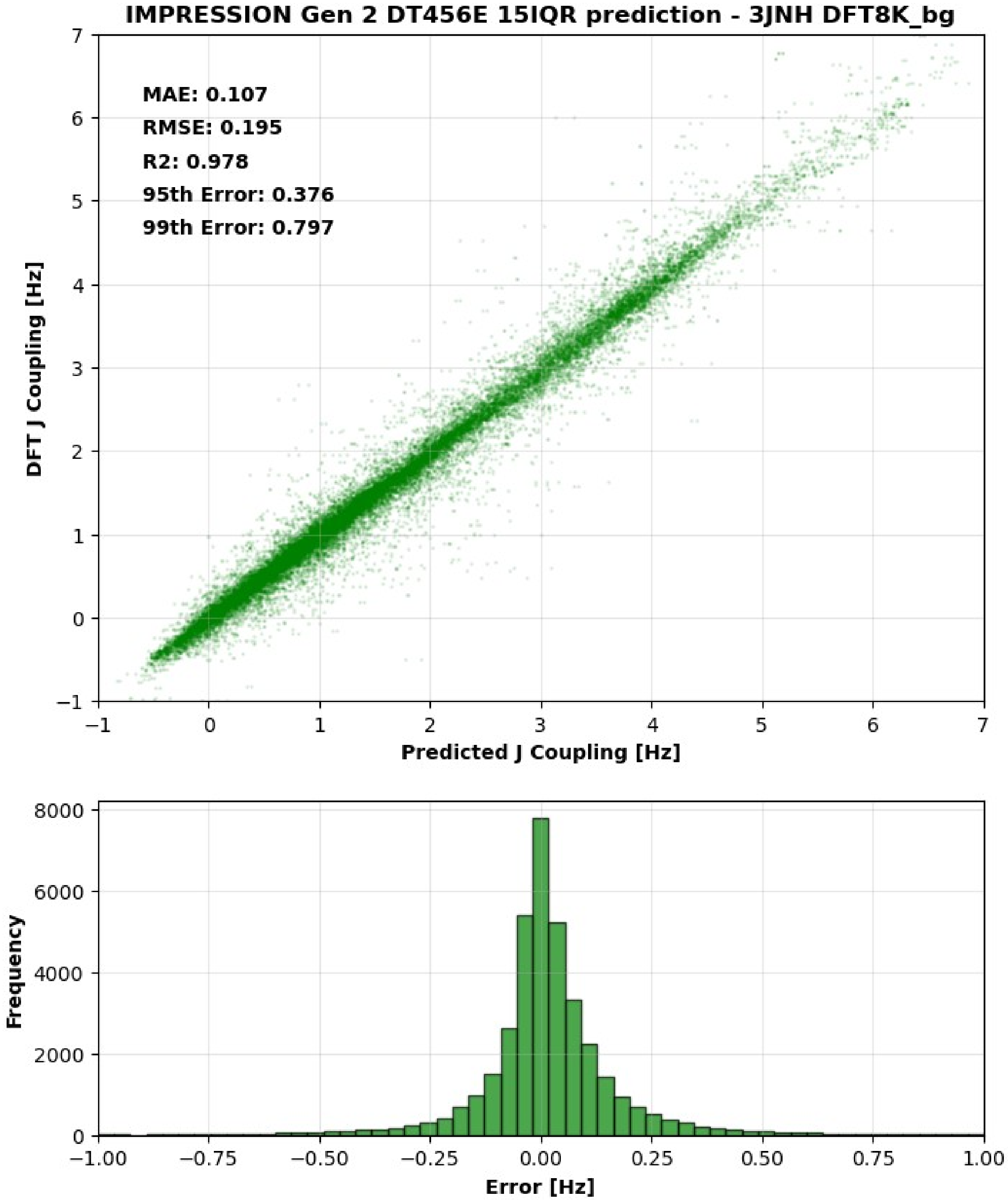
IMPRESSION Gen 2 DT456E 15IQR prediction - 3JNH DFT8K_bg
MAE: 0.107
RMSE: 0.195
R2: 0.978
95th Error: 0.376
99th Error: 0.797
DFT J Coupling [Hz]
Predicted J Coupling [Hz]
Frequency
Error [Hz]

S2.2.15 $^4J_{NH}$

Holdout

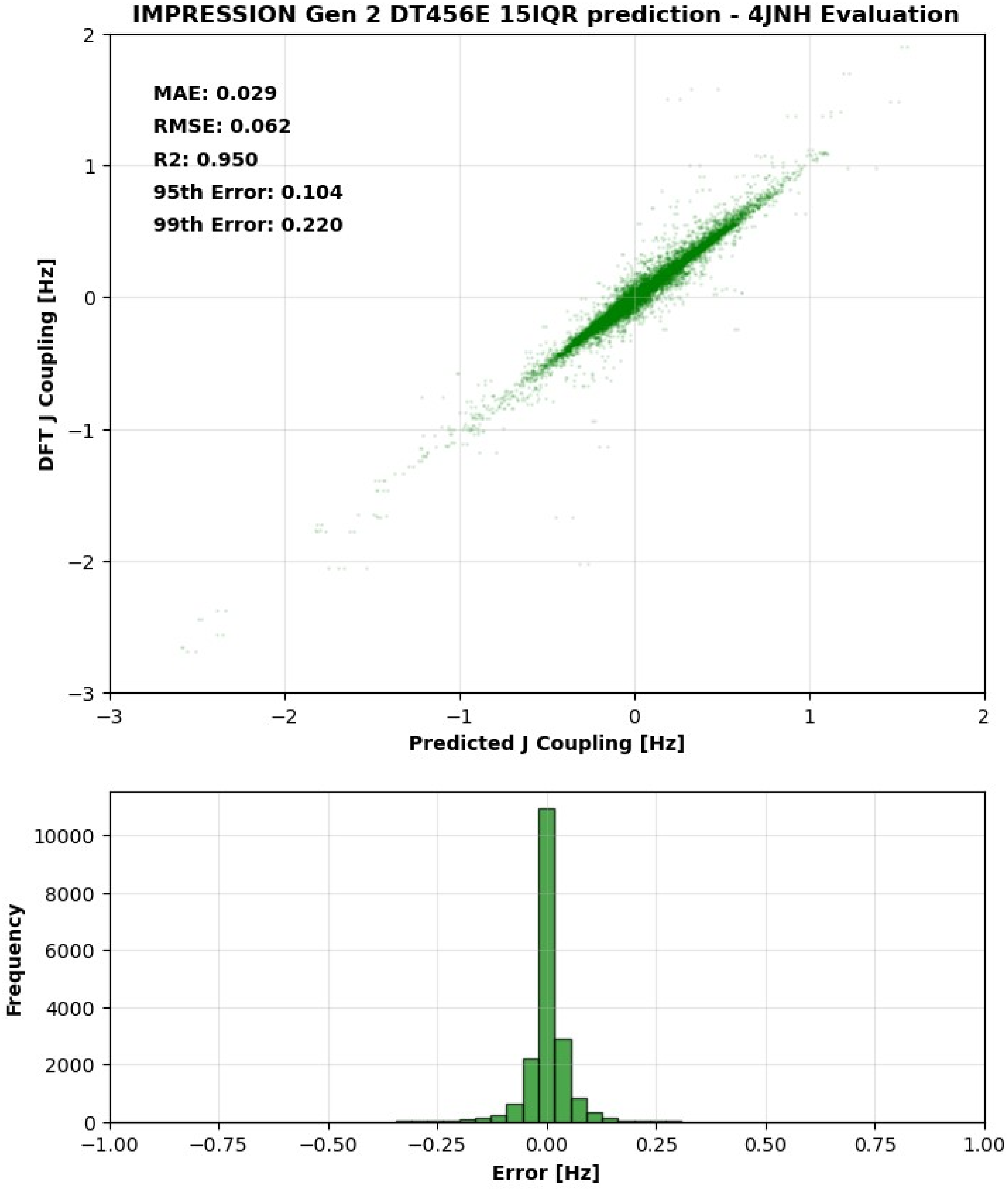

Data3

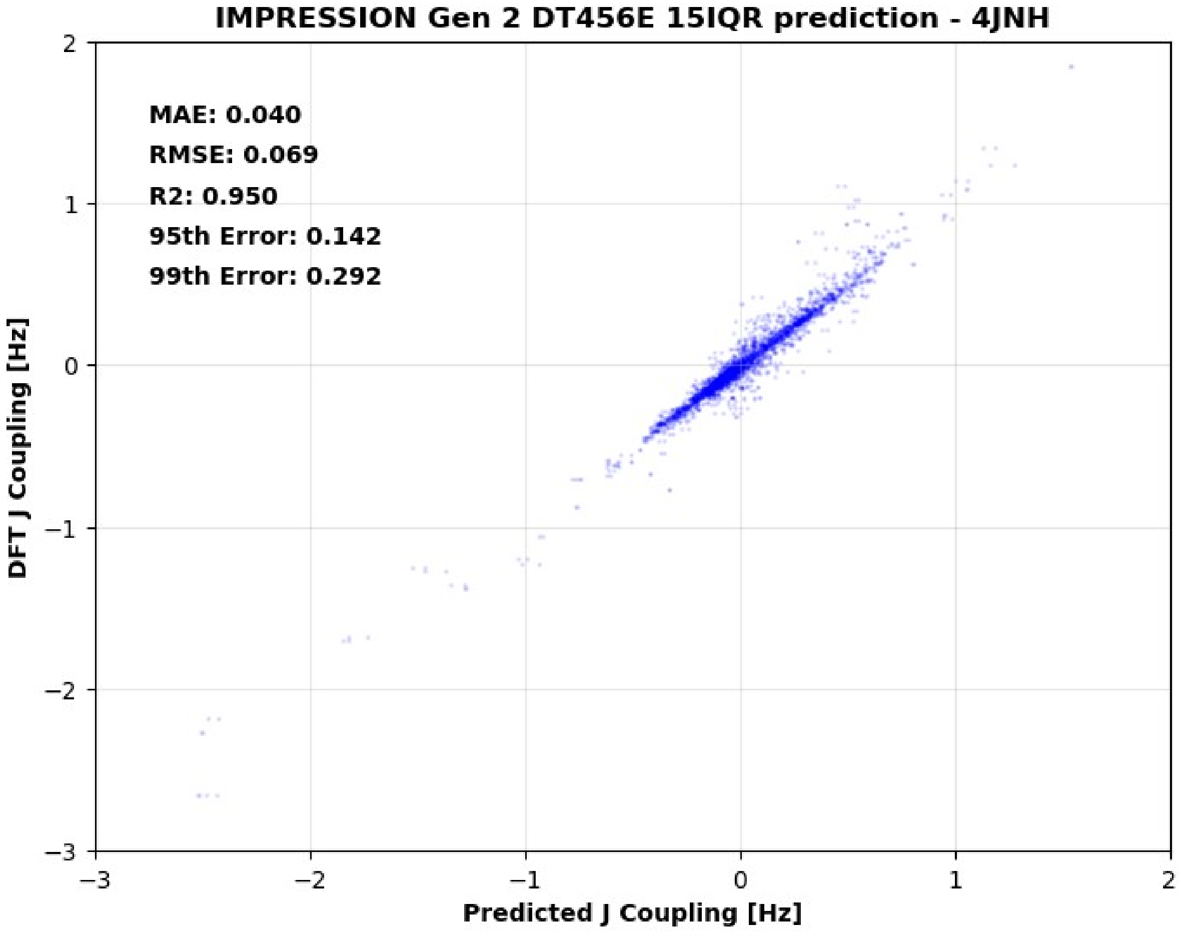
IMPRESSION Gen 2 DT456E 15IQR prediction - 4JNH
MAE: 0.040
RMSE: 0.069
R2: 0.950
95th Error: 0.142
99th Error: 0.292
DFT J Coupling [Hz]
Predicted J Coupling [Hz]
2
1
0
−1
−2
−3
−3
−2
−1
0
1
2

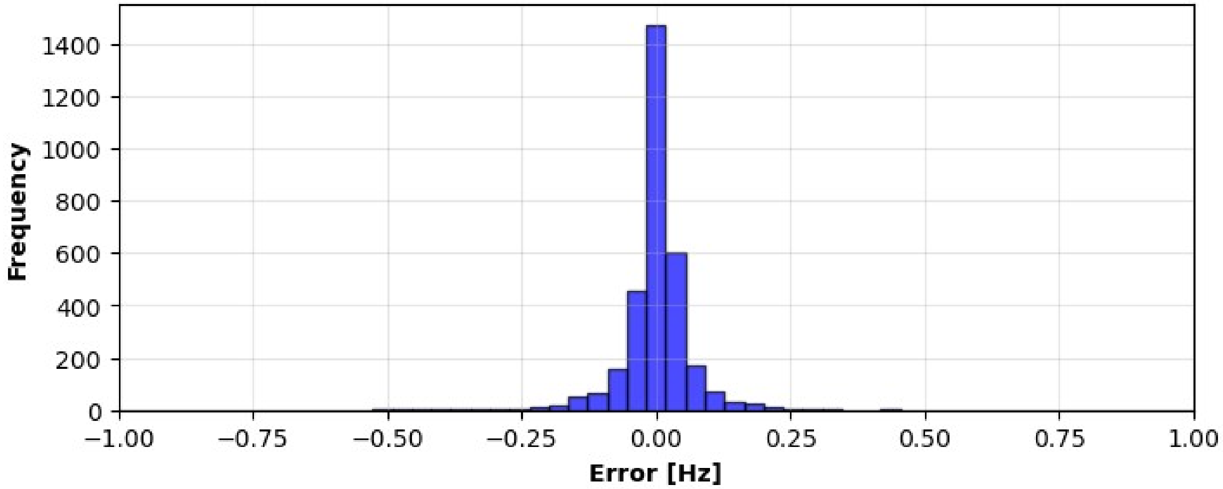
Frequency
Error [Hz]
1400
1200
1000
800
600
400
200
0
−1.00
−0.75
−0.50
−0.25
0.00
0.25
0.50
0.75
1.00

DFT8K_bg

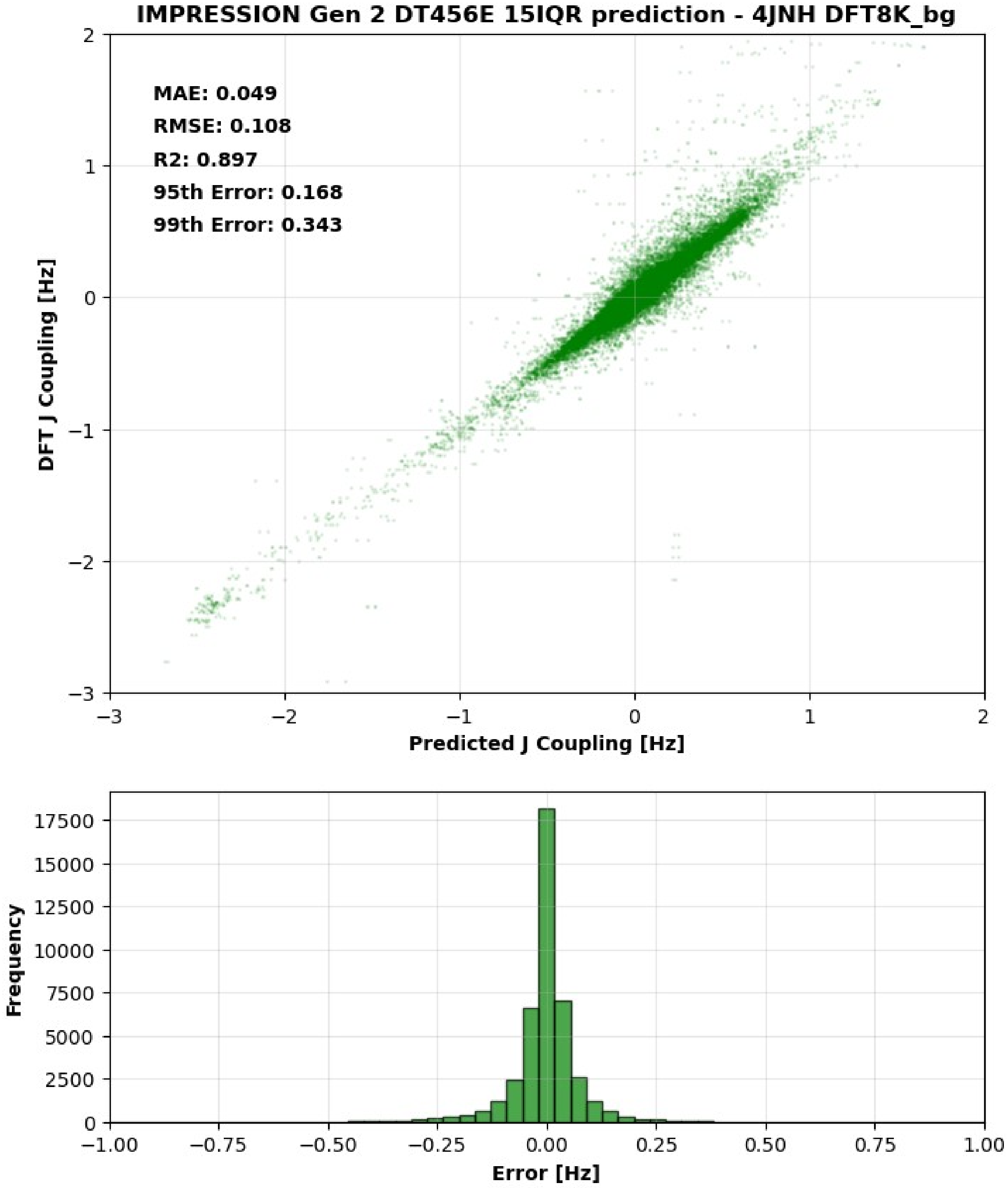
IMPRESSION Gen 2 DT456E 15IQR prediction - 4JNH DFT8K_bg
MAE: 0.049
RMSE: 0.108
R2: 0.897
95th Error: 0.168
99th Error: 0.343
DFT J Coupling [Hz]
Predicted J Coupling [Hz]
Frequency
Error [Hz]

S2.2.16 $^1J_{NC}$

Holdout

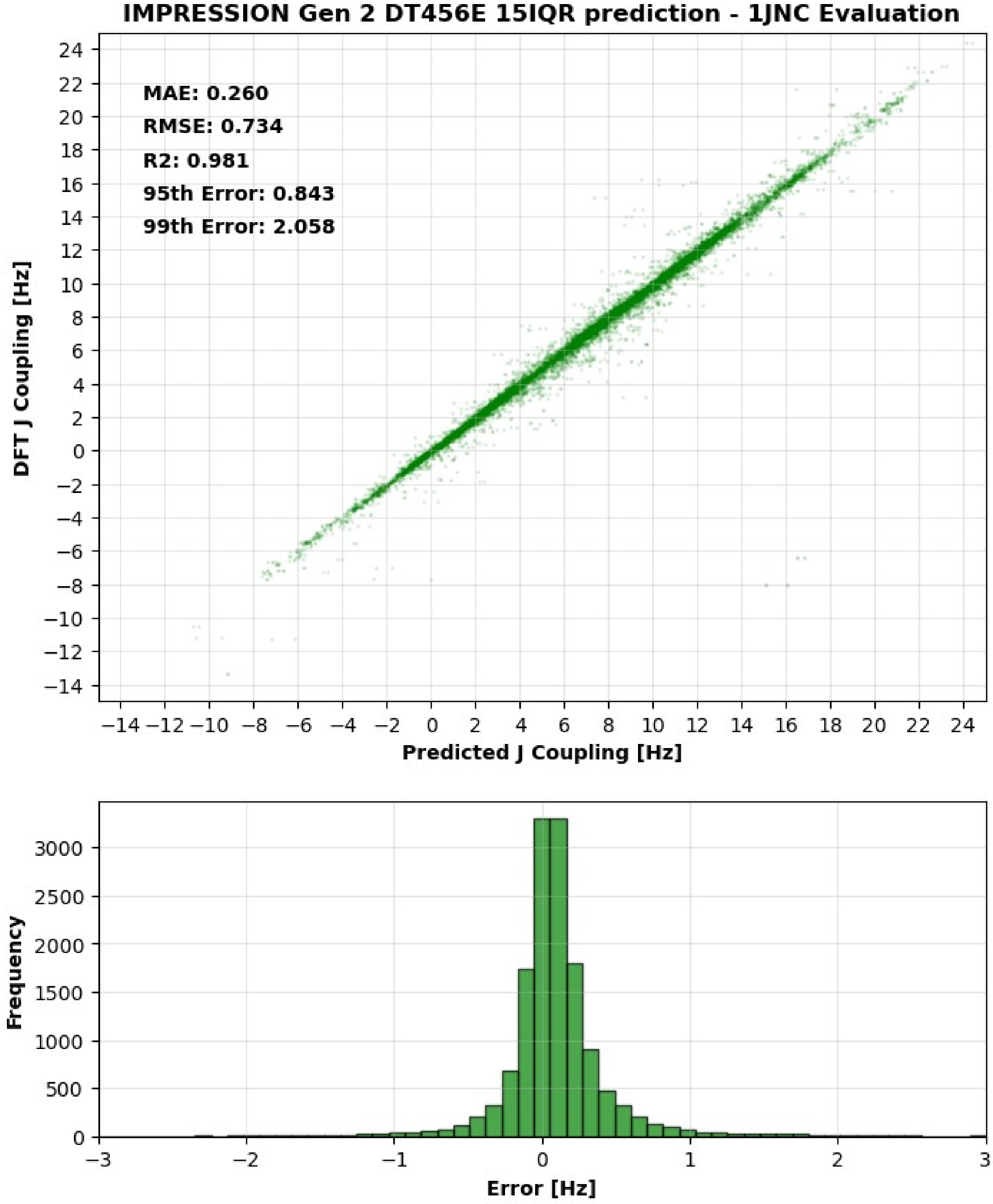

Data3

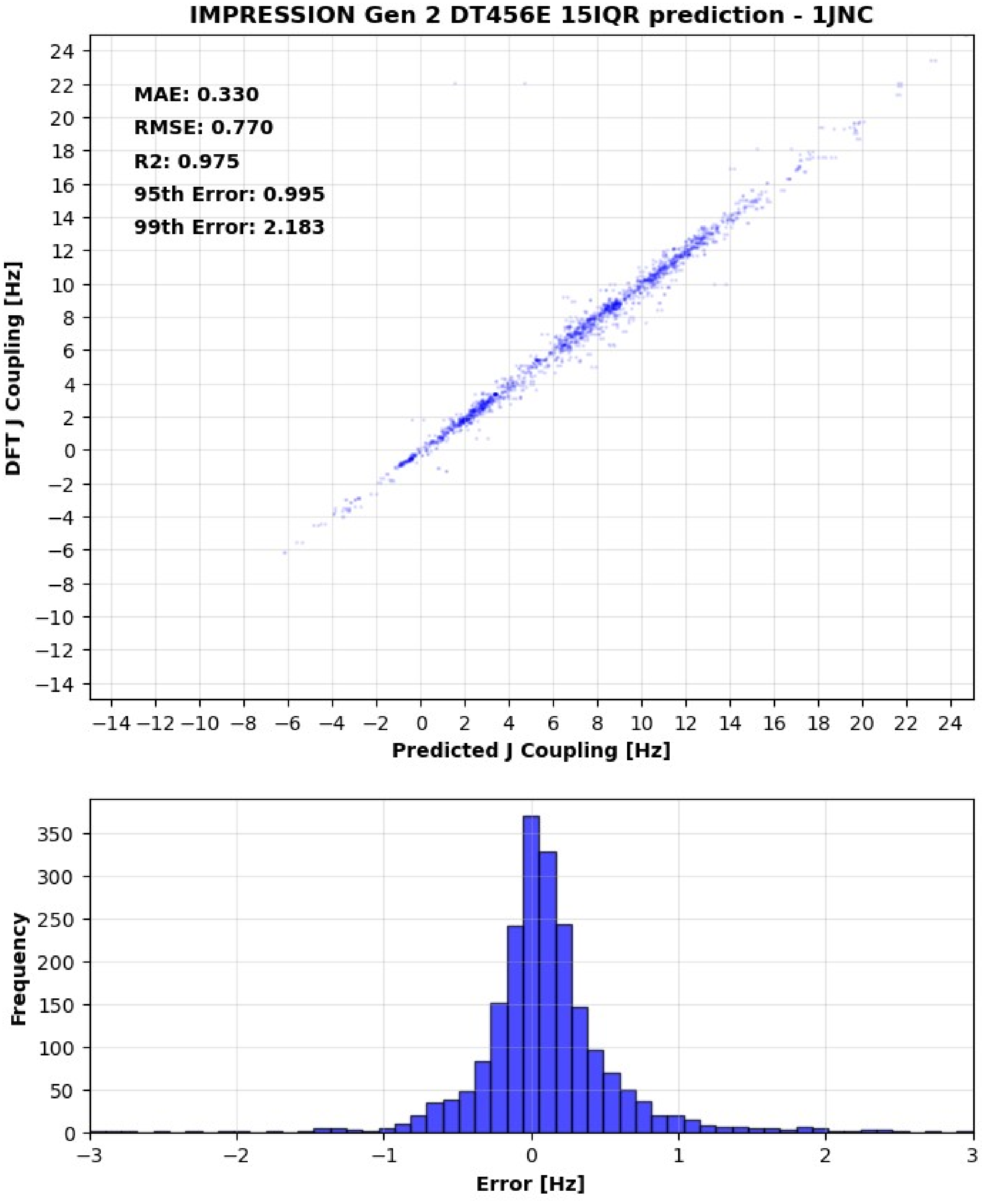
IMPRESSION Gen 2 DT456E 15IQR prediction - 1JNC
MAE: 0.330
RMSE: 0.770
R2: 0.975
95th Error: 0.995
99th Error: 2.183
DFT J Coupling [Hz]
Predicted J Coupling [Hz]
Frequency
Error [Hz]

DFT8K_bg

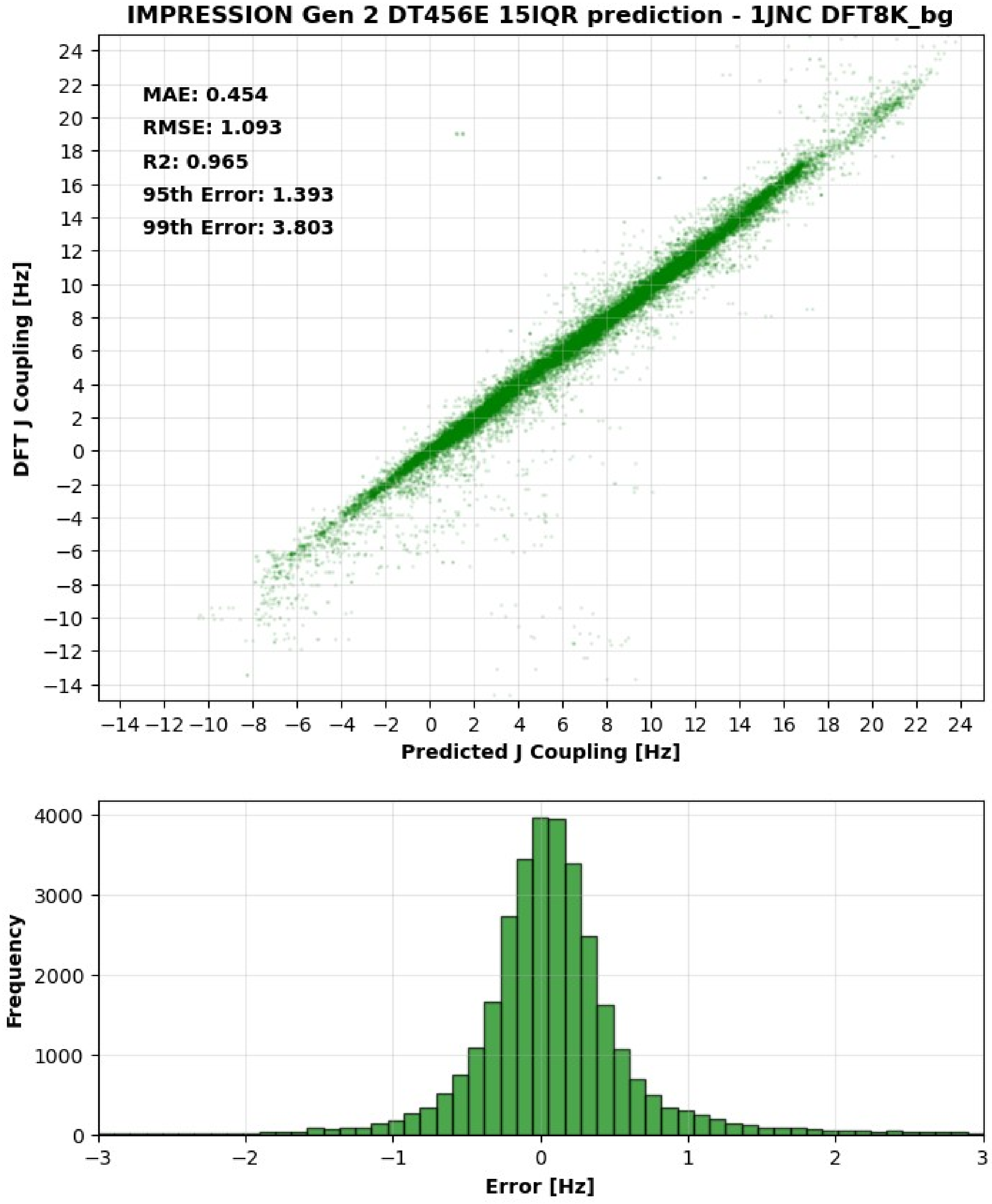

IMPRESSION Gen 2 DT456E 15IQR prediction - 1JNC DFT8K_bg
MAE: 0.454
RMSE: 1.093
R2: 0.965
95th Error: 1.393
99th Error: 3.803
DFT J Coupling [Hz]
Predicted J Coupling [Hz]
Frequency
Error [Hz]

S2.2.17 $^2J_{NC}$

Holdout

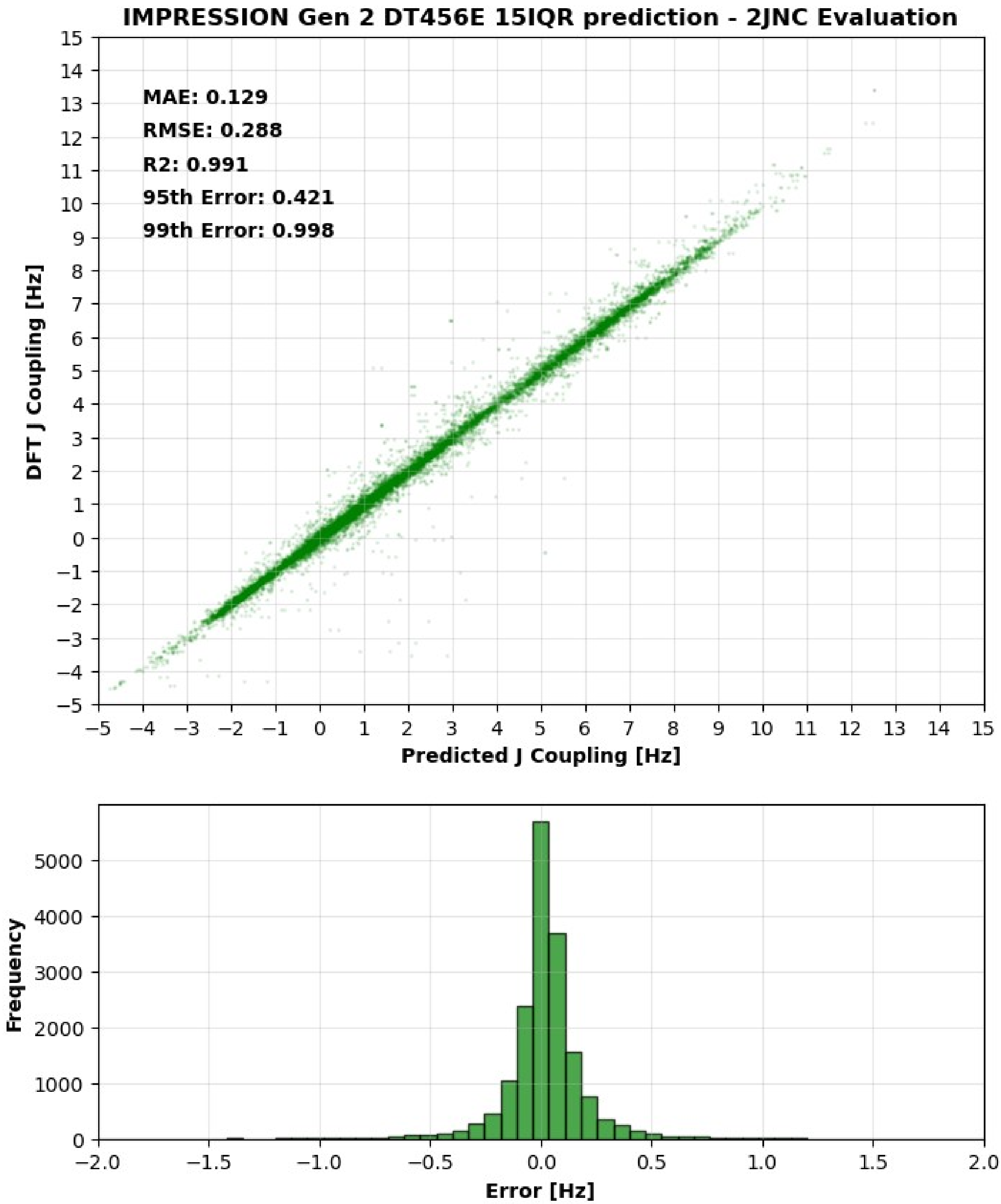

Data3

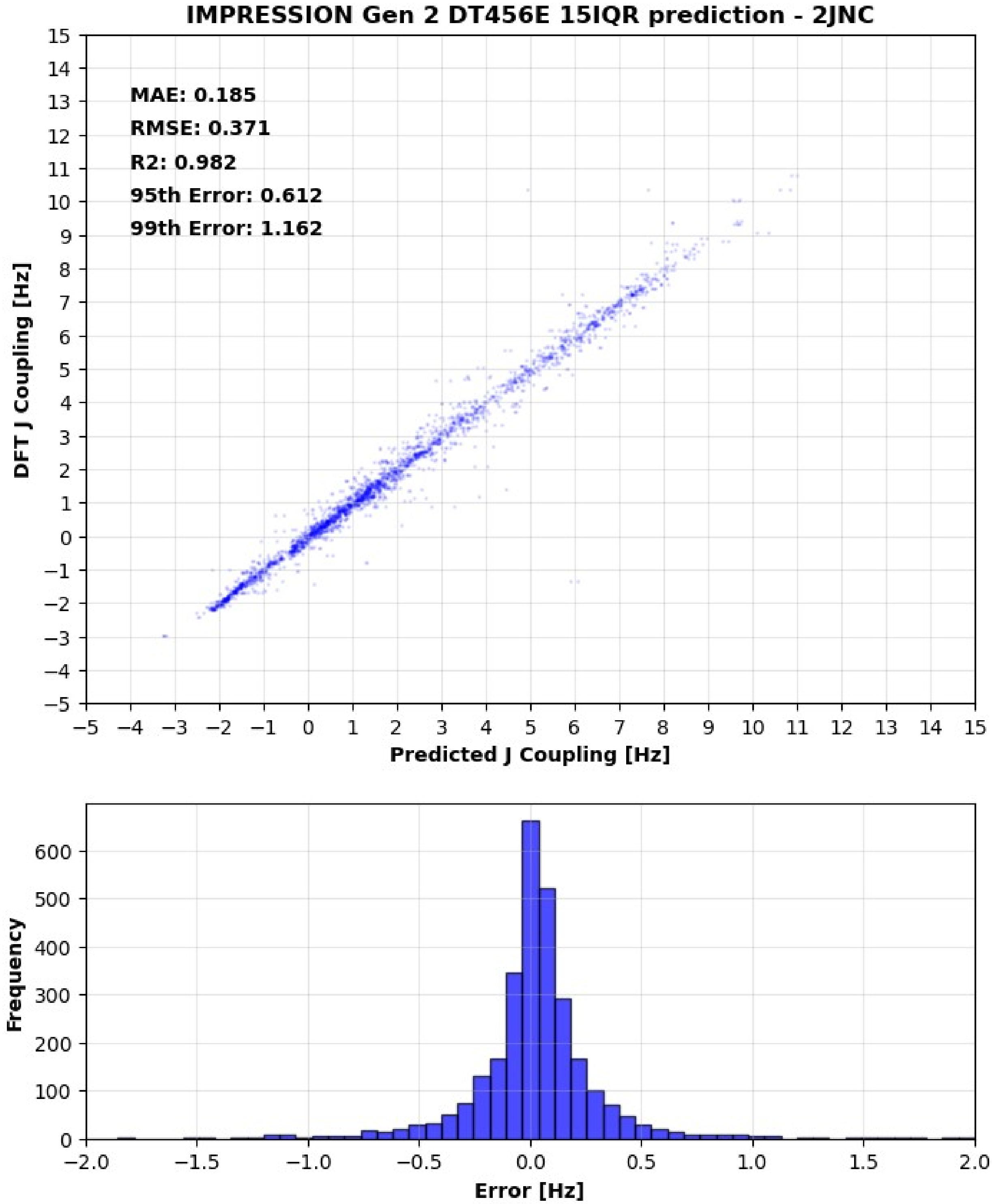
IMPRESSION Gen 2 DT456E 15IQR prediction - 2JNC
MAE: 0.185
RMSE: 0.371
R2: 0.982
95th Error: 0.612
99th Error: 1.162
DFT J Coupling [Hz]
Predicted J Coupling [Hz]
Frequency
Error [Hz]

DFT8K_bg

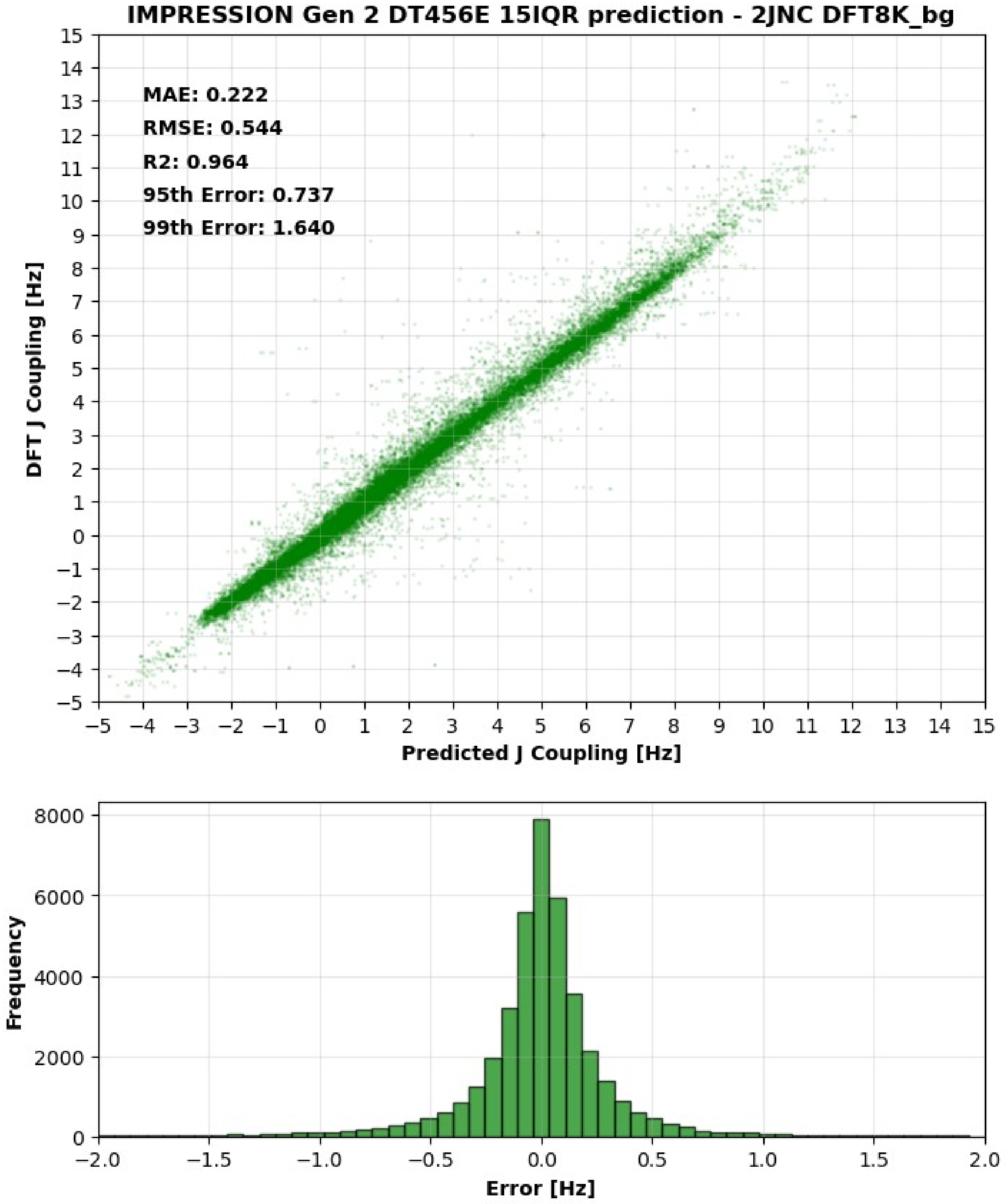

IMPRESSION Gen 2 DT456E 15IQR prediction - 2JNC DFT8K_bg
MAE: 0.222
RMSE: 0.544
R2: 0.964
95th Error: 0.737
99th Error: 1.640
DFT J Coupling [Hz]
Predicted J Coupling [Hz]
Frequency
Error [Hz]

S2.2.18 $^3J_{NC}$

Holdout

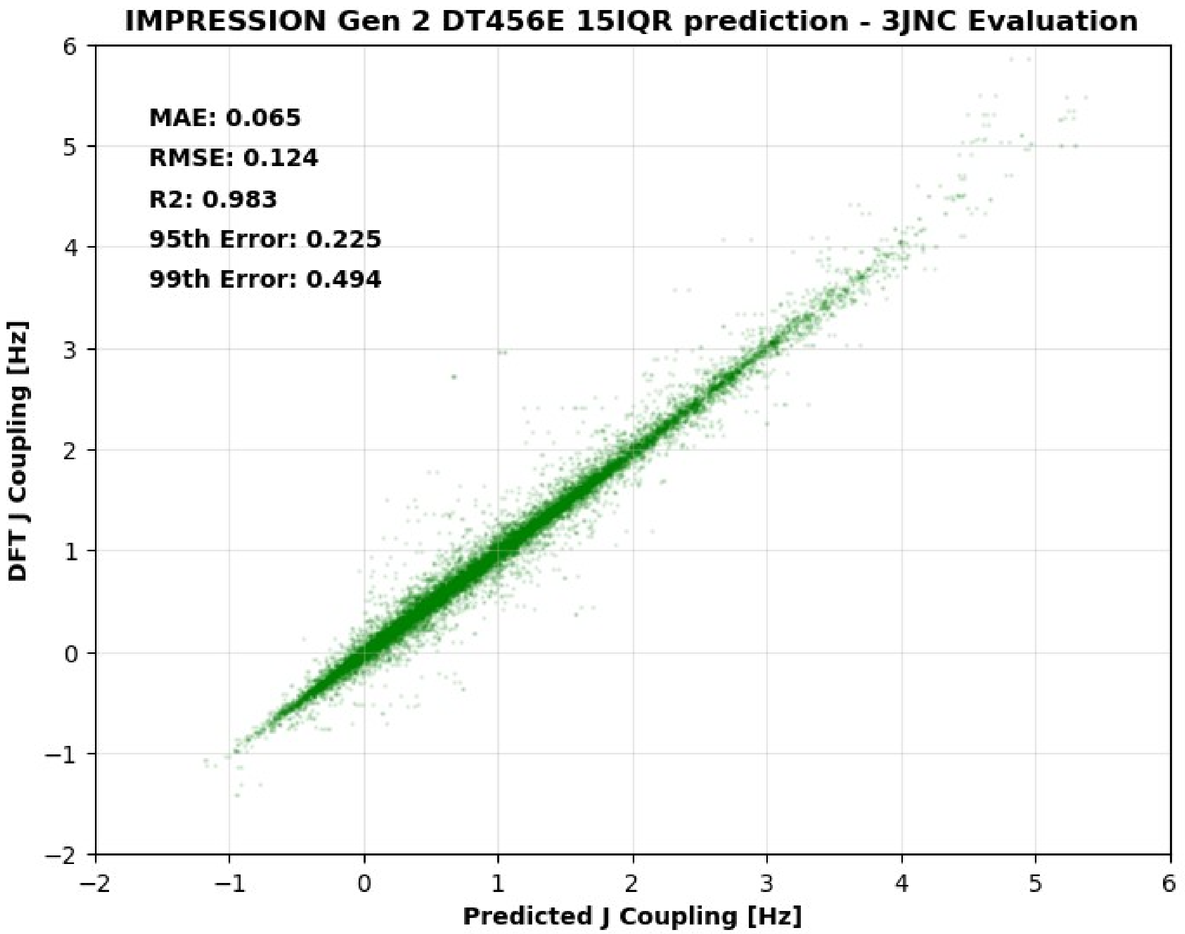

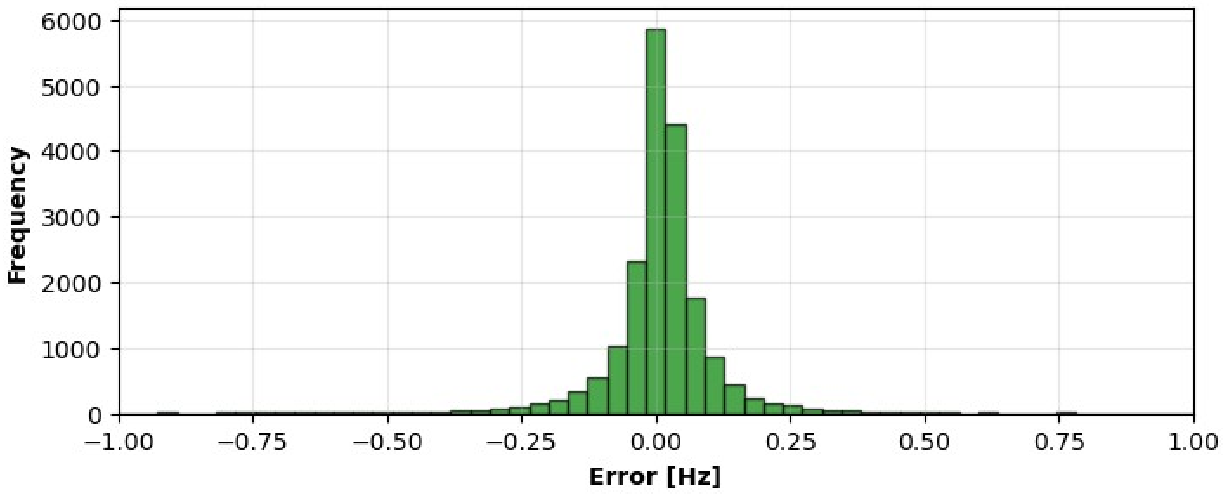

Data3

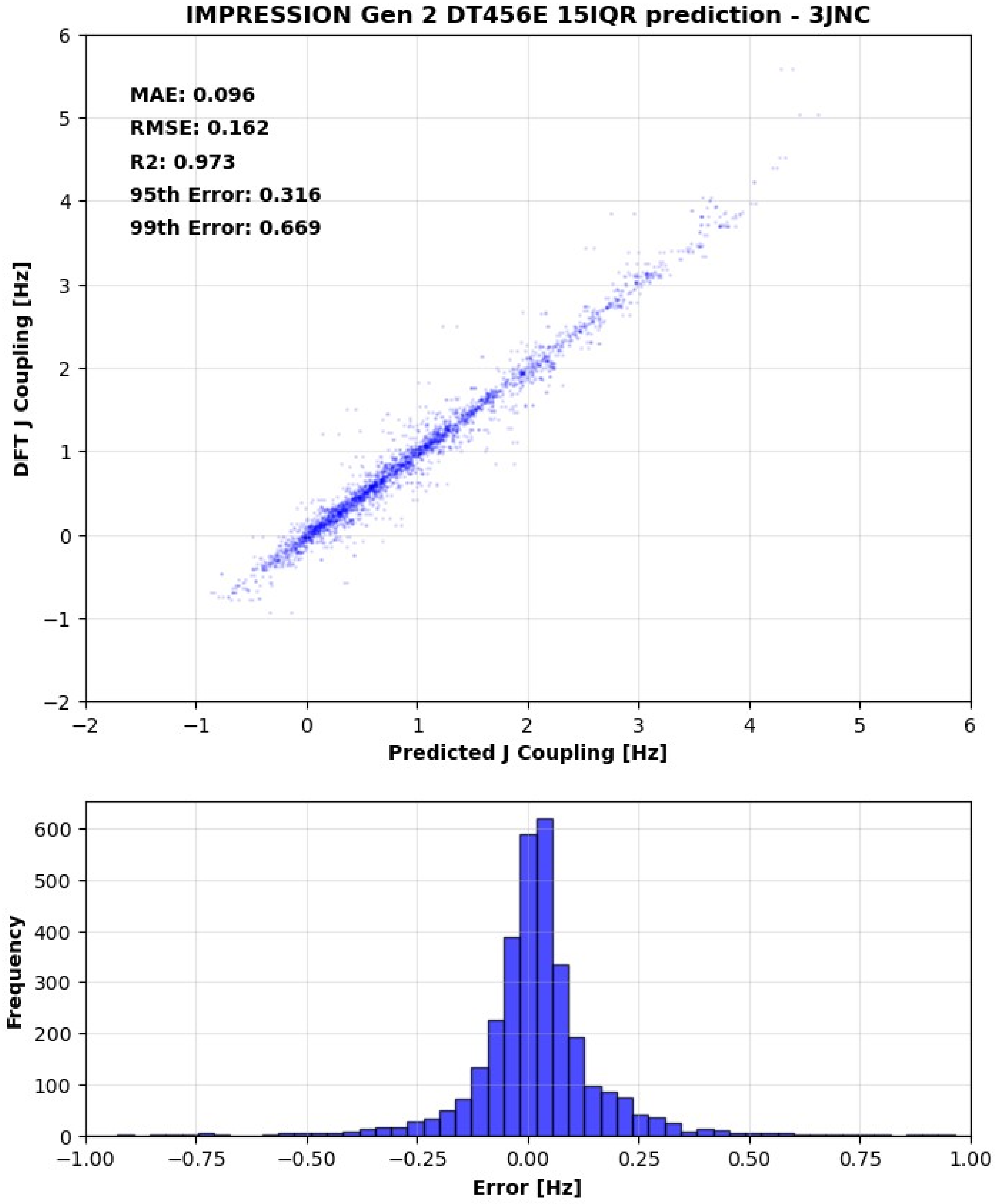
IMPRESSION Gen 2 DT456E 15IQR prediction - 3JNC
MAE: 0.096
RMSE: 0.162
R2: 0.973
95th Error: 0.316
99th Error: 0.669
DFT J Coupling [Hz]
Predicted J Coupling [Hz]
Frequency
Error [Hz]

DFT8K_bg

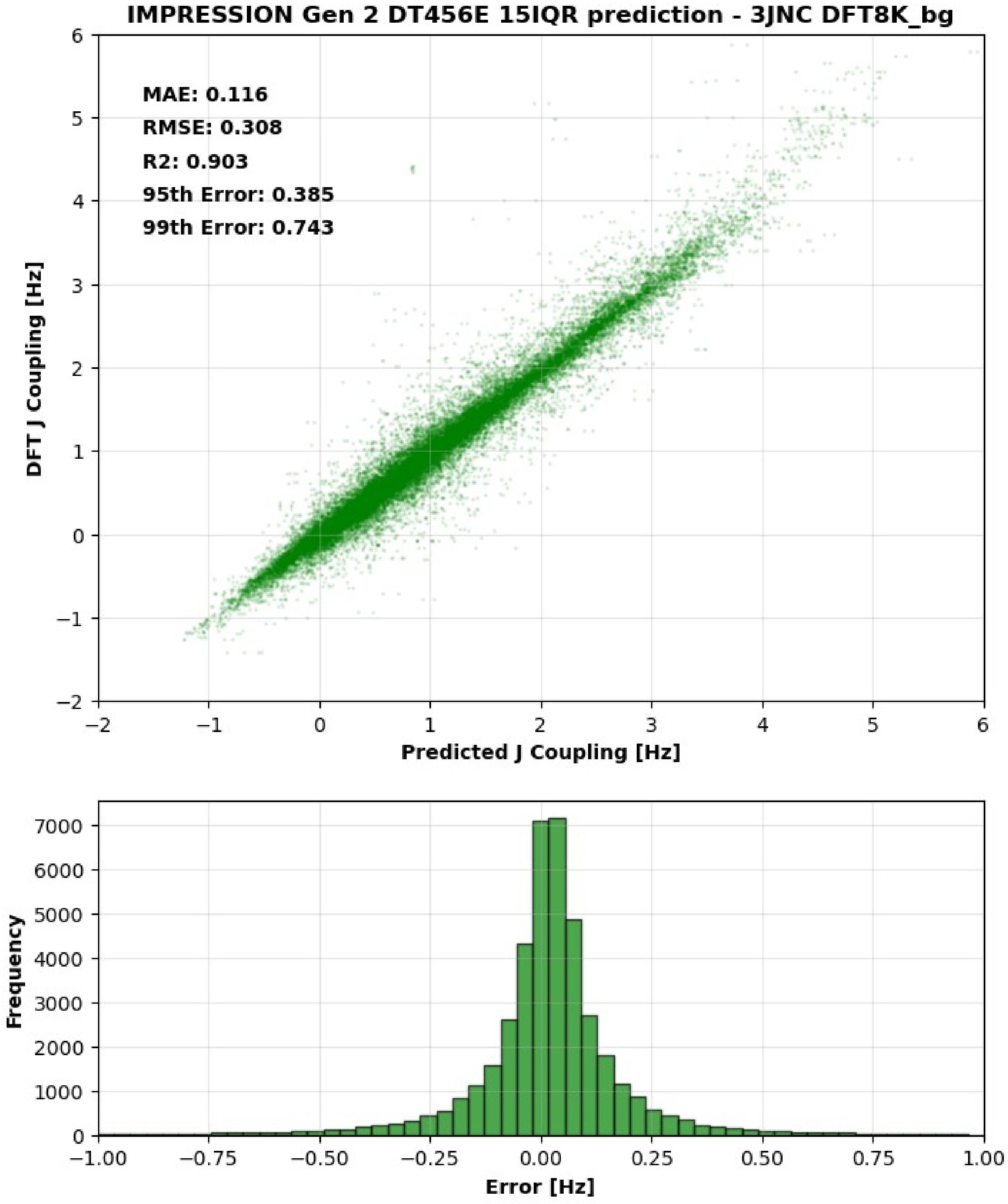
IMPRESSION Gen 2 DT456E 15IQR prediction - 3JNC DFT8K_bg
MAE: 0.116
RMSE: 0.308
R2: 0.903
95th Error: 0.385
99th Error: 0.743
DFT J Coupling [Hz]
Predicted J Coupling [Hz]
Frequency
Error [Hz]

S2.2.19 $^4J_{NC}$

Holdout

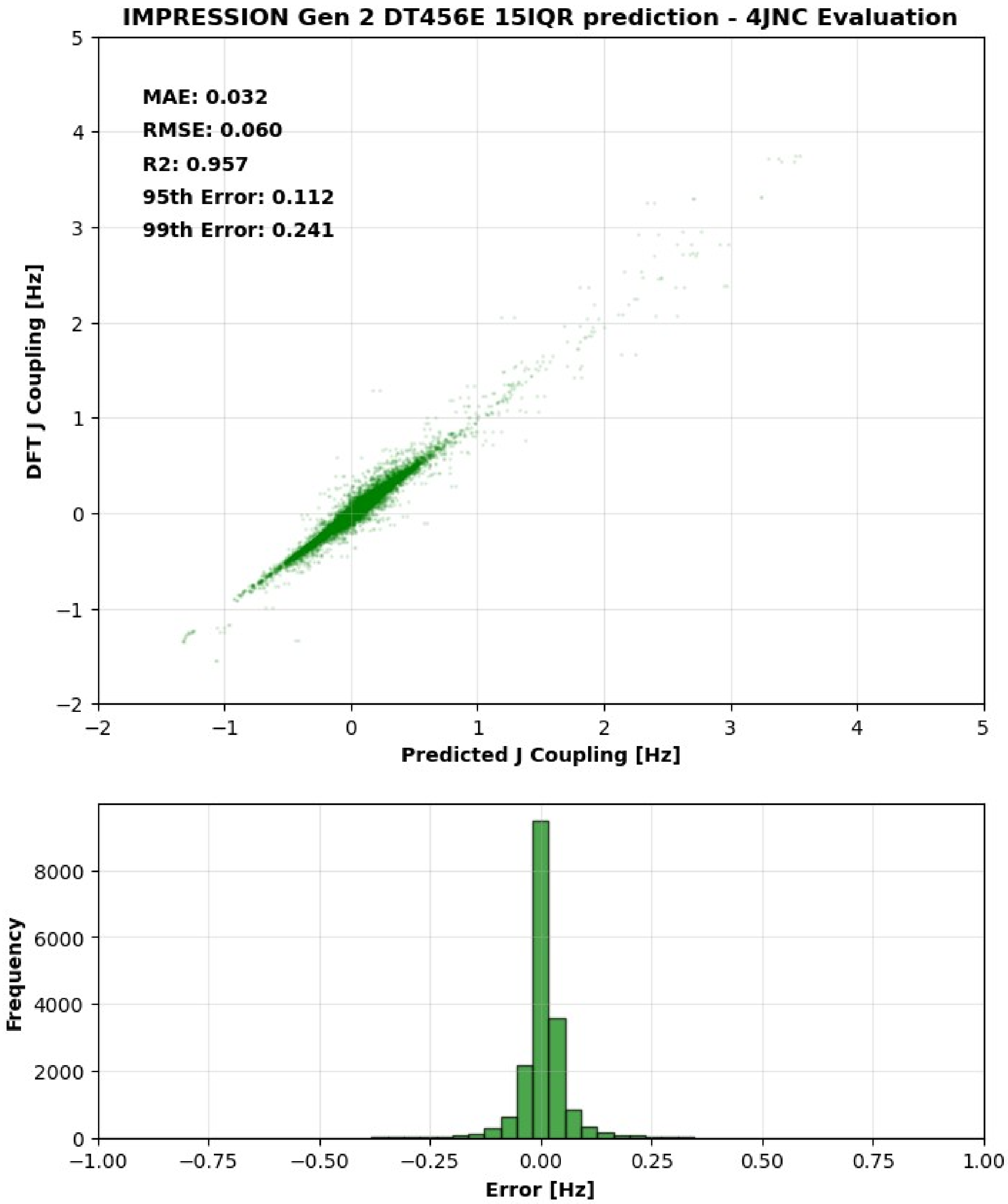

Data3

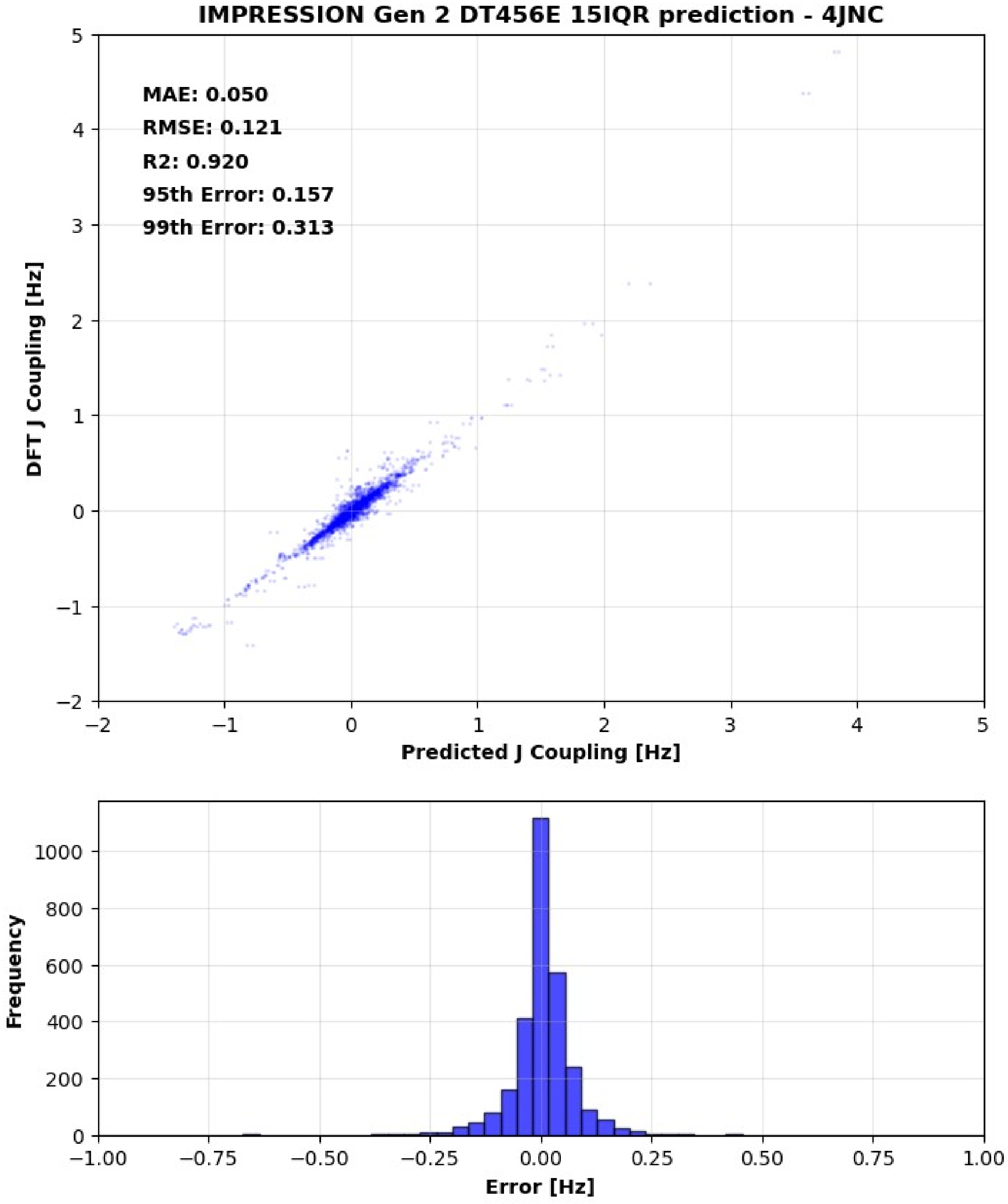
IMPRESSION Gen 2 DT456E 15IQR prediction - 4JNC
MAE: 0.050
RMSE: 0.121
R2: 0.920
95th Error: 0.157
99th Error: 0.313
DFT J Coupling [Hz]
Predicted J Coupling [Hz]
Frequency
Error [Hz]

DFT8K_bg

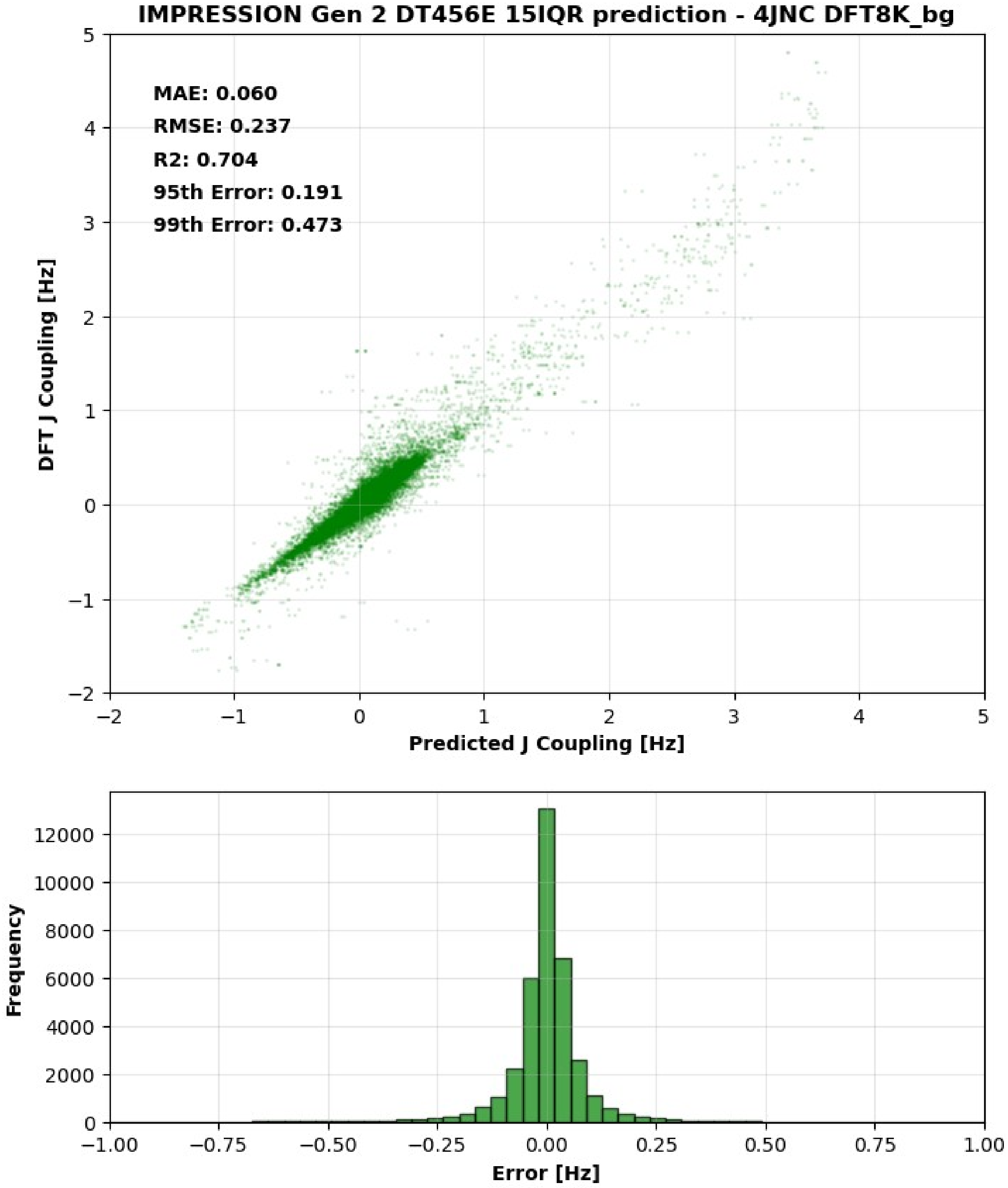

IMPRESSION Gen 2 DT456E 15IQR prediction - 4JNC DFT8K_bg
MAE: 0.060
RMSE: 0.237
R2: 0.704
95th Error: 0.191
99th Error: 0.473
DFT J Coupling [Hz]
Predicted J Coupling [Hz]
Frequency
Error [Hz]

S2.2.24 $^2J_{FH}$

Holdout

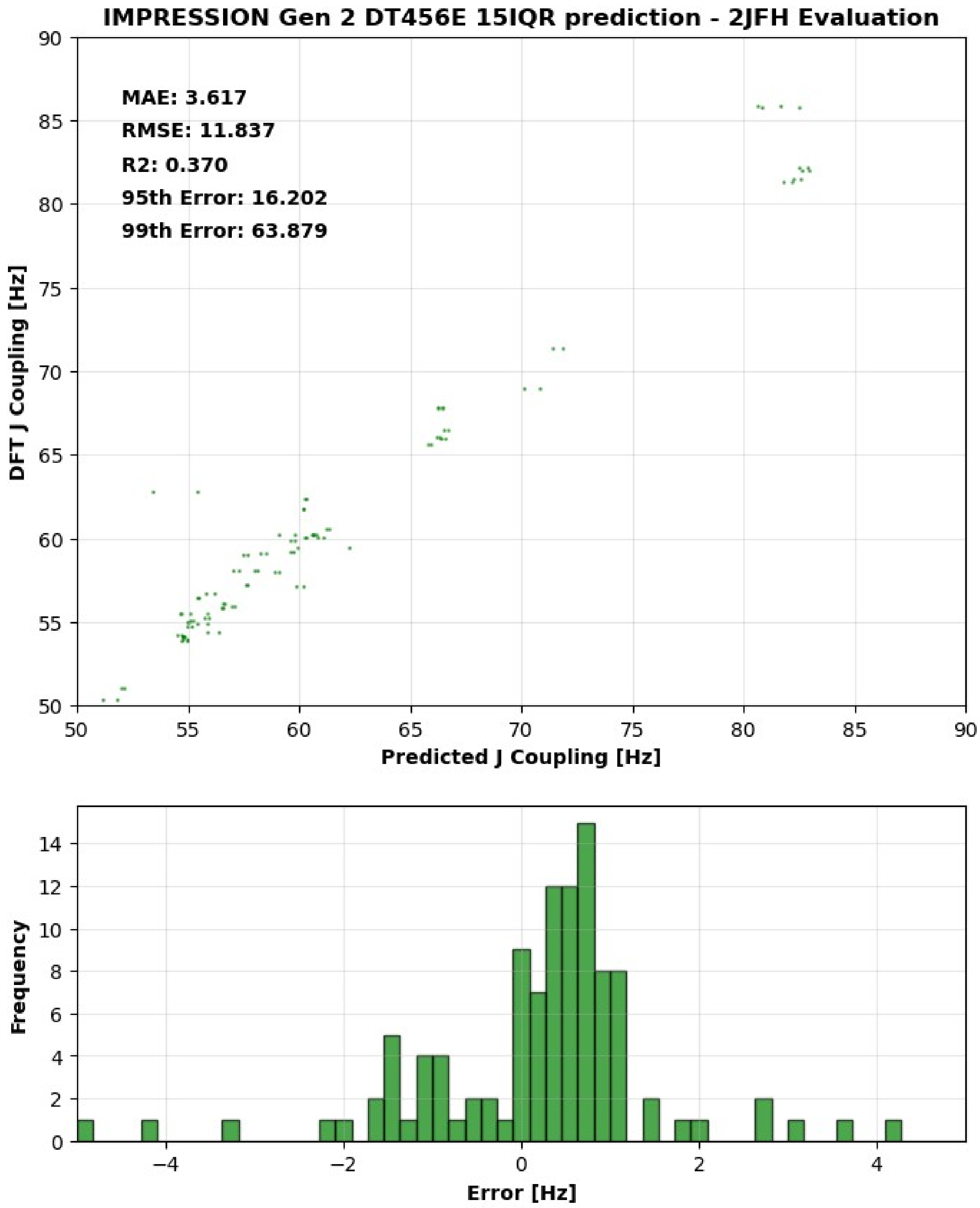

DFT8K_bg

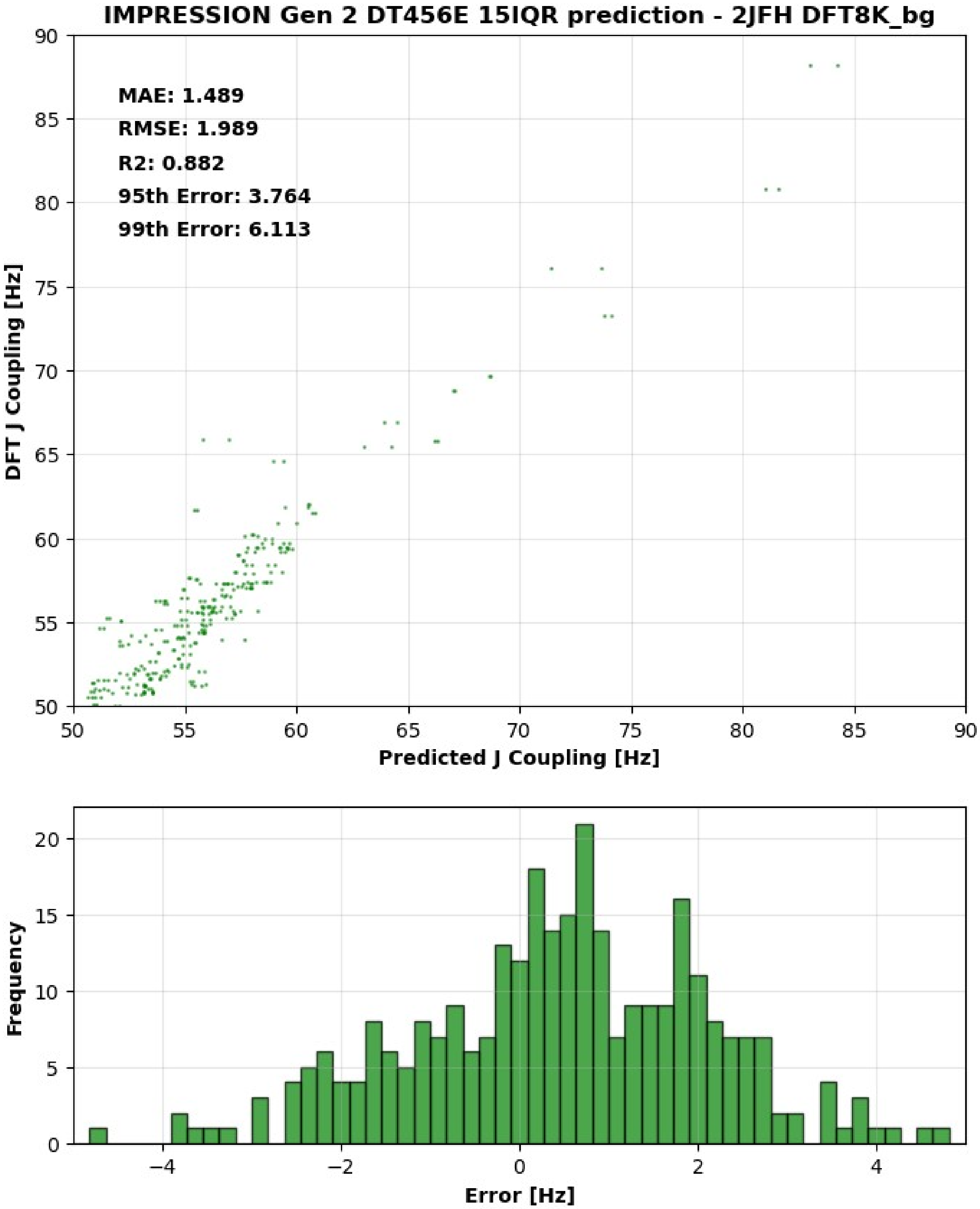

IMPRESSION Gen 2 DT456E 15IQR prediction - 2JFH DFT8K_bg
MAE: 1.489
RMSE: 1.989
R2: 0.882
95th Error: 3.764
99th Error: 6.113
DFT J Coupling [Hz]
Predicted J Coupling [Hz]
Frequency
Error [Hz]

S2.2.25 $^3J_{FH}$

Holdout

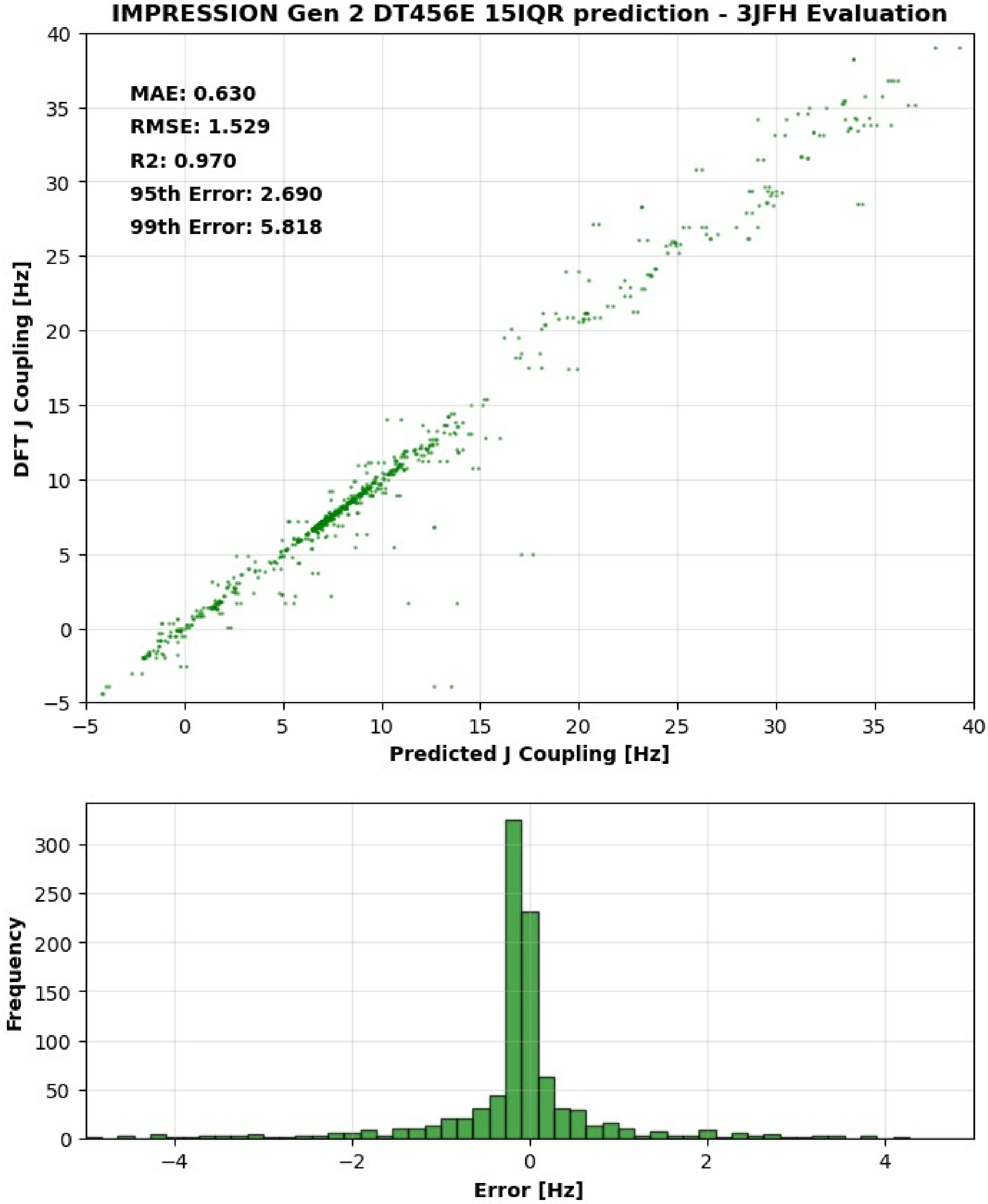

DFT8K_bg

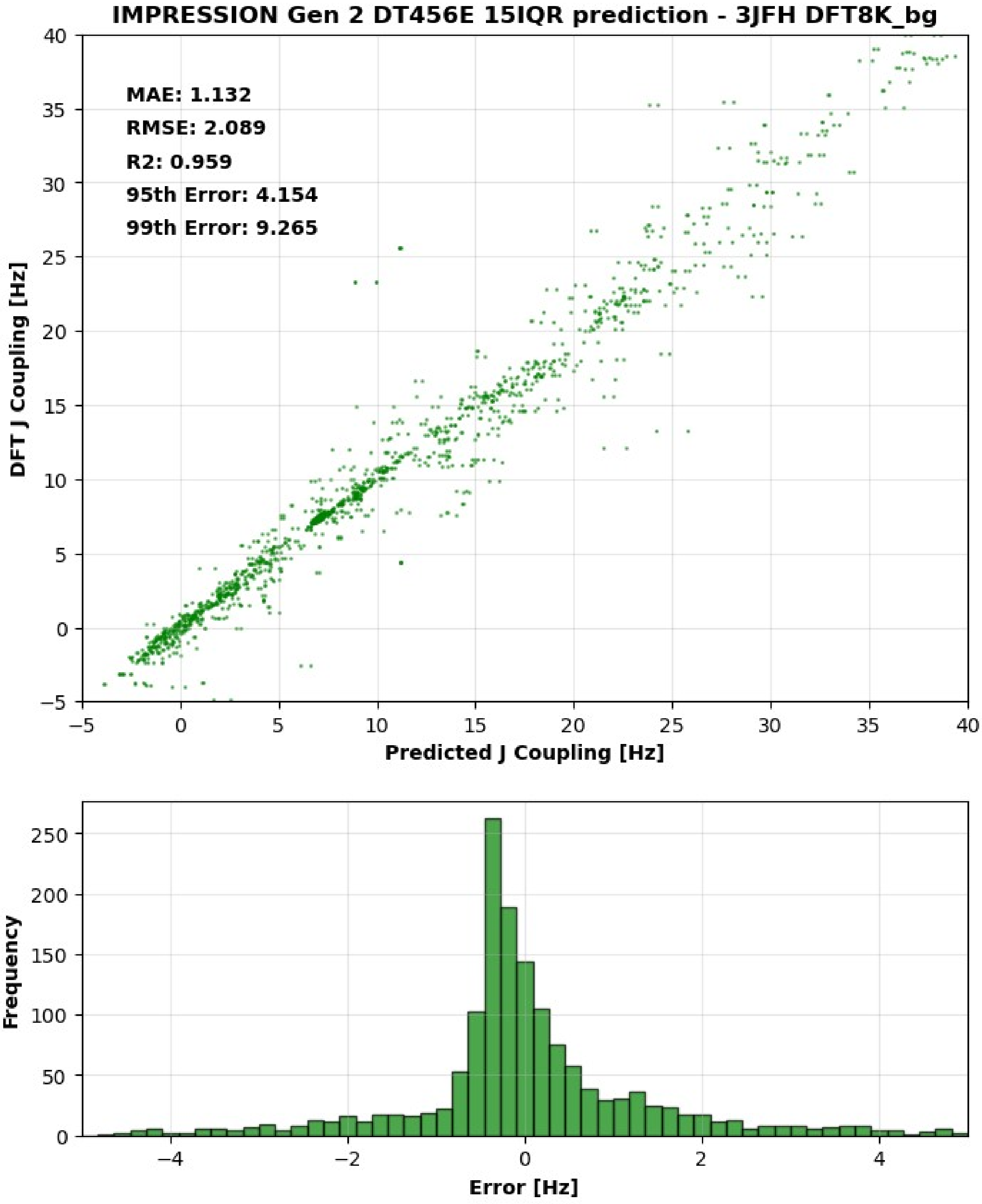

IMPRESSION Gen 2 DT456E 15IQR prediction - 3JFH DFT8K_bg
MAE: 1.132
RMSE: 2.089
R2: 0.959
95th Error: 4.154
99th Error: 9.265
DFT J Coupling [Hz]
Predicted J Coupling [Hz]
Frequency
Error [Hz]

S2.2.26 $^4J_{FH}$

Holdout

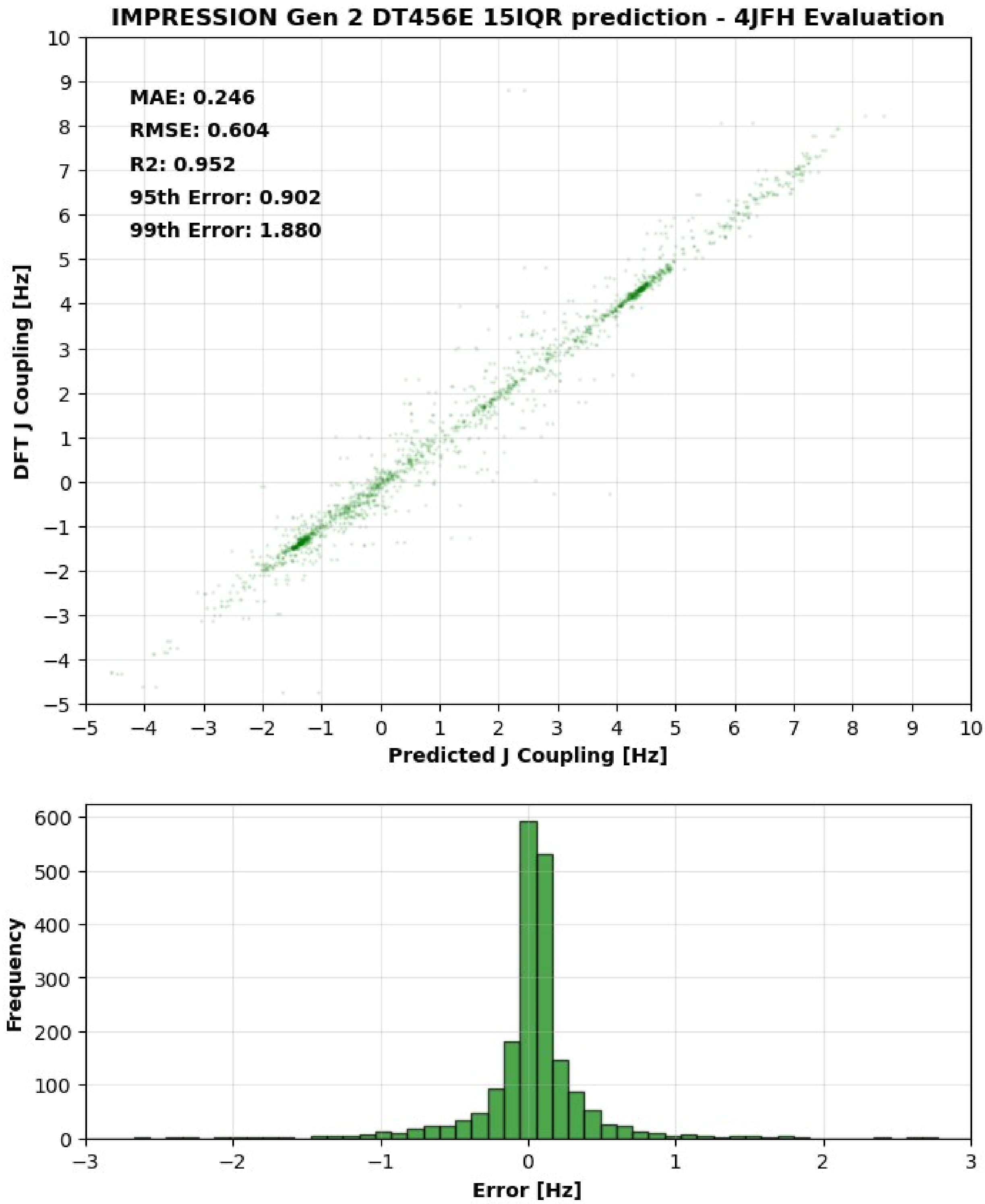

DFT8K_bg

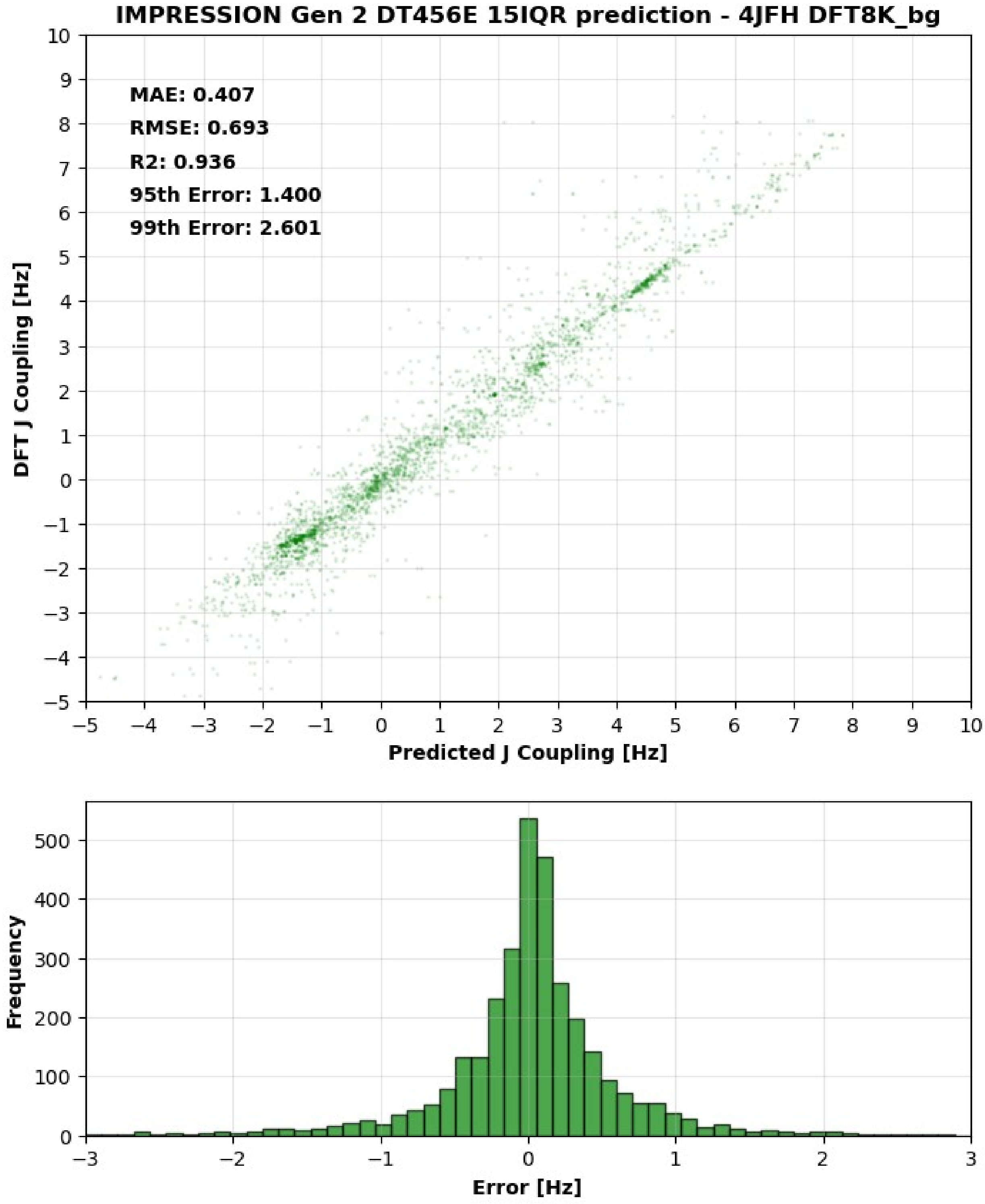
IMPRESSION Gen 2 DT456E 15IQR prediction - 4JFH DFT8K_bg
MAE: 0.407
RMSE: 0.693
R2: 0.936
95th Error: 1.400
99th Error: 2.601
DFT J Coupling [Hz]
Predicted J Coupling [Hz]
Frequency
Error [Hz]

S2.2.27 $^1J_{FC}$

Holdout

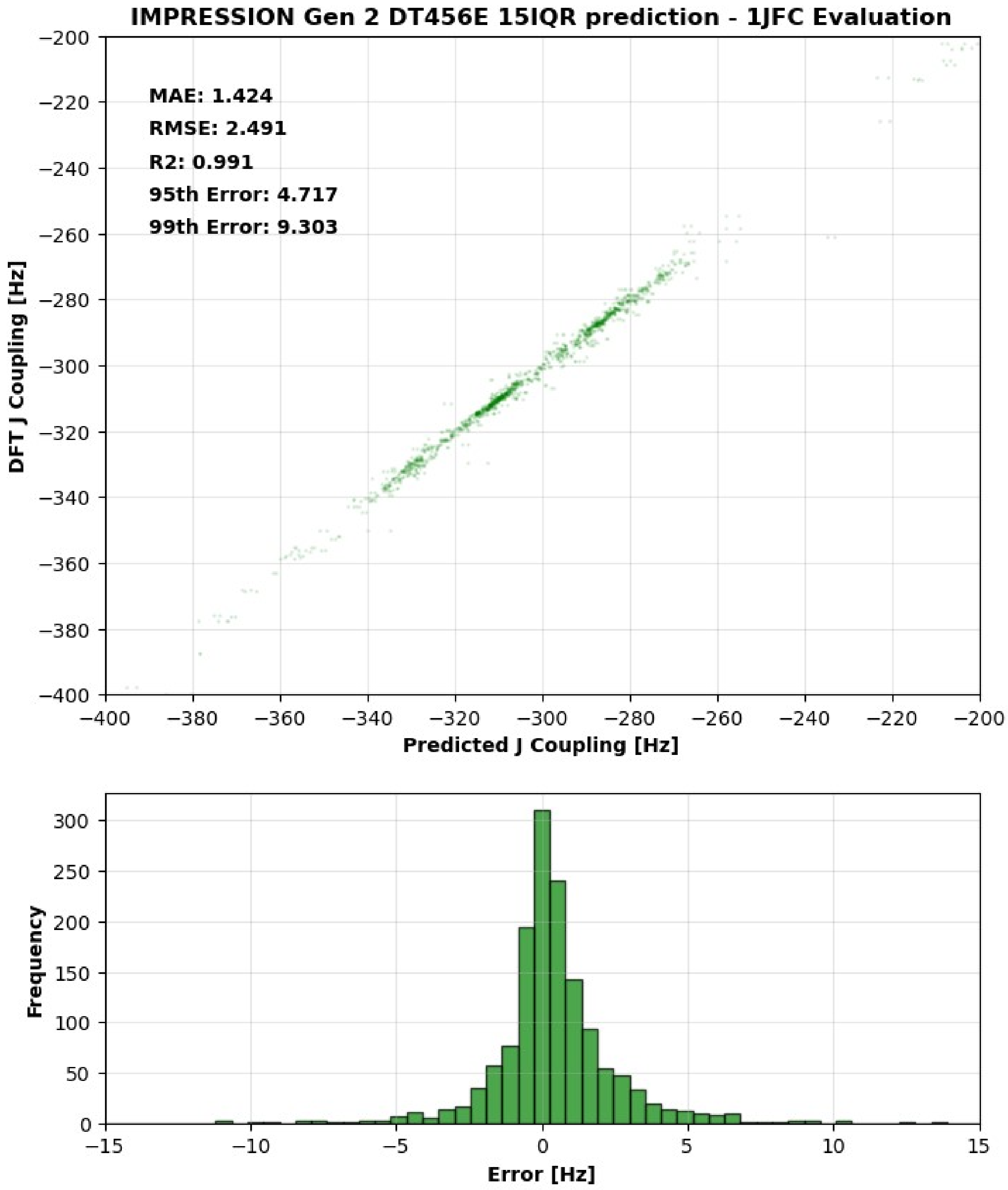

DFT8K_bg

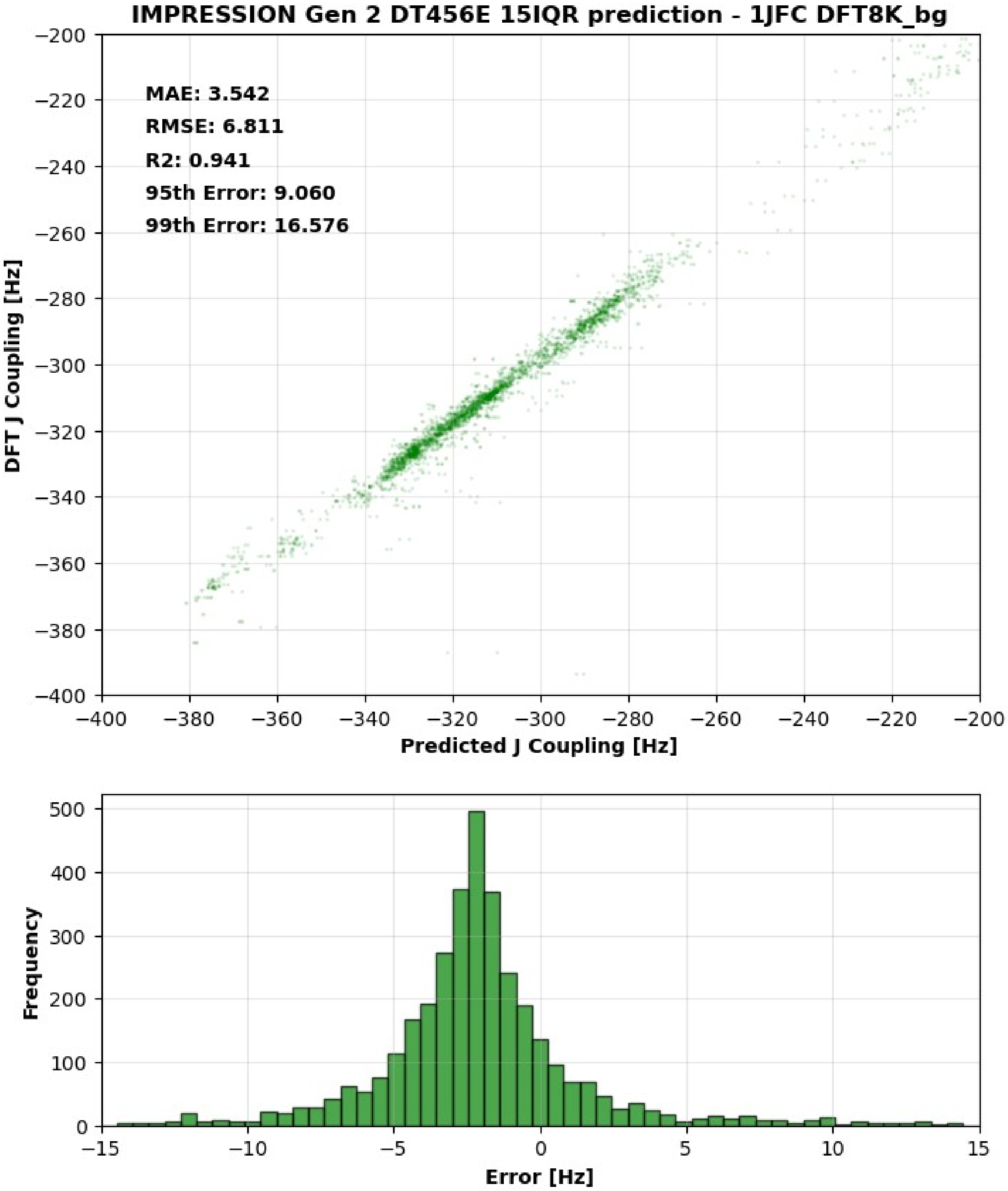
IMPRESSION Gen 2 DT456E 15IQR prediction - 1JFC DFT8K_bg
MAE: 3.542
RMSE: 6.811
R2: 0.941
95th Error: 9.060
99th Error: 16.576
DFT J Coupling [Hz]
Predicted J Coupling [Hz]
Frequency
Error [Hz]

S2.2.28 $^2J_{FC}$

Holdout

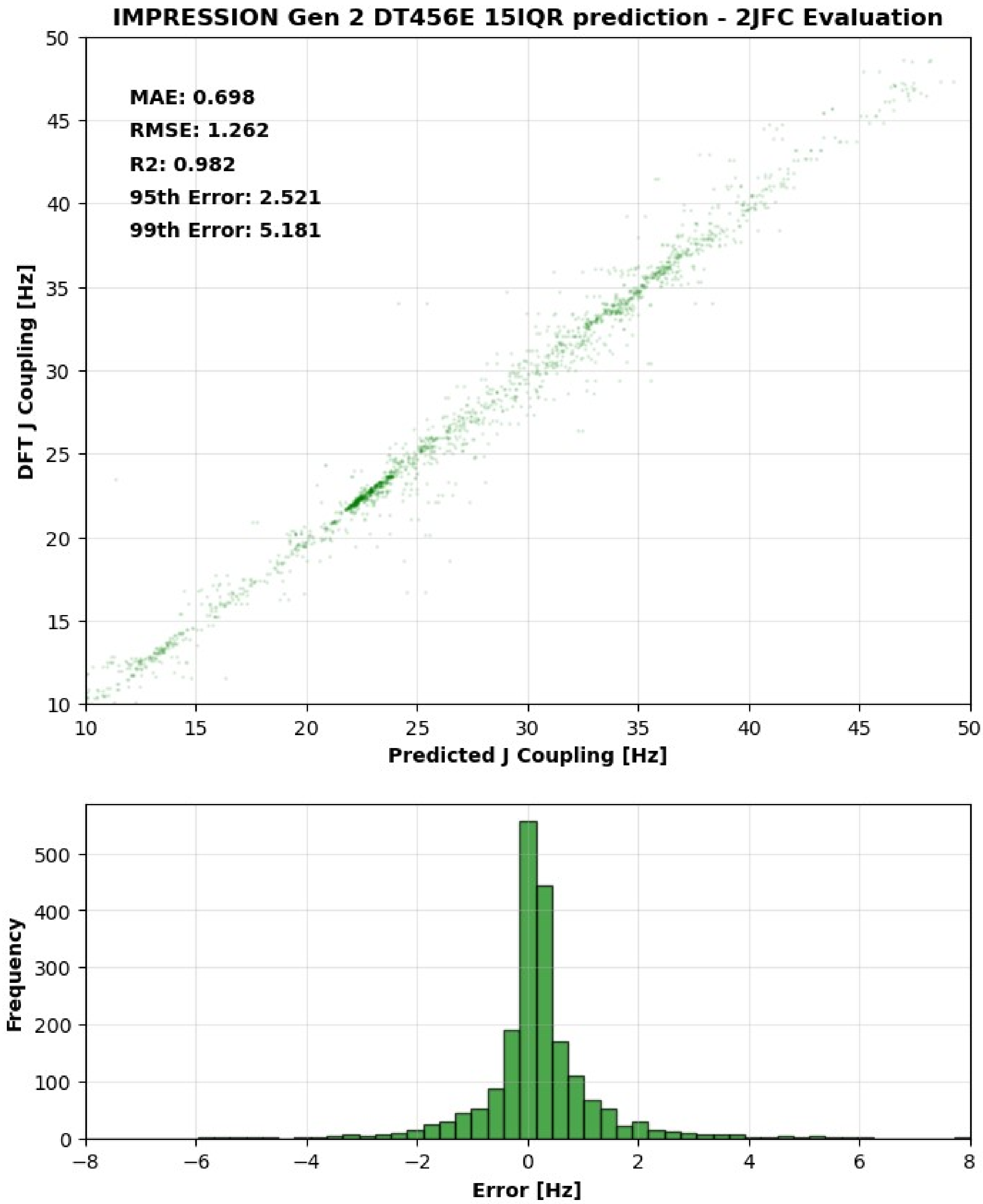

DFT8K_bg

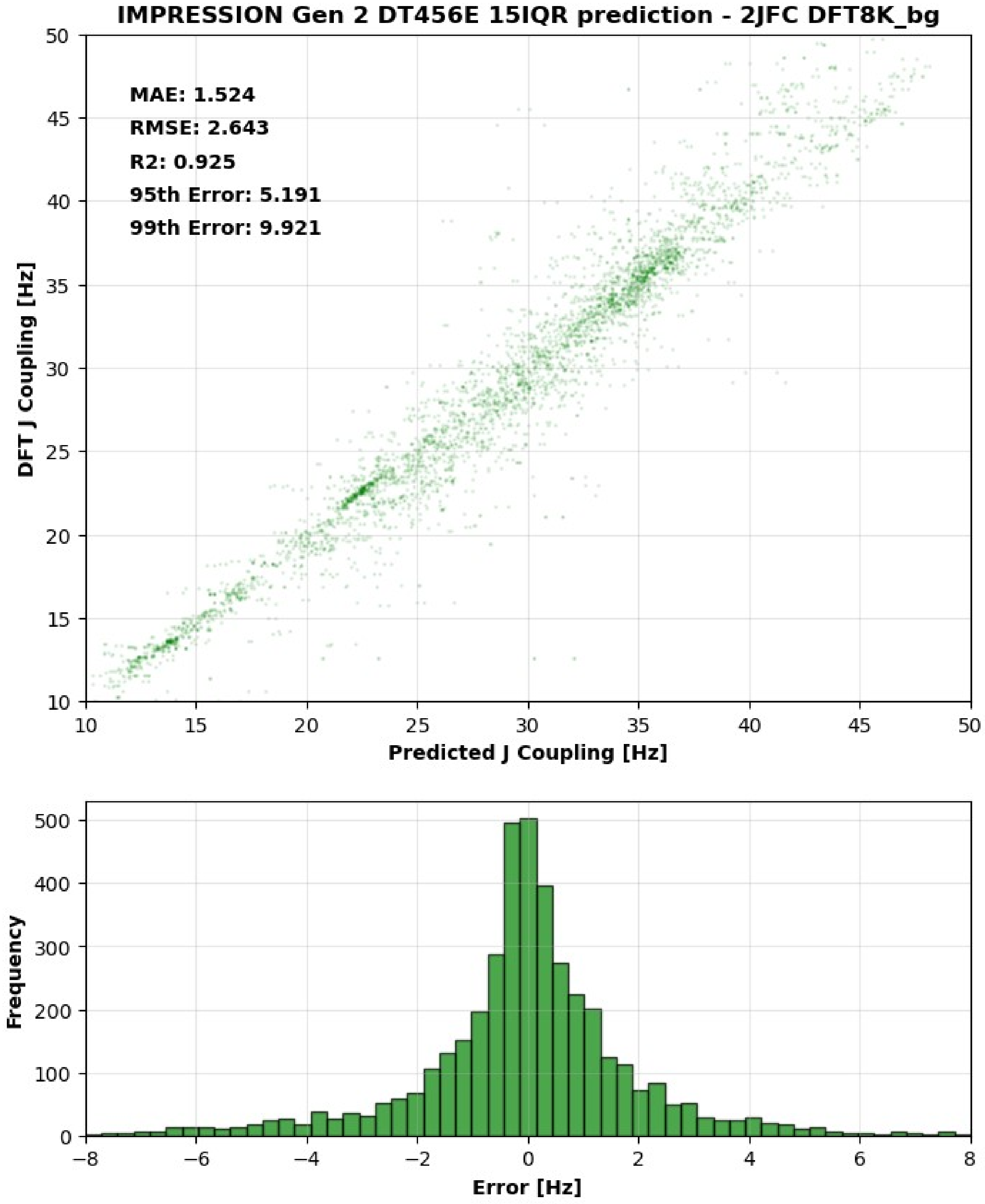
IMPRESSION Gen 2 DT456E 15IQR prediction - 2JFC DFT8K_bg
MAE: 1.524
RMSE: 2.643
R2: 0.925
95th Error: 5.191
99th Error: 9.921
DFT J Coupling [Hz]
Predicted J Coupling [Hz]
Frequency
Error [Hz]

S2.2.29 $^3J_{FC}$

Holdout

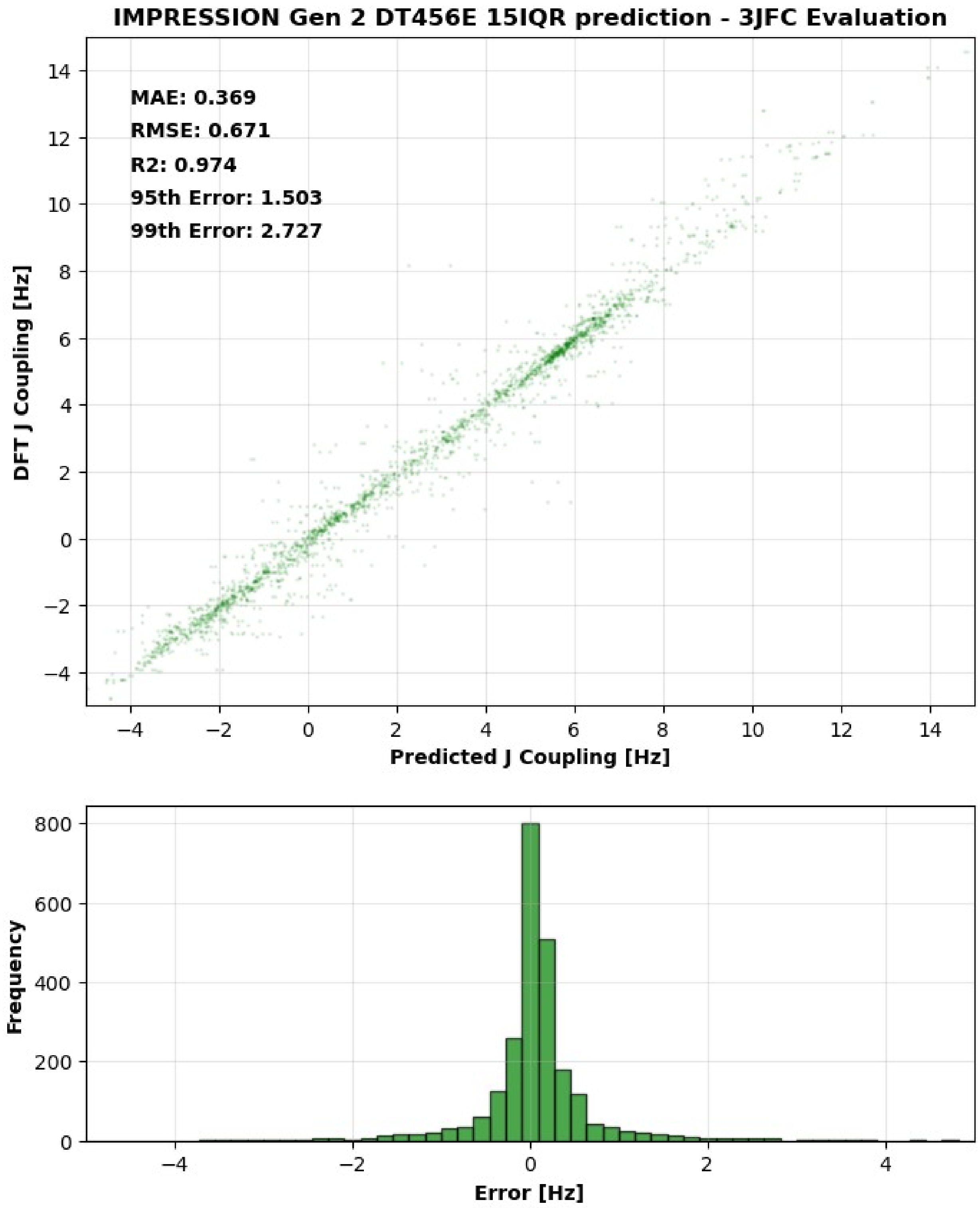

DFT8K_bg

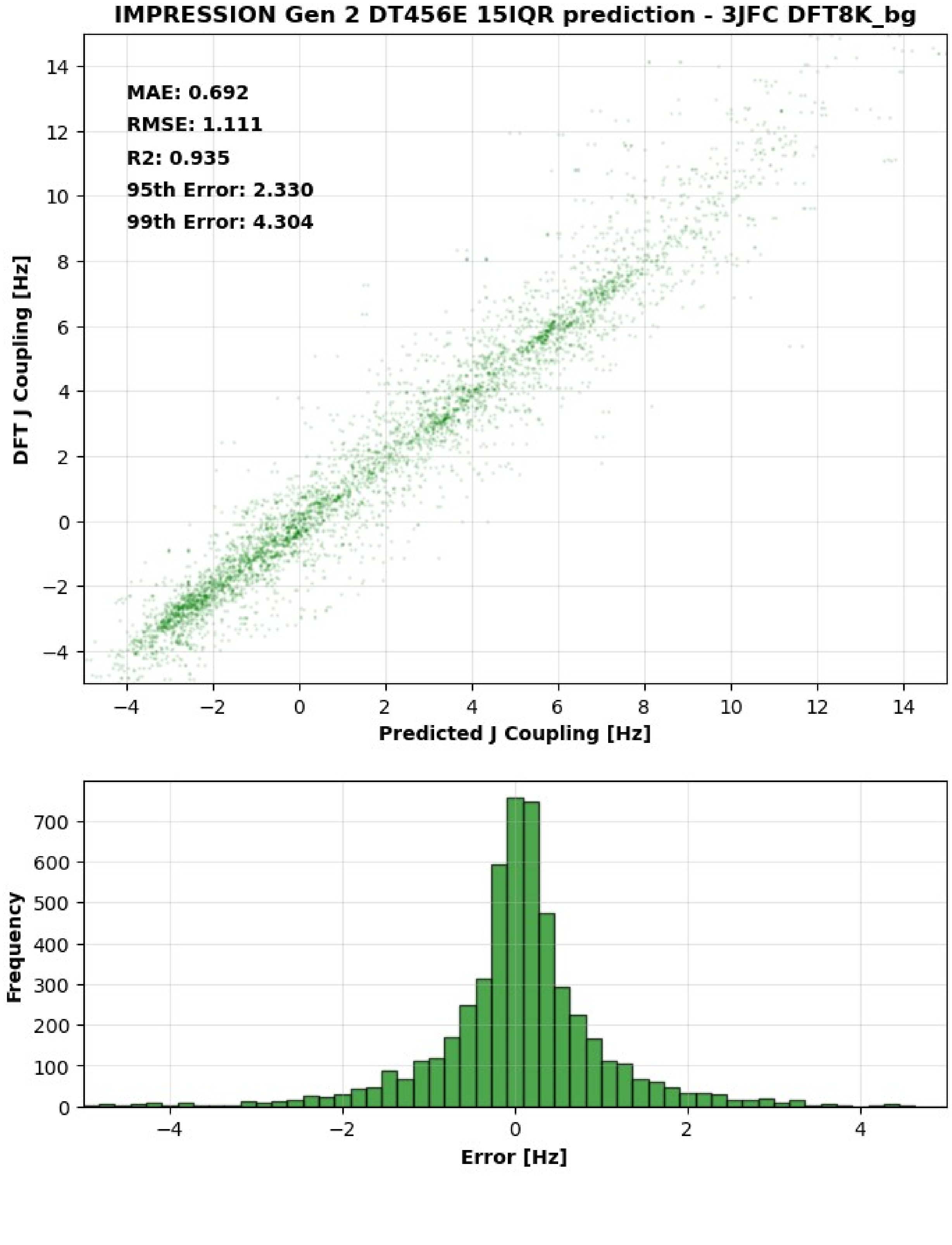

IMPRESSION Gen 2 DT456E 15IQR prediction - 3JFC DFT8K_bg
MAE: 0.692
RMSE: 1.111
R2: 0.935
95th Error: 2.330
99th Error: 4.304
DFT J Coupling [Hz]
Predicted J Coupling [Hz]
Frequency
Error [Hz]

S2.2.30 $^4J_{FC}$

Holdout

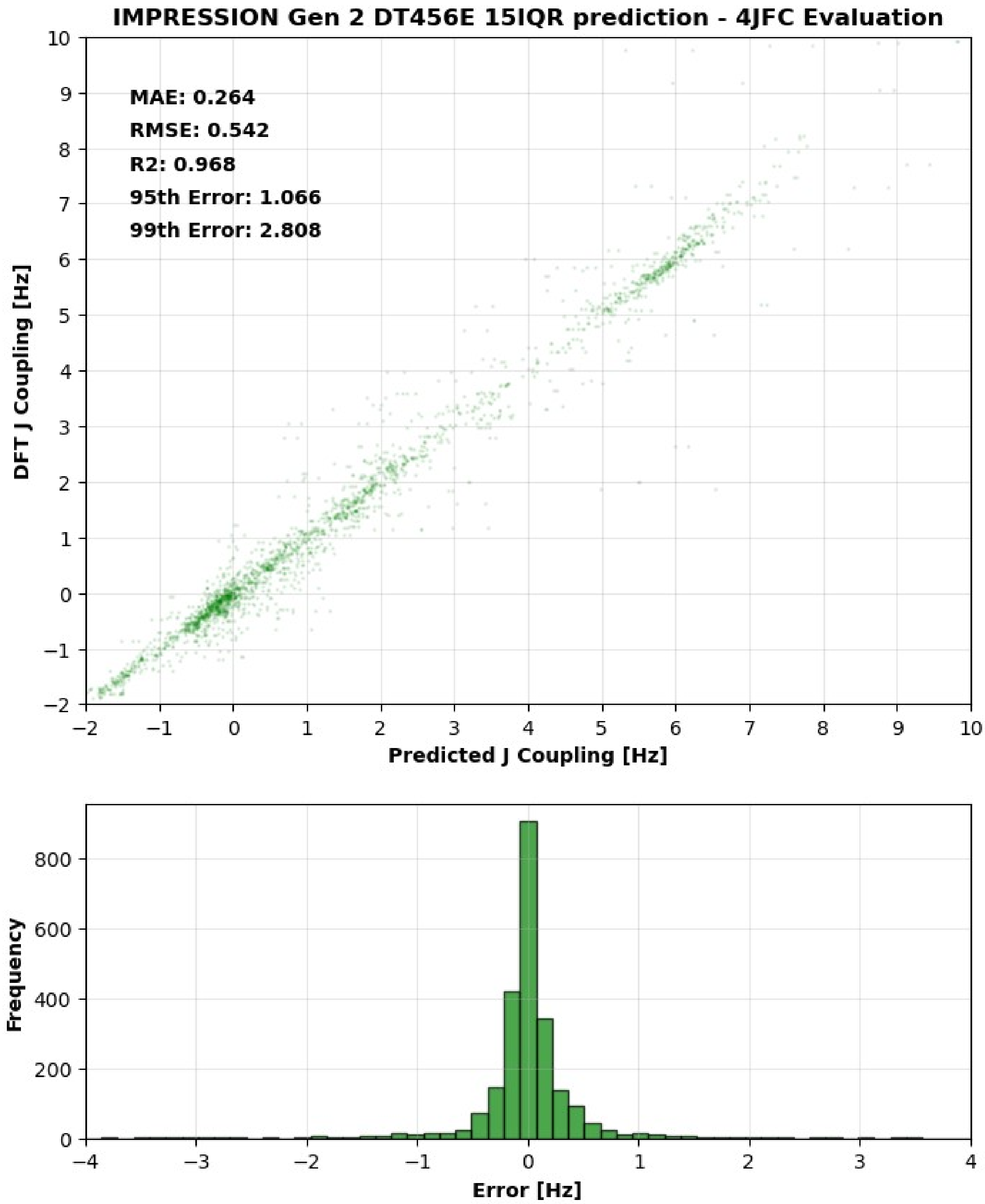

DFT8K_bg

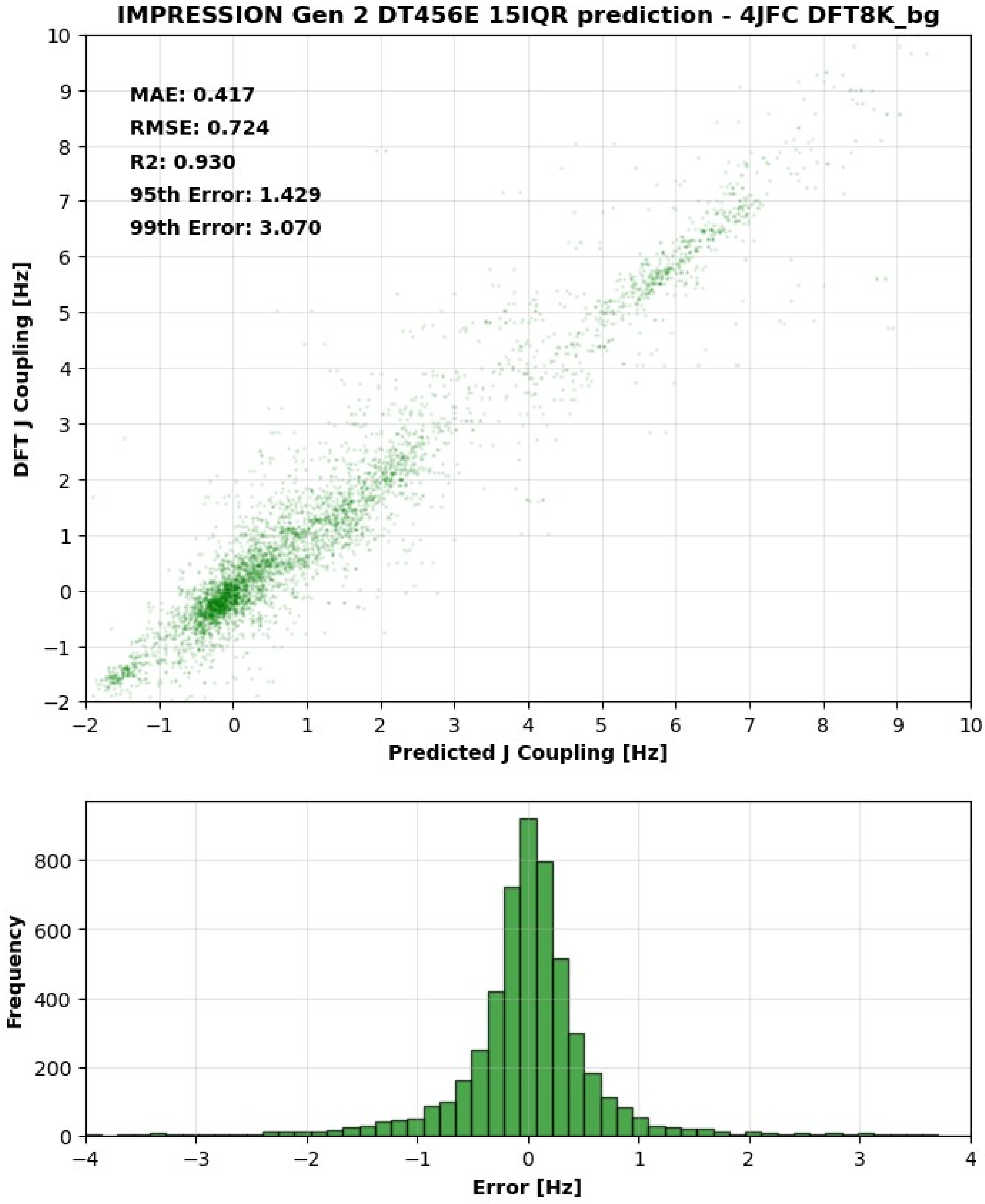

IMPRESSION Gen 2 DT456E 15IQR prediction - 4JFC DFT8K_bg
MAE: 0.417
RMSE: 0.724
R2: 0.930
95th Error: 1.429
99th Error: 3.070
DFT J Coupling [Hz]
Predicted J Coupling [Hz]
Frequency
Error [Hz]

S2.2.31 $^2J_{FN}$

Holdout

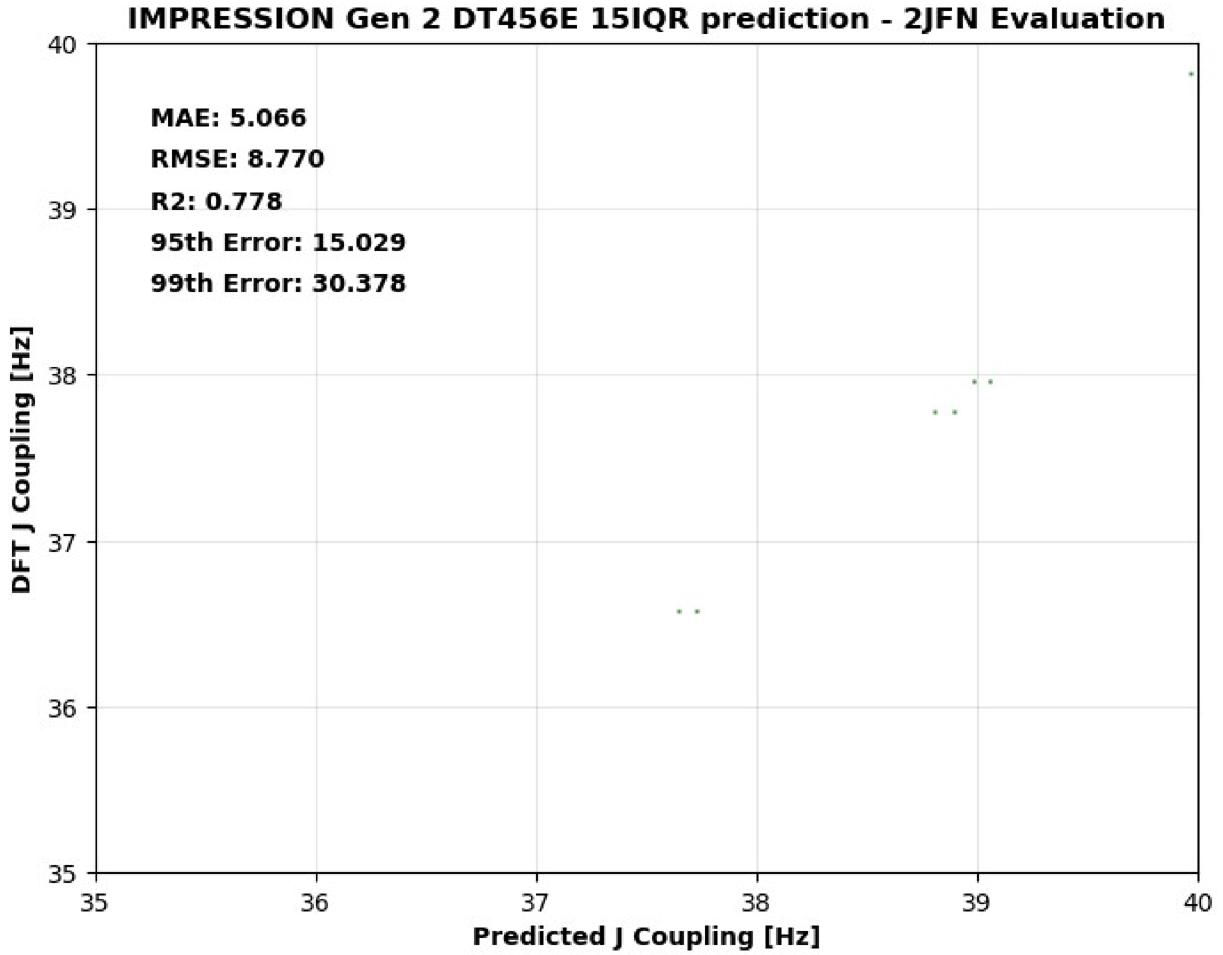

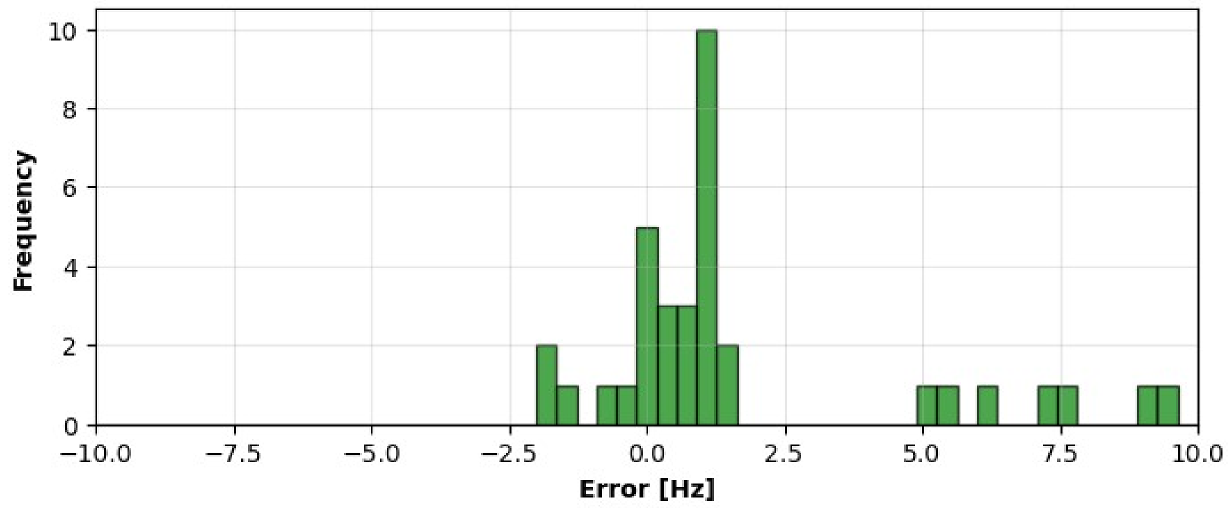

DFT8K_bg

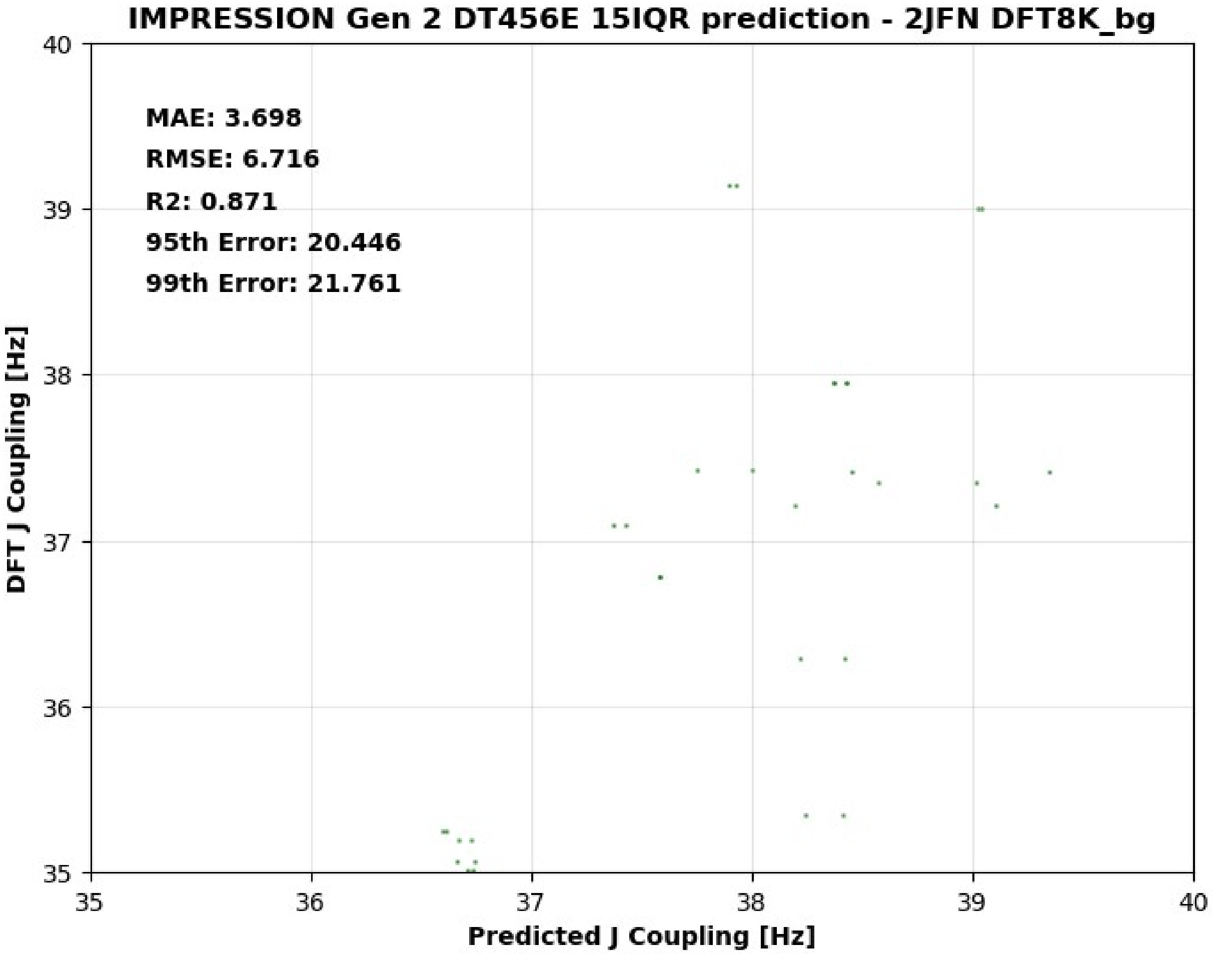

IMPRESSION Gen 2 DT456E 15IQR prediction - 2JFN DFT8K_bg
MAE: 3.698
RMSE: 6.716
R2: 0.871
95th Error: 20.446
99th Error: 21.761
DFT J Coupling [Hz]
Predicted J Coupling [Hz]
35
36
37
38
39
40

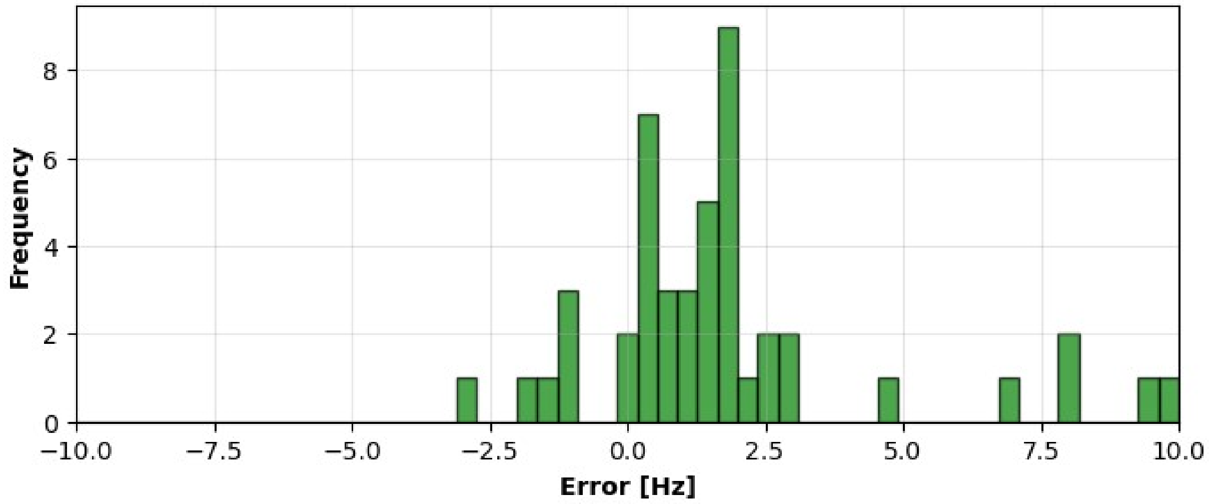

Frequency
Error [Hz]
−10.0
−7.5
−5.0
−2.5
0.0
2.5
5.0
7.5
10.0
0
2
4
6
8

S2.2.32 $^3J_{FN}$

Holdout

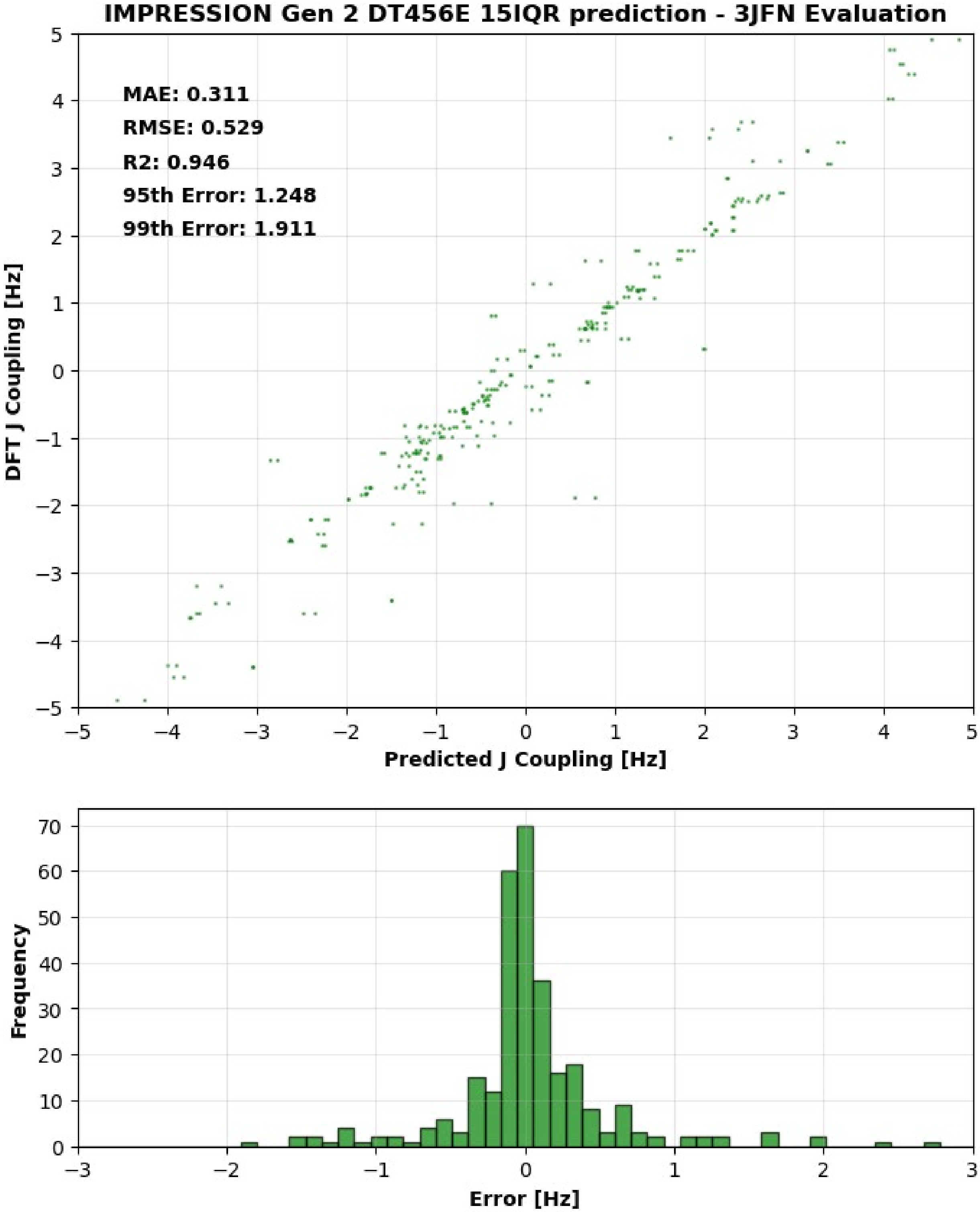

DFT8K_bg

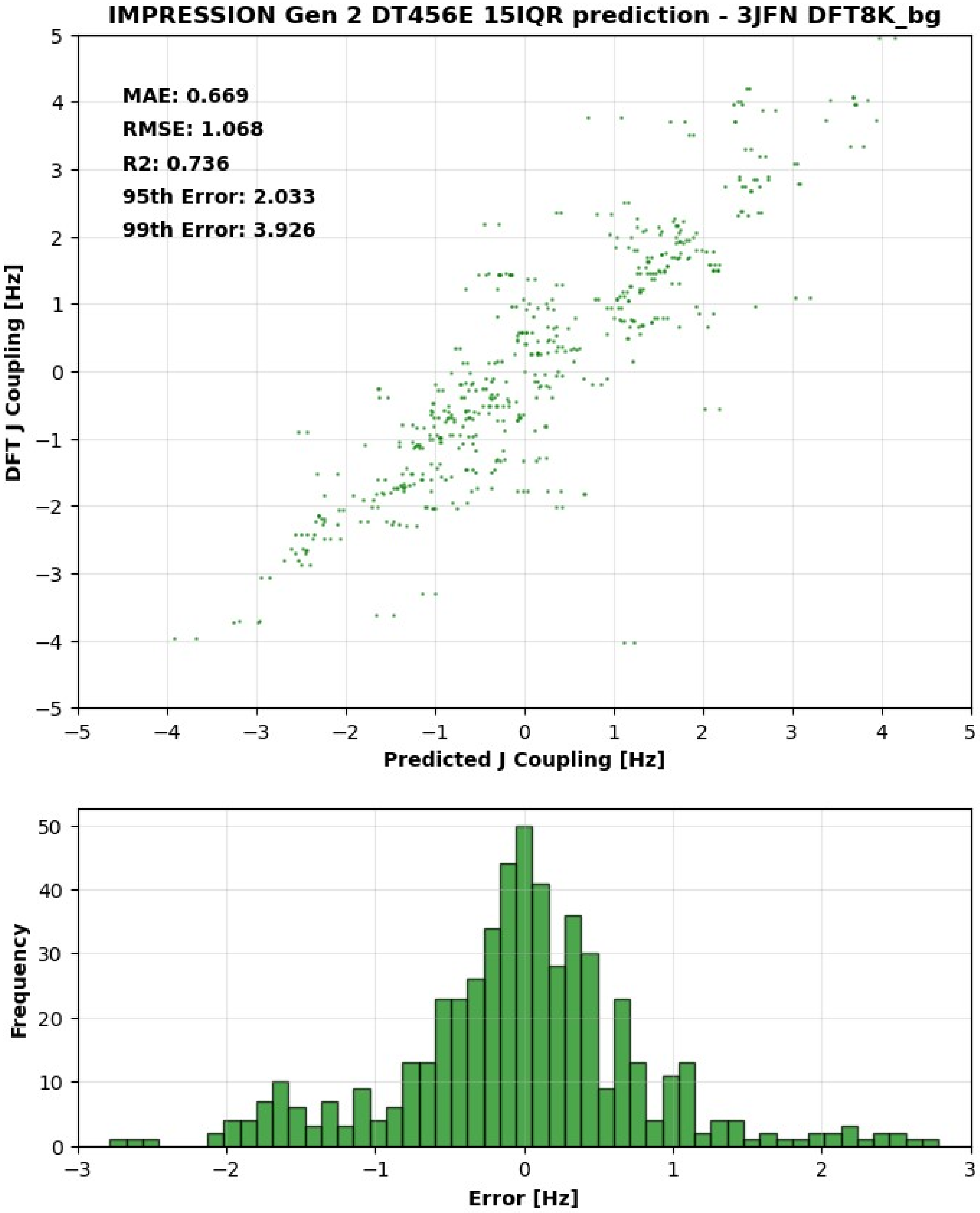

IMPRESSION Gen 2 DT456E 15IQR prediction - 3JFN DFT8K_bg
MAE: 0.669
RMSE: 1.068
R2: 0.736
95th Error: 2.033
99th Error: 3.926
DFT J Coupling [Hz]
Predicted J Coupling [Hz]
Frequency
Error [Hz]

S2.2.33 $^4J_{FN}$

Holdout

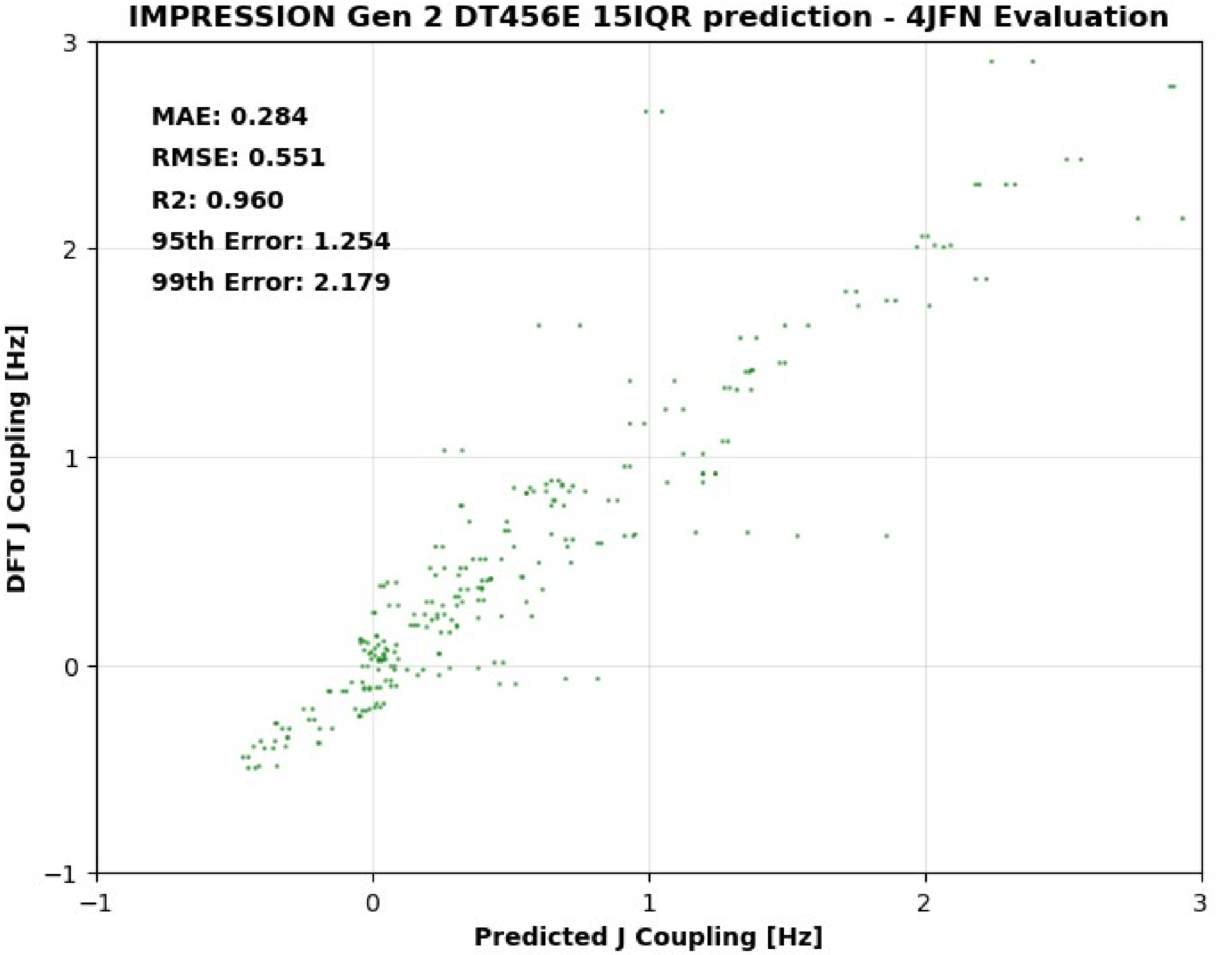

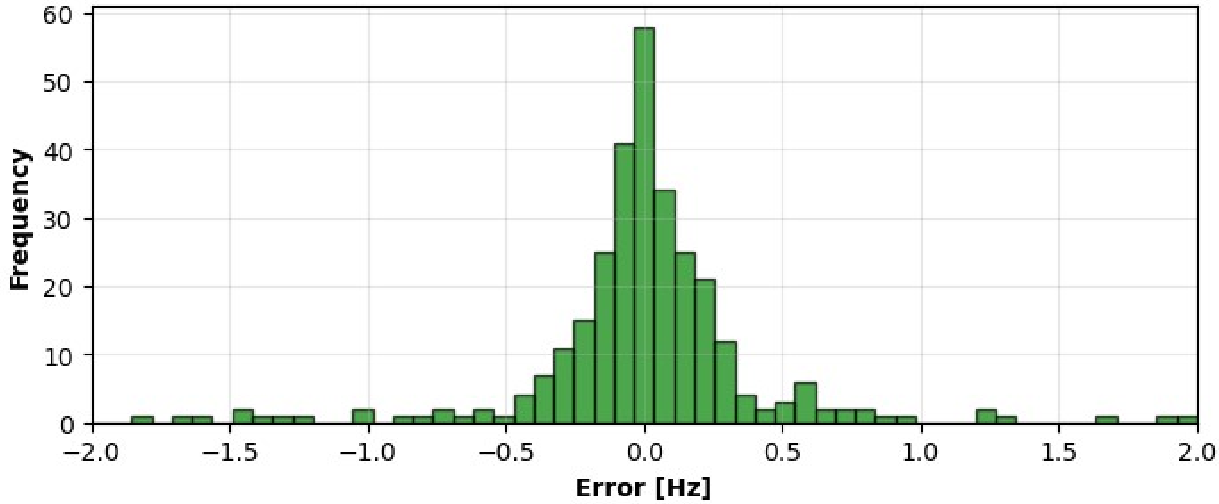

DFT8K_bg

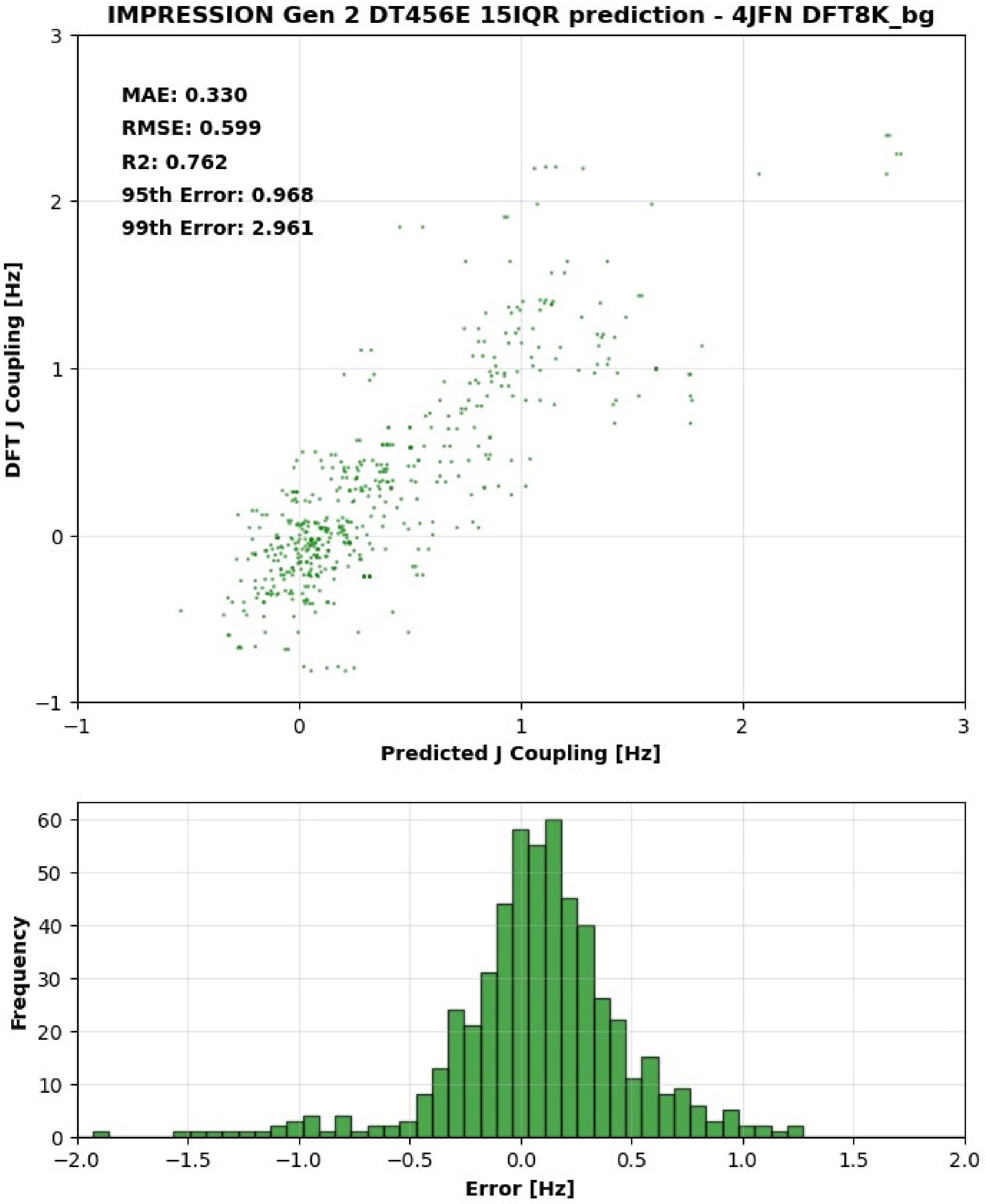
IMPRESSION Gen 2 DT456E 15IQR prediction - 4JFN DFT8K_bg
MAE: 0.330
RMSE: 0.599
R2: 0.762
95th Error: 0.968
99th Error: 2.961
DFT J Coupling [Hz]
Predicted J Coupling [Hz]
Frequency
Error [Hz]

S2.2.34 $^2J_{FF}$

Holdout

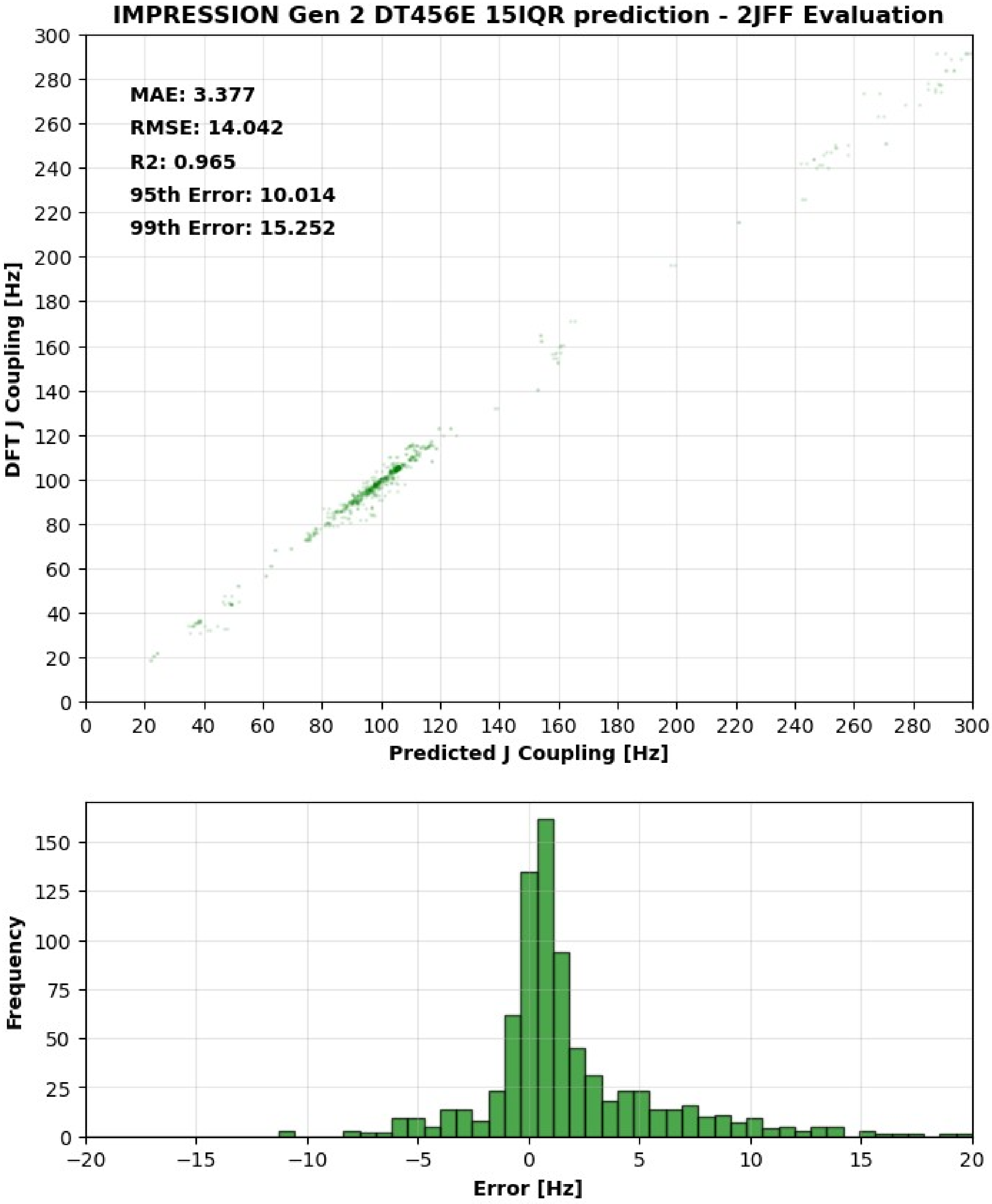

DFT8K_bg

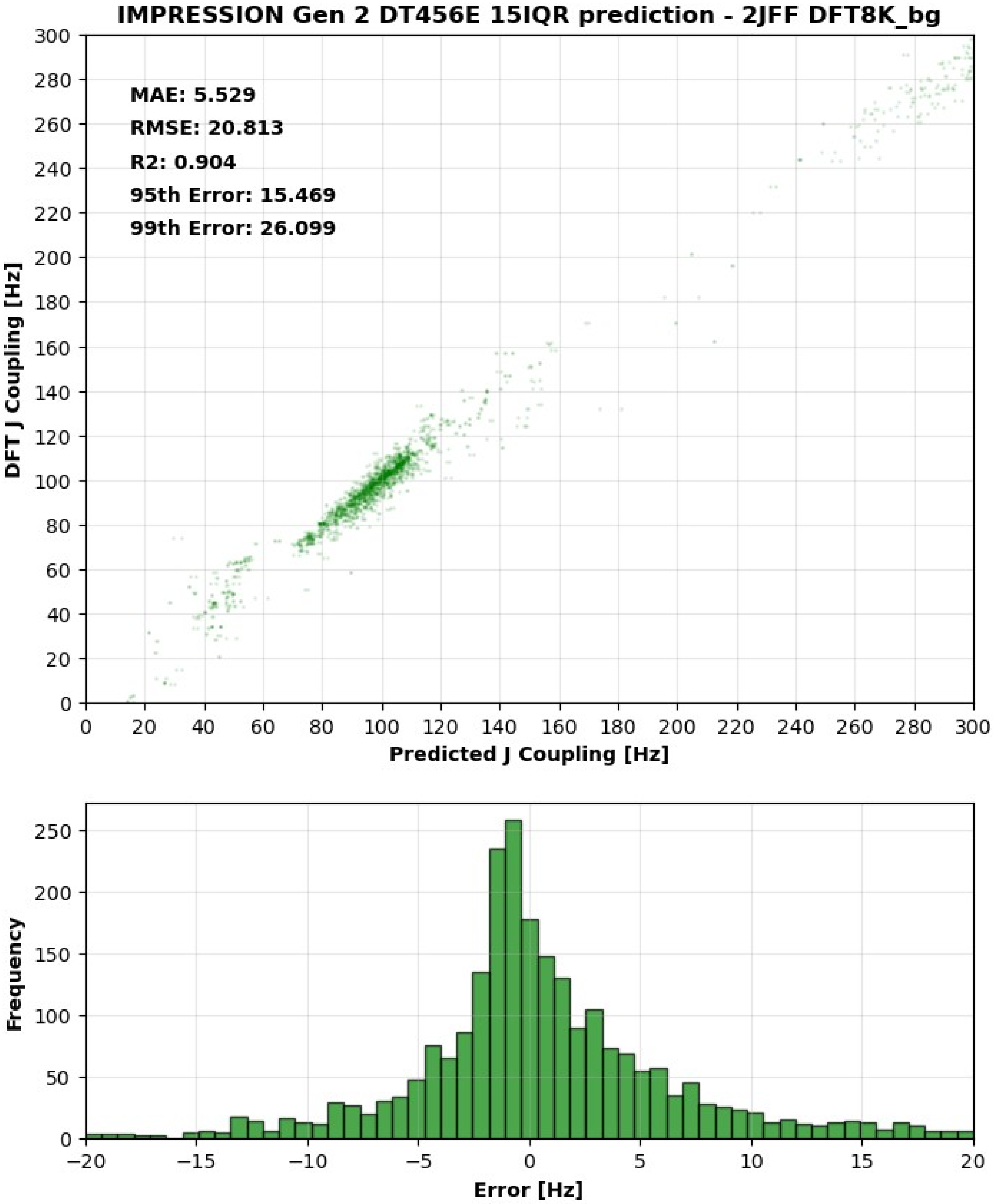

IMPRESSION Gen 2 DT456E 15IQR prediction - 2JFF DFT8K_bg
MAE: 5.529
RMSE: 20.813
R2: 0.904
95th Error: 15.469
99th Error: 26.099
DFT J Coupling [Hz]
Predicted J Coupling [Hz]
Frequency
Error [Hz]

S2.2.35 $^3J_{FF}$

Holdout

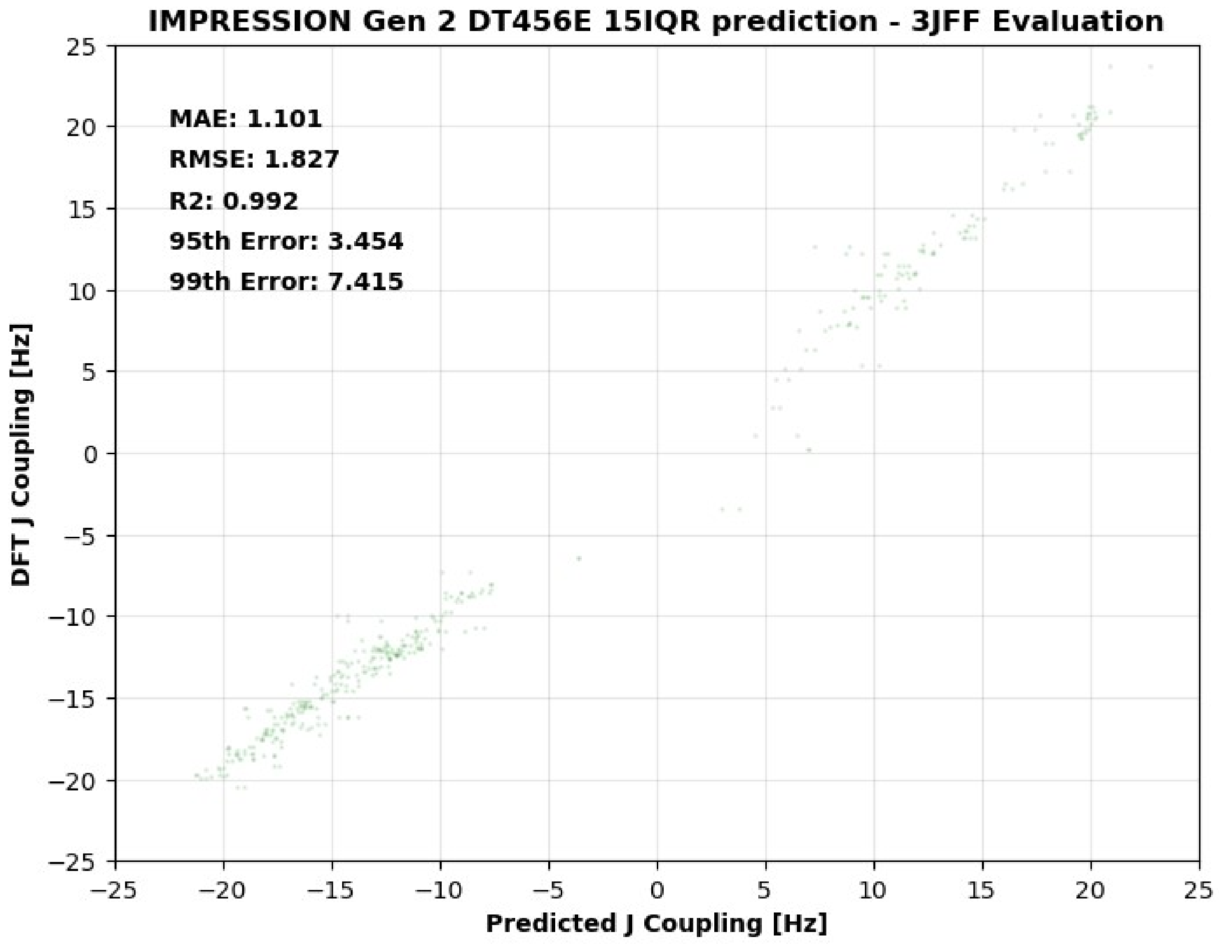

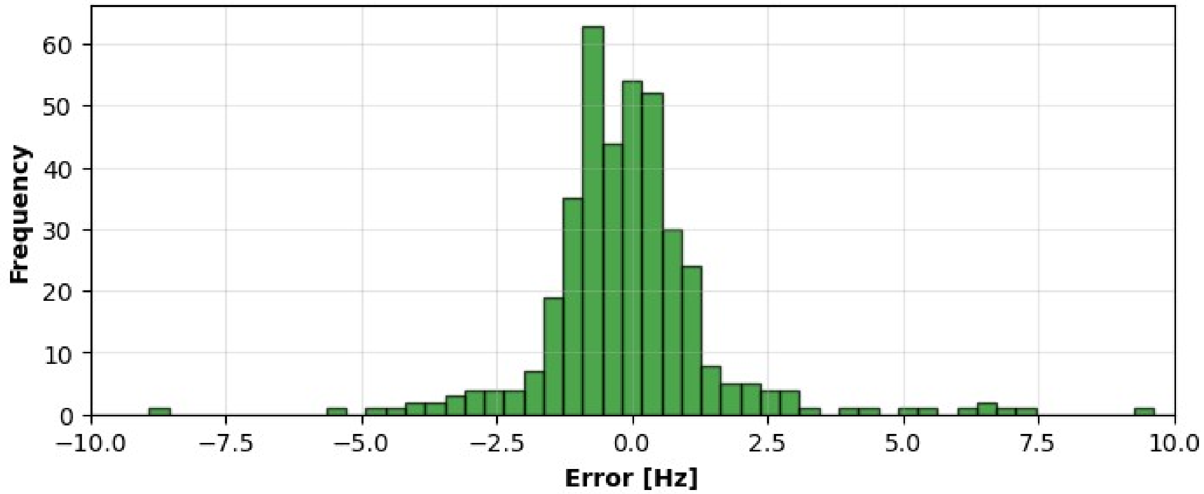

DFT8K_bg

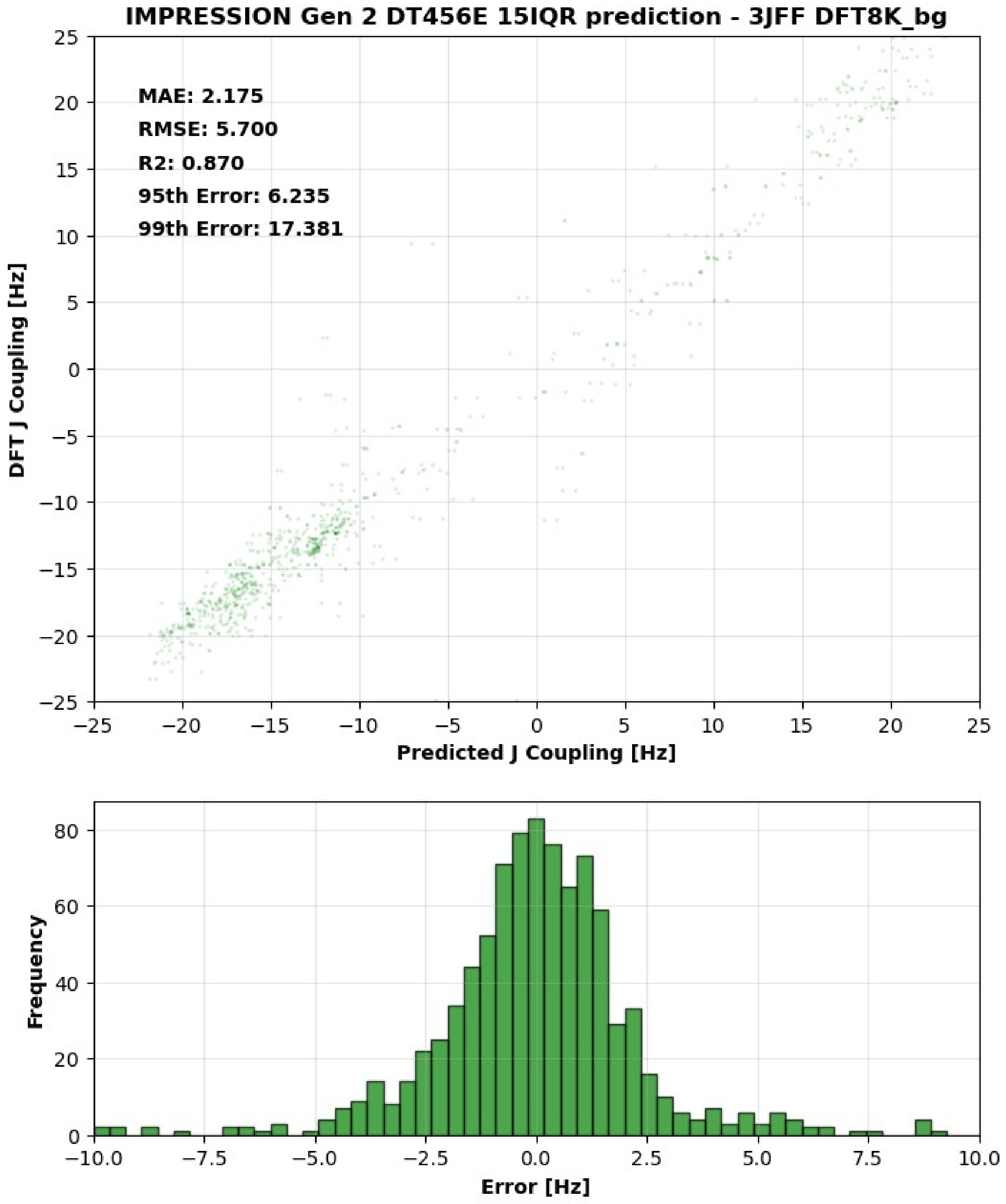
IMPRESSION Gen 2 DT456E 15IQR prediction - 3JFF DFT8K_bg
MAE: 2.175
RMSE: 5.700
R2: 0.870
95th Error: 6.235
99th Error: 17.381
DFT J Coupling [Hz]
Predicted J Coupling [Hz]
Frequency
Error [Hz]

S2.2.36 $^4J_{FF}$

Holdout

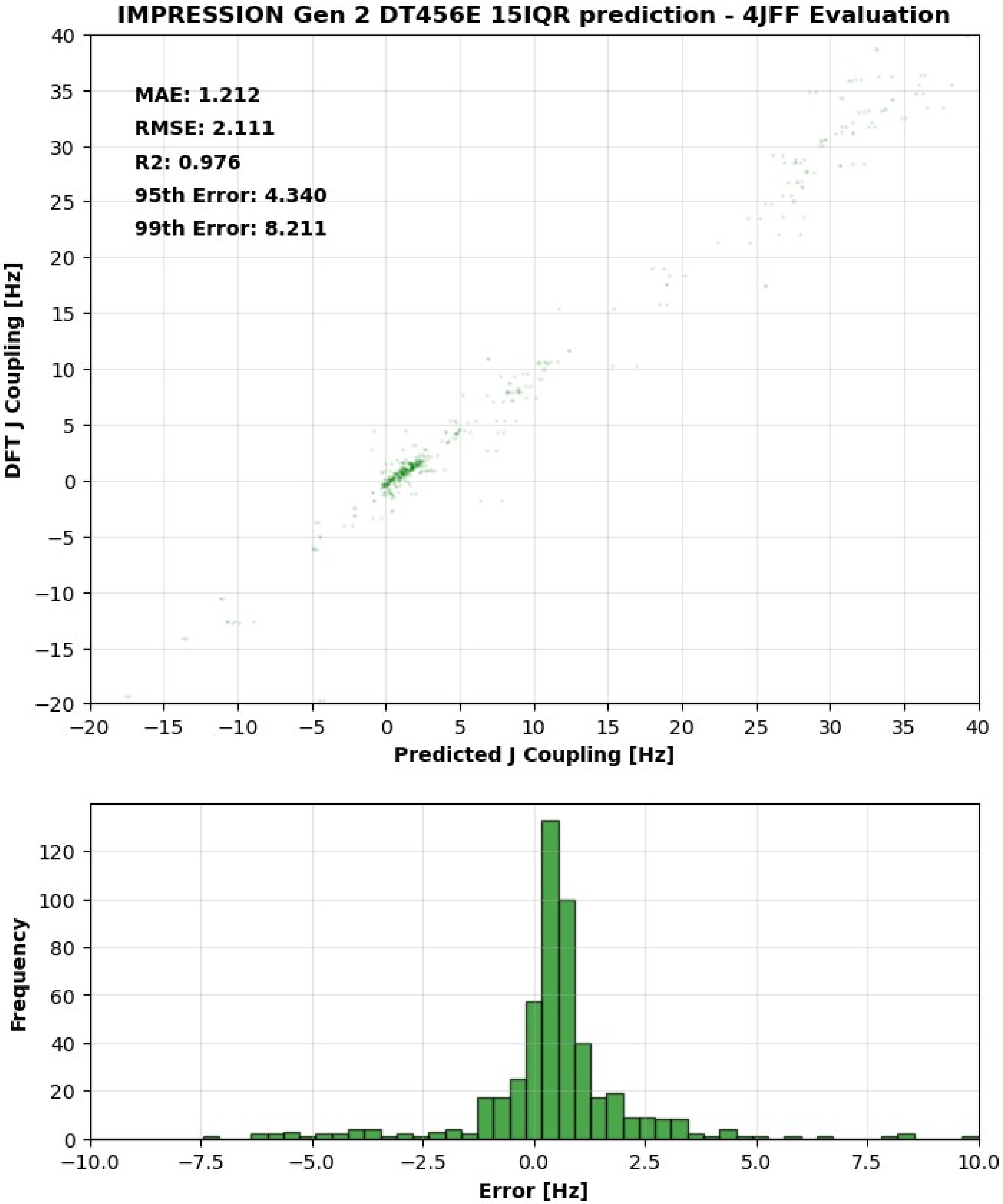

DFT8K_bg

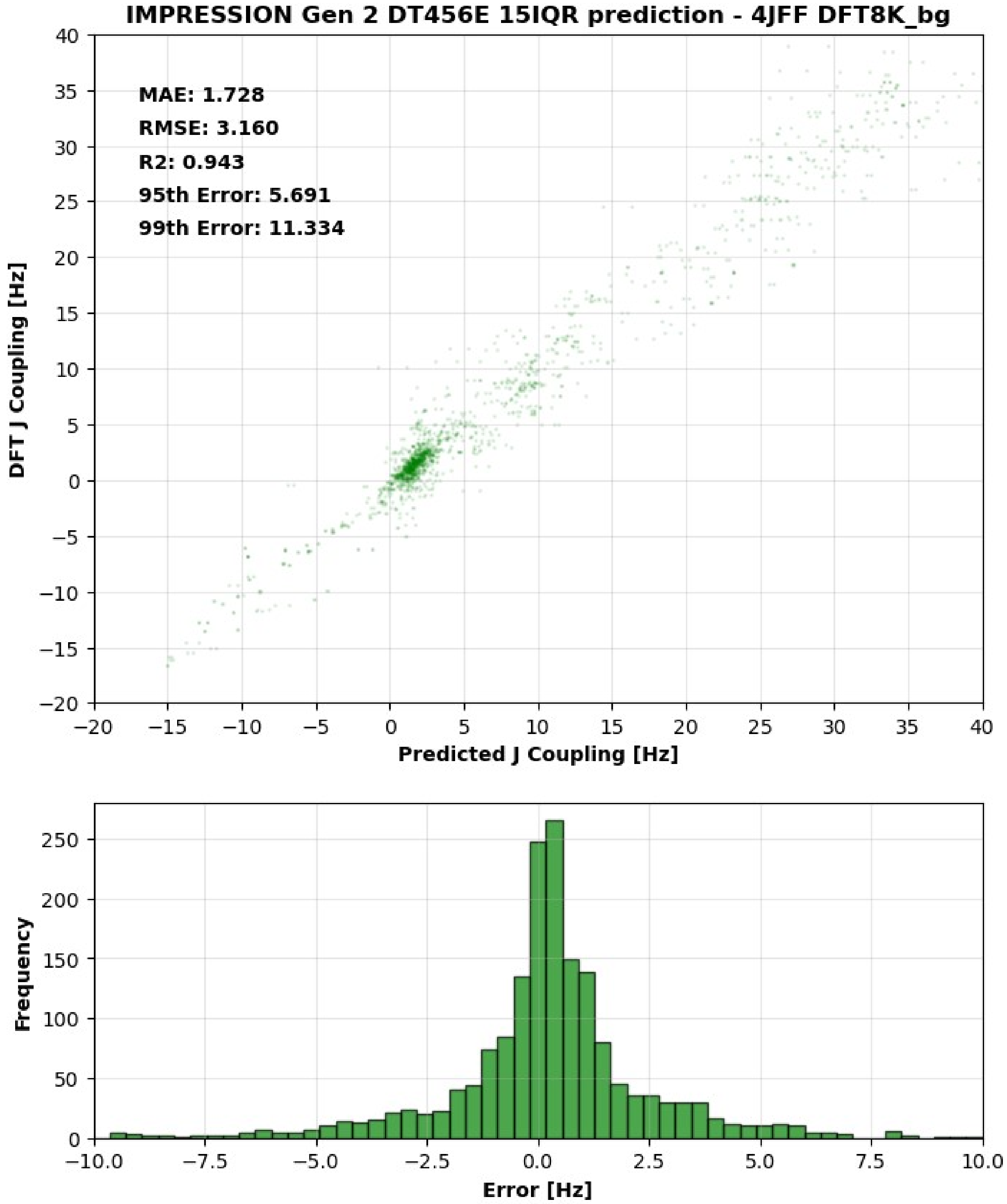
IMPRESSION Gen 2 DT456E 15IQR prediction - 4JFF DFT8K_bg
MAE: 1.728
RMSE: 3.160
R2: 0.943
95th Error: 5.691
99th Error: 11.334
DFT J Coupling [Hz]
Predicted J Coupling [Hz]
Frequency
Error [Hz]

S3 Comparison of IMPRESSION Generation 2 errors between various DFT methods on CHESHIRE Test Set

We compared the mean absolute error (deviation) of IMPRESSION generation 2 predicted values against DFT calculated shifts to see the deviations between the methodologies. The table shows a comparative close alignment with alternate methodologies at a similar level of theory. ωB97XD/6-311G(d,p) was our selected methods as it offered a reasonable balance of n>1JXY accuracy against the computational resources required (underlined in Table VI.7)

	Optimisation Method	NMR Calculation Method	^{13}C MAE (ppm)	^{1}H MAE (ppm)
**Electronic Structure methods**	**mPW1PW91/6-311G(d,p)**	**ωB97XD/6-311G(d,p)**	1.76	0.09
	M062X/6-311G(d,p)	mPW1PW91/6-311G(d,p)	2.52	0.30
	B3LYP/6-311G(d,p)	mPW1PW91/6-311G(d,p)	2.64	0.30
	B3LYP /6-311G(d,p)	ωB97XD/6-311G(d,p)	2.19	0.10
	M062X /6-311G(d,p)	ωB97XD/6-311G(d,p)	1.78	0.10

S4 Experimental comparison

S4.1 Strychnine

Strychnine was used to evaluate the ability of IMPRESSION to predict stereochemistry, it is a rigid molecule and so does not have as many low energy conformers as many other organic molecules. Strychnine has six stereo centres and so 64 stereoisomers which were generated in RDKit. Of those 64, enantiomers were removed and RDKit was then able to create a 3D structure for seven unique diastereomers. The remaining stereoisomers were deemed to be too strained and not possess an energetically accessible 3D conformation.

For each of the seven diastereomers, a molecular mechanics-based conformer search was run with the MMFF94 force field in RDKit. 50 conformers were generated for each structure. This was considered enough to capture the conformational space of strychnine. The conformers were then optimised by GFN2-XTB and a redundant elimination was performed to eliminate equivalent conformers. The root mean squared deviation difference threshold was 0.35Å and the energy difference threshold was 0.1 kcal/mol.

The conformers were processed in RDKit and run through IMPRESSION, then the predicted NMR parameters were Boltzmann averaged based on the energy calculation by GFN2-XTB to give conformationally averaged NMR predictions for each of the seven stable diastereomers of strychnine. These were then compared against the experimental data for ^{1}H and ^{13}C chemical shift and $^{1}J_{CH}$ coupling constant.

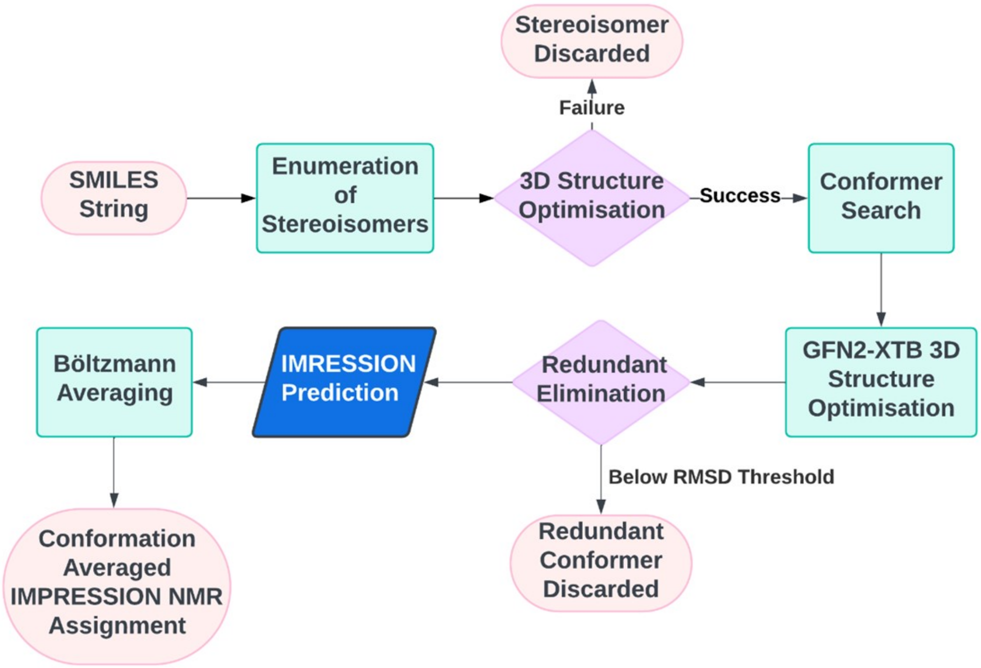

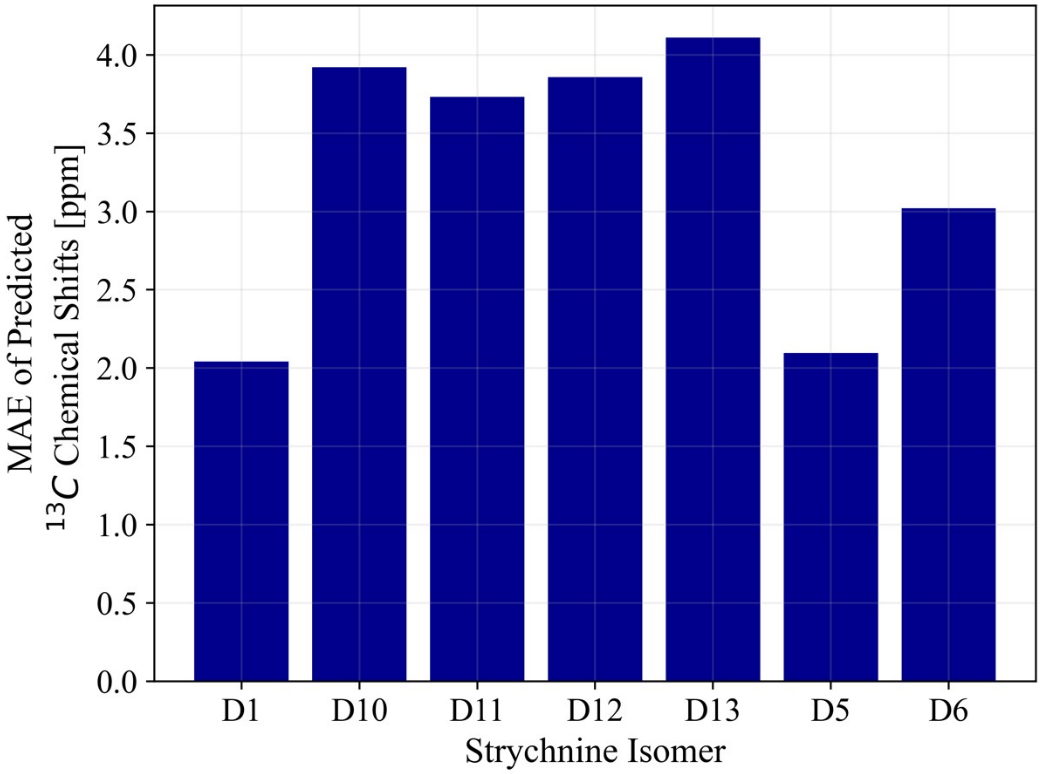

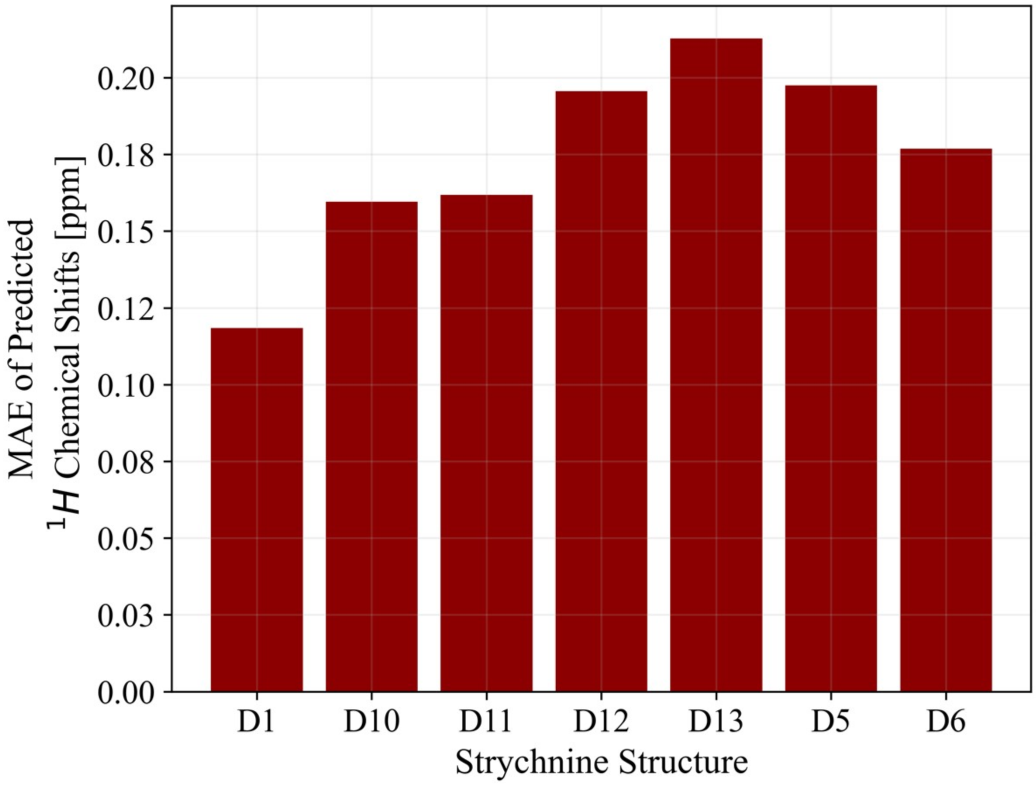

The diastereomer to consistently give the best fit to the experimental data is the right answer.
D1 is the unique naturally occurring diastereomer of strychnine.

S4.2 Exp5K

Figure 2 in the main text demonstrates the performance of computational workflows using IMPRESSION-G2 and DFT, compared against experimental dataset Exp5k from Guan et. al.[13] We note that experimental NMR data are a measured property derived from an ensemble of 3D conformers with populations controlled by relative free energies - and free energy is notoriously difficult to predict accurately. To avoid obscuring the comparison of NMR prediction accuracy with comparisons that also depended on energy prediction accuracy, we use the same approach as the CASCADE report and assess only a single conformer for each molecule calculated. To do this we initiated both IMPRESSION-G2 and DFT workflows from the same initial low energy conformer geometries thus removing energy prediction as an obscuring factor. Thus the workflows using only a single conformer (no Boltzmann averaging) were:

(i) Generate a single conformer for each molecule (both workflows used the same initial conformer)
(ii) Geometry optimize that conformer (GFN-xTB for the IMPRESSION-G2 workflow; mPW1PW91/6-311g(d,p) for the DFT workflow)
(iii) Predicting NMR parameters for that conformer (IMPRESSION-G2 for IMPRESSION-G2 workflow; ωB97xd/6-311g(d,p) for DFT workflow)"

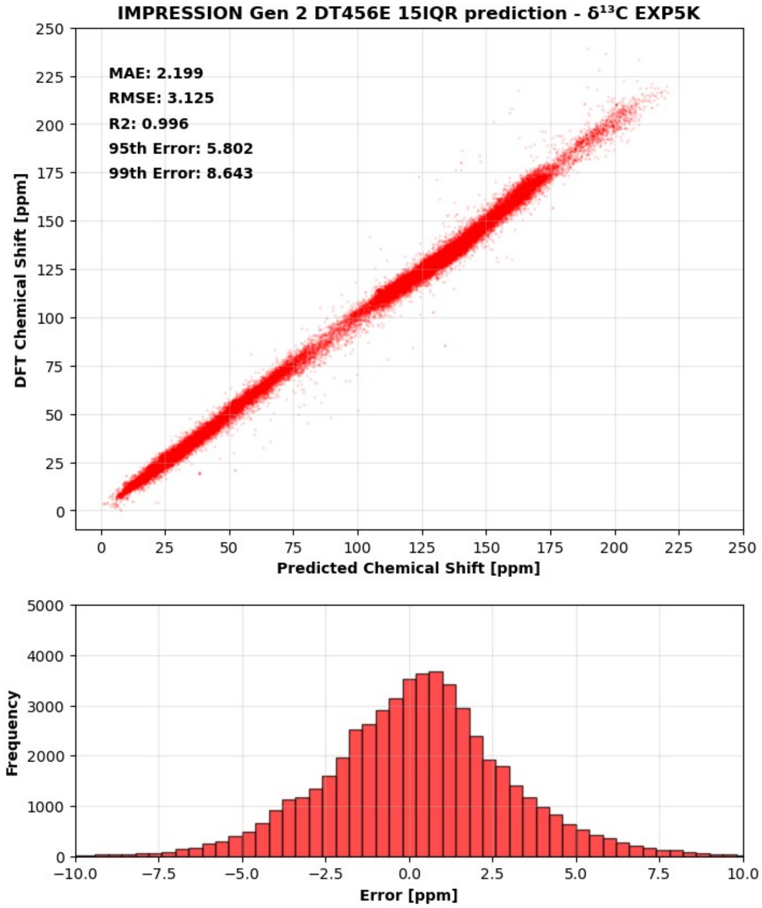

S4.3 CHESHIRE Test Set IMPRESSION/DFT to Experiment Comparison

We compared a selection of DFT methods, and IMPRESSION,

	Optimisation Method	NMR Calculation Method	CHESHIRE TEST 13C MAE* (ppm)	CHESHIRE TEST 1H MAE* (ppm)
**Neural Network**	XTB	IMPRESSION-G2	3.19	0.22
**Electronic Structure methods**	**mPW1PW91/6-311G(d,p)**	**ωB97XD/6-311G(d,p)**	2.31	0.16
	M062X/6-311G(d,p)	mPW1PW91/6-311G(d,p)	2.51	0.15
	B3LYP/6-311G(d,p)	mPW1PW91/6-311G(d,p)	2.73	0.14
	B3LYP/6-311G(d,p)	ωB97XD/6-311G(d,p)	2.58	0.15
	M062X /6-311G(d,p)	ωB97XD/6-311G(d,p)	2.37	0.16

*2-cyanopropane, t-butylacetylene removed from CHESHIRE Test set as DFT calculations failed during testing.

S5. Sampled structures

S5.1 CSD structures

**VESHUX**	**OLOJAB**	**PARHAR**	**BEDJOM**	**YUNYIR**
**CASTEV**	**NESZOB**	**YAPZEU**	**BULKID**	**NUPQEU**
**PAJVOO**	**CIBFEA**	**XIWREA02**	**INACET03**	**PEGLUL**
**UYUDUO**	**COTMEE**	**TARGEB**	**FOCBEF**	**BIBXIT02**
**OCEHIP01**	**MIQNEF**	**JABKUV**	**VOFSEP**	**XAXHOW**
**FEKDUU**	**DAWYEI**	**BULHIZ**	**CTOGBS20**	**JECNUD**
**FULZIV**	**ZIFKEG**	**FIKCAE**	**NEPXOX**	**DICRUD**
**RUWMAX**	**SELKEB**	**ZETHUD**	**HOVFUT**	**KIMSUU01**
**KUVBEI**	**VUFSEU**	**DOTPOS**	**MENNAV**	**SAKJUM**
**EFIKOT01**	**SEHNAW01**	**VAPCEW**	**FOMZUD**	**VIGXAK**
**KUXJIY**	**VOLKIS**	**SUFGAB**	**HOZGAG**	**OHIWUX**
**XEDNAX**	**HAHVIY**	**TUCNUC**	**LOMHOK**	**RUWJAU**
**VUPHIZ**	**EWODEA01**	**XOGXEX**	**JINHET**	**OWOHAL01**
**AGAVOU**	**QIWMUG**	**FUCVOO**	**NOFYEM**	**MECZID**
**ATUJEF**	**XEMDAX**	**WACZUX**	**LUPGAG**	**GITNEE**
**VUDKIP**	**WAZMAL**	**AYEROL**	**CEPKIS**	**MIHZUZ**
**GOCCOS**	**SIQQEP**	**WUSQUY**	**UHADOX**	**AXEHAO01**
**CIXGOF**	**ANIZUT**	**XEZYIK**	**YUDLAM**	**TALNAV01**
**GEYTIN**	**FICTOC**	**MAXDUL**	**SAPHAU**	**TATNEI**
**SAHZAF**	**URESOB**	**TOPSEW**	**OJAQOH**	**LEPPIF**
**NAMZAC**	**ESTILO03**	**KATKIA**	**SUZJAZ**	**NIVMIQ**
**CYTOSM13**	**QUYJUQ**	**QEPNUW**	**SIYYUU**	**BUYZUQ01**
**FASZOP**	**JUNJIN**	**YUQCUJ**	**QALZUA**	**PAGLEO**

KAGZIE	OLOREM	KONTIQ01	BEMZAV	GODSOH
BASDOO	XIJFEB	TUSQUU	ELENEQ	CUSFEC
POZWUW	DAFLIH	NACGOP	RAFINO01	DETPAU
YARDUQ	COWLUX	FULJON	EFUMUP	XAZROH
LIWFEC	CIGJUX	YIDPEG	YOXGIB	CEKPIR
ZZZMBS02	AMUVIP	XEVCEH	ONILAZ	YOKYOO
HIFGEJ	YIFWAM	NINWEO	SAWHUV	QAKMOG
BAFDIV	OMCHDO	AFIHOO	LUQSOG	VIBZUB
JUSQUL	DAJXUI	BAYZUW	LOKDEW	AWOTAH
EDEKOQ	VAJVOU	QAKJUJ	DODWOI	TIQWOG
TARGUO	BOVJOL	VEFPIF	WOGQEO	CMXMCH
ATOGIB	RUCFAX	HURLAI	KUVKES	QIRLUA
NUKXEX	KIGQIA	NIJKEX	TPHETY01	DAQJOV
HDPDXZ	WOBWUF01	CUDSAX	FICLEK	NEWREN
COFGUA	PHTHAC02	CIQHOA	BANJOQ	BOCHIL
HOQSIQ	KUWZOS	JEXBOE	KUKCUP	SUCANH12
BAVZEE	XETMAL	SANWEJ	YUHTOK	PENTYN
TUJJEP	AQUWOY	QIQYIA	FOGKIW	AXADUZ
VUTBUI	PAYJEH	WESVIZ	REZJUC	DOVGUR
PEXLAH	TECQEX	QECHEO	GUHXOY01	NEDYEA
AFIFAX01	TICBUD	URAWEQ	KOKLIH	UCOMOO
SUVCUJ	ITUVOI	CIQYAD	MOGYIR	QANQUR
WOZPUW	RUJQOE	JAPBIO	PADTIX	YEXZIM01
VACLAM02	TIHBAO	CISXOT	COFNUI	HOWWOH
MTYROS01	SUCROS47	RAMZEL	FUTWAU	LILDEP
GIMGIU	HOCPUL	KIZVEV	UCUZOJ	EGUQAY
GATVED	JOZYUU	EGOTAW	SEQREN	TIXPOF
MATVAE	SIWDEH	HALVAT	TOVSUS02	DIBENZ13
POSJAI	MOFCOA	TIQNIQ	NEXMOT	TOPROG
PORROE	HEQWOQ	KEMHAL	CANPEM	BEJTEP
YUNTOR	BICVIS01	UFAGOY	EGAXAL	AVALAM
AHMVAL	HIGCIK	ZAJVAK	LEGXUS	XUVSUE
XAYDIK	FAHPAH	TIMHED	PEXFUT	NUHFEB
REBXON	FOFQOG	EZUJIU	VUZQOX	AXADAF
BASHUA	METAMI02	COYREO	MOBXAC	BZCPRO
MEQFAS	EZUTIC	HEVDIW	HIFQET	XOBGAY
GEHXEZ	YOGSIY	XENLAE	MUVCAI	REGFER
CEBQIK	DOPSAC	NAYZOD	LAFHEH	WECXUZ
YEKVEQ	DOYVUK	DIGGOP	WUYMUZ	ICEMIO01
IHOQUT	LEHJAM	VIHBIZ	BUBPAQ	PEPGEW
SEDMOD	QIKJIF	LUVPEX	WEWTUP	DEYTIL01
LILJOG	FEFYEX	HEXVAI	SADXOL	QAPVOT
RALQUR	NOQBUQ	DUNTOV	CUKSEG	SUXCAQ
NAPTYR11	IVAKAS	FRANAC04	CEGREL	CITQAY

EHAJUS	FNPEYO	VUNFUF	GEFLEK	ITIKEB
XEHTUZ	NAFHOR	BIFFAZ	NEMZAG	DEBDIX04
RIFBUE	BUDHOZ	HAWTEF	YEJPAG	VIDFEV
EKAHOO01	JOCDAG	MATPEC	YUDPAQ	ITAFEP
DLHTDA10	ZUPGUM	MAMKAO	GAMLOV	MIPYAL
FACWUC	ECIPIR	CEKYAS	QAJBUZ	FOTYAP
RIWNEQ	MULBIE	BOAYPI	DLALNI14	LUQYIG
EMAQEQ	ZOLBUX	NIVJAE	SAYWOG	DETLAQ
HXOCTM	SAZLAH	WIPHAG	ILIMEV02	FAFXUF
TENMIK	LOMNUY	KOWCAC	GUKXIT	KAYHIE
XOGWAR	BZPHAN01	ERISII	DOQDET	SUCTAN
IDILUD01	RUJSAS	CIRGOB	DUNSAH	NISMAD
UQIMUE	ICOYEE	JOYGEJ	IQUFUX01	NETIND01
YUFYED	VUTNAB	CUZPAP	COCPAN	UTIHOV
ORIDAW	FEHLEL	EYIKUS	FABVUC	CEFBOH
QUVPOO	TUWCEU	YIGSUE	ZZZLUK05	ETIROQ
PINVOX	QUWJOJ	MIXWEX	MEYCIC	NAMZEG
TANBEP	UXICAH	MENSEE	ZUQVOY	FAMFII
TABNIV	ATEZOO	HIFPIX	PAXCEX	KAVCOC
KINGUJ	GENFUA	MEDLEN	DLTYRS	XEWNES
YUCQUJ	COMXOR	PHTHAC06	CUGLIA	BOMBEK
UYIREB	YAGJEX	OQUHEP	TELKAZ	GILKIW01
TAPCIW	EYOGEG	TUNTUT	MINGAR	FAVYIN
BEZREF	BOPKAS	DEVCIR	ZIYSIL	UKUTUP
CEBKEZ	TEPHME02	CTPROL10	NIHNEY	QAZMIP
RUGCED	LGLUAC13	WOBLAA	ENIJIV	LAVCET
KIXROA	CUVBIF	CAGMIJ	UWACEB	ECMPCA
WIBXUA	QAHSOI	PINYIW	YIHHON16	NATNAA
GASNEU03	OGOXEP	IVIDAS	YENLAF	XINJIN
YAQWAR	MUHZUM	HNOBCH	NIFBEJ	SAGQUO
XIVVAA	UTAGAZ	BOVCEW	SUSYAI01	OTAKEB01
VECSAZ	XISHOY	EVOGOM	DEGREM	RUWQIK
FESNOG	ADAZUB	RURRAY	WIVYUV	RAYXEU
EVAWEE	SEJWOT	YUHTEA03	DUFVEG	UZUHED
LOVCAC	FECQAF	DOFGEK	MVAHIV	MEWROX
ZEWPUM	UPADOG	VUFWAV	SECTIF	MUNWUP
LACVAM	DEBGIB	LOSMOW	TULDAH	CAHBUL
SADJEM	NAXRUC	YAPBUO	KOPBAS	JUPJAH
QUDREM	AHUHUH	XAZQOF	IVIHAY	MOYKUG
DMTCUN10	TEVLIQ	WABTAU	TUNCOW	NIFRAX
ZUPHAT	VOKXOJ	TACRIB02	SUPKET	WIBWIN
PUDDUP	CEMBED	AHUYUX	PIPINE01	REGYEJ
MELVAA	GEFQIS	FOQNUV	GICCEA	WADGEO01
USUZUF	REYCII	UPACUK	MEYWOC	XUHPIB

YOPLIY10	TOHVIW	YONBOT	COBROB	BEWLOG
TALHAR	BUGKIX01	HXMTAM10	BONDIT	AMAMOR
GUYBOR01	GUFXOV	YUQMED	BIPTEZ	CAVQEV
IHANAG	SAWJUX	MAQWIM16	AFEBAO	ACIYAP
ECASAC	CONNUP	ELOKIB	ATELOB	COZKUZ
SUCACB12	IBUYIQ	XIMCOL	TEDJOE	ACUPEV
ESUQOZ	ROSLAO	NUKJIO	BAKVUE	BEBCUG
EREVUS	HAKWUN	CEHZIY	APOHOE	ASUHUS
DIBNEH	JOTBAV	POBSAB	COFHEM	DBPOLO
CEBKEZ06	XOMJIS	PENBUH	BUCZUU	CEJJOR
MESYIS	LESCET	RAYXOH	DABPED	WEFXAJ
SOXHAQ	FOGBIN	RIXXOM	WEGPAC	AHIGAA
DIKFEJ	WUWMEG	DISJEW	CBZMAL10	BESSOJ
NADVIX	WADQID	TEGVUW	DAHSUD	BOFKOX
DUCWAA	BUGMOG	WIFZOC	AGAFAR	AGATOT
CIKBUU	SUXROS	FOLQUT	DIHLUC	AQINIX
XEYRIE	AFIGIG	ZODXEV	ATIRAX	ACEDES
NIFJOB	IGENOZ	EVAWIJ	BEWKUJ12	HEFFIK
ARIWAB	PEZFEG01	IQIDIV	WEHKOM	BAYQUP
AXMQOL	XAVZOJ	OBOWOU	DATWEB	BIBJAY
SOPLEO	GUMMOZ01	MUKBUR	AGARUX	ALEGEF
PAJDOU	HELYOM01	VEXCUW	CAGLAA	BATGOU
BAPQOA	BATVEY	BEVXAD	CEGJOP	DERSID
GELDEI01	MEGNES	ANIBEG	BAXWOP	BASLUE
PABBIF	SIVJOY	CIPWOP	BEXYEJ	DEJXOI
CATKAL	RELCUH	BEFROU	CAPDEE	BONCIS
HIYHAY	TUCJEI	BAZZIN	DBPHEN02	BIBJEC
QEYRER	MIDXIH	ABOBIF	CUPMOR	BPSIND
FERTON	ZOFCUU	COSROT	BOLDOV	ABOGAC
DUDKUJ	BEFJAY	BECLAW	BZDCPB14	TEHCER
WIZZAI	PAFGUA	ABEMIG	BEGYOB	ALETUG
CBUDCX02	XUPYIR	CEWLIA	BEWMEW	AZPACN
IQIZAK	PIGTAC	CEKFOP01	QEFLAR	BAGVUB
WAGBEO	SUHYIE	CEBTEI10	BEWLIY10	BAHQUX
JEGTUN	QIMKIG03	BILBOP	AFUYOP	CUGSAY
KOXBEE	CIFSIV	CILLUG	CACRIK	BALJEG
COLYIN	XEXQOH01	AHEPEI01	AMODOW	COWKIK
OPOZAW	NAPHTA23	ANOCOW01	BHPETS10	BUBVEZ
ESUROZ	FEVHEV	BAGKEB	BALNOU	BAPXOI
MEYTUH	HAFDIC	BOPQIG	APOWIM	AWOJIH
JUMCEB	ABIVIQ	BOXQIQ	CIZGEY	BEVQIE
IXOYEA	WALNEC	BPAFAC	BEDDIZ	ANURAE
LIHMOG	FACQUV	AXOLIJ	BEPNIU	BOGBEE
FEQFIT	NIYWID	BABNEX01	CUTJIM	DEBHOH

ARUWAL	BANTES	DAXKAP	BASMOZ	BZOYAC06
ACULUH	AZIHUO	ZIFROV01	ABULIT	BATROF
BAGLIH	BEBWIP	COFZOO	CUPWUG	BEGJEE
BBCPEO01	BEGBAQ	CEBLAY	CUKDER03	AFUZUW
HAZWUD	MEJREB	AHATAE	QAZTAP	DATSEY
BODCED	BODTOE	DEFWIX	BIMCEG	CEJLAF
DBTACD01	CUVQEQ	DIDMUZ	CIDJUV	BEPYIE
BUGKEU	AVOSAH	BARNUE	BIDRAH10	ACUXAZ
CAKSAJ01	CIKTAS	BASVUM	CUHQEC	CAXREB
CUFTEC01	BOWBEW	COKCOU	CUVWIB	MEFPEV
ACORES	AKOCEJ	AWETED	CANTEQ01	BAXYEH
CACGET	CARJIQ	DICWIW	GEDLEJ	AFAWIO
BZYACO04	BEVREZ	DEMDEF	BALTUD	DARXUS
AYASUP	TAYGEI	CUMKIF	BUGDAI	ATYREE
DASBIL	BUFFAL	VEHSOT	DAWSID	BZQTCQ10
DARVEX02	AHUCUB	DBOXAN10	AMAXAP	WEDKEY
CADWOV	DEGLAD	CILBOP	BESSID	AHEPIO
BZAZPO01	CURDUP	BODMOY	COQDOB01	COSXEO
YEHYUI	COUMAR11	COZFIG	BZOCND	CEFVAK
CURQOV	CAZVIJ	VEJLAA	COBMUB	ACALIA
BUVXAT	BUBPAP	BIJRIW	AYERIH	CIRHER
AVUGON	BENANT03	BEKDEB01	AMATUD01	BEGSEN
NAZPIQ	BOZYEW	BAGVIQ	ABIPOQ	CAXMUJ
CEKFIJ	ADIPOU	DIKSAR	BILCOO12	AXAMER
ADIBOG	BECPIL	AWIXUZ02	DAPHSD	ABUPOC
CUBHEO	DADFEW	ADUNET	AGIYOF	BEDRAD01
CUDXUU	CAPZIF	BASLIS	ABADEO	BALQEM
CLBZAC03	CUXBAA	CAMFOO	CEJWAP	BIDYOE
DATRAT	DAZIMP10	DEJJOR	DCACNP	AKUNAX
ARIGAL	CIBRAI	CIHJAE	BABYIO	CADZOW
NAZPEM	CIYWIR	COBFIK	CEBKEZ02	CEFQIP
CUTLEK	AXOLOP	ACOSIW	CIMJUF	CAYREC
ATIYAF	DEZBEQ	NEBZAY	CIVRUU02	BUDRUO
AMPHFR	BIFLAF	CEFCOU	BUDJER	CACYEN
AZUWEY	CEMGEI	CILKAL	AKIDIJ	ADISEM
COJYAD	DANSOD	CIQDUD	BPTTAZ	ADOHIK
CEDYEQ	CILZUS01	ABELAV	AFUTAX	ARERUL
BEFTAH	CEGCAS10	ABIJAY	BUHJAQ	AZUGIM01
BRBZPH	BAKVUF	AMUVEL	BEPNUG	AFEPIL
CIQFEP	DATDEI	CITSOO	CAFNUU01	BOGXUQ
BULJUN	AXEPOJ	COPTEI	BOKTUS	CUFHES
YEBMOK	ADUPOF	DARVOH	BAVTOK	QEDMOE
AXUDED04	COYKIM	BUCLUI	AROHOG	ATUVOC
CIYWIQ	BAXJUF02	BAQBUS	CUWYUP	CIBMEH

CIKTIA	BABBAH	AXODAS	COZFUT	DATCAF
BIMPTZ10	AJOMUI	COMFAN	CUJHAQ	ACUNEU
BALKAC	CAYKIZ	DALZIZ	VEDBIS	BECPAC01
BADBIU	CEHDAW	CILFAG	BADTIM01	AJIDAA
VEDGOD	BORLIF	CAQSOD	DBRNAQ01	CAQVOH
ATAKUB	CIPREA	ACMEBZ	APEQAO	BONKAQ
AHEFAV	CIMYOO	BZOYEY01	BOBDAZ	BNPHTA01
ASIJOB	CIZNOP	BURGIG	AXOQOU	CAKXUI
ABOFUV	DATTID	CAGFEX	AMAWUI	BAPRUG
BUTYIA	AQASOB	AJIXIC	ATELAO	COBJUA
AYATAW	BOKCEJ	DIMROI	ASOHEV	DEJGII
BAXNIA	CITMAV	BALSAK	CELTES10	ABUBEE
BAWVUR	ANTMEU03	APAVIX	BIXHIA	ABEBIS
BESZOQ	ADETUA	APUVOX	ADAVOS	DAVJER
WEDHUL	QEDSIE	CARYEB	BAVHIR	BAGCER
BUVZAV	BZTZAC	BOCFAA10	DAYHUJ	CAGGIB
CEVTAA	AFUBAG	CINNUK	BZPHOS10	BEYDUG
CAYJUI	HEJPEU	AJOPIA	CEGWOB	DIJYIF
BAPTIX	CEHROX	AZORUD	CIVSIL	CURPUB
DANTRO01	CAPDEF	DHNAPH05	CEZDOA	HEJLAM
BONWEI	BZAMID13	DATTEZ	COPPOO	BUSYIZ
CIVLOK	COJCIP	CAZMOH	TECHUH	CIKCIJ
ADAJUK	CAQVAS	ABUVIE	BUWBUR	BUWBOL
ATUTEQ	CINZOP	GEHVOH	BAWWUS	DAXGOZ01
AXIFET	AQEREV	CUMKEB	ANUKIE	CAKSUF
DADPAZ01	ACRDIN09	DAJXAO	BONQAX	DALGAY
ALUQON	DEBRAG	DAHHAY	BEPYEB	CEGMIM
ACAYIN01	DAMHIL	DERKEQ	ARARUH	BOZSOZ
CEDJAX	CIVWUZ10	AVOBOD	CARGOS	CAJWUG
BACQOM	CIBMIL	DIKLOZ	ACUGUA01	BIGXUM
CUPVOA	AZUJEL	CATYED	QEGMAT	AZISOR
AKIPOB	ALAJUT	BETQAU	CERJIT	CEGSUE
DADGOF	BUYPOA01	DASNUG	CETRUQ01	DIMNAP04
COPDIV	BIBZUJ02	BOQCEP	ATALOX	ACORIV
AQIKES	DAVJUI	ANINOC	BEWDOW10	BANHEG
BESVAW	DANZIE	DIDRAK	CEJRIT	BUVDIG
AFIRIS	CUGDUE	CUTLIO	BORZUE	WEFWUC
BEBRIJ	BPACLA	CAWZEG	DEXWOU	AFURIE
DAYMAU	BUFZOT	QEBVIF	COLROL	AFUZOQ
ATEQAS	AZEKIB	ATISED	DALLAF	BIPZUV
CEGXUJ	AYEGAM	BICFOI	BUPCIZ	CIYTEJ
ACUTOJ	CAWWAA	AKOHUF	BODYUQ	CLDZPB
CURRIS	DEFZIY	AMUXUD	ACENEB	BOCXEX
ABIBES	APUCEU	BIMJEM	MAZQOW	CURPIP

BOVTUD	BOSBOB	APAKOS	CEDBIX	DIBENZ06
CONPAW	AHUSAY	CEWWEH	ACORER	GEHCUU
BAHBOB	BECRUY	DESWAZ	AZAFAJ01	CUBVOM
BEQXUQ	ACSALA23	CUTTAM	AXURIW	BEQWIE
ADOHOS	CIDFIF	AKUXOU	DEDLOQ	CIRKEV
AXUGUX	CEJSOZ	BUHLUN	CINTIE	AXIYEM
DAVCAH	CHMPOA01	CEZGIZ	MEBZAX	BEPJOX
AXOXAN	ABIHOI	YEBMUQ	ANUCOD	DAZBAJ02
DAVBUA	AYUFAC01	BAWQAS	DAVFEO	BCPSBZ
BERDUY	CUBTEA	BABCUF	AFIFOK	BEHMUY
DEMTAQ	NEHVOO	HEHSOF	BACTAB	DABZEN
ALEJOR	BEDREJ	CEGYOC01	BEDROR01	DEZJEY
DACBAM	ASULEF	VEBKOF	ABILOM	YEHFID
ARANEO	BANBEZ	DABRON	YEJJAB	BOHMUH
ADAVEH	CADFUI	CONSII	AVAGIN	CISPOK
DAKJAB	CEVQOL	CIMDUZ	AXADIO	COPQIJ
BORKID	ALURAA	BELPUF	AJIWIB	ACIVEP
CIKFEH	ARIGEP	BIXGUL	COKCEK	COZROA
APUTOU	GEDQAK	BIHMOW	BUVSES	DAHREJ
AFUYEF	BOWCOG	NEBSIZ	DEWYOT	HEBKOR
AKUZIP	TEHPII	BIZYOZ	DIHMAJ	CUBTIE
CIRKAR	CODMAL	AFUKES	AJUTIJ	ACECAN
CIJTIA	BONPOL	CORONE04	YEGBAQ	COTRAE
CAZRIE	ACIJIF	ALEDIF	APINIY	CUYPOB
LEFLOA	BADRON	COLHES	BEJJIL	BAFPED11
AFIJUW	AROBUF	CUNSOT	BICROU	BIBCUL
APOTOP	CAQDUW	BACREF	DIDQOY	BAPXEY
BUFFAJ	BAHPAC	ARUDUM	DIHVUL	AGOLIT
BERFEJ10	BEMDUU	KECZOK	ACAXEI	BIDYEU
VEBVOQ	BUTFIH	DACLIF	CUCPOH	BOPWEJ
BAFTOT	ABEPUU	LEHMAP	BITROL	BOQTIK
BUPDUL	CAXSEZ	ABEJAV	CADDUI	DAFCES
AJURED	AFEBET	AYUSUJ	BUYPUH	GAZDIX
BECVIR	ACSKAT01	CAYJAQ	GEFJUZ	CIKTEW
DECZOA	AFASIK	DASDAD	AXEJUJ	COJKUJ
BESKAM	BOZHIJ	CUVPUG	CADMOK	ATEWEC
BERGOU	BEDZPN10	ADISIQ	BIXKEZ	GEGKEL
BTOLYL	VAQKAC01	BUWCOM	AXARIA	BATBAC
CIKQAP	LECGOS	DIFYAT	BUCTOJ	CIVWAH
BENZON10	BAYDAI	AMIFEH	ALASAH	MEHCUA
DAYBOX	CITKUN	CUVXOI	BUDVON	BRPVAM
BOZLIM	BUJJEX	TEHDIW	AYOPOU	COTDIA
BEFTUB	CIPSOK	AYOZAR	AYOJAZ	BOYCUO
PECKUG	DARTEW	COKKES	CEPPOC	CUXNAM

DIGLOW	ADIJOO	BZAMTZ	CEBJOL	CUHGIW
CERYII	BUVCAY	DASTOG	CITSAZ10	BUKNEC
AMUZUE	DAKCEZ	ATELAN	AXAREX	ATUKUV
CAMCIE	ABAGAN	BAQRET	DIHDUU	DAYLOF
CAWMAQ	CEFWUH	ATEQOG	CISPOL	ASTLLD
DAVQUO	CALCOJ	AFOFAE	AFUZEG	CEKCIE
AKOWUU	BOWSOV	CNAPAN	DIGMAJ	ANUYIT
BIDKEE	ABULEQ	DEYMUR	AGEVIT	MEDXOL
BOXQIO	ASUYES	AJOFUB	BAGKID10	CAMZIC
CIPNOE01	ADAJAR	AHATAF	BEWGUH	AFUXII
DACSAB	ZECSUY	ALAZAP	BIRKIW03	AGASEH
KAXFAT	AKUYIP	BUFMOF	CIWJUO	CAGHAU
CUNHUP	COSDAR01	AKAVIS	CIPWOO	COVGUR
ALUJIC	DATJOY	DIDGIG	DEBLII	ACANAC01
BAPROC	BAPBID10	DILSIC	BACCOY	COMGEQ
BZPPOS	CAYTIG	CUNTAH	ZEFQIN	CACWAF
DEYRUV	BAPZEZ	DABMIF	DABBIT	DEDCUN
TEHFEU	DETFUC	CUFCIR	DEVBIP	VAYRIZ
BOLDIR	CAGFUN	BIXSAD	DAXPAT10	AQOTAC
CIMHUB	AYUPOA	DACKIE	LEJTIG	YEFSOU
AYUMAJ	CAMDEC	CEMZIF	BEJZEV	JONXOZ01
BADMOJ	AHUPAW	DABLOJ	CERTAW	ABAWIM
CUDZOS	CASDEG	CORWOW	BMPPZA10	CUSHUU
AVAMAN	CIQFEO	CUVWAT	BATZIG	BAYDOW
AFALAW	AYEXEJ	CUVQIU	AJOGAJ	BOXZUL
BIKQIW	DILHOW	CERKOA02	BIZTIP	CISDUD
AQEDEH	CEDWIT	ALOFIS	BALXAQ	ARIFEN
YEHDIB	BAJTAJ	CUHGOC	BRTCBN	BUDMUJ
AXAHOY	AXIDER	DAGYES	ATULIL	DARBIK
DEGMOU	BUKYAI01	BESXUU	AMOSUS	COBMEM
BEWYUY02	DAVGAJ	BOLBEL	AMIFAD	BATKIR
CUXHIN	ASINEW	HECQIS	AHOPIW	BZANTC10
BOTLHD	DIHQUI	CECQEI	AZOPEL	ALEYOH
BUFJOB	COTSAF02	AMAZAR	DAYYIM	BEPGIO
CAHDAT	NEDCIL	AQANUB	CUPJOM	CUPWAN
ADYPNL01	CUYDOR	AKERUF	ABEKUN02	DAVLOE
DALJUY	AWUNIP	ARIGEP01	BIQCEJ	BOQSIJ
CONZEK01	DARCIJ	BZHTHP	ADICOG	AJOLAN
DADPOQ	DIHMOX	CETRAV	BAGWOW	LEJWEF
CUHJUL	BOVBUL	AZUPIV	BOVTAJ	KAHHUZ02
ARIMEU	CLMANT	DECMED	CINMAC	AQONUQ
CAXHIS	BZOYAC04	CAWMIY	CAFZIV	CAWHOZ
CUSXOF	ARASUI	CIZMII	AGUXAB	COQCIW
DEYNAP	CORSIL01	WEFXEN	BUCXII	BAZFUG

CEHQIQ01	BETSUO	HEFHEI	AJULUO	BICLEG
BERKEO	AGIYOG	CAMGII	CIHFOP	DAHKAB
AROMAV	BAVCOT	BANHOO01	CURJAB	AREQAR
MEHDEL	BUDXUV	BUVYUO	WEFJAV	ASOBER
BEXLAT	DEFQAJ	LEJWOP	ALUMUQ	ARUJEE
AVAZOM	CAXMOE	CEMGIM	BUYFIM	AYECAI
DABCOA	DAKXET	CITBIR	AXOJIH	DIGVIA
CIXWAJ	BADBOA	BEVHIV	CEFROW	CEZBOA
CADFAQ	BUTWAO	CEHLUY	CACRAC	ADUWIF
DEYSUW	ABAKIZ	ARADUT	CIYPUV	COPHAR
AHETAJ	AYUQAM	ASEDUZ	BESYEF	AFOBOO
YONSIE01	AYUZUQ	CUJWAH	AWODIB	AFOCAB
AYATOK	ASOQOO	BERBAB	BOKYUX	AYIMUQ
ACERAC	VEBTUU	AFIZAS01	COVNOQ	BACLCH
CEJVES	NAYMAE	BAMBID	BEZHUL	CIWYOW
ANINUI	BEPXUQ	ABUSAR	DEZJUO	CUCPUN
NECJAJ	BIYBIW	ARAGAC	CILNOC	AFITAM
DAPNEO	BECSAG	BARWID	DIFZIB01	CARSIA
ALICAZ	CIPZUW	CUBRAT	BIGSEP	AMEXOF
CEFBAR	BPSOME10	AHUFOY	DAZTIK	BAXXUU
DADTAF	DACXAJ	BUBQOF	ABOHUV	BEZGIW
CONXEK	DIHDON	BUCTAW	BEBFAS	ABODON
AFAKOJ	AMEMUB	DIKLIT	CULVOU	CARWAV
ADUMUI	CEDGEY	DEFCEZ	COQCOB	DEJYUM
BUMHEW	GEDQOY	WEDPON	CEPYED	AQABOK
BZIDMS	DARXAW	BETMUI	DEYDOB	ADITAJ
CIQFIS	BAXMAP	BAVXEE	LEDFIM	BOYDAV
CLPOPA	AYUTIY	DHCBAN	AHIGEF	CETRUQ03
BOTDOD	BOGXOK	VEJRIO	CISNOJ	AYUWEX
ANEJUZ	CPPHOS	CIWNUS	CUNHID	BILYEB
BOYDOJ	COGRUN	CEJDEC	CIHGUW	ABUVAX
BUWVAS	CEYKOG	BRPHAN	DEKKIQ	BUWVAR
CAMFII	CAJSOY	BIFBIC	CUHGUJ	BAKTUD
BERFOT	BEDHUP	DETGUF	BENZDC01	AFEQEJ
CEHFUS	DABBAM	CUZHOU01	BURQUA	BPHSUL02
BADSEF	MECWEZ	BABKAQ10	BOBHIL	CITTAB
AYIJEX	AYUFIK	CILPAQ	CURJOQ	ARUWEQ
DENCOO	TEGTAD	CAXSUS	ATUSAL	BAPYOI
CIPROK	CIDPOV	CAGXOX	CEQKAL	CIWJID02
BZTZAC01	COLQOL	BADPUR	COZJOR	HECDEB
CADMEA	COLJUK	CERKAM	BAMZUM	BEWLAR
AKATEM	COVNOS	CUXBEE	BOQPAA	CAJLII
CAKZIA	CAWXEF	AJOHOW03	BAKMAE	ACAZOV
CIDHON	BUDNIZ	BUXBON	BEBGOG	CIJZIE

BANWOE
CAZYUY
BONFAN
BUFCUB
AVEGOX
CERBEH
DELVEW
BTCDOD
AWOFEZ
AKUJEX
AFEQIN
BEZWAE
BERJIT
COBSAO
DACSIM
DAJKUW
BPCXIM
CUQBEW
BESREX
DIFVAQ
BEBMUR
ASUZAP
CUVVUM
DAJLIL
COMGUI
AZERII
AKUSUV
BOCNOX
ASUWIV
BAWSAT01
BUHNOH
LEFNUI
CIBDOG
CEBKID10
APERUJ
AKOWOO
DAWDOX
BETCEK
CLPCPN02
CODDAC
CAMGOP
BEGVIU
BAQTET
APAPEO

ABESIK
ACEFEV
ABEJOJ
CUMZEQ
DAVREZ
COCKEM
CUGMIB
VEGBAN
ASIWEF
BEBPON10
DADTEJ
COMWUW
AYUPUH
BZTRIP
CEBLEC
HECNOV
ANEMIR
CAQTOF
AHEVOY
ARIXEG
DEWLOH
AHUYIM
CAYZIO
DAXYUZ
CIHLEL
CUBZEF
YEGLAA
AYEDIR
CIRHUH
DAJJUV
CAWXEE
BAVREY
DIKCUV
DEXBEN
CAXZIL
BUZYUS
DIDCEZ
AYEQOK
AQAFOO
AXIDAM
AGULOF
CIBPUY
CAVZEE
CIPWII

HEJMAN
TEHNEC
BMCBIB
ANEHUX
ABOLUB
CURKIJ01
DEBMEF
CABRAA
CAHGOJ
DERFIQ
BOLNOH
CUGFEQ
BOVBIY
BOGZOO
AXAWAY
BIDQAH
CIRLIZ
AFIPOW
CADGAR
BOJLAP
BEPDIJ
TEGWOU
BUFLIY
QAZSAO
ALUXUA
COZXIA
BUCHOY
BITBOX
CIMROH
CACXUB
ATIWUV
ANUBIV
QEFBOV
AJEQAH
DETJIW
AKOWUS
CEDBUK
BOHCIL
BOLFIR
JASDAL
DANTEN12
COGREV
BOVVUE
BAZHUI

COUMAR12
AVOTOU
AXEJUK
CEYKEW
CICPEK
APUKUS
CIBCOF
BZTBZT
AZACUA
BIKNUE
COYRUD
ASONEC
BIHVIY
CARLEO
CIQDEN
CLPHUR
AMIXOJ
CIKQAQ
DECBUK
DELKUD
BMDAZC
CABBIR
CAYKUL
CUCSUP
BIGHUW
BASCON
ARANUE
BARVIA
ANTMET05
AXIGOE
CANZEY
DICZUK
CERZUV
AMAFAV
BINGOW
BAQGOS
BAFXEO
AMAJEF
ALAPIO
CUNPOR
CABJEW
BUDWUU02
DELSUJ
AKOVAZ

AZUZIE
CUXHEK
CUYZUT
AVOZAM
RAXFII
AKOZOQ
DAXWIL
CAKNEI
CACHAP
ANTHOB
AZUHAE
BEBMOL
COPBAK10
DAXPEZ01
CUNLEE
BIFPUD
CIWYEO
ABAHAP
CUBLER
CURQUD
CUTHAB
COSVEN
ATUPAH
HEJKEP
YAZCUA
BIRYIM
ABIVAL
BEYXOS
CERBEG
BUBBAC
PHBENZ02
AJOCOR
APOFES
BUVXUN
DEGXOE
AQUTAJ
ANISAZ05
CINSUP
BIZZER
CINMAC04
ASEGUC
CIQPUO
WAZSOI
ANTCEN20

ALOVUS01	AYUTOE	AYOTEO	ABOFON	DIDNEK
BOLZEH	AMUSOS	CEVXEH	BEJYOE	AFOLOY
BAFLAY	MAYHUS	AMETAN	BOVWEQ	CADBOY
BILRAR	HEHXEA	DEGVUG	AKOSIE	BECPUX
COJHEP	CASCEF	CAPQIV	BINDEI	BIHLOV
ATEKOA	BELVIY	NEHDAI	DAJJIJ	DIGZAW
CEKNOV	CADGET	BIMJIQ	BOQZUE	DAPTET
CUWZUR	DAMNUD	ASOVIO	CEBQAC	BIDZOF
AYEDAJ	QEDVUT	COSFOF	ADIJII	VEFYUD
CAXKIY	CAWHUE	BEDWUC	DICSAJ	DAWJET
BAVHIS	AMAJAB	ADOFAC	CIBFUQ	CORWAJ
AGUMOG	AKIZIE	BOQGAP	DEVXUX	DEBKIH
CAJDAV	BISNOI	CUGNID	AHEDIB	TEHHEW
DIGFEE	BINVIF	BINAPH11	AQILOC	BUFHAM
BIXKUP	AFIHII	CIHCUR02	CAPHIM	ADAVUX
DESNOE	BAFHAR	ANAZTP10	CEGGED	VECBIR
BIZFOG	AZIQIJ	AYOVUG01	CAFLAY	BITBIR
CUNRIN	DARVID	AMACPH01	APANBZ	COZTUI
AFUWOP	DEZRAD01	CODHUY01	BANNEL	ATAJUB
BADMAW	CBZYAN01	AYOXIX	AMODIQ	ABOSEP
BACVAF	AMEZOH	CATZUV	CEMJOT02	HEJJOY
AJAKAZ	DIFWAR	BOLLIZ	CIRMUN	COGQOG
AKAWOZ	ABANEY01	COMDUF	CEQNAN	CUCZOQ
MEJLOF	AVILOI02	DASVEY	BIXYOX	BATXOK
AXAGEL01	AXEDUC	BOBFIH	ARIVOM	DADHOG
CAJRAH	DEHCIF	DICHOM	AXARAS	COKPUO
BOMWIJ01	AYUROC	BOJNEV	COZHIJ	ANUWUC
LEJRAW	AFOCUU	CEBKOL	ARAYUO	VAZKIT
AJUMEZ	BIPYRL03	ADIJUU	ANUXIS	CORMOM
YAZQUO	DEGFEB	BOCZOI	BOTCOD	QEGZAG
BZDCPB13	AVAGAF	BURQOU	CDAGLN	DEVREB
CIPMET	ADAPUS	TEHGIZ	BEJXEU	ACIDIZ
APOZEL	CIPQUO	ADISUC	ATELES	ABAJIY
COMQUS	BUBNER	DIBDEY01	BUHCUE	BEVJET
DAQJIP	DECGIE	AQELEP	COMRUR	CAMGUV
CARXOL	APAPAK	ADULUG	COCHOS	CECYEQ
COLROM	BUNKEC	AHOVEY	LECQIW	SAXCAY
BUWWEW	CXAEZT10	AKIFUV	LECQOC	BROPSY
DAGTUB	CARZIF08	BAVGEM	BAJCOH	ANUVUC
BUWCIG	DANJOU	BAJXOZ	DAHLEG	CEKNUB
BERMES	COCGAD	AZOTUF	CEYGET	BOCJAE
BRHPQU	CIWHUM	BAZYOS	ADURIA	BILYIE
CYMINO02	APUZUG	DEDPEK	ANUSIM	BIYXUC
DADCOC	APEMAK	CIJGEJ	ANIVEA	HICFEE01

ARAJIM	CASREU	AVULOS	CIHVUM	CUHRUT
ASOQAB	AJOHOW02	DBANTH11	BICBOF	AMOXUX
COSVUD	ARANAK	BAWFIR	CENYAV	ACATUW
DAJWUG	AXANIY	CIPWED	COFDOS	CARHOS
CENSAQ	BAWGAI	GEDTER	BOQBEQ	CIHGIK
CIKVIB	AMUVUB	COGHOX	BATTAT	CASGEI01
CIQFOZ	ARASOB01	CUNCAP	BESSEZ	COQYOY
TAYPIV	COMHAN01	CEZGOF	AYIHEV	BUVYOI
DAJFUS	DIBENZ11	CPHACR	BOQBOY	AHEBUK
BOZROY	CUNPIL	CIDSEN	AWOBEV	AKEKUW
BAZYUW	COBZOK	CALFIE	BOXCEX	AMODAI
BOPJOG	ACBIPH	AWODOH	AHEJAZ09	BIJDOP
BAPZUP	ADULOA	CIBTAJ	CIQNEW	AJIXUM
BAYHOB	CUQKII	ATEZEE	BUZROD	AREJUE
CAFGOH	BICSOV	DICYAQ	ALUQUT	CIVTAE
CURPAI	CUXBII	CEVDAK02	CAWMOE	CUQCAU
ARATAQ01	YEHSIQ	BUZFEH	BOCDUS10	CAYZAG
BUYXIE	BABYEJ	CIHMOW	CUYSUM	BUNQIM
AWEVEF	ADEKOK	DAFBIX	AMOSIG	ABEKOK
CAQWID	ALEXIA	BAZOCT	ALONUL	AKIPUF
CUPJAY	ACSALA20	DECPUW	CIRSED	AGEHEB
HEFGAD	QAZSUI	DEDMAB	CUDQID	CAMMIM
CUQYES	CAMDUS	CIMRIB01	BAQDOO	BOLJOB
AJETIT	CIYFUN	NECYEC	BOXYIW	AJAJEC
DECNEE	DASHUD	AMIYEA	COGVEB	DACXAI
CARKIQ	BETNUK	ANIZON	CEMVEV	CIWFEU01
DAFMEE	BEDPUX	BETFAH	CARYOM	TEGWIO
BAMYUL	DALZIA	CAMVOE	CIVSUX	ARICEL
APIFOW	BICWOB	AMXBPM10	ADUHEM	BALCUP
CINGUB	DECUSN	HEDKIN	CUZKAL	BILVIB
BUSJEF	BNPERY02	CEPHEL	BAFKOK	AGAMEA01
BEXLIB	ALITOF	CIQREB	DEGGOM	QEFDAJ
CUBQEW	BOWGUP	COKJAO	ANOFUF	ANUXOY
LEGMES	BOKYIL	AMUJUP	DEJGEE	DACNOL
BICSEN	COWNIN	DCTXAN	APOXOT	BAQCAZ
ATEHIR	BAWTOL	DCLANT10	ADIRAH	BIFRIS
CIGTOD	DADBAN	DAWSOM	ACUQOG	AVEWIJ
AJULAS01	CAGKUQ	XEFKAX	AHIKUY	DEJGII03
BEPYIE01	ABUCAD	AZAFOY	DAYNOH	CUSMOU
DAFYEO	CUMVIP01	DAWHEO01	BADJOH	HEDFOO
AYUJUA	CEVKEV	COCBEC	CIYDIY	AFOFEI
BIBQAE	AZERON	BEGJOO	BIKLEO	LAYREL
DIHDII	DAZCAL	BALJUV	DAXCUD	BUVJEI
AHUQOK	AZOTIT	DAMPIR	COZYUM	DACXIQ

ASUJAA	DASDEH	CANDOM10	BIZXAK	CEBGUL
CASGOS	CODDUW	CUCHUF	ACOGEG	CENRIX
CORNUT	AXARUM	ABOQUD	CANTAO	ADOYAT
ACEQEG	AKOZUW	BEBXUB	AKATIQ	BTRYPT
BOLMIZ	BILDAD	DEXYOV	AWAHOX	AMATOX
AYEGEQ	ANPHXN	CIHHUW	BRFUSO02	BEBDET
BENZID13	CUVJIO	CIMZAB	DICRAI	CIHLOV
CUCDAF	BUCKUH	AZEJEU	CAWHOY	DADTEH04
CEPNIV	ADOTUI	BAGXOX	BAXNAQ	DICFOK
DEDCEX	AXANEU02	ACUYAA	QAYHUW	ACOKUA
CAPZOL	BILCUU12	BARKAH	BAFYOZ	BIGBEY
DAXSOM01	GEHNOZ	CIYSUZ	APEZEB	CEPFAG
COYPIP01	AWUFEF	LECWIC	CAYTOM	CIRLUL
BIBSUB	COXCAT	CUWCAA	COVDOI	DEHXIY
DAMPEO	WEBGUI	QEDVED	ASUZUK	BASJAI
COVPIM	BUCKOZ	PECDIN	AYUKEL	DAQNOZ
GAYZUE	AXEHER	DETGOZ01	BHXPAM10	CEMZEB
AGIPAJ	CIPRUQ	NEBXUQ	DABVAD01	BORLEA
CANXAR	HEHJEM	DIKTAS	AYATEA	BBZOCO
COFYON	ANISIC03	DABLAV	ATEVOL	BAXHEO
AQEBAB	CONPEB	BAWVAZ	AXUDED05	BUVKEK
BIFQIS	QEDTIF	DANTEN01	ABIBIY	BAKCUM
AQUCEV	DACBEQ	ADODUT	CEXNOJ	BASMAL
BUBLIT01	CUSWIY	AZUKEM	NEGJIV	DAGJOK
LEJRIE	AROBOZ	COZCUQ	DEPRUM	BOLGOZ
AXAMES	BERDOT	BOKPEX	BICZET	TEHGEV
DAKZAQ	BARVEV	BAKYUK	DIBENZ14	ADAKOG
BOPRAZ	AWULEK	DAHBAP	ADUQOG	CAYXEI
CIPFAJ	BOMSIH	BEJZEV11	CUWZOL	AQANEL
CAWDUB	COVPEK	AMUGAS	ACEPII	AVUTOB
CUNJEB	AZEQUT	ACORUH	TAXRIW	BOWVIT
ABUSEV	BODFOR	BATKIR01	DAFFIZ01	BUWBIF
CUXXAW	ANIVID	BEDQEI	BAVDEJ	CEDPEI
ASOXUC	DAQVUM	CUNTUA	DERVIG	GECTUG
CINNDS01	DIJLIT	ANIXUR	BINAPH12	DAXXEI
CEYQEC	CORWAI	ATOPOP	BEVROJ	BUFQOI
CABYEK	AVAFIM	BOQTAC	BZTETO	BOYHED
ACSLCB10	BDPPRP	CUQHAX	BOFKIR	CEPGEK
CARXOI	ABAGOB	DERHIR	AZUJIP	BARKOX
BESKEP01	AXONIM	ABABEL	CUJMOJ	QEDYAC
BADVEH01	CAFPEG01	BUBSAT	DEGDAV01	AZOQEM
ASECEI	BASMAJ	BUMWIP10	COYCUQ	AYERUR
DEBYEO	BEPZEB01	AZAFEN	BEYFER	AZUYUR
CESPIB	CINXAZ	ATUJOP	DANTEN13	DEGCEY

CUCTIE
BARNUE01
AXOWAM
VEFNEC
CAHDEW
QECLIW
CARWOK
CIYDUK
ASOHUN
AWAZAB
COCHUY
CAQZOL
WEDPIH
BAGVOV
ABABOV
BAQBIG
ADUXED
AHUMEW
DEYREG
BOHTAT
ASOTAE
DEKQOC
HAZDUK
AREROE
AGUREZ
CEDPOS
CAMHAC
BAJSUC
CFPZBZ
HECLAF
ACAGIX
WEBZEL
BAFYEM10
CEBPAA10
AXOXUH
BAHMIG
DATHUE
BUYZOM
QAZYIC
AHACUJ
ARERAQ
WEDPED
CAFGOF
QAYJIM

BOZDIE
AMEXEX
BMBZCH
CAQKEN
BADJEX
DATMAO
ALUVOS
AHUNEX
AGUZUZ
CIYRUY
BUVNIQ
CAHHID
BEKBAV
CEWYIN
CAZSAX
BIKXID
BIXVIP
CURSOZ
BAMTEO
AWATUO
CBENPH02
AZPCYM
DEKMUC
TEHCOB
CASGAF
BOCZAV
ACIYIX
CAHXAM
APELAJ
ANIBAC
AJEGOM
BUVBEZ
DASNOA
AWOQUA
CIPVII
BEGBAR
DAZKAT
BRACPH02
ALABOE
CPCPRB
DABVUX
COWXAP
DERSOJ
CEBJUR

AZOMEI
APUDOF
CUQGOK
DAYJEV
ADABOX
BAVYOP
AQEFAE
DEJBUP
AHAVAI
DEMTUL
WAZHIR
DAFBOD
DIMNAP02
CEHRUD
BAXCOT
DAQHUY
VEJQAF
CUZXUQ
BUMHUM
ANUYOZ
WAZMUI
CAGDOG
CIDKEH
ABEFAQ
CHBZOA10
AGEBUL
CARNOA
CEYJAR
VEGBER
BEBBUH
BAQCED
CUQCAU01
BODKOW
OHOCEV
ATAMEO
ADELUS
ALAHEA
CIKSIZ
AYUVOH
CETDUB
AVUXAS
MAZREN
CAPBIG
BENLUD

CUQFIE
AMUBIV
BANZIA
DIGGAD
ATELER
CIZRAF
DEBXAL
DASJOX
CEFSEN
CUWQUI
AKUQIH
AKOFOW
AKESOA
CEPPIX
CISMEX
DAPNIS
AGURIF
ATIQUR
BILJOX
CUWCEE
BEPZIH
ANISAZ04
CEPZAZ
DIDJOQ
ASOTEH
TEHBAM
CEFSAJ
ATUGAX
BAXBEI
CIMWOL
AJUQIG
CAJQAG
CUXYUR
BUQPEK
CARREU
DAFGIA
DEWZEM
CICHEB
ACEFIZ
AKIPUH
CUYHEL
BORZOY
DAKDAX
BONXUZ

BEYTUU
APAWEV
BUBHIP
CIYLAY
CUFQAW
CARBIJ01
BUXGAD
DASPUL
DIDJEG
ARUBEV
BZAPRM10
CMPDZB
YEJPIP
AMHCIN
ASOFOD
AYUBIG
UQUYIQ01
ABOJAD
CIVWUB
ALORIE
DAMLEL
CAVGEN
AGAKIE
COMSEE
BAVXAA
ACRDIN06
BZTROP10
BAHROS
CUFRIF
CODDOQ
ARUGEB
BZILBY
GEBHED
CELCIF
COWNEJ01
AXUDED02
CASMIT
ANIROG
DELKEL
BOPSUW
CEWYEK
AGATIN
COYJAB10
COZTIW

AQUBEV	DIDRUD	CIQDUB	DATMIW	AXADOT
CEZCEQ	DACGOE	CIPTIF	AWUXAT	BZCPTK01
ARABOK	DAVJOC	BOGZIH	BYITOT	CONCAL
AGAVUB	BEWNEY	BUNROT	AZEYIP	ANEWEW
CUQXER	CUCKES	CEXKUM01	AXUSET	AZIJOK
BAGXEN	AFOFAC	BAQROD	DBZOSS05	AFIPUC
DASPEV	CAPDIJ	DIGXOH	BEPJEN	BURMOS
DBNTHR01	AJUMOJ	BECYIT	QEDKAO	BAFLOL
DAJGOL	AZITEK	ABIJUT	DECCAR	BELZEY
BALCAS	BUGPUP	ALUKOH	AROHUM	ANIFOU
BAPALC10	CETRUQ02	DASPOF	BAKWAL10	DIMQUN
COJKET	BIRFOX	BOCWEW	BAKRIQ	AVAKOX
BAKWOA	CUVXEY	CIRDIR	ANICEH	DANTRO02
CAJWIW	APELIR	DAJMEH	COVYES01	AHIDOM
COSXAL	DIBENZ04	ASUMOR	AMOBIN	YAZXAB
CENBON	DANKIN	BAGTUZ	DEBQEJ	CPHPPS
DETZUX03	CEDVEO	COXJAC	MODDOA02	BAVTIE
CUNQAE	AVIWIN	BOYREM	AJEDOI	COTROT
QECNOE	AXAXON	CUWBOM	AXAMIW	BIHYEW10
DEMSUK	BAYCUB	DAVWUU	DAVFEN	BOYDAV02
ADIBIA	COCJAG	QAYHOQ	AVIYAF	CARYOK
BYIMVX	COTQAE	BAPRAN	ARAZIC	DELWUN
BUFWEE	LEGCUY	BABMAS01	BUDYOP01	CANCEA
DAMZIA	CAFGEV	BOMRIG	CHRTCQ01	CUMHEY
BUSQOV	DADDIY	ABUNOA	CAKZUJ	FECHAY01
COTGUP	BRBZNT03	ANETUJ	FINHIU02	DAJKAC
DIFMEL	BUCJUG	BAYNOH	ABOWET	DIKREU
COBNUD	BEVQUQ	BANPAI	ABOBEB	BEWBUC
BAWNUL	ABOVAP	BIHMIQ	HAZSOT	CAYGUF
YEGQAF	CEGBOH	AWEROJ	APEYUQ	ATERAS
ALOHAM	AWOXEP	CUVTOC	BAKTEN	AGUXEF
AXURES	AREFIO	ARUVAK	BERDOS	CIQMAR
BARVIA02	APIPOG	AREPIY	COBJAG	BEZHIZ
DADBIV	BEZLEZ	DADSOT	CUMKAY	APUWAJ
DAXLIA	ATECAF	DICXAO	CEGSAI	WEJGOK
BEQCEH	CAYXUY	BAWFUD	BPTPCA01	BUJTAC
DASBII	DAYXOR	DEHBEA	DBNTHR02	CESXAZ
WEHHOJ	HEDHIK	DHTRBP	BISMIB	CEWYUA
COTJOL	CEJJAD	BERXIG	COFZUU	CACYAJ
AZEYEL	BUNYIT	AGAXUD	MEDNOB	DIFXIB
AFESIO	CERKEQ	ACITOX	BUKTUY	BIRKOC01
CIVRUU01	BOXTIT01	CARLOY	HEJJIS	AMOQIE
DEGMOS	DIBENZ15	WEDGOE	BIXGOF	CUBJIT
CABCEQ	DICHON	CEGXIX	HEHWOJ	BADQEC

BINPOD
CHBZOB20
AKOTIF
AZEXIO
DIBVER
BEYFAO
DAQJOX
BIJLOX
ASOHAR
DADWAJ
APOXUZ
TEFMUP
CUJNAX
BZCPTJ10
CIBFOK
AGAYAK
ASOREF
BUWSAP
DELDII
CIXVUC
ARIXAC
SAWSUH
CUBJEP
CEBVOW
AXOQIP
CILLUE
AXABAD
DAZQIH
CEGDEZ
CAFWUE
COTYUF01
HEDQEP
COCMOX
AWAPOD
AVOBIX
DEZSOQ
CANMOV
ACEXOW
BIHPOZ
CUJPIG
BANMAE
CIGSOC
ACOBEB
AZOSUL01
CEJFIH
COVXEQ
NEJGUH
DIDKEI
BAWGIQ
AKOYOO
APAKAF
AXEQEA
WAYQIZ
BANWEV
HEHZEC
CUTZUM
DAYTUV
AWUTOD
ASAKOU
DALJOS
BUCVAW
AFEBIY01
BEKVIW
CLMIND
AZOVAN
BAHDIY
VEBRUS
DERBOS
BZDCPB12
COUMAR02
CAFTIO
AZIBIV
AHASOS
ASIZUY
DERXAA
DIJZON
AVIYIP
BOLMEV
BAQYUQ
DAVFOW
AVEPIC
BAHDOF
BONXAF
ABUCUV
ABULIU
DATTUO
AYUTAQ
BUTCEZ
BAKXOD
APAXAR
BEZHAR
AYUHOT
DIDGUS
BEJLIM
CAJSUE
DALTIT
BEMXTZ10
AGOTIB
CIWYEN
ARAYID
COVCAT
BAPGUW
CACVUX
OFOJOI01
CEJHIJ
NAYFUR
ATIPOI
BOLQAW
ADAFEQ
DIGTOD
AXENAU
DATMEQ
VEBWUX
BAZGEO
TEHTAE
AZABIN
BAGXAJ
ZAXVOM
VEBPOK
NEDQIZ
BIMSIZ01
VEHVEM
BEHROX
CIZPIL
ADIGUR
BUCKAN
BUPPOR
AFIBIB
DAYJAO
ATAVUN
AVUZOI
CANNOW
LEFLEQ
BONZUZ
HEKLIV
TEBRIE
CETKUJ
AYUWOH
AYEPOL
BEZBIT
AJUFIV
CITSEE
CAFTEL
AJEGUS
BALFAW
ARABUQ
CEJTIU
AHAYOY
CEQGEL
BAYJUJ
DAFPEI
BANZUM
AKEKIK
QEDJER
CICJAZ
ASOHOF
DEWCEO
BUWYEZ
CUHRII
BIKDAC
CEDRIN
AYOYUJ
CIVTUY
DILDAF
AJUMIC
AJUDIT
COQQIK
BICJOO
AJUXUZ
BEFJUT
DEBVOX
BEGNEI
DEMFUX
CUJMIE
DICPAI
PBPACB03
DICFOK10
DARVUN
BAFWOU
BEGTUC
CAGLAX
AWEMOF
BEMZEZ
BEGTUE
BALQAI
CURYEU
ARICAH
GEGKOV
AJEDIC
DADRAD
AWEKAP
ATUQAJ
DEHYUN
DIGGIL
CUVJUZ01
CUMQIK
BENZID12
BECNII
YEBXUB
ABUTIA01
ADOKEJ
CIQZAD
COJMET
CORPII
BILQUK
CUPNIM
ABOVOE
CUKYUC
CIKKUE
CEKVIX
BAPWAS
BUGJIW
VEDSUV
COTCAR
NAYGEC
CILTAT
WAZXUT
BAGXUC
XEDVUA
COHHAI

BIXPON10	BEPHSI	CAZZUZ	QAZGOQ	CAJFAX
ATIDAJ	ADELOL	AMEQIT	AKULUO	BARYIC
CEJMEK	NAZYAR	DAWSAV	CANLOS	CUSSUG
DIDPUC	CNPHAN	DAYLIZ	CAGNUV	APOWEI
CEGSOY	COQQUW	BOJFOW	BAMBOJ	BOGFOT
DIFFEE	CEKDOL	DEPGEK	ABUHUD	BIWFAO
ABUBIJ	VAYZAZ	AQAMOW	BUZSOF	CEYJIZ
BITDAL	CEXKIB	DAVBIM	AVOTUB	DETZEH
TECGEQ	BUDJUG	CEVPEZ	BUCNEU	QEDQUO
DILDOR	ANIYEC	DEDZUH	DAKLEJ	CIQRID
BIHXAS	DALDAY	DCDPCP	BANNUA	COKLIZ
AJEDOK	BUTDOJ	BUJVOS	ACETUZ	CIZDUM
BUXMOX	VEGZUF	CERHOX	AVAWEB	BILTAR
ABALUM	BASPEQ	BASNOA	BEJBEZ	AFOLEO
BXBFUR	CEGYEU	DAWCIQ	BUMBOA	BAVQEX
DIJTIA	BUVXEX	CUKDUI	COBVIZ	CEWMOI
BURMUY	VEGQEG	ABAHAO	BUNTUB	ANUQUX
CAQZUR	AFANAW	AYANEU	COYHAB	BIYPUW
ABESOS	BAGTOU	AFOHOU	BENRIX	CALZAR
BICKOP	CENSIY	COFHAI	BUGPEY	RASZAP
CEWZUB	AZISAD	BAVXUU	DEYGUL	ACSALA19
BAGRUX	CMXPXZ	ANTPYR	NAYLAD	DAKQOX
CADKAW	APUGUN	NAZTIU	ALAFOI	AFIZIA
COXLEG10	ANDRAN	COMHIV	ASEVAX	BEPPAO
DECFAT	DELJUC	CAWFAJ	AZAHUG	CIDCAV
CUSNIO	GEGKIP	APOCOY	ACOGIK	ABELEZ
DAFVOW	BATLIS	ACENAP04	HAZSUZ	CAQSUJ
CEHWUI	AJIBOL	BUYDAA	AKUSAA	BHNAPO
BOWWEQ	BAHGAU	BEFFEX02	CABSEF	BORREM10
CUTCUQ	AGONOA	BUVFUV	CUNDIA	CEFHUQ
CIQXIL	DAFREK	DAXQUR	ANQDAZ	ALOKAP
BOPQUS	CEGVER	COYQIS	AWABAD	MEHVUT
APUVUC	ATAYAV	AJESEO	COSQUX	AGAWEM
CUFHIW	CURJEF	BEVJIX	CIHDUT	YEJPOV
AJUQOM	BIPHME	CAFXIT	BEBBOC	COFGAG
QEDXUV	CUBWAZ	ACUPAR	TEHPAA	BENZID07
BADSIJ	JASTIJ	CIPGIR	DEBLUR	DIMNAP03
DIBENZ12	WEGCUJ	DERJAM	MEGRAU	CNANTH01
WEDQAA	AYUWAS	BOJVAX	DAXRUR01	BEFSAG
BEZXIP	BAVCAF	BECRAE	CEPPOD	CUCBUX
DEWGUH	BAXVAA	AJEGIH	DADNUR	AQABOJ
CETHEP	CAQBAA	DAQJAJ	DABHIX	ABIZUJ
ARAWIB	ATEQUM	BEXKAS	AKIXUN	AVILEW
BIFVUI	AZEHUI	BEQBEF	CLPXAD01	COQDER01

NEDZEE
BUDXED
COKYEH
AXUDED03
APIBEH
BUNKIF
BANSOZ
AWIRIJ
TEHDOC
BIWWUZ
DAVVEC
CUHBIS
DAJZAP
COSFUN
GAYXOW
AVUDEB
BOVVUF
AZANUM
AMUHOG
AJAXUE
DEMYEZ02
DAXSOM02
BOWPEJ
BOCCIH
BAKWUF
AYEXIN
AGIROZ
CNDPEU10
CUGWEI
CANDEB01
DELQAN
BUPDUN
CAMGAY
CIJYAX
AFITIU
CIXHOI
TEBSUR
DABBEQ
BALXET
ASASOD
BIGLIM
BOPBIR
AJEZOF
AWERUP

CABGUI
AMOCIQ
AJEXOE
BAJRAH
COCFAC
QESNIM01
CIBPEI
VEDDEQ
CAKBAU
BOSYAL
DANDUT
DEFGEA
DIGMEL
CAFFUM
BUXLOW
AWAVAW
AVARIZ
ATUJIJ
AJEXIY
BAXBOS
AFUSOK
APUREI01
DEMBAZ
AWUQIU
BUDPEW01
APALOU
TECXAD
BANDIF01
BEWYUY01
ANTCEN17
CABTAC
BAWWOO
AJANAC
CADBAN
DAGWOY
ASIZAF
CEGQUC
ACENAP03
CANPAK
GUHCOB02
DABLID
CUPXIV
CAPISQ
BENTIZ

DABCUG
AXUDED08
ASIDIP
AJOROH
DIBCIC
ANOXIM
DEXWAG
CONXUZ
CUXYIE
COJYUV
CUWGEI
BUPPAD
DANNAH
DCPZCO10
COFSUL10
GAZXOX
AMOHAN
HEFCAZ
AGOBOP
BOJVOL
AXALAN
BAVCUZ
QEDRAV
AXELIZ
CEWKAQ
CICJIH
AFUJUH
BABVIM
ABEXOX
BUXQIU
AKAYIW
ACOWIZ
ARORAB
DAGXIV
BEZHEV
CAZJOF
AGOPOD
AMUCIW
CEQCOR
AZAMOE
DATXON
AGEHUR
DEMBON
ARAZID

DEMYEZ03
CUNJIG
APUYIT
BOMDAJ
COSRIM
CEWMUO
CALDOL
COCXID
DAZJOF
DEJWUM
DADTOU
ABILOO
BUWDED
ANAZUM
BEFCEU
COCJEJ
BAGJAU10
TEHFAQ
DERVUS
DATJOA
AXAVUR
BACSUT
DIBZUK
COTLAA
CUSHAA
CUMHOI
CIPQOI
BAPQOY
CAVMOB
CMBTDZ
CUWDIH
ANIXIG
BUXWOI
BEJDUP
ADICIB
AQEDEG
BUNVOX
DIDGAY
AWOMIK
BAXPOI
APERUK
AFADEQ
ANOYAE
BOTCUJ

AXIHAR
DHXANT10
ALOLAQ
DEJSAM
AVUNUB
BOPGAQ
CUMQUX
AYUQER
CAYNIB
AVEGIS
MEHRAV
BAWMEU
ATODOD
AFUNOG
BEDVIP01
BINXIH
ACOCAY
ATAMUE
CIVRUU10
AHUSIF
ANUMAZ
BITVIJ
BACPEB
AMEZEZ
AXUDED06
BOWTEO
AYASOI
AVEREY
CIWYAJ
CORWOV10
CONQAY
ALOGOZ
BODHAE
BAFFOF
CIFNOV
CEZCIU
AKERAK
CIMQEW
CEMBUT
AYUYOJ
AZEGUH
BAHDOD
BUJRUT01
BZHYDX01

CEDPOR	AZEYUB	DAKRUF	CIPQID	DAXTIH01
QECTUQ	AJOJEP	CEWHES	AXOQUA	DADNEB
BENTUJ	TEGYUC	AKERUE01	ACETOT	COMQUQ
FOCYOM01	COWKUW	ATIXOS	BUCKIV	BIVWEJ
CACKOJ03	CEBGOF04	DAVQUN	COTXUE	CEWYUZ
DATGOV	BAQYAW	AQERAQ	CEGCAS01	BOWGOJ
AWELIY	WEGWAJ	ATACON	BOKZAE	BEXLOF
AHIQUD	CANWUK	QEJMUQ	CEYKOG01	BINGUC
DANCIG	DARYON	BAMLAF	BALVIW	CALWOD
BUDJIV	DBONAP	YEBMAW	DEXWUA	LEJZUY
DACWUC	BQUINL	CAZWEF	ACUDEI01	ALONAQ
AFOMOX	CUNDAR	ASONED	BESFUB	BABMUP
CENCUU	BENMIS	AGELED	ALAGUR	TEHNOM
ALIMEN	CIWYAK	CUFHUH	ACEYIR	AJOMOC
AZOJOO	AKOSAW	CABQUT	CAVHOZ	CISGUI
BENZEB	CEKDIH	AVILAS	BUTSEO01	ASONAZ
JAHQER01	DIBLOP	COBYOJ	BEGZIX	DEJMUC
BEGTOW	BUKMUR	AYIRIJ	DIBBUN	AXEPID
DEHCIE	QEBZAB	ABOBAX	ADAMOI	BUGTII
DAKXOD	CILTUO	DAFKAY	BEQKEP	ACEPEF
CEFSOV	BOHROH	HEGKIQ	CIYTOU	BUJXEK
BEFZUI	CUHSAA	AMDPYR10	MEDQAQ	CUQYAO
BOTTUA03	CAMJUW	ADIWOZ	BEJTAM	CIGPUD
MEGCOT	BUHQOK	GAZCES	BIQCAF	AHAPEG
BEXLUL02	DEWZIP	CAKMEI	AXEFOZ	ASEFAF
DAWZOS	BAHRUY	AHEDEY	AYAGUE	ACUHAH
DENYUQ	AWUGII	CEMHUZ	ABAQOL	BMBZDZ
DIGPAK	TEGVAF01	NEBNAM	BENZIL03	ANEWUM
CUHHET	AJEFAW	AVAMAM	ATEGOW	AGELUV
AJOSIC	CIKXOK	AQIQOI	BUJWAG	APUXAK
ABEKIB	CIJWEX	MECXEA	AGALIF	AVEWIH
BELQEQ	DADDER	AYUVUN	AVEHOA	DAXMIB
AKUXAG	AGEWOA	BESHUE01	AHUXOR	AKEYOF
BOPNOJ10	CEMJOT01	ABUNIU01	BOVSUC	BENZID14
CIJLAK	COZLEK	AGAJOI	BOLYUW	DEWSOO
CIRNAU	AMEXUL	AYORIQ	CARYIE	HEKRAT
CEWYAF	CUKGOE	AGERAH	COPVEK	DESMOD
BRANIL	DEMCEE	BIDWIU	LECCOO	AYOCIA
BATNIW	TEHNAY	DAXBIQ	DARXEC	COXBEW
HEGWAU	DASJIT	AGISUG	ASEWIF	BONJIY
BONSEE	CIMBAD	ACEBIT	CIQGOZ	DATGUB
COXKAB	BAWXED	BUFQEY	TEFBOY	BAPYUO
BUDXUU	BURLAC01	BIKBIG	CRYSEN02	DALHED
COMSOO	BEYNID	CASZII	ACEWAH	CATWEC

BUWGOR	BURFUR	AXUMEN	BALFID	BECPEH
BAWYUU	DETSEB	AHUFEP	BRBZET10	AVILOG
BPHENO03	IMEMUI01	CLPHCA	AMOQOK	CUNPUX
BTPMET	BUKMIF	CBOZET	CUZWAX	AFIFOL
AVULAE	BAZVEE	LEJSEB	ABIWIU	BOVWIU
ARAZUP	BULLUP	CIRHAN	DABGIW	DBEZPO
COPKIB	CAXRAX	DARYUR	DECCIY	DAYVAD
AFIJIJ	CEHYUL	CIWLUQ	COQRAD	AROFOC
CUCLAP	ABUMOZ05	DERFEM	DAMWOE	CIBPIM
DAQLAL	BIYSUY	AJEVOB01	BOZXOE	BOHBIL
CADFAO	CURREO	CEWGUG	AJOSID	ARUWIU
ASIKOC	AQABUQ	ASEPOE	AQOFIV	AHILIN
APUCOE	BAWDAF	ASOYOX	ALODUC	DAZKUN
ADUWAY	ACONUD	BERWEA	BEDFUL	AZIWEN
AFAZIS	ACINDD	DIGWOG	COZTOC	APAPYR
BEFQEI	CEXQUT	CEBBAN	ARAZOK	APUGUO
CIWCIW	BRPCYQ01	BINDPY	DEZKAV	BIHWIZ
BIDREN	AXEMIZ	TEBPOI	SAWTIW	AGASUY
ALUQIH	DAVCOS	BZCPTG10	BUDJEP	BECRIM
BUJBEN01	WEDJOH	COMCAI	AZOXIX	DEPXOL
BOHCEH	BACVUX	BAJJOL10	ADUQUM	CAGKEC
NEGZAD	AWEKUJ	GAZBAN	BAPRER	CURFOL
CEHGUT	DAZYIP	BEFVEN	AHUFIS	AMPTOP10
BONTUV	CAMPIR	AJAROU	ASAXEX	NEHDOW
COJWEF	CUNHOJ	ANIJUE01	ANUBAN	DEJMEN
CUZQIZ	CUBMIW	NECGUA	BIKCIJ	ASITAY
DIHNUD10	DAJTEN	BAVHOY	CIGPEN01	BEZGOE01
CADXAI	CUBXOO	XEDBEQ	DEGVUI	CUZXOL
BUXBUT	DADVEJ	CUYYUS	ASEPUJ	TEBPAU
BEMZID	HECNIP	BOWGAV	AKOJAN	ACEYOY
CIWXIR	BZOYAC03	DEWCAK01	ATOFAR	KEFFIN
BOQXUC	ACAYEL	AYEFAL01	ACAYIP	CADPAA
ARAGOQ	AXEMOF	APESIY	DADDER04	CIZQAE
AGARAD	AXOSIR	ALACEV01	JAWBAN	ACUMEQ
CASHUC	DICGED	BOTTUA01	BOCYEZ	CODHAE
BUPPOT	DECFAS	COCKEK	BERHEM	BEHZOF01
CEFPUA	COPLEA	CUNQAF	AGICEA	BESLOC
BALDUP	DIFWEV	BETTID	CEGLOR	AHETOW
CUVSET	ABICIZ	CERPAR	AJOHOW05	ADEDOD
CEMRAO	CADZOZ	BAGQOP	COZSIV	CIHHEG
AROQIJ	VEJKUT	TAYQES	ABOFAB	TEHPUU
BAGXEO	DATDIO	AFIGAZ	CAQVEW	AMATAL
AGINEL	CNCHAL	QEGCUD	VEDGIX	AMOWIK
AREVAW	ATIWIL	SAYCUT	CIJXUO	BAGLED

CAWGAK
AGATAF
ALEKEJ
CODDEG
DIBENZ05
DBANQU
WEGVUC
TEHHIA
AXUNIR
CIWBIT10
AQEQOE
BEGLAA01
DAVBOT
DENWAU
ASOLAX
DEBHEA
NEBXOK
CIPWAB
BAGGUL
COBPAL
COFQIY
TEGZOX
BITJOD
CUSLUX
CACDOA
DESSAV
AGANIH
ACOTUI
ACOVUK
CBXPCP10
BZCDOD
ASUQAG
CALDOK
CUDWED
AKOWEE
ADAXOT
CETQOJ
BUYGAF
COYNAF
PAZHUW
CATMOB
DIMNAP01
DACQII
CESVIF10
BOQVUY
AJIKIN
CIKLEP01
ACIDEV03
BFUOXF10
BETCAG
AFURCM
BIMYIH
COCQAM
COXGED
BOBMEL
CEFSUD
ABAVIL
COGYAA
DAGTIP
AYEYOU
AZONAE
CAWJES
DAVTAY
AWECAG
DANCOM
VEDKAT
CIBZIW
DARZUU
BATREU
CAWHIT01
BOSDUJ
BUBYEC
COLQUQ
BUGQEA
AFOKUB
DARVUQ
AZABAG
AZUBON
DAKYOD
BIZZOB
AXASUP
BEMHAC
ABOFIJ
CASHAH
ASORUV
BOVYAO
CAGKAZ
DEXXAG01
CUMPEH
DEBGUP
AFININ
AXUDED07
DEKRUJ
BAXXEE
FEGBAX
AQILER
AQACIG
NAZBUO
AHOXIF
CEXVOQ
BAPWUM
CUZQET
AJUZIQ
CIRPOK
ATIJEU
ASIJIV01
CAPDAB
BACTOQ
WEGWIR
ATATIZ
BITNAT10
ABUVEY
BAXBIM
BAVHUB
BUJGUK
AKUHAR
DIBPUZ
DIBZOE
AFAXAH
AVUHEG
CUSTAN
DANKAH
BUJTEG
CUMVAJ01
DAKPAG
CORPIJ
DECPIN
AVEHIU
BAFYEP
CENXUP
APALAG
DAFCEU
ATEKEQ
AFERIN
DADCAM
DABHET
DIBDUO
ASUKIJ
DENHIN
BIPHME01
BOQHAR
CORGUM
BEQGUB
BUQWUF
BEGVAM
BTCRDX
DEPMIV
ATEHOX
DAVFAH
CAMGEF
CUPYIW
CAQXAV
BEBCIU
ATOZOY01
BOPDER
DEGCIC
QECRIC
CAPXAT
BEXMUM
DAKYOE02
AFOJIQ
BOGZUU
NAZDIE
ABALEX
BIGXOG
AWIWAG
ACEQUV
DEDDOF
BAQQOC
CAQLEO
DEBLOO
CUNNEG
CAWNET
BUWRIW
DEXTIK
ANEDII
BESNAO01
BONJUJ
CIRLEV
AVIBEN
QEGQOL
BEQWAX
AXEBOW
ACGLPR
CURCAV
CIVYUD
DEXQIG
ALACIA
BIMSEW
AGOLAJ
ATOMIG
DADPDS01
CEJVUI
DIMGOV
HAZXIS
CEZTUY
DEBHIB
COFQEU
CEGCEY
BPHENO19
BAPNAH
HECKUY
ADILAB
CUSKOS
BODMIQ01
CEFXES
AVIFUI
CADDIW
AWUVAR
BAYDAH
AYUZAW
COJXIK
CBENPH01
ACOSET
CARVAV
CUFWAB
COBFEF
AHUFIT
CUYXIF
AWABUX

CUVLUB	CALTEQ	COBJEK	AZESTD	APERET
AGASIM	CIQSAW	CIQCUC	AXEPUP	BOVTOX
BUWVIZ	AXOLAB	CAFMUU	AKOVED	DIGDUS
BOTROR	QEGLUM	CADJEY	AJOCEJ	CEHBAS
COPLUQ	DAMMAH	CIMZEF	ABUMIV	BISREA
CUWLUC	CUKDES	AJOHOW01	BONQUS	CEGFOL
AWUNEL	BUMCIV	ASULIK	DIGSUH	BASNOZ
QEBREX	ABOWIX	DAGJUQ	AMOXEG	BADNAX
ATUDUQ	MEFZOP	CIZGOH	BZPPOS01	DAFJAV
AFIPIP	COGVUR	AKUREF	COWNUZ	AWIVEH
DIJCOO	ABUSOH	BAWFIP	AYANAQ	AZENOK
CILMAN	ZEBDUI	CIQHIU	ANUVIP	ACULIV
BIQZOS	CEPZON	BABCIQ	ADUTIE	DEFGAW
AXUDED01	COGWUQ	CEGBIB	ACETUY	AKELEI
DALGON02	BABLIC	DASJAL	AQEQUJ	CAQBON
CAMGIH	CASDEI	DAZJEV	DIKGIO	CIWJEX10
DEXHEU	APEKOX	CAMNOV	BAXMUJ	YEKPAI
BIQCIN	BYITOT02	BUNHAV	CEWRED	AKOQUO
CEDVUC	BULBEP	DAYVIK	BMBZTP	CEWWOR
DADCOD	CXPTDZ	DETHAZ	AHEFEA	ALAFIC
CUNTIO	HEHTIA	AFUSAX	BAPZID	BULQOQ
AGEKAA	AKUZAH	CAMZOI	DEZNOL	AWUKIN
AFUZIK	CAPROA	ACICAR	AZUMUE	DECKIF
YEFKOM	BEBKUP	CEKWOE	BEDXIR	BUSGIH
AGAJUN	COXKEH	AYUWIB	BATPET	DANHUW
DEYJUO	AFEXOZ	CUZYEB	TEGMEA	DEZWEK
DECDUM	AVAFEK	ABEFUJ	CAKBEY	BEKMIN
CAGWUE	ADUSOJ	BETTAW	HEDCEB	ZEFPEI
ADUMOB	CARMEP	DEFZEV	COFJOW	AFESEK
AGAWAH	CAHHEZ	CEWXUY	MAZMOS	AGEXAN
ABONIQ	DEKKUA	CEVYUY	BEWTEE	CATTID
CINMAC06	BAJNIJ	DEGLAE	CUHRED	BAFPEF
CEZGEU	ALEMOV	AKOJEQ	DECYUF	DEKNOY
BICFUQ	AKOGAJ	VEDYEL	ALAFID	VEFVUA
COHTIE	ASONIF	BOMLUM	COZPEM	CEWGES
COLBUA	BOXQEK	BUXSIX	CEVRIG	CAWTIE
AXOQIO	ATEBOS	AMEXOH	DIDHII	LECHAF
CUBNES01	AXOYOB	BRACTN01	COGNOD	AZADAG02
CUVSUH	ABACOW	BOPTAD	DHXBZP	ALEXIZ
DEZJAU	CEKRIT	DAPYID	AXULOW	CIKSEU10
BOVTEN	CECGOI	DICNUY02	DANTEN14	AXIGUK
COBHUW	YEHSEM	BPHCOY10	HAZXAK	DAGCIZ
DEYBOY	BIVRUU	CAYPIE	QEGNUO	CANKOR
ACOSIV	BRPCYP02	AWODEX	DEGHIJ	BARZUQ

**DELKAH**	**AXALIV**	**APUQOT**	**CEJXOE**	**ADAJUK01**
**CITSII**	**DAKYAP**	**AVOYIT**	**COWBIB**	**CEMBAX**
**AGODEH**	**TEFNAW**	**HEDCOL**	**ACACTA**	**AWUSOB**
**BAFHUO**	**COMFER**	**TEBCAH**	**ACUYIG**	**DEGLEI**
**BUDYAB**	**BUYMAL**	**CODNOZ**	**BADTAE**	**DEPJAK**
**BUFZUZ**	**ACAYAE**	**ACAXEK**	**BICXUG**	**CUYCAA01**
**ALOHIT**	**DAXRUR03**	**BUVDAZ**	**BUWYEY**	**BADMID**
**AQEDOQ**	**CORTEJ**	**BOCBOM**	**ATUDIE**	**COSNOP**
**GEFFOP**	**BPEBXP**	**AXIPUT**	**CASBUU**	
**BOBVOF**	**CUGHIW**	**CIPPUO**	**ACOTOD**	
**CEZDOC01**	**CAFFAT**	**ABOCUS**	**BAWMUK**	
**CIKFAE**	**BESMIW**	**ARIMIZ**	**LEBKOV**	
**DAQPOD**	**ARODIW**	**TEHFOE**	**BAFZIU**	
**COPCUF01**	**COYZAT**	**BITNAT11**	**AFOVUO**	

S5.2 ChEMBL structures

CHEMBL6233	CHEMBL6317	CHEMBL507894	CHEMBL1782891
CHEMBL530730	CHEMBL6512	CHEMBL582894	CHEMBL503670
CHEMBL526346	CHEMBL4116113	CHEMBL3309345	CHEMBL1093288
CHEMBL501968	CHEMBL1199062	CHEMBL503828	CHEMBL6218
CHEMBL415615	CHEMBL540947	CHEMBL1076286	CHEMBL6239
CHEMBL4116156	CHEMBL1082898	CHEMBL6620	CHEMBL158232
CHEMBL589524	CHEMBL268086	CHEMBL609497	CHEMBL265732
CHEMBL608409	CHEMBL1165739	CHEMBL438329	CHEMBL3309505
CHEMBL507464	CHEMBL589825	CHEMBL1161924	CHEMBL1077609
CHEMBL4116093	CHEMBL570853	CHEMBL1076214	CHEMBL500437
CHEMBL501871	CHEMBL265564	CHEMBL1094711	CHEMBL3309555
CHEMBL1205595	CHEMBL553669	CHEMBL501542	CHEMBL504800
CHEMBL1214096	CHEMBL4116123	CHEMBL6521	CHEMBL162280
CHEMBL583727	CHEMBL3309323	CHEMBL1089598	CHEMBL1078513
CHEMBL1077805	CHEMBL525228	CHEMBL1213147	CHEMBL1161178
CHEMBL1214513	CHEMBL507756	CHEMBL1099016	CHEMBL3309775
CHEMBL541498	CHEMBL1091719	CHEMBL1084425	CHEMBL3309304
CHEMBL1077089	CHEMBL443602	CHEMBL1088782	CHEMBL3347277
CHEMBL1084042	CHEMBL1215108	CHEMBL4116099	CHEMBL505403
CHEMBL6222	CHEMBL1077528	CHEMBL503345	CHEMBL155636
CHEMBL525996	CHEMBL6500	CHEMBL501828	CHEMBL1076072
CHEMBL506308	CHEMBL154556	CHEMBL502187	CHEMBL1160112
CHEMBL4116103	CHEMBL1159641	CHEMBL6352	CHEMBL1096263
CHEMBL442894	CHEMBL526882	CHEMBL6517	CHEMBL1187970
CHEMBL1076054	CHEMBL4116162	CHEMBL6623	CHEMBL504162
CHEMBL154714	CHEMBL501923	CHEMBL500179	CHEMBL1165499
CHEMBL503237	CHEMBL1224871	CHEMBL490449	CHEMBL505036
CHEMBL571967	CHEMBL553904	CHEMBL1097360	CHEMBL503422
CHEMBL500090	CHEMBL610484	CHEMBL1082532	CHEMBL500548
CHEMBL1201843	CHEMBL3309396	CHEMBL164518	CHEMBL505924
CHEMBL503977	CHEMBL3309458	CHEMBL524158	CHEMBL1086220
CHEMBL582852	CHEMBL555454	CHEMBL444522	CHEMBL6789
CHEMBL592631	CHEMBL569917	CHEMBL1170659	CHEMBL439520
CHEMBL500790	CHEMBL163819	CHEMBL1207940	CHEMBL611662
CHEMBL1163275	CHEMBL572743	CHEMBL162495	CHEMBL501877
CHEMBL1094672	CHEMBL155656	CHEMBL612001	CHEMBL3309508
CHEMBL6586	CHEMBL260906	CHEMBL534280	CHEMBL1076290
CHEMBL2094221	CHEMBL1076577	CHEMBL1076712	CHEMBL155537
CHEMBL6347	CHEMBL593625	CHEMBL1213701	CHEMBL499809
CHEMBL4116092	CHEMBL262267	CHEMBL503454	CHEMBL1213094
CHEMBL6211	CHEMBL1159612	CHEMBL1200656	CHEMBL507903
CHEMBL572203	CHEMBL526533	CHEMBL1084482	CHEMBL505139

CHEMBL506407	CHEMBL1163239	CHEMBL499568	CHEMBL1084615
CHEMBL1215831	CHEMBL1076999	CHEMBL507307	CHEMBL499529
CHEMBL383917	CHEMBL504363	CHEMBL199468	CHEMBL609878
CHEMBL504357	CHEMBL1083541	CHEMBL1090695	CHEMBL403325
CHEMBL3309292	CHEMBL501943	CHEMBL1078223	CHEMBL553645
CHEMBL160738	CHEMBL1205150	CHEMBL1208484	CHEMBL6312
CHEMBL500738	CHEMBL1195488	CHEMBL575375	CHEMBL555367
CHEMBL526313	CHEMBL4116000	CHEMBL505936	CHEMBL1205733
CHEMBL589736	CHEMBL1090660	CHEMBL503400	CHEMBL1187588
CHEMBL1224864	CHEMBL526157	CHEMBL1161558	CHEMBL1159511
CHEMBL501766	CHEMBL1163216	CHEMBL3309423	CHEMBL1222790
CHEMBL6624	CHEMBL1097917	CHEMBL1088344	CHEMBL500734
CHEMBL1083268	CHEMBL1076128	CHEMBL1222611	CHEMBL1170054
CHEMBL476536	CHEMBL6240	CHEMBL155439	CHEMBL1162204
CHEMBL1204470	CHEMBL443686	CHEMBL6234	CHEMBL502243
CHEMBL500370	CHEMBL1222867	CHEMBL499583	CHEMBL1165018
CHEMBL1094701	CHEMBL1214058	CHEMBL444024	CHEMBL157367
CHEMBL502312	CHEMBL564427	CHEMBL406648	CHEMBL4116131
CHEMBL505405	CHEMBL3309480	CHEMBL1092426	CHEMBL1215356
CHEMBL268339	CHEMBL1083543	CHEMBL503860	CHEMBL607490
CHEMBL441131	CHEMBL1077287	CHEMBL506403	CHEMBL498871
CHEMBL505038	CHEMBL1159458	CHEMBL462274	CHEMBL438024
CHEMBL500105	CHEMBL593912	CHEMBL1076217	CHEMBL573461
CHEMBL1222581	CHEMBL503357	CHEMBL157397	CHEMBL154192
CHEMBL157042	CHEMBL154463	CHEMBL439138	CHEMBL584773
CHEMBL1075839	CHEMBL505819	CHEMBL508224	CHEMBL576313
CHEMBL590981	CHEMBL1209501	CHEMBL502613	CHEMBL1077360
CHEMBL501517	CHEMBL1076803	CHEMBL548334	CHEMBL216546
CHEMBL501597	CHEMBL572530	CHEMBL3309524	CHEMBL1209731
CHEMBL1170648	CHEMBL1076614	CHEMBL593672	CHEMBL6415
CHEMBL265900	CHEMBL1094354	CHEMBL503535	CHEMBL502354
CHEMBL1086957	CHEMBL1097210	CHEMBL1159639	CHEMBL1098688
CHEMBL3309440	CHEMBL504410	CHEMBL540242	CHEMBL1095673
CHEMBL532261	CHEMBL574347	CHEMBL525765	CHEMBL154228
CHEMBL504164	CHEMBL2070335	CHEMBL504254	CHEMBL1160429
CHEMBL595990	CHEMBL501682	CHEMBL498849	CHEMBL552766
CHEMBL501801	CHEMBL154818	CHEMBL499523	CHEMBL1076341
CHEMBL584981	CHEMBL4116090	CHEMBL3347310	CHEMBL4116132
CHEMBL1091763	CHEMBL552528	CHEMBL1076212	CHEMBL262299
CHEMBL538385	CHEMBL1160274	CHEMBL1213533	CHEMBL1081781
CHEMBL501493	CHEMBL1089958	CHEMBL527081	CHEMBL1083863
CHEMBL503606	CHEMBL568973	CHEMBL217730	CHEMBL504192
CHEMBL1201356	CHEMBL571980	CHEMBL156363	CHEMBL590751
CHEMBL1076792	CHEMBL502307	CHEMBL504150	CHEMBL1185211

CHEMBL577777	CHEMBL269191	CHEMBL6424	CHEMBL1165220
CHEMBL1089152	CHEMBL3309661	CHEMBL154837	CHEMBL262924
CHEMBL574570	CHEMBL4116153	CHEMBL1094671	CHEMBL470546
CHEMBL574928	CHEMBL385384	CHEMBL563301	CHEMBL1199671
CHEMBL1162102	CHEMBL593658	CHEMBL153812	CHEMBL501610
CHEMBL6324	CHEMBL6788	CHEMBL1097916	CHEMBL3347285
CHEMBL1163213	CHEMBL503539	CHEMBL1215838	CHEMBL1078396
CHEMBL155780	CHEMBL1099322	CHEMBL4116109	CHEMBL6224
CHEMBL4116160	CHEMBL439400	CHEMBL503391	CHEMBL538460
CHEMBL503508	CHEMBL6414	CHEMBL503986	CHEMBL583299
CHEMBL1076268	CHEMBL6376	CHEMBL6316	CHEMBL501770
CHEMBL1076408	CHEMBL503467	CHEMBL1077303	CHEMBL1224265
CHEMBL593674	CHEMBL1090651	CHEMBL570399	CHEMBL509250
CHEMBL501544	CHEMBL573234	CHEMBL501136	CHEMBL509348
CHEMBL570152	CHEMBL588522	CHEMBL503991	CHEMBL601080
CHEMBL504059	CHEMBL266902	CHEMBL503434	CHEMBL590259
CHEMBL529450	CHEMBL6243	CHEMBL576264	CHEMBL6562
CHEMBL564926	CHEMBL1078741	CHEMBL607213	CHEMBL498846
CHEMBL503315	CHEMBL443332	CHEMBL1199236	CHEMBL1205646
CHEMBL159567	CHEMBL6489	CHEMBL577782	CHEMBL584402
CHEMBL405398	CHEMBL3309512	CHEMBL1162403	CHEMBL6321
CHEMBL3309314	CHEMBL538639	CHEMBL500088	CHEMBL501969
CHEMBL1171471	CHEMBL553933	CHEMBL500206	CHEMBL6498
CHEMBL1096890	CHEMBL1162462	CHEMBL1095821	CHEMBL3309375
CHEMBL3309763	CHEMBL590000	CHEMBL502210	CHEMBL4116119
CHEMBL6442	CHEMBL1161567	CHEMBL158217	CHEMBL1223976
CHEMBL528112	CHEMBL504250	CHEMBL444736	CHEMBL1091740
CHEMBL503994	CHEMBL157232	CHEMBL6396	CHEMBL581077
CHEMBL1094912	CHEMBL1078134	CHEMBL550693	CHEMBL1207979
CHEMBL501647	CHEMBL6378	CHEMBL584356	CHEMBL1076555
CHEMBL579790	CHEMBL508213	CHEMBL443179	CHEMBL504907
CHEMBL1188238	CHEMBL6505	CHEMBL444748	CHEMBL498962
CHEMBL568755	CHEMBL6519	CHEMBL1076027	CHEMBL1099227
CHEMBL502986	CHEMBL1159526	CHEMBL502653	CHEMBL6309
CHEMBL569907	CHEMBL554546	CHEMBL4116114	CHEMBL1196830
CHEMBL526702	CHEMBL78310	CHEMBL4116121	CHEMBL502494
CHEMBL4116143	CHEMBL1162169	CHEMBL6232	CHEMBL610892
CHEMBL265282	CHEMBL583726	CHEMBL1079305	CHEMBL1210347
CHEMBL1094634	CHEMBL501691	CHEMBL564205	CHEMBL159668
CHEMBL4116117	CHEMBL1162317	CHEMBL547407	CHEMBL1164909
CHEMBL508600	CHEMBL502441	CHEMBL1620719	CHEMBL262819
CHEMBL583319	CHEMBL6417	CHEMBL585377	CHEMBL559342
CHEMBL504077	CHEMBL503469	CHEMBL1165423	CHEMBL6282
CHEMBL1207533	CHEMBL1186606	CHEMBL607833	CHEMBL1187952

CHEMBL1172056
CHEMBL608190
CHEMBL385868
CHEMBL3309538
CHEMBL558352
CHEMBL1224734
CHEMBL6320
CHEMBL1209733
CHEMBL502626
CHEMBL1084477
CHEMBL499101
CHEMBL1099299
CHEMBL569918
CHEMBL156263
CHEMBL570602
CHEMBL608847
CHEMBL160417
CHEMBL588329
CHEMBL1173577
CHEMBL503350
CHEMBL499543
CHEMBL6230
CHEMBL500111
CHEMBL606119
CHEMBL1076357
CHEMBL454492
CHEMBL502357
CHEMBL501858
CHEMBL445258
CHEMBL6349
CHEMBL610879
CHEMBL1201754
CHEMBL413611
CHEMBL1170061
CHEMBL1075666
CHEMBL538188
CHEMBL1089238
CHEMBL541013
CHEMBL1091391
CHEMBL154209
CHEMBL1205641
CHEMBL6942
CHEMBL612143
CHEMBL1163477
CHEMBL1215684
CHEMBL159389
CHEMBL4116104
CHEMBL1224854
CHEMBL505127
CHEMBL415423
CHEMBL501070
CHEMBL538686
CHEMBL502983
CHEMBL6516
CHEMBL264899
CHEMBL6306
CHEMBL610765
CHEMBL1093316
CHEMBL1160256
CHEMBL1172235
CHEMBL502212
CHEMBL1088337
CHEMBL542011
CHEMBL265174
CHEMBL1208485
CHEMBL559228
CHEMBL20226
CHEMBL500195
CHEMBL6486
CHEMBL570634
CHEMBL1076491
CHEMBL500079
CHEMBL593671
CHEMBL507459
CHEMBL1201295
CHEMBL592409
CHEMBL603059
CHEMBL1222975
CHEMBL1077476
CHEMBL1097293
CHEMBL177285
CHEMBL1086439
CHEMBL3309411
CHEMBL1223923
CHEMBL611935
CHEMBL520435
CHEMBL6509
CHEMBL552742
CHEMBL503449
CHEMBL1075896
CHEMBL503616
CHEMBL504351
CHEMBL583947
CHEMBL156224
CHEMBL1207630
CHEMBL503902
CHEMBL525672
CHEMBL500097
CHEMBL1199724
CHEMBL1077336
CHEMBL1092024
CHEMBL1164952
CHEMBL500758
CHEMBL1198965
CHEMBL1213498
CHEMBL165532
CHEMBL157446
CHEMBL263810
CHEMBL1172581
CHEMBL1209811
CHEMBL1165737
CHEMBL583519
CHEMBL1089589
CHEMBL503702
CHEMBL1199159
CHEMBL611044
CHEMBL3347290
CHEMBL1076911
CHEMBL507538
CHEMBL593668
CHEMBL610272
CHEMBL1187270
CHEMBL1221917
CHEMBL573437
CHEMBL1077257
CHEMBL503559
CHEMBL6792
CHEMBL1089583
CHEMBL438301
CHEMBL4116154
CHEMBL611047
CHEMBL504086
CHEMBL155896
CHEMBL576493
CHEMBL167990
CHEMBL1165530
CHEMBL443598
CHEMBL509252
CHEMBL266873
CHEMBL500202
CHEMBL1223980
CHEMBL508100
CHEMBL534251
CHEMBL583071
CHEMBL154414
CHEMBL407362
CHEMBL405416
CHEMBL1075841
CHEMBL4116107
CHEMBL6311
CHEMBL438839
CHEMBL563935
CHEMBL541686
CHEMBL4116164
CHEMBL508166
CHEMBL572999
CHEMBL161573
CHEMBL155331
CHEMBL539203
CHEMBL4116111
CHEMBL576309
CHEMBL267832
CHEMBL500495
CHEMBL507207
CHEMBL1207998
CHEMBL6308
CHEMBL1165404
CHEMBL600052
CHEMBL503204
CHEMBL6323
CHEMBL499943
CHEMBL1094665
CHEMBL156703
CHEMBL579880
CHEMBL6496
CHEMBL504333

CHEMBL1076248
CHEMBL1210852
CHEMBL504168
CHEMBL164375
CHEMBL584341
CHEMBL525428
CHEMBL6395
CHEMBL6287
CHEMBL501763
CHEMBL443597
CHEMBL4116129
CHEMBL1213185
CHEMBL155451
CHEMBL3309270
CHEMBL441620
CHEMBL498861
CHEMBL6446
CHEMBL532920
CHEMBL525413
CHEMBL503770
CHEMBL500372
CHEMBL3309860
CHEMBL590067
CHEMBL3309871
CHEMBL1223703
CHEMBL1091702
CHEMBL1096572
CHEMBL1076905
CHEMBL504035
CHEMBL1187750
CHEMBL3309490
CHEMBL501587
CHEMBL1077081
CHEMBL266960
CHEMBL506057
CHEMBL3347320
CHEMBL502201
CHEMBL507224
CHEMBL540696
CHEMBL538901
CHEMBL575372
CHEMBL504397
CHEMBL1162080
CHEMBL541969
CHEMBL1207937
CHEMBL501922
CHEMBL3309287
CHEMBL573078
CHEMBL1077407
CHEMBL4116118
CHEMBL1162174
CHEMBL6445
CHEMBL564260
CHEMBL503619
CHEMBL1076770
CHEMBL1088966
CHEMBL1206474
CHEMBL8
CHEMBL261102
CHEMBL444231
CHEMBL1171794
CHEMBL6497
CHEMBL1224447
CHEMBL167929
CHEMBL539921
CHEMBL1079618
CHEMBL180233
CHEMBL1164922
CHEMBL157834
CHEMBL259626
CHEMBL503865
CHEMBL584783
CHEMBL438327
CHEMBL265830
CHEMBL3309279
CHEMBL574025
CHEMBL502624
CHEMBL1076221
CHEMBL499961
CHEMBL1077021
CHEMBL174668
CHEMBL505283
CHEMBL1086190
CHEMBL547643
CHEMBL1077098
CHEMBL593933
CHEMBL1076587
CHEMBL573664
CHEMBL1213309
CHEMBL1091854
CHEMBL163579
CHEMBL3309327
CHEMBL1077261
CHEMBL573459
CHEMBL1097634
CHEMBL539714
CHEMBL6235
CHEMBL1188857
CHEMBL591437
CHEMBL563918
CHEMBL503818
CHEMBL573674
CHEMBL525924
CHEMBL503266
CHEMBL437851
CHEMBL503028
CHEMBL553887
CHEMBL499517
CHEMBL9
CHEMBL503623
CHEMBL1195116
CHEMBL4116168
CHEMBL1186290
CHEMBL262399
CHEMBL573919
CHEMBL571523
CHEMBL6322
CHEMBL583920
CHEMBL6225
CHEMBL1097356
CHEMBL584397
CHEMBL569031
CHEMBL1163060
CHEMBL444368
CHEMBL541005
CHEMBL264055
CHEMBL1091275
CHEMBL502172
CHEMBL502640
CHEMBL6350
CHEMBL595793
CHEMBL3309654
CHEMBL3309443
CHEMBL1171111
CHEMBL504349
CHEMBL1186168
CHEMBL609524
CHEMBL505930
CHEMBL507700
CHEMBL503030
CHEMBL157236
CHEMBL1162097
CHEMBL1083881
CHEMBL1213854
CHEMBL1163095
CHEMBL263193
CHEMBL1205279
CHEMBL1170046
CHEMBL500474
CHEMBL592623
CHEMBL408
CHEMBL1186195
CHEMBL546744
CHEMBL6304
CHEMBL504216
CHEMBL503982
CHEMBL504249
CHEMBL1223009
CHEMBL498970
CHEMBL6399
CHEMBL153717
CHEMBL500021
CHEMBL3309670
CHEMBL3309324
CHEMBL502181
CHEMBL501665
CHEMBL562776
CHEMBL1093521
CHEMBL508095
CHEMBL6422
CHEMBL1213767
CHEMBL6441
CHEMBL501130
CHEMBL610474
CHEMBL558744
CHEMBL1091561

CHEMBL595064
CHEMBL1079127
CHEMBL540227
CHEMBL595330
CHEMBL576723
CHEMBL508212
CHEMBL549249
CHEMBL6229
CHEMBL1189651
CHEMBL158960
CHEMBL539138
CHEMBL503451
CHEMBL1086530
CHEMBL6404
CHEMBL3309471
CHEMBL6487
CHEMBL4116137
CHEMBL572625
CHEMBL438139
CHEMBL1086459
CHEMBL578763
CHEMBL182355
CHEMBL1096673
CHEMBL504451
CHEMBL1163392
CHEMBL1160680
CHEMBL157459
CHEMBL1170815
CHEMBL523218
CHEMBL507542
CHEMBL603409
CHEMBL539434
CHEMBL580660
CHEMBL541695
CHEMBL163871
CHEMBL6216
CHEMBL1170662
CHEMBL6462
CHEMBL570872
CHEMBL539442
CHEMBL153086
CHEMBL1091402
CHEMBL558954
CHEMBL405225
CHEMBL265763
CHEMBL1186068
CHEMBL1088217
CHEMBL541693
CHEMBL498986
CHEMBL505126
CHEMBL501256
CHEMBL1091285
CHEMBL4116157
CHEMBL574221
CHEMBL1076895
CHEMBL1093669
CHEMBL1097664
CHEMBL1076814
CHEMBL1096337
CHEMBL539185
CHEMBL507884
CHEMBL527552
CHEMBL1085658
CHEMBL154341
CHEMBL1170642
CHEMBL1161220
CHEMBL154655
CHEMBL1094237
CHEMBL600373
CHEMBL4116089
CHEMBL508215
CHEMBL158175
CHEMBL526543
CHEMBL3309541
CHEMBL156901
CHEMBL4116122
CHEMBL1162071
CHEMBL1229215
CHEMBL1091340
CHEMBL6425
CHEMBL1076131
CHEMBL6409
CHEMBL541483
CHEMBL6237
CHEMBL580443
CHEMBL1086527
CHEMBL506660
CHEMBL6565
CHEMBL3309334
CHEMBL501315
CHEMBL264521
CHEMBL605173
CHEMBL6504
CHEMBL1076755
CHEMBL499532
CHEMBL6402
CHEMBL6561
CHEMBL552993
CHEMBL1210336
CHEMBL1159426
CHEMBL1185850
CHEMBL1171366
CHEMBL6334
CHEMBL1200714
CHEMBL6418
CHEMBL6626
CHEMBL583305
CHEMBL1095192
CHEMBL445025
CHEMBL1076077
CHEMBL160341
CHEMBL498936
CHEMBL501918
CHEMBL3309261
CHEMBL1172222
CHEMBL1094708
CHEMBL4116146
CHEMBL507540
CHEMBL502296
CHEMBL500730
CHEMBL505923
CHEMBL1097906
CHEMBL1088204
CHEMBL505397
CHEMBL500467
CHEMBL1090111
CHEMBL1209153
CHEMBL1096781
CHEMBL4116091
CHEMBL1224732
CHEMBL540943
CHEMBL262664
CHEMBL155459
CHEMBL592411
CHEMBL572517
CHEMBL3309390
CHEMBL460994
CHEMBL6447
CHEMBL501280
CHEMBL608687
CHEMBL556060
CHEMBL1088145
CHEMBL1083224
CHEMBL1076101
CHEMBL609929
CHEMBL1097992
CHEMBL1085285
CHEMBL1077282
CHEMBL441569
CHEMBL261894
CHEMBL506560
CHEMBL1091334
CHEMBL1205123
CHEMBL1169603
CHEMBL6423
CHEMBL6790
CHEMBL540167
CHEMBL590478
CHEMBL1163408
CHEMBL1213982
CHEMBL504425
CHEMBL1093640
CHEMBL1092064
CHEMBL502048
CHEMBL1075834
CHEMBL4116130
CHEMBL578823
CHEMBL584399
CHEMBL502053
CHEMBL540427
CHEMBL1075878
CHEMBL1163140
CHEMBL1091319
CHEMBL1093989
CHEMBL1186096
CHEMBL1094608

CHEMBL558759	CHEMBL553918	CHEMBL269289	CHEMBL552187
CHEMBL506071	CHEMBL1096298	CHEMBL506640	CHEMBL500627
CHEMBL1184894	CHEMBL584197	CHEMBL1162464	CHEMBL4116106
CHEMBL502461	CHEMBL154392	CHEMBL575370	CHEMBL608706
CHEMBL1170234	CHEMBL593812	CHEMBL1077368	CHEMBL499520
CHEMBL595765	CHEMBL6502	CHEMBL574851	CHEMBL6259
CHEMBL502490	CHEMBL3347288	CHEMBL6566	CHEMBL263555
CHEMBL526377	CHEMBL1094998	CHEMBL155374	CHEMBL499823
CHEMBL1163147	CHEMBL1223111	CHEMBL265130	CHEMBL606771
CHEMBL1207835	CHEMBL504429	CHEMBL594331	CHEMBL6440
CHEMBL502192	CHEMBL1207408	CHEMBL504909	CHEMBL1172041
CHEMBL445177	CHEMBL503232	CHEMBL6939	CHEMBL503430
CHEMBL1075993	CHEMBL6400	CHEMBL1093647	CHEMBL155263
CHEMBL1210364	CHEMBL468771	CHEMBL574930	CHEMBL438807
CHEMBL155979	CHEMBL1087095	CHEMBL501701	CHEMBL579584
CHEMBL541740	CHEMBL539191	CHEMBL4116148	CHEMBL1159434
CHEMBL1163069	CHEMBL500085	CHEMBL162493	CHEMBL1075723
CHEMBL1186537	CHEMBL568776	CHEMBL1163197	CHEMBL505023
CHEMBL1162300	CHEMBL3309352	CHEMBL577964	CHEMBL503252
CHEMBL1162209	CHEMBL503713	CHEMBL503221	CHEMBL1082938
CHEMBL444145	CHEMBL1092059	CHEMBL584792	CHEMBL507764
CHEMBL1088996	CHEMBL6518	CHEMBL538627	CHEMBL4116145
CHEMBL1185294	CHEMBL1160771	CHEMBL1099329	CHEMBL1198796
CHEMBL1209204	CHEMBL1076088	CHEMBL1093605	CHEMBL6348
CHEMBL500267	CHEMBL1163207	CHEMBL6242	CHEMBL1163234
CHEMBL538692	CHEMBL568607	CHEMBL503046	CHEMBL1076110
CHEMBL154314	CHEMBL1207867	CHEMBL1200037	CHEMBL557964
CHEMBL1088309	CHEMBL3309302	CHEMBL6413	CHEMBL577321
CHEMBL503463	CHEMBL498905	CHEMBL1165801	CHEMBL584146
CHEMBL506415	CHEMBL584990	CHEMBL160210	CHEMBL538670
CHEMBL6887	CHEMBL1091501	CHEMBL1090356	CHEMBL1186245
CHEMBL1092443	CHEMBL26565	CHEMBL501694	CHEMBL445172
CHEMBL577535	CHEMBL441948	CHEMBL540674	CHEMBL1171643
CHEMBL165039	CHEMBL4116120	CHEMBL502166	CHEMBL608971
CHEMBL502183	CHEMBL1229187	CHEMBL264812	CHEMBL610630
CHEMBL504052	CHEMBL555469	CHEMBL608826	CHEMBL503304
CHEMBL554586	CHEMBL1159451	CHEMBL6511	CHEMBL601469
CHEMBL3347298	CHEMBL146675	CHEMBL1170828	CHEMBL1083581
CHEMBL1222016	CHEMBL541435	CHEMBL263614	CHEMBL1162109
CHEMBL525153	CHEMBL1077248	CHEMBL504332	CHEMBL500769
CHEMBL6465	CHEMBL4116096	CHEMBL593201	CHEMBL1079031
CHEMBL6463	CHEMBL1224737	CHEMBL509195	CHEMBL1076925
CHEMBL500109	CHEMBL574027	CHEMBL500346	CHEMBL506058
CHEMBL501674	CHEMBL1077381	CHEMBL6314	CHEMBL606548

CHEMBL590853
CHEMBL154832
CHEMBL1076775
CHEMBL1093690
CHEMBL539179
CHEMBL1214894
CHEMBL4115984
CHEMBL1085713
CHEMBL6514
CHEMBL1214854
CHEMBL6403
CHEMBL1214455
CHEMBL506642
CHEMBL6583
CHEMBL164857
CHEMBL154360
CHEMBL6618
CHEMBL6284
CHEMBL503340
CHEMBL444924
CHEMBL1090668
CHEMBL6370
CHEMBL1076294
CHEMBL1082437
CHEMBL1206762
CHEMBL576519
CHEMBL603735
CHEMBL1076668
CHEMBL500054
CHEMBL1090649
CHEMBL1184883
CHEMBL1076769
CHEMBL494682
CHEMBL1207158
CHEMBL577467
CHEMBL500527
CHEMBL502582
CHEMBL415391
CHEMBL591201
CHEMBL1085225
CHEMBL414319
CHEMBL4116005
CHEMBL596211
CHEMBL1210356
CHEMBL1094324
CHEMBL1182827
CHEMBL1095997
CHEMBL500519
CHEMBL405667
CHEMBL6510
CHEMBL584766
CHEMBL6538
CHEMBL535264
CHEMBL1199633
CHEMBL501671
CHEMBL1179567
CHEMBL1179555
CHEMBL1088958
CHEMBL6220
CHEMBL6484
CHEMBL501593
CHEMBL602514
CHEMBL4116102
CHEMBL1173726
CHEMBL1089592
CHEMBL6615
CHEMBL444434
CHEMBL6226
CHEMBL444987
CHEMBL260909
CHEMBL6699
CHEMBL590003
CHEMBL3309436
CHEMBL581928
CHEMBL557764
CHEMBL503643
CHEMBL6398
CHEMBL538457
CHEMBL1172569
CHEMBL412007
CHEMBL504560
CHEMBL6467
CHEMBL438822
CHEMBL507305
CHEMBL1200285
CHEMBL557954
CHEMBL500377
CHEMBL1160450
CHEMBL503044
CHEMBL1077444
CHEMBL558162
CHEMBL501508
CHEMBL6214
CHEMBL504419
CHEMBL1214724
CHEMBL157450
CHEMBL502200
CHEMBL6286
CHEMBL3309496
CHEMBL600456
CHEMBL1090670
CHEMBL583567
CHEMBL1170005
CHEMBL500257
CHEMBL540195
CHEMBL500809
CHEMBL1077878
CHEMBL6258
CHEMBL1204471
CHEMBL502655
CHEMBL1077270
CHEMBL3309266
CHEMBL1090597
CHEMBL503058
CHEMBL507303
CHEMBL538380
CHEMBL502052
CHEMBL610050
CHEMBL553689
CHEMBL540153
CHEMBL1077410
CHEMBL1171146
CHEMBL3348817
CHEMBL592632
CHEMBL4116141
CHEMBL1163233
CHEMBL154170
CHEMBL501940
CHEMBL574800
CHEMBL165949
CHEMBL267740
CHEMBL559340
CHEMBL6468
CHEMBL1171136
CHEMBL4116167
CHEMBL3309460
CHEMBL500249
CHEMBL541967
CHEMBL1214569
CHEMBL502429
CHEMBL502203
CHEMBL503838
CHEMBL506068
CHEMBL593676
CHEMBL1079027
CHEMBL1237043
CHEMBL500704
CHEMBL525418
CHEMBL1160711
CHEMBL578346
CHEMBL1222706
CHEMBL1096640
CHEMBL541754
CHEMBL1086372
CHEMBL501274
CHEMBL1077485
CHEMBL6444
CHEMBL501687
CHEMBL500709
CHEMBL6564
CHEMBL498921
CHEMBL4116140
CHEMBL6215
CHEMBL501821
CHEMBL1098866
CHEMBL1086312
CHEMBL1088641
CHEMBL3309284
CHEMBL1187911
CHEMBL409812
CHEMBL1076074
CHEMBL503549
CHEMBL498966
CHEMBL154940
CHEMBL6619
CHEMBL1096579

CHEMBL1096583
CHEMBL1098259
CHEMBL505806
CHEMBL1095988
CHEMBL1089585
CHEMBL4116128
CHEMBL1222793
CHEMBL6437
CHEMBL1095336
CHEMBL538453
CHEMBL6328
CHEMBL502486
CHEMBL4116144
CHEMBL1079125
CHEMBL501276
CHEMBL576512
CHEMBL507896
CHEMBL1223621
CHEMBL605451
CHEMBL503541
CHEMBL502423
CHEMBL4116001
CHEMBL262244
CHEMBL157267
CHEMBL527864
CHEMBL505400
CHEMBL1077333
CHEMBL264137
CHEMBL4116136
CHEMBL1089276
CHEMBL548124
CHEMBL583100
CHEMBL6938
CHEMBL442595
CHEMBL555916
CHEMBL574253
CHEMBL1209874
CHEMBL504372
CHEMBL601082
CHEMBL6464
CHEMBL504252
CHEMBL503725
CHEMBL501874
CHEMBL601272
CHEMBL1214612
CHEMBL1088980
CHEMBL541956
CHEMBL6698
CHEMBL502411
CHEMBL501251
CHEMBL1208835
CHEMBL164817
CHEMBL1080664
CHEMBL1089317
CHEMBL409297
CHEMBL1215806
CHEMBL165476
CHEMBL1207360
CHEMBL499959
CHEMBL1089576
CHEMBL1171133
CHEMBL1089574
CHEMBL574797
CHEMBL1185408
CHEMBL504420
CHEMBL574932
CHEMBL589985
CHEMBL590250
CHEMBL547008
CHEMBL1076637
CHEMBL1076098
CHEMBL6888
CHEMBL154771
CHEMBL1075679
CHEMBL591773
CHEMBL6534
CHEMBL1180347
CHEMBL1204461
CHEMBL414390
CHEMBL1076906
CHEMBL1160275
CHEMBL508159
CHEMBL503870
CHEMBL3309338
CHEMBL196395
CHEMBL1163153
CHEMBL526183
CHEMBL610275
CHEMBL6393
CHEMBL6335
CHEMBL503814
CHEMBL501650
CHEMBL578483
CHEMBL1204670
CHEMBL1082274
CHEMBL1215485
CHEMBL504055
CHEMBL1189068
CHEMBL6330
CHEMBL1222580
CHEMBL1183203
CHEMBL500269
CHEMBL503548
CHEMBL22
CHEMBL1161852
CHEMBL554494
CHEMBL4116158
CHEMBL1082278
CHEMBL503523
CHEMBL503634
CHEMBL584554
CHEMBL258525
CHEMBL4116100
CHEMBL1198802
CHEMBL3309273
CHEMBL1163186
CHEMBL502985
CHEMBL1207397
CHEMBL6208
CHEMBL3309383
CHEMBL1089667
CHEMBL3309561
CHEMBL504142
CHEMBL501507
CHEMBL1206530
CHEMBL1086063
CHEMBL525627
CHEMBL1171525
CHEMBL162786
CHEMBL501328
CHEMBL445000
CHEMBL505033
CHEMBL263329
CHEMBL1224292
CHEMBL499563
CHEMBL1187868
CHEMBL1164921
CHEMBL1213497
CHEMBL1208314
CHEMBL1163149
CHEMBL6560
CHEMBL444233
CHEMBL506982
CHEMBL592870
CHEMBL3309336
CHEMBL1207983
CHEMBL1171716
CHEMBL1162315
CHEMBL1088591
CHEMBL1208034
CHEMBL6217
CHEMBL503289
CHEMBL6231
CHEMBL260023
CHEMBL607364
CHEMBL154357
CHEMBL1199367
CHEMBL1210111
CHEMBL1082636
CHEMBL316793
CHEMBL572573
CHEMBL1199618
CHEMBL4116125
CHEMBL501326
CHEMBL6332
CHEMBL531135
CHEMBL1188581
CHEMBL1159483
CHEMBL526908
CHEMBL6466
CHEMBL503501
CHEMBL575374
CHEMBL1094663
CHEMBL3309504
CHEMBL6261
CHEMBL1186057

CHEMBL570151
CHEMBL402708
CHEMBL6245
CHEMBL1199171
CHEMBL1162038
CHEMBL524317
CHEMBL6791
CHEMBL503846
CHEMBL6696
CHEMBL506637
CHEMBL1084953
CHEMBL1160322
CHEMBL6209
CHEMBL603145
CHEMBL503845
CHEMBL540214
CHEMBL503644
CHEMBL444524
CHEMBL1205831
CHEMBL1186159
CHEMBL438132
CHEMBL505943
CHEMBL1214443
CHEMBL6283
CHEMBL538895
CHEMBL1170644
CHEMBL503474
CHEMBL503411
CHEMBL611973
CHEMBL1077272
CHEMBL1163200
CHEMBL504184
CHEMBL1210578
CHEMBL501132
CHEMBL6257
CHEMBL1161944
CHEMBL589753
CHEMBL1163148
CHEMBL1077276
CHEMBL603312
CHEMBL591703
CHEMBL505140
CHEMBL592890
CHEMBL1206631
CHEMBL582415
CHEMBL3309413
CHEMBL507226
CHEMBL503907
CHEMBL582554
CHEMBL4115992
CHEMBL1085980
CHEMBL154789
CHEMBL593669
CHEMBL1210341
CHEMBL4116124
CHEMBL4116147
CHEMBL1171705
CHEMBL524910
CHEMBL1210789
CHEMBL1159637
CHEMBL1097899
CHEMBL414339
CHEMBL264472
CHEMBL3309531
CHEMBL1078833
CHEMBL154687
CHEMBL505285
CHEMBL16498
CHEMBL610191
CHEMBL161838
CHEMBL1189598
CHEMBL3309427
CHEMBL6941
CHEMBL1076790
CHEMBL1190414
CHEMBL158670
CHEMBL1088172
CHEMBL1077686
CHEMBL6416
CHEMBL508099
CHEMBL4116155
CHEMBL503419
CHEMBL500234
CHEMBL540973
CHEMBL529327
CHEMBL1098309
CHEMBL1184967
CHEMBL1096892
CHEMBL3347280
CHEMBL1183536
CHEMBL3309295
CHEMBL1170714
CHEMBL1075761
CHEMBL3309491
CHEMBL3309461
CHEMBL507127
CHEMBL500357
CHEMBL1163145
CHEMBL1171068
CHEMBL1083537
CHEMBL154609
CHEMBL591912
CHEMBL1215753
CHEMBL2310848
CHEMBL507116
CHEMBL1086503
CHEMBL1160667
CHEMBL499846
CHEMBL1209290
CHEMBL602107
CHEMBL503258
CHEMBL570855
CHEMBL6940
CHEMBL541185
CHEMBL409208
CHEMBL569690
CHEMBL595820
CHEMBL1077330
CHEMBL3309767
CHEMBL1160741
CHEMBL1171953
CHEMBL443992
CHEMBL584784
CHEMBL155478
CHEMBL538928
CHEMBL610757
CHEMBL1214515
CHEMBL444506
CHEMBL606769
CHEMBL557754
CHEMBL595083
CHEMBL595526
CHEMBL1095005
CHEMBL1223924
CHEMBL4116133
CHEMBL265362
CHEMBL583568
CHEMBL501513
CHEMBL1095653
CHEMBL500286
CHEMBL583284
CHEMBL1208039
CHEMBL572762
CHEMBL1096236
CHEMBL602507
CHEMBL1092453
CHEMBL1163203
CHEMBL526134
CHEMBL6503
CHEMBL504374
CHEMBL6310
CHEMBL609992
CHEMBL6221
CHEMBL1169618
CHEMBL541676
CHEMBL1083240
CHEMBL498858
CHEMBL602709
CHEMBL572199
CHEMBL1083593
CHEMBL500805
CHEMBL439542
CHEMBL601070
CHEMBL501925
CHEMBL1084170
CHEMBL1089969
CHEMBL503698
CHEMBL6535
CHEMBL260970
CHEMBL502456
CHEMBL1088207
CHEMBL3309451
CHEMBL1173565
CHEMBL1091420
CHEMBL443462
CHEMBL404

CHEMBL1093624
CHEMBL156555
CHEMBL1084510
CHEMBL1096822
CHEMBL501963
CHEMBL504440
CHEMBL1172204
CHEMBL500246
CHEMBL265216
CHEMBL6305
CHEMBL1188474
CHEMBL501689
CHEMBL558556
CHEMBL602115
CHEMBL501589
CHEMBL500207
CHEMBL585178
CHEMBL1180192
CHEMBL6210
CHEMBL533122
CHEMBL386654
CHEMBL594149
CHEMBL1214847
CHEMBL1208419
CHEMBL506069
CHEMBL418
CHEMBL1209155
CHEMBL608407
CHEMBL505029
CHEMBL1163139
CHEMBL1162104
CHEMBL1077002
CHEMBL1214531
CHEMBL6244
CHEMBL500056
CHEMBL584401
CHEMBL1191207
CHEMBL604773
CHEMBL503376
CHEMBL592393
CHEMBL6307
CHEMBL6625
CHEMBL529058
CHEMBL1159610
CHEMBL502237
CHEMBL1090248
CHEMBL6369
CHEMBL1094870
CHEMBL500450
CHEMBL6236
CHEMBL6411
CHEMBL4078546
CHEMBL4529157
CHEMBL332858
CHEMBL3441076
CHEMBL2048223
CHEMBL191759
CHEMBL1650551
CHEMBL294410
CHEMBL269464
CHEMBL1324243
CHEMBL2030910
CHEMBL2377372
CHEMBL3112973
CHEMBL1734202
CHEMBL135836
CHEMBL4543756
CHEMBL3409495
CHEMBL3452909
CHEMBL3958100
CHEMBL3557909
CHEMBL3237787
CHEMBL1479915
CHEMBL1812537
CHEMBL1382951
CHEMBL3936891
CHEMBL596323
CHEMBL271588
CHEMBL390614
CHEMBL152445
CHEMBL3137562
CHEMBL2419144
CHEMBL1192024
CHEMBL74450
CHEMBL2112054
CHEMBL3183213
CHEMBL515888
CHEMBL4436228
CHEMBL289779
CHEMBL3739481
CHEMBL1088150
CHEMBL131097
CHEMBL3955959
CHEMBL424967
CHEMBL4572335
CHEMBL3218234
CHEMBL1989235
CHEMBL1667921
CHEMBL3441304
CHEMBL1650467
CHEMBL604722
CHEMBL3618497
CHEMBL3556800
CHEMBL3957997
CHEMBL2048581
CHEMBL460096
CHEMBL4636037
CHEMBL274183
CHEMBL203392
CHEMBL3115317
CHEMBL2207970
CHEMBL1615456
CHEMBL79630
CHEMBL4553068
CHEMBL3805346
CHEMBL3415405
CHEMBL2348969
CHEMBL366062
CHEMBL462788
CHEMBL1096763
CHEMBL427519
CHEMBL1916116
CHEMBL428747
CHEMBL1990521
CHEMBL3183784
CHEMBL2158459
CHEMBL4543501
CHEMBL4444818
CHEMBL46506
CHEMBL1425875
CHEMBL4207592
CHEMBL4537930
CHEMBL61605
CHEMBL4204905
CHEMBL569827
CHEMBL2182022
CHEMBL51151
CHEMBL1531846
CHEMBL3640646
CHEMBL581523
CHEMBL56885
CHEMBL4282746
CHEMBL1979929
CHEMBL1595334
CHEMBL3897080
CHEMBL3309938
CHEMBL3526419
CHEMBL172867
CHEMBL1173073
CHEMBL1462296
CHEMBL4297215
CHEMBL4203795
CHEMBL3394220
CHEMBL512887
CHEMBL84242
CHEMBL3248229
CHEMBL3466603
CHEMBL4068222
CHEMBL1956786
CHEMBL141977
CHEMBL3806041
CHEMBL1907881
CHEMBL1966041
CHEMBL2070663
CHEMBL3989180
CHEMBL3973634
CHEMBL488233
CHEMBL4561227
CHEMBL3691457
CHEMBL2177508
CHEMBL3932241
CHEMBL3466621
CHEMBL3556032
CHEMBL65007
CHEMBL3786718
CHEMBL306840

CHEMBL583246
CHEMBL1885037
CHEMBL1190778
CHEMBL1160240
CHEMBL1372076
CHEMBL353197
CHEMBL197128
CHEMBL2023246
CHEMBL3961170
CHEMBL405690
CHEMBL1170180
CHEMBL3415394
CHEMBL3739450
CHEMBL3937091
CHEMBL4104992
CHEMBL3691458
CHEMBL583518
CHEMBL1990818
CHEMBL1668923
CHEMBL4437625
CHEMBL4584678
CHEMBL311081
CHEMBL4094629
CHEMBL1389442
CHEMBL4547144
CHEMBL85343
CHEMBL324401
CHEMBL3647387
CHEMBL2333414
CHEMBL359123
CHEMBL3142427
CHEMBL4225252
CHEMBL2348964
CHEMBL3401975
CHEMBL2203388
CHEMBL3893190
CHEMBL4543268
CHEMBL485833
CHEMBL4173641
CHEMBL1161212
CHEMBL4436573
CHEMBL1256
CHEMBL2376013
CHEMBL3138195
CHEMBL36348
CHEMBL4440249
CHEMBL1097370
CHEMBL3181857
CHEMBL290479
CHEMBL2324595
CHEMBL3649498
CHEMBL1983576
CHEMBL2048585
CHEMBL4574746
CHEMBL3968890
CHEMBL273814
CHEMBL1276046
CHEMBL1521612
CHEMBL1739678
CHEMBL107385
CHEMBL4513664
CHEMBL2003201
CHEMBL3916123
CHEMBL3245854
CHEMBL3314286
CHEMBL342190
CHEMBL525288
CHEMBL4638238
CHEMBL284852
CHEMBL3647006
CHEMBL1976646
CHEMBL2058378
CHEMBL1579407
CHEMBL2425323
CHEMBL311208
CHEMBL3343701
CHEMBL2448947
CHEMBL1197316
CHEMBL3394041
CHEMBL457687
CHEMBL1984735
CHEMBL1797642
CHEMBL228314
CHEMBL3040357
CHEMBL3553261
CHEMBL3437298
CHEMBL3526894
CHEMBL2106567
CHEMBL2153100
CHEMBL3246126
CHEMBL1532453
CHEMBL3706958
CHEMBL520137
CHEMBL2207027
CHEMBL3393064
CHEMBL462988
CHEMBL334013
CHEMBL1975973
CHEMBL3040319
CHEMBL4071953
CHEMBL568730
CHEMBL1864390
CHEMBL380552
CHEMBL3288966
CHEMBL226787
CHEMBL3221654
CHEMBL1964702
CHEMBL12693
CHEMBL140791
CHEMBL3249051
CHEMBL2430211
CHEMBL286807
CHEMBL3137552
CHEMBL1968388
CHEMBL3786266
CHEMBL280939
CHEMBL3248217
CHEMBL34210
CHEMBL1997172
CHEMBL3394219
CHEMBL567183
CHEMBL2207048
CHEMBL1974528
CHEMBL1192232
CHEMBL4071497
CHEMBL4073422
CHEMBL3417683
CHEMBL3702086
CHEMBL3585642
CHEMBL2153778
CHEMBL556139
CHEMBL610125
CHEMBL2064253
CHEMBL2007109
CHEMBL80063
CHEMBL352277
CHEMBL1802449
CHEMBL1892551
CHEMBL1672280
CHEMBL3971210
CHEMBL52274
CHEMBL3553788
CHEMBL500823
CHEMBL3716758
CHEMBL1998051
CHEMBL162797
CHEMBL2429967
CHEMBL4062594
CHEMBL1642720
CHEMBL1299988
CHEMBL4544479
CHEMBL2089178
CHEMBL464002
CHEMBL3401893
CHEMBL75846
CHEMBL3451098
CHEMBL3110038
CHEMBL3793496
CHEMBL352900
CHEMBL1222256
CHEMBL602415
CHEMBL1835095
CHEMBL117934
CHEMBL1278108
CHEMBL1984480
CHEMBL450878
CHEMBL72307
CHEMBL279498
CHEMBL2333408
CHEMBL463556
CHEMBL333149
CHEMBL175420
CHEMBL608422
CHEMBL1784603
CHEMBL4540688
CHEMBL488592

CHEMBL4632641	CHEMBL435822	CHEMBL3647011	CHEMBL2068853
CHEMBL1090576	CHEMBL1457741	CHEMBL3137548	CHEMBL1598455
CHEMBL455505	CHEMBL253859	CHEMBL48990	CHEMBL3548366
CHEMBL1575663	CHEMBL2079257	CHEMBL4095996	CHEMBL1209022
CHEMBL449475	CHEMBL2429976	CHEMBL2023249	CHEMBL2006718
CHEMBL240886	CHEMBL1607747	CHEMBL2079099	CHEMBL3040301
CHEMBL3235475	CHEMBL339494	CHEMBL557235	CHEMBL3818945
CHEMBL1987982	CHEMBL4170044	CHEMBL202007	CHEMBL3303080
CHEMBL3542366	CHEMBL367470	CHEMBL1213740	CHEMBL439048
CHEMBL4224782	CHEMBL3892613	CHEMBL458169	CHEMBL3650844
CHEMBL3771284	CHEMBL3462316	CHEMBL2035994	CHEMBL4639049
CHEMBL1978058	CHEMBL1974954	CHEMBL2115066	CHEMBL1482221
CHEMBL3137613	CHEMBL468629	CHEMBL3603950	CHEMBL2368677
CHEMBL4535954	CHEMBL2409745	CHEMBL435648	CHEMBL458824
CHEMBL582530	CHEMBL4513613	CHEMBL3394221	CHEMBL2068852
CHEMBL3234633	CHEMBL3221642	CHEMBL195290	CHEMBL3542188
CHEMBL131655	CHEMBL3650835	CHEMBL1172946	CHEMBL234105
CHEMBL294050	CHEMBL3729963	CHEMBL4291268	CHEMBL1254538
CHEMBL1025	CHEMBL1315122	CHEMBL422097	CHEMBL3329546
CHEMBL3808491	CHEMBL1966199	CHEMBL3895205	CHEMBL4515642
CHEMBL1714511	CHEMBL2420767	CHEMBL3915858	CHEMBL2230041
CHEMBL97035	CHEMBL585906	CHEMBL84420	CHEMBL138253
CHEMBL3245853	CHEMBL1965624	CHEMBL1585762	CHEMBL469660
CHEMBL1370000	CHEMBL3677272	CHEMBL4163700	CHEMBL1683547
CHEMBL462609	CHEMBL1163672	CHEMBL1684704	CHEMBL3221640
CHEMBL2325611	CHEMBL3701653	CHEMBL179005	CHEMBL164538
CHEMBL548113	CHEMBL2272370	CHEMBL4472568	CHEMBL2203381
CHEMBL4212576	CHEMBL3668940	CHEMBL1975762	CHEMBL1348593
CHEMBL1977371	CHEMBL1975608	CHEMBL3774797	CHEMBL1966134
CHEMBL4169915	CHEMBL4555224	CHEMBL1971012	CHEMBL1812543
CHEMBL3898110	CHEMBL4101156	CHEMBL111183	CHEMBL3932731
CHEMBL426578	CHEMBL386742	CHEMBL3668938	CHEMBL3487004
CHEMBL3415174	CHEMBL4090045	CHEMBL451937	CHEMBL290151
CHEMBL337683	CHEMBL3142428	CHEMBL116690	CHEMBL3702524
CHEMBL4447202	CHEMBL3276858	CHEMBL3668941	CHEMBL3557804
CHEMBL62214	CHEMBL241378	CHEMBL402328	CHEMBL1773655
CHEMBL1726653	CHEMBL3473156	CHEMBL1234118	CHEMBL4126492
CHEMBL1301397	CHEMBL4455499	CHEMBL4173134	CHEMBL191737
CHEMBL1698565	CHEMBL4278756	CHEMBL3138189	CHEMBL523489
CHEMBL396761	CHEMBL3398242	CHEMBL3655813	CHEMBL366250
CHEMBL3932452	CHEMBL91271	CHEMBL1985262	CHEMBL2203394
CHEMBL460806	CHEMBL3228271	CHEMBL2322945	CHEMBL1579535
CHEMBL1731974	CHEMBL1089118	CHEMBL462986	CHEMBL3647388
CHEMBL1997677	CHEMBL4580953	CHEMBL3137633	CHEMBL3484482

CHEMBL222680
CHEMBL3824396
CHEMBL3349453
CHEMBL449970
CHEMBL3892322
CHEMBL1214266
CHEMBL1491795
CHEMBL357533
CHEMBL1969924
CHEMBL93610
CHEMBL1964436
CHEMBL2429990
CHEMBL3935609
CHEMBL1864263
CHEMBL3452341
CHEMBL571494
CHEMBL4462678
CHEMBL3655781
CHEMBL104840
CHEMBL1812545
CHEMBL180514
CHEMBL4475855
CHEMBL1979180
CHEMBL110490
CHEMBL1774257
CHEMBL3680179
CHEMBL3195235
CHEMBL2429978
CHEMBL1499339
CHEMBL206753
CHEMBL577606
CHEMBL141714
CHEMBL1085580
CHEMBL1370929
CHEMBL3680993
CHEMBL3403133
CHEMBL49205
CHEMBL3272711
CHEMBL122925
CHEMBL3134508
CHEMBL3116053
CHEMBL3331132
CHEMBL4557988
CHEMBL1625579
CHEMBL1995617
CHEMBL3614035
CHEMBL4094977
CHEMBL3248228
CHEMBL122109
CHEMBL2178008
CHEMBL1715074
CHEMBL333248
CHEMBL2022084
CHEMBL1796703
CHEMBL2230044
CHEMBL1999240
CHEMBL4089403
CHEMBL3895911
CHEMBL144201
CHEMBL2153093
CHEMBL2377514
CHEMBL1077640
CHEMBL2348950
CHEMBL603223
CHEMBL4214876
CHEMBL2349204
CHEMBL4580307
CHEMBL113262
CHEMBL4583071
CHEMBL3343030
CHEMBL3461570
CHEMBL1208898
CHEMBL512641
CHEMBL3221641
CHEMBL1610518
CHEMBL3806003
CHEMBL2429970
CHEMBL4217348
CHEMBL2180774
CHEMBL1161210
CHEMBL2268607
CHEMBL1971264
CHEMBL351109
CHEMBL1277395
CHEMBL49736
CHEMBL113553
CHEMBL220187
CHEMBL3696959
CHEMBL4517324
CHEMBL1971239
CHEMBL1080588
CHEMBL4525360
CHEMBL2440479
CHEMBL4640604
CHEMBL329341
CHEMBL1570390
CHEMBL3972399
CHEMBL3195139
CHEMBL2006602
CHEMBL1989911
CHEMBL527224
CHEMBL2420755
CHEMBL3685664
CHEMBL1973714
CHEMBL3634482
CHEMBL4439353
CHEMBL1812526
CHEMBL3732132
CHEMBL4528880
CHEMBL1978480
CHEMBL1197204
CHEMBL402342
CHEMBL1378796
CHEMBL178975
CHEMBL320164
CHEMBL1999657
CHEMBL336952
CHEMBL1185151
CHEMBL2164549
CHEMBL1990577
CHEMBL1762730
CHEMBL1404000
CHEMBL3441911
CHEMBL134167
CHEMBL3702535
CHEMBL4299822
CHEMBL2007250
CHEMBL4096773
CHEMBL4587108
CHEMBL50285
CHEMBL4091185
CHEMBL509693
CHEMBL73334
CHEMBL4569256
CHEMBL4556094
CHEMBL1200733
CHEMBL87468
CHEMBL95490
CHEMBL3436915
CHEMBL3246125
CHEMBL4085240
CHEMBL477235
CHEMBL4531043
CHEMBL1883757
CHEMBL3349728
CHEMBL570121
CHEMBL596450
CHEMBL248505
CHEMBL4215774
CHEMBL91860
CHEMBL138520
CHEMBL3655799
CHEMBL3244467
CHEMBL4475896
CHEMBL1302895
CHEMBL3684924
CHEMBL67299
CHEMBL363147
CHEMBL1213132
CHEMBL367680
CHEMBL261402
CHEMBL1994840
CHEMBL504358
CHEMBL510635
CHEMBL2005228
CHEMBL2104707
CHEMBL3210714
CHEMBL1224465
CHEMBL1462254
CHEMBL367533
CHEMBL445390
CHEMBL3785465
CHEMBL3349706
CHEMBL3553201
CHEMBL1096762
CHEMBL4100441

CHEMBL92101
CHEMBL1254455
CHEMBL1079089
CHEMBL176097
CHEMBL1546101
CHEMBL3684925
CHEMBL1992649
CHEMBL1973651
CHEMBL1650469
CHEMBL4542157
CHEMBL458968
CHEMBL1311331
CHEMBL1650729
CHEMBL2296727
CHEMBL3785151
CHEMBL3983001
CHEMBL3192862
CHEMBL4585484
CHEMBL42008
CHEMBL3961300
CHEMBL1214267
CHEMBL3775734
CHEMBL433599
CHEMBL1935513
CHEMBL2205265
CHEMBL1780177
CHEMBL2336725
CHEMBL4096589
CHEMBL3691405
CHEMBL4531951
CHEMBL4281721
CHEMBL3639547
CHEMBL3728343
CHEMBL327842
CHEMBL177980
CHEMBL550899
CHEMBL106537
CHEMBL1650730
CHEMBL558334
CHEMBL2429992
CHEMBL1927329
CHEMBL539672
CHEMBL1616261
CHEMBL20307

CHEMBL1982000
CHEMBL1901588
CHEMBL477874
CHEMBL2385853
CHEMBL3810341
CHEMBL285500
CHEMBL3228162
CHEMBL3237101
CHEMBL490423
CHEMBL3680938
CHEMBL2207974
CHEMBL1337500
CHEMBL332234
CHEMBL206883
CHEMBL1098700
CHEMBL4460625
CHEMBL1087898
CHEMBL8010
CHEMBL4556555
CHEMBL3116056
CHEMBL593307
CHEMBL2024017
CHEMBL4558419
CHEMBL1360733
CHEMBL3958929
CHEMBL103789
CHEMBL4064408
CHEMBL1995739
CHEMBL3677273
CHEMBL253219
CHEMBL302780
CHEMBL3104324
CHEMBL3797236
CHEMBL160980
CHEMBL2021552
CHEMBL404999
CHEMBL4460060
CHEMBL483294
CHEMBL3657979
CHEMBL1459133
CHEMBL3651542
CHEMBL3581925
CHEMBL3966144
CHEMBL1430386

CHEMBL2006509
CHEMBL14411
CHEMBL3734048
CHEMBL3137557
CHEMBL241333
CHEMBL1410630
CHEMBL2207950
CHEMBL1503655
CHEMBL256684
CHEMBL3952827
CHEMBL165298
CHEMBL4574562
CHEMBL2304134
CHEMBL1585793
CHEMBL1957877
CHEMBL2377366
CHEMBL200131
CHEMBL1993019
CHEMBL414496
CHEMBL275767
CHEMBL87319
CHEMBL88020
CHEMBL1164221
CHEMBL3350937
CHEMBL469014
CHEMBL3248215
CHEMBL2371300
CHEMBL2089270
CHEMBL1981917
CHEMBL459250
CHEMBL307028
CHEMBL294005
CHEMBL1443003
CHEMBL3603907
CHEMBL2377515
CHEMBL1087373
CHEMBL337856
CHEMBL3981708
CHEMBL4589664
CHEMBL593776
CHEMBL1908916
CHEMBL321993
CHEMBL3557658
CHEMBL3753845

CHEMBL2235882
CHEMBL1982714
CHEMBL3470452
CHEMBL2016941
CHEMBL352227
CHEMBL3981104
CHEMBL1213577
CHEMBL4642935
CHEMBL57757
CHEMBL1209023
CHEMBL2057565
CHEMBL1338044
CHEMBL1741992
CHEMBL66601
CHEMBL3248216
CHEMBL2062899
CHEMBL2022237
CHEMBL3260629
CHEMBL4066645
CHEMBL63286
CHEMBL3098265
CHEMBL3698623
CHEMBL250933
CHEMBL3040314
CHEMBL456436
CHEMBL432253
CHEMBL4059578
CHEMBL75945
CHEMBL1209020
CHEMBL493313
CHEMBL2271386
CHEMBL2313680
CHEMBL192233
CHEMBL1492841
CHEMBL1087375
CHEMBL3354717
CHEMBL283997
CHEMBL3765205
CHEMBL4110262
CHEMBL14745
CHEMBL2114451
CHEMBL77442
CHEMBL2348948
CHEMBL2283525

CHEMBL4581022
CHEMBL3698749
CHEMBL4206842
CHEMBL338305
CHEMBL184047
CHEMBL3138458
CHEMBL593198
CHEMBL4088588
CHEMBL1221607
CHEMBL2272279
CHEMBL4467268
CHEMBL2272270
CHEMBL275472
CHEMBL4177016
CHEMBL1204125
CHEMBL2021568
CHEMBL226921
CHEMBL340496
CHEMBL1430705
CHEMBL208505
CHEMBL248497
CHEMBL3542289
CHEMBL4541452
CHEMBL1989071
CHEMBL3702520
CHEMBL2094283
CHEMBL1377709
CHEMBL381752
CHEMBL491435
CHEMBL3311125
CHEMBL1343543
CHEMBL334014
CHEMBL2079097
CHEMBL3982394
CHEMBL15910
CHEMBL3819116
CHEMBL2017344
CHEMBL4300199
CHEMBL1733948
CHEMBL3334540
CHEMBL117097
CHEMBL156856
CHEMBL319113
CHEMBL3133266
CHEMBL1867901
CHEMBL362032
CHEMBL2058887
CHEMBL4089504
CHEMBL3616857
CHEMBL2010900
CHEMBL30129
CHEMBL2230056
CHEMBL473511
CHEMBL3797871
CHEMBL233563
CHEMBL3085803
CHEMBL4281451
CHEMBL4582433
CHEMBL1269651
CHEMBL487270
CHEMBL3661679
CHEMBL2359648
CHEMBL1998915
CHEMBL4288516
CHEMBL1535671
CHEMBL511430
CHEMBL1999090
CHEMBL2004804
CHEMBL78395
CHEMBL1894696
CHEMBL3959593
CHEMBL1905774
CHEMBL1213864
CHEMBL297437
CHEMBL102602
CHEMBL3237330
CHEMBL2442178
CHEMBL4646127
CHEMBL3309925
CHEMBL55478
CHEMBL1969577
CHEMBL1205491
CHEMBL474323
CHEMBL68536
CHEMBL1159563
CHEMBL176667
CHEMBL1565121
CHEMBL2001178
CHEMBL510965
CHEMBL3786934
CHEMBL4086447
CHEMBL176944
CHEMBL2177479
CHEMBL3915217
CHEMBL4517464
CHEMBL3703269
CHEMBL1760545
CHEMBL121029
CHEMBL3246100
CHEMBL1466844
CHEMBL4457089
CHEMBL3394614
CHEMBL4168418
CHEMBL4568160
CHEMBL337210
CHEMBL554103
CHEMBL3925182
CHEMBL4648941
CHEMBL519717
CHEMBL514182
CHEMBL4117473
CHEMBL1994083
CHEMBL1927330
CHEMBL3925996
CHEMBL565262
CHEMBL3655838
CHEMBL1949717
CHEMBL2022061
CHEMBL54250
CHEMBL1916139
CHEMBL144551
CHEMBL3989498
CHEMBL104347
CHEMBL3248219
CHEMBL3913753
CHEMBL95184
CHEMBL1093010
CHEMBL1978912
CHEMBL3805966
CHEMBL1386444
CHEMBL130512
CHEMBL4517883
CHEMBL4104991
CHEMBL1992156
CHEMBL1834129
CHEMBL3936842
CHEMBL4175291
CHEMBL3137647
CHEMBL4214014
CHEMBL1325053
CHEMBL100223
CHEMBL4634674
CHEMBL4083235
CHEMBL1705539
CHEMBL3218239
CHEMBL292117
CHEMBL1996403
CHEMBL3632725
CHEMBL4554706
CHEMBL1164247
CHEMBL418814
CHEMBL607755
CHEMBL1586533
CHEMBL3684216
CHEMBL91575
CHEMBL353822
CHEMBL2376346
CHEMBL23981
CHEMBL3092353
CHEMBL1762746
CHEMBL4647885
CHEMBL60062
CHEMBL2377363
CHEMBL129851
CHEMBL133346
CHEMBL1897499
CHEMBL1870267
CHEMBL111849
CHEMBL259559
CHEMBL294308
CHEMBL3190273
CHEMBL4166348
CHEMBL2064252
CHEMBL574144
CHEMBL140819
CHEMBL3660592

CHEMBL133187
CHEMBL1165786
CHEMBL1435133
CHEMBL3960775
CHEMBL482943
CHEMBL4209249
CHEMBL1741942
CHEMBL3349747
CHEMBL595197
CHEMBL2348941
CHEMBL281328
CHEMBL3471600
CHEMBL1642722
CHEMBL397633
CHEMBL3237098
CHEMBL522009
CHEMBL1612739
CHEMBL4526588
CHEMBL2409837
CHEMBL2442536
CHEMBL1650468
CHEMBL487689
CHEMBL1323610
CHEMBL4098037
CHEMBL2135237
CHEMBL2079098
CHEMBL23271
CHEMBL2253329
CHEMBL1232805
CHEMBL601298
CHEMBL2106403
CHEMBL1997118
CHEMBL3246099
CHEMBL523585
CHEMBL1957472
CHEMBL2368687
CHEMBL3989188
CHEMBL107182
CHEMBL2017346
CHEMBL4639052
CHEMBL1080412
CHEMBL3330078
CHEMBL111555
CHEMBL357651
CHEMBL192016
CHEMBL4209425
CHEMBL3931404
CHEMBL3113540
CHEMBL314916
CHEMBL17537
CHEMBL4553018
CHEMBL2377373
CHEMBL205797
CHEMBL2068864
CHEMBL1780073
CHEMBL1088151
CHEMBL1199792
CHEMBL1988307
CHEMBL1575476
CHEMBL298526
CHEMBL360123
CHEMBL1991290
CHEMBL194326
CHEMBL176040
CHEMBL461945
CHEMBL1186121
CHEMBL572019
CHEMBL3808598
CHEMBL234651
CHEMBL1983029
CHEMBL2369023
CHEMBL1980697
CHEMBL188613
CHEMBL4456410
CHEMBL135193
CHEMBL3246097
CHEMBL3466298
CHEMBL150450
CHEMBL1982971
CHEMBL4587496
CHEMBL1178865
CHEMBL3680906
CHEMBL1978220
CHEMBL3451139
CHEMBL464691
CHEMBL4589845
CHEMBL1732715
CHEMBL4161650
CHEMBL3439291
CHEMBL1973052
CHEMBL2131153
CHEMBL1672272
CHEMBL2182016
CHEMBL1915636
CHEMBL2322944
CHEMBL4568559
CHEMBL194087
CHEMBL1084536
CHEMBL1974484
CHEMBL548097
CHEMBL281827
CHEMBL3466372
CHEMBL1858122
CHEMBL1604748
CHEMBL1711667
CHEMBL4561060
CHEMBL498222
CHEMBL3759863
CHEMBL4462041
CHEMBL1214342
CHEMBL1956785
CHEMBL2409753
CHEMBL3640645
CHEMBL130432
CHEMBL3655795
CHEMBL85973
CHEMBL566261
CHEMBL565189
CHEMBL1774275
CHEMBL3310823
CHEMBL1672288
CHEMBL447973
CHEMBL2377380
CHEMBL569875
CHEMBL406983
CHEMBL3899991
CHEMBL3962303
CHEMBL3248457
CHEMBL3969744
CHEMBL50022
CHEMBL453774
CHEMBL1927331
CHEMBL2022085
CHEMBL4116762
CHEMBL193406
CHEMBL3938261
CHEMBL2177506
CHEMBL1525098
CHEMBL3247956
CHEMBL1762752
CHEMBL513935
CHEMBL4165228
CHEMBL2420759
CHEMBL11455
CHEMBL472103
CHEMBL3191776
CHEMBL4082649
CHEMBL2115087
CHEMBL3703313
CHEMBL1324305
CHEMBL262149
CHEMBL4109968
CHEMBL158091
CHEMBL519811
CHEMBL3554371
CHEMBL3805347
CHEMBL568112
CHEMBL172763
CHEMBL1096761
CHEMBL1989439
CHEMBL3310822
CHEMBL1724490
CHEMBL1991458
CHEMBL3251641
CHEMBL367618
CHEMBL3104451
CHEMBL594485
CHEMBL2348967
CHEMBL74994
CHEMBL151997
CHEMBL426404
CHEMBL177726
CHEMBL458981
CHEMBL3746699
CHEMBL585951
CHEMBL4521041

CHEMBL483295	CHEMBL1161207	CHEMBL3137830	CHEMBL3634453
CHEMBL271678	CHEMBL512245	CHEMBL3581926	CHEMBL4062715
CHEMBL503392	CHEMBL3358844	CHEMBL3247955	CHEMBL4443587
CHEMBL4552519	CHEMBL169486	CHEMBL507898	CHEMBL3916298
CHEMBL304757	CHEMBL511785	CHEMBL1088152	CHEMBL1980132
CHEMBL1407910	CHEMBL3236032	CHEMBL1161206	CHEMBL1914577
CHEMBL1161211	CHEMBL572404	CHEMBL4070143	CHEMBL4213680
CHEMBL4578688	CHEMBL1803022	CHEMBL4112636	CHEMBL4107593
CHEMBL279824	CHEMBL1977057	CHEMBL4524833	CHEMBL3310824
CHEMBL2288591	CHEMBL3655796	CHEMBL467598	CHEMBL1098699
CHEMBL1375634	CHEMBL242809	CHEMBL1087374	CHEMBL203896
CHEMBL1812539	CHEMBL2335307	CHEMBL1160235	CHEMBL3248218
CHEMBL548190	CHEMBL2220663	CHEMBL2165262	CHEMBL3702522
CHEMBL4209784	CHEMBL3656326	CHEMBL1523799	CHEMBL3188332
CHEMBL3785817	CHEMBL385320	CHEMBL3786360	CHEMBL4167148
CHEMBL3309928	CHEMBL473029	CHEMBL2035007	CHEMBL75991
CHEMBL1994982	CHEMBL170666	CHEMBL3437297	CHEMBL2442537
CHEMBL1796582	CHEMBL1980015	CHEMBL3985967	CHEMBL1985210
CHEMBL248506	CHEMBL1415763	CHEMBL1086770	CHEMBL3986788
CHEMBL2048414	CHEMBL4167614	CHEMBL2332786	CHEMBL3972003
CHEMBL2008338	CHEMBL1480954	CHEMBL556428	CHEMBL3698625
CHEMBL4530643	CHEMBL554584	CHEMBL258256	CHEMBL332439
CHEMBL1956787	CHEMBL1876554	CHEMBL133852	CHEMBL1934081
CHEMBL4541623	CHEMBL3805960	CHEMBL4572201	CHEMBL3653695
CHEMBL1762731	CHEMBL610711	CHEMBL133870	CHEMBL1088732
CHEMBL4470668	CHEMBL3800017	CHEMBL3896898	CHEMBL2333397
CHEMBL2253356	CHEMBL494077	CHEMBL527424	CHEMBL2436548
CHEMBL2088980	CHEMBL3718927	CHEMBL3922846	CHEMBL4573180
CHEMBL4527341	CHEMBL558543	CHEMBL3246101	CHEMBL3702525
CHEMBL114308	CHEMBL2288590	CHEMBL3727899	CHEMBL1800575
CHEMBL4537263	CHEMBL4447250	CHEMBL1642719	CHEMBL1376784
CHEMBL393504	CHEMBL2447939	CHEMBL3640079	CHEMBL1516432
CHEMBL4593219	CHEMBL4640448	CHEMBL240017	CHEMBL1190202
CHEMBL1556282	CHEMBL2046993	CHEMBL293596	CHEMBL3798615
CHEMBL2134669	CHEMBL3701652	CHEMBL293753	CHEMBL4462879
CHEMBL1812506	CHEMBL4296902	CHEMBL1347743	CHEMBL63145
CHEMBL570002	CHEMBL1987098	CHEMBL584142	CHEMBL1975203
CHEMBL121253	CHEMBL312505	CHEMBL2017333	CHEMBL337369
CHEMBL1257	CHEMBL3797893	CHEMBL440336	CHEMBL1996546
CHEMBL1906987	CHEMBL2397970	CHEMBL153478	CHEMBL2370698
CHEMBL234478	CHEMBL2370191	CHEMBL3922487	CHEMBL167526
CHEMBL2115386	CHEMBL132772	CHEMBL3331131	CHEMBL2371735
CHEMBL4277626	CHEMBL3187456	CHEMBL3246093	CHEMBL84917
CHEMBL1968653	CHEMBL397056	CHEMBL4110944	CHEMBL3415387

CHEMBL501370	CHEMBL3403128	CHEMBL1907895	CHEMBL3248220
CHEMBL432514	CHEMBL520676	CHEMBL2205024	CHEMBL1983253
CHEMBL2104018	CHEMBL22245	CHEMBL1773765	CHEMBL251826
CHEMBL3499952	CHEMBL335968	CHEMBL3603918	CHEMBL1699799
CHEMBL156402	CHEMBL1208897	CHEMBL25971	CHEMBL1989452
CHEMBL372597	CHEMBL1430414	CHEMBL3439653	CHEMBL3114950
CHEMBL3085395	CHEMBL1076850	CHEMBL41517	CHEMBL235231
CHEMBL1878248	CHEMBL106137	CHEMBL2023245	CHEMBL369388
CHEMBL3116054	CHEMBL1377398	CHEMBL3814769	CHEMBL4113501
CHEMBL1728028	CHEMBL2068856	CHEMBL563916	CHEMBL139918
CHEMBL507182	CHEMBL3603915	CHEMBL3984439	CHEMBL524638
CHEMBL88248	CHEMBL194414	CHEMBL3809327	CHEMBL2289141
CHEMBL513127	CHEMBL172045	CHEMBL2017347	CHEMBL1098701
CHEMBL341888	CHEMBL2268930	CHEMBL3137835	CHEMBL1789399
CHEMBL4100706	CHEMBL278068	CHEMBL52638	CHEMBL452928
CHEMBL3394039	CHEMBL1992795	CHEMBL1209021	CHEMBL4646144
CHEMBL132476	CHEMBL1573628	CHEMBL3545420	CHEMBL207840
CHEMBL3604964	CHEMBL2228916	CHEMBL2289142	CHEMBL3462134
CHEMBL1966135	CHEMBL470330	CHEMBL3229287	CHEMBL335100
CHEMBL3415391	CHEMBL394175	CHEMBL466554	CHEMBL4074791
CHEMBL2165261	CHEMBL4639367	CHEMBL1778133	CHEMBL3554209
CHEMBL155696	CHEMBL2095075	CHEMBL91100	CHEMBL153068
CHEMBL1823722	CHEMBL4209207	CHEMBL468413	CHEMBL3740821
CHEMBL467774	CHEMBL227477	CHEMBL177180	CHEMBL213055
CHEMBL1214206	CHEMBL3730369	CHEMBL1773652	CHEMBL202400
CHEMBL4642801	CHEMBL3190818	CHEMBL3703731	CHEMBL3104323
CHEMBL1722482	CHEMBL4570439	CHEMBL24243	CHEMBL2172339
CHEMBL3349705	CHEMBL4525576	CHEMBL2377362	CHEMBL3650819
CHEMBL3752892	CHEMBL3623168	CHEMBL3425750	CHEMBL1496480
CHEMBL1878217	CHEMBL1087987	CHEMBL3967304	CHEMBL268542
CHEMBL128265	CHEMBL2097056	CHEMBL493314	CHEMBL1418081
CHEMBL179028	CHEMBL2136944	CHEMBL2429982	CHEMBL358917
CHEMBL4470324	CHEMBL1477237	CHEMBL11278	CHEMBL167982
CHEMBL1650515	CHEMBL1991796	CHEMBL121644	CHEMBL131152
CHEMBL381706	CHEMBL134222	CHEMBL2115088	CHEMBL2377368
CHEMBL1780180	CHEMBL1988309	CHEMBL2253327	CHEMBL127769
CHEMBL332183	CHEMBL3753063	CHEMBL2230046	CHEMBL383303
CHEMBL3198085	CHEMBL4126895	CHEMBL3970331	CHEMBL163242
CHEMBL1980017	CHEMBL4210404	CHEMBL120422	CHEMBL227370
CHEMBL4520272	CHEMBL3246092	CHEMBL2177912	CHEMBL602272
CHEMBL4166624	CHEMBL4579346	CHEMBL3441280	CHEMBL3695524
CHEMBL252692	CHEMBL113263	CHEMBL185946	CHEMBL3740855
CHEMBL3785932	CHEMBL1984550	CHEMBL276951	CHEMBL2409734
CHEMBL2429991	CHEMBL3969331	CHEMBL135138	CHEMBL163917

CHEMBL2333413
CHEMBL464874
CHEMBL2271389
CHEMBL3546543
CHEMBL3614037
CHEMBL3754504
CHEMBL376093
CHEMBL4546697
CHEMBL1328748
CHEMBL1532448
CHEMBL3703310
CHEMBL3461571
CHEMBL4531814
CHEMBL4287066
CHEMBL132310
CHEMBL1539110
CHEMBL1994403
CHEMBL4563890
CHEMBL1922540
CHEMBL1616692
CHEMBL1086641
CHEMBL1310091
CHEMBL4161267
CHEMBL4586381
CHEMBL494366
CHEMBL3542213
CHEMBL3542228
CHEMBL3596457
CHEMBL2008207
CHEMBL486046
CHEMBL1981684
CHEMBL171861
CHEMBL3809245
CHEMBL1558536
CHEMBL341766
CHEMBL1970959
CHEMBL3138005
CHEMBL175988
CHEMBL134277
CHEMBL1412775
CHEMBL2035995
CHEMBL2068854
CHEMBL2272933
CHEMBL4075503
CHEMBL1335867
CHEMBL1780179
CHEMBL3924478
CHEMBL2323852
CHEMBL1969849
CHEMBL2385783
CHEMBL1979003
CHEMBL52802
CHEMBL24963
CHEMBL1186156
CHEMBL3785428
CHEMBL1319601
CHEMBL3696102
CHEMBL3408058
CHEMBL466341
CHEMBL1790471
CHEMBL2131907
CHEMBL2006722
CHEMBL2030434
CHEMBL411730
CHEMBL3655840
CHEMBL3249046
CHEMBL3582316
CHEMBL538653
CHEMBL3805722
CHEMBL3554372
CHEMBL418061
CHEMBL52323
CHEMBL18162
CHEMBL3781426
CHEMBL1990066
CHEMBL371869
CHEMBL2017324
CHEMBL1735305
CHEMBL94836
CHEMBL4455293
CHEMBL3692934
CHEMBL4527019
CHEMBL2004312
CHEMBL194795
CHEMBL4565803
CHEMBL272273
CHEMBL3585645
CHEMBL1828951
CHEMBL435000
CHEMBL3542220
CHEMBL468379
CHEMBL513860
CHEMBL2385777
CHEMBL1257737
CHEMBL1991121
CHEMBL1985297
CHEMBL512758
CHEMBL1668924
CHEMBL4291289
CHEMBL497613
CHEMBL3137630
CHEMBL1983216
CHEMBL568551
CHEMBL477673
CHEMBL4564735
CHEMBL1461196
CHEMBL67942
CHEMBL1773767
CHEMBL3703296
CHEMBL447148
CHEMBL2236772
CHEMBL460808
CHEMBL3134277
CHEMBL1209025
CHEMBL4209769
CHEMBL2005126
CHEMBL2335297
CHEMBL3815006
CHEMBL4458436
CHEMBL35140
CHEMBL236728
CHEMBL192861
CHEMBL1197334
CHEMBL3928122
CHEMBL3647012
CHEMBL1350836
CHEMBL343098
CHEMBL1417591
CHEMBL4559932
CHEMBL3618496
CHEMBL3557065
CHEMBL271888
CHEMBL4574038
CHEMBL3668200
CHEMBL350912
CHEMBL4081500
CHEMBL449041
CHEMBL1682043
CHEMBL3729381
CHEMBL1973247
CHEMBL2336866
CHEMBL1510240
CHEMBL2272776
CHEMBL1903007
CHEMBL4541114
CHEMBL1458921
CHEMBL399028
CHEMBL1797384
CHEMBL4577528
CHEMBL3698627
CHEMBL1556112
CHEMBL331338
CHEMBL1784291
CHEMBL1990974
CHEMBL3984891
CHEMBL1164626
CHEMBL3113887
CHEMBL4545525
CHEMBL4577790
CHEMBL4461830
CHEMBL4078728
CHEMBL3234634
CHEMBL2386784
CHEMBL4446968
CHEMBL3817891
CHEMBL1389716
CHEMBL3787520
CHEMBL2172338
CHEMBL2429989
CHEMBL341379
CHEMBL574804
CHEMBL3702492
CHEMBL117188
CHEMBL3581931
CHEMBL117820
CHEMBL447851

CHEMBL203491
CHEMBL1499828
CHEMBL2113524
CHEMBL4293460
CHEMBL1198521
CHEMBL280658
CHEMBL3219981
CHEMBL176894
CHEMBL2420140
CHEMBL10938
CHEMBL4476538
CHEMBL1684715
CHEMBL425934
CHEMBL4286840
CHEMBL172988
CHEMBL2393378
CHEMBL3427499
CHEMBL2235628
CHEMBL4530842
CHEMBL3546263
CHEMBL2332879
CHEMBL1231750
CHEMBL4573310
CHEMBL3286553
CHEMBL3246094
CHEMBL397569
CHEMBL163117
CHEMBL2437105
CHEMBL4116962
CHEMBL4065182
CHEMBL335298
CHEMBL2105885
CHEMBL3797663
CHEMBL2429971
CHEMBL4081530
CHEMBL160018
CHEMBL4088432
CHEMBL1828953
CHEMBL584144
CHEMBL3907152
CHEMBL2112857
CHEMBL2377361
CHEMBL2062900
CHEMBL1093893
CHEMBL593810
CHEMBL194522
CHEMBL4127393
CHEMBL2376558
CHEMBL3957091
CHEMBL409749
CHEMBL4065995
CHEMBL2335310
CHEMBL3764228
CHEMBL587213
CHEMBL4637136
CHEMBL2420749
CHEMBL1934073
CHEMBL3827597
CHEMBL554120
CHEMBL3774501
CHEMBL367836
CHEMBL3116064
CHEMBL3354718
CHEMBL2177522
CHEMBL3805667
CHEMBL3286617
CHEMBL275215
CHEMBL3194606
CHEMBL1718016
CHEMBL4529559
CHEMBL2335311
CHEMBL2079083
CHEMBL464553
CHEMBL3471940
CHEMBL132880
CHEMBL351912
CHEMBL2236773
CHEMBL1589397
CHEMBL2057567
CHEMBL1988194
CHEMBL3647389
CHEMBL4101475
CHEMBL468630
CHEMBL392141
CHEMBL2430223
CHEMBL4467147
CHEMBL3547797
CHEMBL248094
CHEMBL515401
CHEMBL1605817
CHEMBL2036951
CHEMBL1976072
CHEMBL3394218
CHEMBL4558211
CHEMBL2272506
CHEMBL1990233
CHEMBL2003907
CHEMBL1502919
CHEMBL539657
CHEMBL3898129
CHEMBL1884611
CHEMBL3557493
CHEMBL3650829
CHEMBL255259
CHEMBL3355773
CHEMBL1375572
CHEMBL397055
CHEMBL1987012
CHEMBL2333416
CHEMBL3655782
CHEMBL3040318
CHEMBL4297648
CHEMBL1424596
CHEMBL299653
CHEMBL1760087
CHEMBL1529265
CHEMBL1301464
CHEMBL4228807
CHEMBL3261639
CHEMBL1708447
CHEMBL3797961
CHEMBL3787003
CHEMBL380775
CHEMBL3696112
CHEMBL3249039
CHEMBL4587837
CHEMBL1990509
CHEMBL1237184
CHEMBL1968082
CHEMBL9805
CHEMBL2051969
CHEMBL2203382
CHEMBL3918970
CHEMBL3526734
CHEMBL4544960
CHEMBL1974992
CHEMBL3650836
CHEMBL1972540
CHEMBL353640
CHEMBL2230045
CHEMBL4561327
CHEMBL3727622
CHEMBL3235952
CHEMBL2021560
CHEMBL3623167
CHEMBL3673949
CHEMBL1164754
CHEMBL1276654
CHEMBL4088884
CHEMBL2064257
CHEMBL3137990
CHEMBL1807898
CHEMBL1770350
CHEMBL4100920
CHEMBL1972870
CHEMBL4550292
CHEMBL2004260
CHEMBL3407847
CHEMBL1650725
CHEMBL4091105
CHEMBL175876
CHEMBL3235941
CHEMBL3989286
CHEMBL173263
CHEMBL140942
CHEMBL3637807
CHEMBL4595084
CHEMBL3496600
CHEMBL1304814
CHEMBL131633
CHEMBL1770349
CHEMBL312968
CHEMBL4444882
CHEMBL3742288
CHEMBL4286312
CHEMBL3818975

CHEMBL4545744
CHEMBL296439
CHEMBL3208744
CHEMBL491004
CHEMBL1583281
CHEMBL12528
CHEMBL316704
CHEMBL262395
CHEMBL110735
CHEMBL2371298
CHEMBL3677270
CHEMBL2381652
CHEMBL236389
CHEMBL3810274
CHEMBL4166678
CHEMBL4549987
CHEMBL3785240
CHEMBL2325638
CHEMBL4066149
CHEMBL1200694
CHEMBL3815145
CHEMBL1347477
CHEMBL4277007
CHEMBL4065045
CHEMBL2429966
CHEMBL3422740
CHEMBL1214341
CHEMBL494078
CHEMBL1765726
CHEMBL1979443
CHEMBL5008796
CHEMBL1207572
CHEMBL2114432
CHEMBL4579760
CHEMBL266010
CHEMBL135456
CHEMBL4215207
CHEMBL276375
CHEMBL3245121
CHEMBL287749
CHEMBL485879
CHEMBL8877
CHEMBL8467
CHEMBL395025
CHEMBL606446
CHEMBL57655
CHEMBL3246388
CHEMBL427457
CHEMBL1981020
CHEMBL8848
CHEMBL251224
CHEMBL4541853
CHEMBL2392401
CHEMBL4598412
CHEMBL3989804
CHEMBL1170075
CHEMBL1765721
CHEMBL272188
CHEMBL425673
CHEMBL3893497
CHEMBL2028080
CHEMBL4540800
CHEMBL563630
CHEMBL387582
CHEMBL3647330
CHEMBL29288
CHEMBL606708
CHEMBL2004103
CHEMBL417844
CHEMBL230164
CHEMBL1992317
CHEMBL1991417
CHEMBL293698
CHEMBL562893
CHEMBL1194828
CHEMBL486112
CHEMBL68527
CHEMBL4277911
CHEMBL230162
CHEMBL248408
CHEMBL1968867
CHEMBL2028089
CHEMBL4290812
CHEMBL29207
CHEMBL2104194
CHEMBL519291
CHEMBL230589
CHEMBL4909626
CHEMBL27964
CHEMBL78311
CHEMBL1205500
CHEMBL1078078
CHEMBL329689
CHEMBL481629
CHEMBL116898
CHEMBL1204965
CHEMBL161016
CHEMBL69099
CHEMBL1077615
CHEMBL9064
CHEMBL302031
CHEMBL4128572
CHEMBL116739
CHEMBL1978209
CHEMBL344557
CHEMBL135567
CHEMBL231418
CHEMBL1998259
CHEMBL8695
CHEMBL1981129
CHEMBL167966
CHEMBL3982465
CHEMBL230590
CHEMBL8974
CHEMBL2448178
CHEMBL2164464
CHEMBL104337
CHEMBL1984080
CHEMBL4102662
CHEMBL4282523
CHEMBL443611
CHEMBL1765722
CHEMBL3350682
CHEMBL1991379
CHEMBL1983953
CHEMBL230161
CHEMBL1206866
CHEMBL313478
CHEMBL226728
CHEMBL3137989
CHEMBL441014
CHEMBL440684
CHEMBL2005301
CHEMBL104056
CHEMBL4920785
CHEMBL5004210
CHEMBL1995937
CHEMBL4638856
CHEMBL3961247
CHEMBL424067
CHEMBL1968432
CHEMBL3138078
CHEMBL9068
CHEMBL1765723
CHEMBL542547
CHEMBL439191
CHEMBL399851
CHEMBL251967
CHEMBL135238
CHEMBL230588
CHEMBL4556044
CHEMBL2028079
CHEMBL2369356
CHEMBL3951138
CHEMBL287672
CHEMBL2093106
CHEMBL2028012
CHEMBL2092822
CHEMBL2115363
CHEMBL2009192
CHEMBL2028081
CHEMBL230591
CHEMBL3342184
CHEMBL4643074
CHEMBL1945345
CHEMBL2285806
CHEMBL606922
CHEMBL3352954
CHEMBL4284396
CHEMBL310061
CHEMBL4541720
CHEMBL3930208
CHEMBL1984259
CHEMBL502439
CHEMBL3273636
CHEMBL13725

CHEMBL183446	CHEMBL1797864	CHEMBL3250971	CHEMBL3039654
CHEMBL4468244	CHEMBL4793039	CHEMBL120050	CHEMBL4090968
CHEMBL3230509	CHEMBL192604	CHEMBL1971009	CHEMBL4163124
CHEMBL394540	CHEMBL1983889	CHEMBL4097338	CHEMBL1982974
CHEMBL3249463	CHEMBL4159077	CHEMBL3114543	CHEMBL1978207
CHEMBL4441388	CHEMBL1172576	CHEMBL1994914	CHEMBL1492669
CHEMBL1672430	CHEMBL474997	CHEMBL1973563	CHEMBL1970002
CHEMBL4571247	CHEMBL1900521	CHEMBL3793105	CHEMBL1989165
CHEMBL1991438	CHEMBL2005016	CHEMBL2138563	CHEMBL1977870
CHEMBL1462306	CHEMBL1982444	CHEMBL497813	CHEMBL1970156
CHEMBL4567273	CHEMBL1935096	CHEMBL2270085	CHEMBL3214527
CHEMBL3273015	CHEMBL3325775	CHEMBL2106852	CHEMBL4174275
CHEMBL2017189	CHEMBL2017888	CHEMBL2403750	CHEMBL1446522
CHEMBL143829	CHEMBL1967871	CHEMBL2437155	CHEMBL3618492
CHEMBL4451399	CHEMBL3086206	CHEMBL3899927	CHEMBL1709846
CHEMBL274120	CHEMBL1974459	CHEMBL2002229	CHEMBL1299546
CHEMBL1976613	CHEMBL257118	CHEMBL2238044	CHEMBL3217818
CHEMBL1539342	CHEMBL1993057	CHEMBL2027871	CHEMBL1572728
CHEMBL4514743	CHEMBL1439237	CHEMBL1971777	CHEMBL3629550
CHEMBL322225	CHEMBL3679684	CHEMBL3769545	CHEMBL148461
CHEMBL491430	CHEMBL113908	CHEMBL3922254	CHEMBL1303019
CHEMBL2203962	CHEMBL3091701	CHEMBL571098	CHEMBL1967661
CHEMBL4238436	CHEMBL1727460	CHEMBL243820	

S5.3 OTAVA structures

D7019052333	D7017440106	D7014791118	D7116411077
D7020070919	D1081052	D7014211377	D0107620825
D7020260402	D7720500152	D7211790148	D7020610029
D4802794	D7020649721	D6239824	D7119461554
D7015571248	D7119151192	D7210800099	D7715460139
D7020601250	D7012701390	D0128760137	D7019081194
D7020204413	D7114490510	D7012340112	D7714330221
D0129620419	D7211730625	D1039098	D1446137
D7020556856	D6054626	D7113670392	D7212090734
D7020532712	D7119390063	D1646484	D7012620885
D2833381	D7119832996	D7119790865	D7013941203
D7013830899	D1106079	D6057749	D0104640051
D1435604	D2193380	D7210430638	D1111910
D7016000803	D7014320326	D3458595	D0139990032
D7114830463	D7020530056	D1159737	D7012700003
D6668292	D0139960052	D1043513	D7116120027
D7217981381	D7013060352	D3619933	D0128680276
D7718980005	D1182363	D7114600898	D0129720236

D7016280173
D7020410038
D7119911007
D1762761
D7413920214
D7413810059
D1102159
D5835123
D1651289
D2033074
D7020070816
D1903305
D7020601175
D7010590338
D6328694
D0107490021
D6641974
D4800172
D7119630130
D0124140102
D0129663340
D1088253
D7110920340
D7211790408
D7020410080
D7117010016
D7020530667
D1034932
D0109280102
D0107340031
D7020390493
D7119831081
D7119850484
D7020617721
D7020593199
D7116460005
D1183102
D7017470060
D0103950034
D7013460368
D7112890249
D7020510162
D1098238
D1000199
D7119830092
D7413920239
D7020350295
D7119880224
D7020160517
D4790404
D0107210801
D0125240535
D7217930032
D7020470542
D0123380323
D7020550013
D0217330024
D7213390058
D7117350120
D1111624
D1467498
D7020420375
D7013540005
D7020460186
D7018731150
D7114090602
D0118780249
D0123400618
D1475786
D7114471464
D7020460012
D3458722
D7016870281
D1115213
D0124070072
D7115240034
D7216571007
D7016390281
D7013831462
D1114959
D7011060218
D7115710060
D7214580166
D11761005
D7119832028
D7017740874
D7020556979
D7014260079
D7012120269
D7212090605
D6242247
D7020721486
D1739108
D7015410210
D1102258
D7119631484
D0127443013
D7020431733
D7111620199
D7119850350
D1016409
D0105620184
D7212070028
D0129680063
D1033986
D0127500084
D1234075
D7016361495
D7119831771
D3498707
D7214590648
D7214540116
D7117330033
D1582045
D1563056
D7119740012
D0109420328
D1183214
D7110920747
D7117840594
D7214931446
D7216690197
D7119641451
D1140199
D1298118
D0128660176
D0107640042
D1436615
D0138120072
D0118671775
D7010130043
D7017470995
D7714300132
D1014801
D7714310012
D7216930005
D7020531804
D7012670938
D7118240174
D7119831893
D0105620249
D1054670
D7013860077
D7113690025
D1294037
D7119631198
D7119891469
D0109210053
D7017680286
D7712640009
D7119850787
D1482632
D0129620064
D7020691436
D7010120726
D7213780028
D7119812530
D0109370558
D7020601348
D7020521055
D11186089
D1902916
D1162208
D7119832965
D1758883
D7015410006
D0104840058
D7020060006
D7020090208
D7013870429
D1115318
D7020431845
D1234041
D7117170402
D7012621032
D11186123

D1309148
D7570449
D11649882
D4049676
D6298889
D7020260352
D3459746
D7110920781
D7216670239
D7119390001
D3460564
D7020649704
D0105460047
D1445795
D1655173
D5946914
D7119640646
D7559669
D7212580062
D7020603489
D4797467
D7017560328
D7020720876
D7114960004
D6052890
D7013530142
D7119850456
D7020151021
D7013191425
D6300011
D7020510002
D7020662066
D7020554401
D1111875
D0129510002
D1363547
D7119880860
D1164155
D6666649
D7016232920
D7216440636
D4802691
D7116170072
D7211800014
D1115251
D7018670040
D7115740019
D1030235
D7014760172
D7116410653
D1556458
D7211800123
D7413030072
D6243655
D7119891765
D7212090484
D0123400435
D2115235
D1090317
D4187372
D7119641182
D7015860190
D0117380128
D7020532420
D7020460203
D7016870304
D0129663983
D4250112
D7020540953
D4061570
D7014000157
D6244159
D1740026
D1446216
D0109750158
D7020070632
D1151690
D7119830239
D6057555
D7017470397
D1759000
D7014671049
D1489352
D1789056
D7020390397
D7020692944
D7210710019
D2630919
D11186707
D7110952669
D1347860
D1151845
D7018654739
D7210430487
D7119130060
D1014859
D6971276
D1359852
D7020556648
D7211760297
D0149430074
D1148572
D1740536
D7116080172
D7012700458
D7012702110
D7015030244
D7119610020
D7015121405
D7018811808
D7119832966
D7571006
D7563227
D6923282
D0108560564
D1305170
D0104500001
D0117370055
D2129115
D0107740005
D7020554824
D1107676
D7117091141
D11649930
D0109370647
D1603701
D0107070008
D0129660889
D7013891844
D7020300542
D7010120357
D1161804
D1787012
D7012120299
D6921583
D0127460129
D7114830560
D7720500779
D1108292
D0127460293
D1597866
D9079177
D2909599
D7119891284
D0128761072
D7016270405
D7116110080
D7014260275
D3851319
D1090419
D1312695
D7119930097
D7214540354
D7110950757
D7012740299
D7018880029
D7011520004
D1056717
D1534912
D0120630193
D7114471310
D0111370068
D5000150600
D7119760101
D2170670
D7216440886
D1263363
D7018801326
D7010170605
D7020431711
D5110070061
D7119691900
D7129824
D1158695
D1000454
D6052596

D1113846
D1004674
D1817602
D7020602389
D7211731053
D7020141027
D0129720684
D7020693553
D0138230390
D7020300893
D7117160163
D7413960693
D7112902103
D4401431
D7210430116
D7210742318
D7017470560
D7119290144
D5047246
D7020596418
D7019070266
D7013050083
D7020691403
D7212970107
D7118060101
D7119832960
D6241238
D7020431766
D7119561419
D7112490046
D0110490026
D5980805
D7111610008
D11186215
D7020556647
D7119830564
D7117320024
D1363177
D1109944
D7112901064
D7015052269
D7714330946
D0117890054
D1688350
D7013430217
D7116410147
D6297597
D7017440823
D7119891967
D7217970263
D7119987068
D0107520061
D0109780055
D5569469
D1619721
D7119330021
D4802829
D7118230236
D7020557132
D7020350127
D7214590538
D7714330895
D7562912
D1096093
D7111640215
D7116580513
D7119891531
D7013911425
D7017440306
D7018652285
D0125160302
D7016220002
D6238752
D1446119
D1330719
D0116270057
D1000886
D6237864
D0217270032
D7012340376
D7119910038
D7018770804
D7119760077
D0109750473
D7114510098
D0120630143
D7213330339
D1361748
D0125210066
D7020370081
D7020090756
D2934335
D7114600043
D7114520017
D7117810027
D7020470326
D7020550366
D0103230785
D7013941215
D1496702
D0109320066
D7212570016
D1016038
D0112190051
D6641970
D7017470584
D7110952631
D7017530061
D1055381
D7114960241
D3463559
D6243935
D7020531101
D7138690
D3215442
D1000496
D6080172
D4800911
D1780094
D7018780045
D7117840310
D0105850550
D6871429
D7020310234
D3353975
D7012620024
D7119480831
D1446226
D7016260742
D7011480003
D7018670049
D7011940161
D1348380
D7215380136
D7114551668
D981951
D7020602469
D3698308
D7013590616
D7118350099
D0217270025
D7020550042
D7020601067
D1104226
D7211731032
D7211810162
D7020350064
D1879920
D7012740051
D4791839
D7012702097
D7210740524
D1109327
D7015920558
D7112902061
D0111150115
D7119984945
D6052628
D7119920584
D1529747
D7016390794
D7015101941
D7020554707
D7011090003
D1025946
D7211730638
D0109420337
D7011440030
D5060473
D4802440
D5114510
D7018710850
D1006827
D1300992
D7013830870
D7017560928

D7014320241
D1729035
D7127317
D1180869
D7020618961
D7020554652
D0107620410
D7013050445
D0128760085
D7118062281
D7012740417
D4788177
D0109200029
D7116990130
D1392677
D7013590724
D9080053
D9078856
D6248759
D7214590457
D7110940230
D7114420272
D0127460306
D7013050377
D1572802
D7012180037
D0109080049
D7013050358
D7020556496
D0118650045
D1055867
D1159173
D7018860494
D7020431799
D6236579
D7014320632
D7216441034
D7020260231
D7020252439
D0125160003
D7213750059
D7110952662
D7020430939
D1191313
D1108967
D0109370312
D3679685
D7020210364
D7018958728
D7012270711
D2897178
D1655076
D0109290072
D7012730062
D7118460267
D7012180017
D7020554262
D7013870060
D7014261103
D7020542749
D7119850630
D0125290193
D1220690
D7020531962
D0105470063
D7020555998
D7016750042
D7016270164
D1797582
D0123750149
D7118460354
D7114490494
D7016220021
D7119850059
D0123751029
D7020618952
D7020662878
D7117940519
D7010120546
D7020410122
D7119880561
D2630859
D7020300043
D1059220
D5110070332
D1084232
D7015101776
D7212010516
D1316223
D7020366408
D7119691905
D7020557077
D1086972
D7126170
D7012270627
D7015150163
D7113230443
D7212090374
D7014750525
D7216620011
D4434605
D0108890419
D7018801343
D7212580171
D4014205
D1772150
D7011500051
D2195479
D7211740007
D7015920203
D7213330260
D7020595760
D7020070146
D7119832851
D6055524
D1056227
D5257226
D6246171
D6466175
D1178928
D1038879
D7020530675
D1202440
D7217180007
D4308657
D1090554
D7217960136
D0139900007
D3493668
D7013050406
D7211720245
D7015820162
D1342131
D5300300091
D7114090665
D1016272
D1047723
D7211730710
D5416435
D7119812693
D7014750417
D7010590341
D1499342
D0128660086
D4706218
D7015570769
D7012270814
D1112059
D7015102725
D7009656
D1011682
D7020470037
D6239131
D3336886
D1104224
D7020530669
D0115030777
D0127480173
D7020721760
D0129660994
D7020410020
D1746631
D7016280295
D1287416
D7562977
D7114551886
D1112277
D7020553631
D7012160043
D2085787
D1997447
D2927617
D1046028
D11186076
D7010476
D7012270095

D1446108
D7119680161
D4435318
D7014250403
D7020554253
D7218010559
D7012620943
D1178718
D6641936
D4383043
D0109790063
D7014750408
D2202711
D7216440052
D7020721571
D1113718
D1356856
D7111580200
D7217961251
D7214580128
D7010280049
D7114480028
D7211720792
D1685185
D1201260
D7119810836
D2051173
D6248245
D7216512309
D7020470396
D7211730687
D7216441701
D6247127
D7119630570
D2084454
D7510410200
D7012320034
D1094943
D7119531341
D7216920009
D1559120
D7117940508
D7014211282
D7112440015
D7118150845
D7214930561
D7214160310
D7212090876
D1366327
D7119480556
D7014791390
D2630811
D3644572
D0128680219
D1308762
D0115030567
D1298062
D1511321
D1089754
D1307008
D5010470
D7212090543
D2630922
D7217300124
D7018990570
D7212010664
D7116940132
D6238925
D7210480018
D0119470013
D7020410212
D7213780102
D2835176
D7020553897
D6054741
D7020390363
D7020531594
D7119920550
D7014320361
D7018770771
D7116080146
D7020710029
D0109210001
D3463463
D0105620406
D7012120295
D1180859
D7117870404
D7112410425
D7011070009
D1205465
D1586161
D3359598
D7211790298
D7113230918
D1444907
D7212090125
D7216090065
D0129620155
D7020365864
D3517625
D7020603052
D7018890442
D7118210482
D1364527
D4019994
D1821293
D7133176
D7119760300
D7212090263
D7020533070
D1090321
D7410330220
D7020520241
D7015180022
D7119681176
D7211690047
D7020431481
D7015870005
D7714780011
D1055319
D7017580141
D0103580280
D7212090045
D6245233
D7020620335
D2125333
D7013990282
D7011420065
D6239885
D4802716
D7412610188
D7015120629
D7135145
D6053550
D7212090839
D1444915
D7119920710
D7020602478
D7018959625
D7020580828
D7020666384
D7116170184
D6237756
D7113610001
D1123610
D4568615
D7214942874
D7020350427
D7119812615
D7214590055
D7117810048
D5459293
D1186280
D7013830334
D7018771064
D7119480826
D1481188
D7720500052
D1458173
D7020557094
D0115940036
D7020598061
D7114490092
D7020430735
D7413920006
D7019081247
D1328399
D4478592
D7016000439
D7115740061
D1037984
D3515834
D7715460440
D7020470584
D7020556395

D7119880354
D7013170487
D1372115
D7018610654
D7114830257
D7212090737
D7117160109
D6237515
D7010160113
D3521544
D7010010189
D7020542488
D0115080018
D7020540189
D7212090676
D0139940035
D0127443446
D7016350223
D7119832731
D7020530711
D7016232888
D7715040048
D6051775
D3463459
D7561485
D7119830132
D7118061246
D1085142
D11182094
D7011470968
D0112120193
D0117390818
D1046122
D7214140290
D1234061
D1444388
D7212580089
D0123400527
D1501193
D7020533027
D7569354
D7116990218
D0116470239
D6665740
D7020554070
D7013870406
D7117010066
D4792377
D7020350053
D7020430802
D1332020
D7020160478
D7211230304
D0125210266
D6298372
D7012120257
D6923205
D7017560335
D7114510666
D5662468
D7211230187
D7126866
D7011070096
D0149430056
D7013910821
D7119891439
D4013798
D7020430857
D7210440087
D2194301
D2148388
D1771630
D1057083
D7020204776
D7213330140
D0104720026
D1183245
D7020557147
D7020550427
D0105750036
D1151451
D1784821
D7019052129
D7016280138
D7020390376
D7020160394
D7020554264
D7117170415
D0123750268
D1688599
D7013070250
D7020600639
D0110520189
D6666753
D7013830818
D7014750677
D6244062
D0103331342
D0128660128
D7118060222
D1505378
D7013160122
D7861265
D0103350048
D7211970045
D6514357
D7020601392
D8653834
D7212060151
D7020598172
D0125240653
D7211790276
D7014660356
D1113483
D7119630165
D7018670057
D7131979
D1474103
D7119910890
D7712640003
D6328174
D7020580670
D7020410047
D7415110111
D7119480141
D7020604400
D7211720755
D7126723
D7020721248
D1090337
D5946909
D7020520544
D4061534
D7211730120
D0103540478
D7211180015
D1076329
D7020470156
D7114511981
D7020554258
D7211760135
D0118673158
D0124070071
D7020721744
D7012340141
D6465794
D7114890071
D7213340197
D0107620654
D0107700001
D1016870
D7020431961
D7119851560
D6246704
D7211760222
D7119812967
D7017560612
D6871440
D3644546
D7114880040
D7211750042
D7114551526
D7213330153
D7119341363
D1138262
D7020532085
D7013050605
D7015101405
D970482
D7016400786
D0110420197
D7020654134
D1048714
D7018880155
D7020580177
D7012340185

D7213330135
D7020532241
D7020691501
D7016390716
D7216950032
D7010120162
D0115030862
D0129663767
D7020591540
D7017440815
D7020554839
D11186069
D0108480080
D0108580164
D7020531178
D1798949
D7119611083
D7020431000
D1033300
D1056269
D7018811825
D7014671050
D7018810589
D6050984
D7012790084
D1055336
D5791454
D1114552
D7119130109
D1732632
D0103970121
D7013060750
D1087043
D7010080013
D7119811748
D1026749
D7018770978
D7217980444
D7011830573
D7020527903
D7013830858
D7020252457
D7510400009
D7013160004
D7114830570
D2213355
D1344856
D7015920538
D0108480066
D7011830159
D7211760290
D1298429
D7217973834
D7020554072
D0110490110
D7117170682
D7114090613
D7217982282
D7020554219
D7110952577
D11180503
D7015070166
D6641956
D7018860823
D0115540098
D7117840773
D7015880410
D1380382
D11186525
D7119691929
D1904105
D3354028
D7127080
D7020591101
D7210730025
D7014792055
D1596757
D0103420058
D1146588
D7010670101
D1049810
D5110090056
D7017560338
D7020556586
D1362075
D0117470039
D7510370042
D7015571005
D7119760028
D0105420039
D7018670061
D7020432298
D0125330361
D1331029
D4789274
D7010670125
D7214590177
D7110950771
D6668245
D7020532953
D7016000356
D7119981933
D7216910038
D7020430797
D7014750371
D1304149
D0107570121
D1120049
D7020554841
D7013070396
D7016350264
D6923320
D7114420399
D7114520032
D7117810089
D7119983086
D1107539
D7116940095
D7112901161
D7211760362
D2213576
D7016232944
D4802627
D7211760122
D7119800103
D1232933
D7118070121
D7119480733
D7012320434
D1298064
D7020554257
D0125200031
D0109290053
D7020491517
D0117370446
D7217972846
D7212580349
D7020592584
D1655127
D7020553986
D5786826
D7414620741
D1392570
D7020554878
D7560359
D2125469
D7018655951
D7018860693
D7020431815
D7020430658
D0105850570
D1272572
D7012340156
D0115090039
D7717710004
D7716300002
D0119470076
D7114830473
D5748440
D1113298
D7118330044
D0108610002
D7018990257
D7020363929
D7119740021
D0110420320
D7018850983
D7020480381
D0128630022
D7020691740
D7110920790
D1114737
D7014250196
D0129720708
D1033224
D1033217

D7215640004
D7119812652
D7020602506
D7114510373
D7138747
D6464360
D2792529
D7017480591
D1410541
D1047704
D7117170979
D0105470094
D7114830058
D7114510208
D7020430688
D7410930008
D7118280205
D7119880269
D7118600026
D7014260003
D6849450
D6236043
D7111630947
D7013980027
D0125270016
D7448960
D7020532579
D7020530166
D1113463
D7118510168
D0138950122
D7119641534
D7119681183
D6299551
D7133189
D7012270557
D7210470001
D7015450907
D7112892011
D0110420468
D7016220010
D1342563
D7015120216
D7110950228

D7016350280
D7570792
D7114510056
D7210770062
D7211730554
D7119692405
D3463650
D7212580156
D7213400113
D7020653773
D7013990337
D7014320477
D7010385
D5259736
D0114090050
D7413920220
D7212090808
D1870769
D7541258
D7020618039
D7119250460
D7210480069
D7413920209
D7018802044
D7138811
D7119910229
D7020690777
D0103420268
D7559301
D7214190350
D7020070428
D7213380086
D1366351
D7010000187
D7015990013
D0103560021
D1305174
D6666077
D7018771421
D7020410015
D7020531714
D0115520348
D7015920292
D7211720141

D0128680124
D3460099
D7012230018
D7016760088
D7020604287
D7218420346
D1097759
D7211230012
D1583890
D7020556742
D0118671613
D7117091210
D7020556715
D0118780191
D7012230261
D7216430009
D0103980089
D1741769
D7139016
D7020554422
D1006097
D1114155
D7017534404
D7112905285
D7020688456
D1618806
D7113225252
D7216660300
D7013161257
D1014770
D7119830423
D0108560069
D11186113
D7012700894
D7010570645
D7014211280
D7020402299
D7020720208
D1880316
D7020600736
D7020532838
D7116120034
D7569961
D4114592

D7111630107
D10177615
D1232972
D1001922
D7717490013
D7214191046
D7218310542
D7210440122
D7510350001
D0109750311
D1165118
D7012160050
D7016400574
D7020556613
D1110718
D7019081235
D0109770696
D4347292
D1762710
D1456317
D6053589
D1123453
D7712650064
D7011830378
D3463611
D7210770064
D7018670060
D7119831539
D7211990154
D0117381123
D7010620041
D7118062531
D0127500056
D7020533790
D5946936
D6236022
D7020366390
D1287914
D7118070126
D1015286
D7720500159
D7210741427
D7020553792
D6247681

D2046630
D7020580039
D7015170121
D7008458
D7015570309
D4802677
D2285152
D1468723
D4799219
D7013050308
D7015450890
D2824420
D7016261117
D7113260085
D1342149
D7015541484
D1482217
D1111413
D2429478
D7212580219
D7020293008
D4772953
D1687299
D7112420001
D0105850382
D1148371
D1108707
D7012270680
D7119880275
D7110950655
D1014767
D6543476
D7011830319
D3644608
D7020618953
D7020542463
D4802621
D11041339
D9080313
D7216508163
D0129720154
D0105850201
D6923301
D7020530015
D11186503
D7133058
D0111070393
D7119891860
D6466279
D7020520646
D7020531631
D3461887
D7116590198
D7020431890
D0118780197
D7119760078
D7113230650
D1655151
D7119640347
D7211760240
D6641943
D7115670006
D6237911
D7216690606
D7018890463
D7126996
D7119631298
D10026091
D7213330516
D7020554142
D1602981
D7119891479
D7119891880
D7119911300
D3458663
D0113020003
D4435170
D1182482
D7020610985
D7020594690
D7016870373
D7110920814
D7018801430
D7014250469
D6946310
D7014670487
D7014210434
D0118671819
D7129580
D1290258
D5984567
D7119880303
D7119620537
D1257278
D7012700855
D7012740018
D0111170167
D1113744
D7011950157
D7020510153
D1357938
D1202022
D7012780006
D5105558
D1156746
D7012740426
D1654201
D1257905
D7215330004
D7011500045
D7216440029
D7018822400
D7013161724
D7020720997
D7013830892
D7013911758
D0109770118
D1217693
D7011880033
D7020721176
D5030040664
D7020260037
D7119880164
D7561176
D7114600602
D7010640279
D1121232
D0123751057
D7114510716
D7020603304
D7020610170
D7020694393
D7214540195
D7020310168
D7020580523
D0108420254
D7016232100
D7010170530
D7016210959
D7012700309
D1782966
D7020550612
D7114960219
D7212090983
D0111070134
D7210710065
D7010270063
D2168231
D7118352164
D7112420155
D7018766689
D0105480012
D4801307
D7113670296
D0105850388
D0108890730
D1035289
D7561030
D0127441751
D0139030013
D7013060576
D0113310012
D7113260344
D0104640235
D7112870278
D7010000198
D11186511
D4790346
D3463089
D0128680116
D7112906316
D1158592
D1977052
D7116590261
D7119561954
D7210430278

D9958099
D1301802
D7127224
D7570899
D4311232
D7012120247
D6328102
D7126917
D6053544
D7118173597
D2191000
D7020370021
D0109160109
D0107320517
D7020510029
D7020591981
D7119831109
D0128680020
D0115030444
D4434563
D7119691978
D7010000346
D7012320013
D9080240
D4790426
D7110940275
D7017570249
D1015049
D7119892191
D7020300786
D7020557065
D7020532526
D7020431565
D7020402298
D7011420062
D2125383
D6641929
D7126787
D7720500047
D7119620645
D7217300316
D7216571624
D0104230307
D7114960231
D7015880425
D7114830624
D6247647
D7117940041
D7111640093
D1202095
D7119480016
D1468857
D1006387
D7114850299
D7018930043
D6922926
D1974332
D1510684
D1661007
D1000297
D7020602448
D7116080029
D7113630050
D4061518
D0105710066
D7020361154
D7010040018
D7116110221
D0127480024
D7214540228
D7015150360
D7211760298
D7018801408
D7117080128
D0107320387
D4061559
D7020556581
D4814455
D7119930157
D7019081245
D4434524
D0125228726
D1342155
D2244430
D7119151217
D6238148
D7020350517
D11186594
D3671265
D6923476
D7212980003
D7020674251
D3355543
D2582794
D7139183
D7015510484
D1523198
D7217972793
D1039086
D1042017
D7413520089
D7016233535
D7017655172
D7217961237
D7020352221
D6666079
D0129720083
D1131018
D7020600204
D0117410090
D0115540642
D7114471115
D7110940355
D7117830086
D7017480136
D0111070341
D1043659
D0115950101
D7114420145
D4683386
D1000153
D7565942
D7211760467
D6244545
D7014670996
D1733330
D7012700002
D7561908
D0127460257
D1182905
D4347314
D7114040271
D7011460503
D1469064
D7119620050
D7020594691
D0109420332
D1901306
D0120630206
D7110920507
D7215630200
D6426493
D1006710
D7020260422
D7713880199
D7118460600
D7118560552
D7110952507
D7020533169
D0109080040
D7118350444
D7217972613
D7410330235
D1359721
D0103420080
D8916320
D7214590013
D7012780027
D1006716
D7020555917
D7116150013
D7119832989
D7020593477
D7115610014
D7119691913
D0117940046
D7013160767
D7119480693
D7129625
D3356389
D7119480778
D7020204747
D3463628
D1367449
D3851334
D0107050091

D7117160129	D7020532598	D7119880184	D7119692000
D3606877	D7020553444	D9079193	D6328581
D1392694	D7020580290	D7020604025	D0128760728
D1392602	D3311203	D4402653	D0109280060
D0110830297	D7013830930	D6328586	D7211731211
D7011460525	D1375628	D7211720391	D7561221
D1508553	D0123400526	D7020532907	D7020531647
D7119832971	D7119631505	D1095389	D1301717
D7116410257	D7010387	D7217970705	D7111620136
D7119380123	D4798168	D7015120438	D1115317
D7114090787	D1000335	D5008590	D7119850129
D7114480026	D7020721620	D7571101	D7119380012
D7011070053	D1183330	D7016390796	D1400608
D7020610097	D1060583	D7015880239	D4802697
D1296017	D1344573	D7020361400	D7714330798
D7217970062	D2125387	D7012701773	D7211720676
D0103540364	D6210187	D7110952681	D7020596581
D7217980095	D7214170024	D7015880070	D7018771297
D7020720105	D3688279	D7020530636	D7215630180
D7212090589	D6329870	D7138626	D1621223
D0118670483	D7010000341	D1714625	D7020554670
D7210430355	D1005950	D7119832959	D7216950037
D7119691404	D7014751122	D3487484	D7218000008
D7114490403	D1032376	D1232879	D1176981
D7012701396	D1347371	D7569876	D7020554280
D7214190556	D1349046	D7216420010	D7114510453
D7020470057	D7020550608	D7020610178	D2033086
D0108320037	D7014670173	D7020721638	D6923362
D2104173	D7570597	D2069898	D7017470475
D7114430362	D1906408	D7015150049	D5110090010
D0104820585	D0108890312	D7012740475	D7013870547
D1202027	D9713233	D7114420380	D3353963
D7020210354	D7020550063	D7010120184	D7010640276
D0104990019	D1088549	D7217982321	D1182787
D0104560271	D7011100520	D7010620051	D7112420180
D1113489	D3463404	D7119891614	D0149430076
D7119691995	D1032331	D7119480906	D7020530314
D1824552	D7020593426	D1651156	D7018611210
D0139950106	D7020556483	D7119760240	D7212980011
D7014320334	D3808544	D7012701119	D0118671826
D7214540243	D7013110145	D7013830248	D7020460177
D1111259	D7215260005	D7211730947	D7014750843
D1468704	D1303971	D7119341291	D7020430608
D7116170168	D7013940789	D0107780080	D7116940046

D2205677
D7018670058
D1309356
D7119983556
D1482211
D7117170526
D7018890486
D7212090955
D7561234
D7553443
D7410930001
D7018912201
D4789943
D1112345
D7020350059
D5662426
D6934889
D1688681
D7212960014
D5681569
D7119844551
D7115710045
D7020102421
D1475755
D6465704
D7118340129
D2630873
D6239927
D6466304
D7214590067
D7117940181
D7111640171
D7110950152
D1196972
D7011510018
D0110420479
D7015010662
D7020532489
D7139215
D7213380072
D7717730017
D1267216
D7020526835
D0124080017
D0119470011
D7117940524
D0118671830
D999741
D7119561957
D1975659
D5980509
D7111630158
D7017470341
D7211730693
D1087054
D7118170761
D7559450
D7014250415
D7127098
D1442191
D7119891512
D7117370284
D7133170
D7117170522
D7011060136
D0108480002
D7014710144
D0103580221
D7119610207
D7020556820
D1446027
D7110940282
D7560077
D7118240267
D7015880078
D0104640024
D9958143
D7014260777
D0129720551
D7119989515
D7010010015
D7114500074
D7020591059
D7020431870
D1119873
D1118634
D0105810090
D7570766
D7020532957
D7020430673
D1150260
D7015020396
D7116580154
D6248919
D1159404
D7415110159
D7119341272
D1304853
D7119880887
D7119630152
D1392619
D7020710067
D7116410214
D7211760081
D7217970193
D1038055
D1088453
D6044409
D7016310403
D0109810012
D7119691861
D1740276
D7114980069
D7016350039
D3463357
D7020593437
D1044033
D1298042
D7716340010
D7020300138
D7020160106
D1192753
D1705455
D7015030088
D1123754
D1173363
D5635621
D1301496
D2073336
D7210770051
D7020555956
D7011410106
D2191620
D7214931157
D0109790127
D7119360829
D7114490541
D7020531841
D7119911528
D1103140
D7110940382
D7010260085
D1468632
D7212580068
D0108560243
D1603903
D7020594071
D0103420086
D0108890264
D7012340363
D7559101
D1446165
D7020470198
D7563605
D1447712
D7020554457
D0149430037
D7119891474
D11186058
D1164646
D7119720005
D7216690433
D7113221772
D2189991
D1121230
D7559776
D1398882
D1102985
D3466491
D7013490313
D7012340386
D7112810010
D6248164
D7020593541
D7119680431
D3627931

D0125320103
D1762837
D1033357
D7119831788
D7020554316
D7119831402
D6057577
D7013831172
D1325776
D6241588
D7119691638
D7211760493
D7020160558
D7014250177
D1121226
D7017470355
D7214590075
D6327712
D7020604318
D7212090427
D7119830367
D1196714
D0139960035
D5995682
D7011460032
D1112048
D7020431051
D7020557151
D5159615
D7020618356
D7114470059
D0125330588
D7020430629
D0125790090
D7020490385
D1304034
D7014710244
D7110310011
D7020470342
D3064193
D7216571124
D1158232
D7020690663
D0105670032
D7133109
D3458750
D3851577
D1365449
D7118061665
D7218011027
D9078800
D1859778
D7211790483
D7714780004
D7114551660
D0125230090
D7020470573
D7211810371
D7114601467
D1446161
D7010160004
D3477200
D1046209
D0110510071
D1156764
D7116410565
D6236648
D7013540456
D1112284
D7020620704
D0129610013
D0123750160
D7010000256
D1007209
D7017441880
D1301116
D7211790322
D0112576027
D0104660006
D7011460718
D7112891252
D7211790229
D5005932
D7118020039
D0107760145
D7020532723
D4797328
D0109450034
D11186664
D7020721590
D1098425
D11180507
D7020431726
D1822984
D7020554491
D7018890437
D7015101566
D7213330112
D7013830944
D0104010035
D11640173
D3463474
D1158345
D6298341
D7112890052
D7211180014
D7014370035
D7020532661
D7020556374
D7510420064
D7010210030
D7020692999
D7111630266
D7018850064
D6465888
D7560698
D1108437
D7119630692
D0103580100
D7118060339
D7020593339
D1196946
D7210770050
D7714720260
D7020554404
D1446086
D7115210012
D3346952
D7020556260
D7020470072
D7114600345
D7016000914
D7020601136
D0113050026
D7119812440
D9080679
D7013530151
D1445963
D6921837
D1591581
D7213330454
D3460196
D0119670002
D2831945
D7111630190
D7210430154
D7115750010
D7216050107
D1605432
D7119681145
D7015930014
D0128761234
D1033337
D7114830535
D0107210828
D1071403
D1110541
D7119760254
D7114010408
D7015860134
D6668105
D0109280012
D7117330021
D7114490429
D7119640864
D0125800042
D5014482
D7017571169
D0129620434
D0104820388
D7020401558
D7216930002
D7020693934
D7016390402
D7017470664
D7211730667

D7018771019	D7110320008	D1760292	D7216430003
D1708415	D5969932	D1288293	D0128761503
D0104550307	D0109820016	D7020630350	D7013540959
D0107840038	D0111030414	D1112250	D1557844
D7020380002	D7414620649	D7020390504	D4569737
D2630801	D7008240	D9080664	D0138120085
D7012740235	D7119911468	D7015160123	D7211730310
D7013870044	D7570152	D7018670063	D7019052382
D7216441010	D7011830183	D7413030046	D7213330068
D7211730476	D7216430019	D1183114	D7011080347
D7016000822	D3507352	D7011960005	D7119851677
D1035209	D7114490505	D1112294	D1436762
D7010570743	D1156730	D7012701219	D2134220
D7113703023	D7119520592	D7020530931	D7212980002
D0118673128	D7214910063	D7020601447	D0107770079
D6053360	D7020671251	D7014750863	D6246220
D7014750508	D7020490309	D1217722	D6921838
D4490119	D1782538	D1204359	D7020210417
D1182455	D7013870326	D4667147	D7018770001
D6246959	D7217970015	D0123380217	D7013590756
D7014320659	D7119692004	D7020260064	D7010620254
D1671248	D7216430018	D7214190420	D1072574
D7212090953	D7715460194	D7715460083	D7714330284
D0128660166	D4794773	D7020532920	D0104840052
D1588492	D1578246	D3530251	D7212090443
D7016382646	D7014750814	D7720500085	D7020554923
D1156741	D11181225	D2044920	D7113260031
D7010288	D7014370030	D7018670052	D1115204
D6464252	D7211680021	D7020542821	D7414620212
D4250260	D7015990117	D1048439	D7013980218
D7127652	D7110950825	D7135234	D7212090046
D7212090857	D7020533512	D7011460522	D1655181
D1341977	D7018880251	D6248793	D0109790012
D3463314	D7114830598	D1864385	D5230690005
D7020533978	D1581291	D1118622	D7011470878
D1446151	D10054808	D0117470024	D7011010106
D7012740169	D7211790266	D7011010155	D7119330063
D0104580063	D7020692336	D7119692197	D7012320332
D7012320363	D7020390181	D1107057	D7020556591
D0128761053	D7018655936	D7212090626	D7113232069
D3691051	D6923429	D7020600045	D7015052300
D4799649	D7216690582	D0109820023	D7113260249
D7020141071	D0129660783	D999800	D7115250030
D7020350046	D970183	D7017430416	D2151761

D7020554139
D7569933
D7114900432
D7020490022
D1111705
D1112107
D7210430309
D7020520993
D7111620069
D7211290023
D7216440085
D6850009
D7013060756
D7011450002
D1085959
D7010600052
D1017087
D7020201454
D7020430848
D0104000077
D1763100
D7020692746
D0107210266
D7713527
D1365432
D7020380020
D0127480015
D7113680030
D7112905893
D1182415
D7211210044
D7117330117
D7016270218
D7113670363
D1000135
D7216850075
D7119870080
D7020556783
D7113260400
D7114551369
D7020721549
D7013831076
D6668399
D7126039
D7119480812
D7117940498
D3619968
D7112870262
D7119681131
D7020530251
D7010150085
D7015970142
D7019050248
D7113222028
D7015101660
D1580580
D4355612
D0138280132
D4787315
D7015011023
D1112017
D6057957
D1112033
D7013010743
D7013870421
D7118120118
D11186574
D7567692
D7119812636
D7216440028
D7119610430
D9078875
D1182740
D3627935
D7214540318
D0118640012
D7013910838
D1149836
D0108560013
D7012120020
D7117170336
D2202722
D4016766
D5980475
D7020350017
D7015500184
D7020070785
D4500788
D7217970345
D1392595
D7020556840
D7211970042
D1107293
D1328385
D7014740317
D4794360
D7020570030
D11186085
D3637885
D1587208
D0129661059
D0109870025
D6241476
D7211730207
D7215270117
D7110950335
D7119590051
D1362104
D7020580449
D4789856
D5946931
D1392702
D1114791
D7119880634
D0104170017
D7020350006
D3908681
D7010080017
D1045711
D7119831965
D7112471255
D7012720023
D7020554351
D1364924
D1752553
D1287067
D7020533883
D11186644
D0115090133
D7014713247
D7413120421
D2833074
D7119910022
D1373956
D7211730777
D1060087
D0125190022
D0129720488
D7013010835
D0123750145
D1151701
D1482334
D7020470119
D5005274
D1737362
D7020532795
D7714300115
D1337637
D1122741
D7119832077
D1330759
D0107320524
D2125346
D1435431
D1232814
D7119760066
D7013990215
D7014320098
D1086796
D7110960092
D6923428
D7013830838
D7212970014
D0125340036
D1055427
D1113970
D11639958
D7119151212
D0104720033
D7114490511
D995716
D7019081241
D7117310056
D7018652110
D7122656
D0139910051

D1677929	D7133182	D7114490075	D5980490
D0105010069	D7020533825	D1517666	D7559848
D4673002	D7119987976	D7012270102	D7020550353
D0110870044	D7210710006	D7114420506	D7016350053
D1003152	D7712640076	D7214590312	D7020091019
D7020350120	D6668545	D8916342	D7118351105
D0129570031	D7018990393	D7119832037	D7012120078
D0109790056	D1445699	D7135505	D7119891621
D1115338	D0107210251	D7020300880	D1445784
D7114420176	D7020554238	D8014438	D4059514
D7012320382	D7020430843	D7010280065	D7011960044
D6466595	D1300745	D4053337	D7020350128
D7020260722	D1456700	D0111150064	D7014000168
D2084310	D7020331326	D7570037	D1685637
D0105480015	D1285115	D0113430142	D0115120650
D4034047	D0115090149	D7119691402	D7217970868
D1104234	D0129710204	D7020460457	D7210430923
D7112897857	D1394643	D7119631477	D7119792259
D4435284	D7113270190	D7119760056	D7715560117
D7020532274	D7114010575	D4798297	D1150479
D0111240005	D7128104	D9080308	D7113214313
D1271012	D7116940124	D1090359	D4060632
D7020430631	D7118110077	D7020540043	D7114010488
D7012790061	D4802966	D7118481222	D1043355
D7013070026	D0105651080	D1179414	D7013010833
D7119610200	D7020470554	D6057025	D0124040092
D7020532901	D1035282	D7015870006	D0107760152
D7119790128	D7020554244	D1867227	D7119880569
D1306737	D7212090101	D7113230664	D1717934
D7114830681	D0127443287	D4059337	D7119880139
D0123751236	D5441566	D4802140	D7119851396
D7214590379	D1112336	D1287021	D7015020057
D1108973	D7014750164	D7117100146	D2442671
D7018766255	D1049815	D7212580356	D7012180019
D7017560034	D7413130502	D1342115	D1727131
D7020331315	D7020556936	D1526199	D3356467
D7117370368	D7020260356	D1146992	D3703092
D0103620022	D7018770739	D7119910774	D7212010339
D3340579	D1006974	D7117090141	D0138120040
D7020366415	D7014370002	D6932228	D0114300102
D1344515	D7020430934	D4347296	D2194650
D7117320039	D7020533066	D7020410204	D7210430502
D7020601233	D3360039	D3458598	D7020430729
D0105850161	D5110070073	D0149860053	D7017430575

D1157984
D1333478
D7119480725
D0125340047
D1043362
D7012701744
D7213400688
D7119831919
D7020602688
D7211250050
D11186722
D7020550032
D1841552
D0110490104
D7211290028
D3194247
D7014760401
D6236309
D7565760
D7020555856
D1717063
D7011060190
D1312052
D0109210162
D7212580235
D7127309
D7212570240
D7016280097
D4570835
D7020530996
D1159209
D0127441861
D7020160312
D1112263
D7116980088
D7569869
D7016350249
D7016323638
D1484762
D7119611148
D1158288
D7217221649
D1049584
D7510780114
D1182404
D7016232243
D7216660759
D0109370328
D7119692063
D7119880765
D7020050002
D1159367
D0129663858
D1446039
D7216470013
D7114980046
D7114600411
D7017550546
D6244561
D1113413
D7217970797
D0104500022
D7217970333
D7216441037
D7117170226
D7211810314
D7216101125
D7215700198
D3678667
D7119911464
D7117171150
D0128680063
D0109420113
D6244658
D7112905864
D1001531
D1347145
D0118670900
D6236066
D7114890067
D1811331
D7014660015
D0125290200
D7020526967
D0104820073
D7020390281
D1477780
D7119630666
D7413920205
D7020410189
D7118080030
D7020532991
D7020410067
D1106122
D1757315
D7211790551
D7112830014
D7013170333
D7117090728
D1043193
D1056946
D7118600571
D7019051076
D7020532871
D7119480913
D7018880190
D0107310017
D7020556599
D1572686
D7014260151
D7018971607
D1740543
D5152569
D0113050007
D7138820
D7114011359
D5110090063
D6240725
D7011010133
D4434731
D8027221
D1183056
D7119641977
D7118062021
D1389017
D1178464
D1043074
D7018771279
D7211730787
D0129620370
D7119880251
D1059549
D0105820617
D7020550478
D1394136
D7119610734
D7119691477
D7114520026
D0138220023
D7126156
D7558453
D7557982
D7014791043
D2473821
D1115536
D7117840756
D1318813
D7116410004
D7215400038
D7020720533
D0117390120
D7211760262
D7010650056
D7016000397
D7114600717
D1361131
D5103222
D1472477
D7013940618
D7018654856
D7020553385
D7561859
D3509315
D7020721038
D3485520
D1196296
D1392691
D7110950837
D1737355
D1264816
D7110940550
D1433875
D7015070164
D1118518
D7020721729
D1305623

D0117391182
D1243653
D7113231535
D3463632
D1446190
D0118670884
D0115540321
D4403532
D7020693112
D1328571
D7138821
D7113232096
D1065720
D7018860272
D1779968
D0129720052
D7020597995
D7114010567
D7020720942
D1005712
D7020430771
D1059487
D2305939
D7014400395
D7020510166
D7020554964
D7119691352
D1007164
D0138070013
D7110950332
D7114020026
D7011060023
D7116580407
D7013050021
D1150868
D7211730853
D7019050094
D7119831482
D7114490332
D7119680331
D1043517
D7119691797
D7217970031
D0109280050
D1162745
D7119831463
D7135498
D1090608
D7018780018
D7020160568
D1180049
D7211720715
D7119891825
D7010150091
D3463194
D7020520454
D7119831846
D1437679
D7115680069
D0115080009
D7015052293
D0110830136
D0112430079
D7211760401
D0109370794
D3851342
D7138712
D7015550288
D0105620209
D0138230246
D0112820087
D0110950003
D7566775
D0104940004
D2195890
D7013430270
D0104640459
D7014790241
D7119830224
D0129660552
D7018720623
D1308808
D1862840
D7013831547
D2216661
D7119911058
D7017560732
D0125180167
D7020692755
D0129720203
D0109420208
D7020530132
D4788569
D7570662
D1287411
D7211750061
D2204975
D1411023
D0109070087
D0110420194
D1314510
D7119984114
D7014250140
D7010010185
D7216100428
D7020070756
D0217270022
D0109420105
D7020431046
D7114601648
D7113214836
D0109070104
D7117940530
D7014710436
D7114600508
D7018611186
D7014670684
D7020527001
D7016890043
D7010170281
D7012620684
D7017430356
D1311741
D4347305
D7119760185
D7020721822
D0138230374
D7020601231
D7013831427
D7414240186
D7119860042
D1125142
D1655050
D7119985271
D7019081213
D7714230044
D7114830054
D1496756
D7119986377
D7014750379
D4434705
D7112903950
D2174330
D1369904
D3684923
D7210710004
D7020143419
D7117230424
D7011000008
D0138120020
D7117171082
D1056617
D1113325
D7412300033
D5270560005
D7020532058
D2832269
D6463761
D7211790503
D7119631447
D7013460401
D7119620475
D7119892231
D1501192
D7114420217
D7211730263
D7210730047
D7216690382
D0104800663
D1392733
D6667738
D7014750434
D969963
D6246658
D7013050346
D7210480009

D7133202
D1112011
D0107630057
D1097959
D1521866
D6240024
D1781371
D1664738
D7119911579
D7210440052
D0104840048
D7015150009
D7013160536
D1000114
D1573818
D7714800340
D7020533438
D0138070019
D6240151
D7011940644
D0123750886
D7014750808
D7114020011
D1123615
D7020530855
D0115080158
D7114490418
D1446074
D7020430695
D7020470199
D5980529
D7020240325
D2824896
D7011060114
D7015990151
D1435600
D7020720195
D7020601150
D1088027
D7413030025
D1291637
D1001272
D7119641439
D7013910074
D7119911093
D1112609
D7020140947
D7118560784
D7114420303
D1002318
D7111550211
D1628372
D1111812
D7117010178
D6057851
D6626166
D7119891748
D1159408
D7020594676
D7020688140
D7010240317
D7214910245
D7714330013
D7112905876
D7210500073
D7216520058
D7013941168
D7020533368
D7012620015
D0111070345
D1170841
D7212090039
D6329859
D7116411086
D0128680080
D7714300152
D1186247
D7119880067
D0118670356
D3644507
D7020390502
D0129660834
D7112890153
D1556456
D7020594506
D0115950160
D7020555593
D7113230604
D1436854
D7113220802
D1048146
D7119830174
D7217250050
D1470093
D7013050394
D7569360
D7011830387
D7119631228
D1118512
D7213330052
D1973512
D1313347
D2095958
D7016390834
D7119692339
D7012701879
D6327731
D7119520512
D7119740007
D6052899
D6594982
D1304932
D0108420200
D0107620424
D7020470226
D1001839
D7110952480
D1195896
D7213330535
D7119982117
D7020591155
D7714330095
D0108850457
D7020521053
D7213400123
D0109770169
D2630917
D6052905
D7117090803
D7117940575
D0111370144
D7211240054
D7020590829
D7020431042
D0129720335
D6641960
D7020720933
D6052903
D1782985
D4791628
D7714230343
D2824881
D7017550060
D7017560495
D7119760184
D3463846
D1975232
D7110920994
D1446283
D7119641582
D1043201
D7018810795
D1114132
D1100767
D1368496
D7020591108
D7013060707
D7011460256
D7122648
D7020410347
D7119891789
D6125735
D7020240400
D7119832951
D7015102162
D1284190
D7013460033
D7020532286
D7020531504
D1437731
D2971506
D7112420292
D7119830894
D1715120
D7020350319
D7217980158

D7214540048
D3459440
D7112900080
D5980541
D1763445
D1159756
D1320738
D7119891491
D7010260180
D0120630100
D7211270010
D6328868
D7542028
D7012320688
D1112103
D4885837
D7014750845
D6300641
D7211790111
D7013870261
D1006470
D7020580661
D7117840816
D7020531858
D7119983250
D7116411080
D1037943
D1048759
D7117160190
D6246901
D0109420029
D6666880
D7510410178
D7119610374
D7111580089
D0105820709
D5300300078
D7119800052
D7416250002
D3353948
D7005253
D7019080356
D1646611
D7116410321
D1183334
D1109089
D7118350433
D6668526
D7113260366
D7012780018
D0105660524
D7115750091
D7118470249
D7020693140
D7214180039
D9080257
D0125226326
D3851327
D1043951
D2204921
D7213350002
D7018704001
D7413030022
D7011960062
D7213330362
D3526390
D7119630657
D7020510050
D1446152
D0129720475
D7113231958
D7128114
D7020618394
D7013050533
D1059240
D0109450038
D0115540271
D7214590318
D1276814
D7020470389
D7111580128
D7110952492
D1182835
D1446069
D7116030280
D7113213161
D1456787
D1125630
D7012740141
D3644616
D0127460272
D7015860145
D7213330451
D7012180038
D7020556167
D7118292296
D7020410096
D1170757
D7110950280
D7118350069
D7020431696
D1007076
D7017470434
D7563448
D7119811042
D1106602
D1114377
D7010383
D7020531110
D1052305
D1483281
D0105850675
D0115030052
D1249453
D7017690447
D7020533349
D7013831360
D7015410137
D7116411112
D7117350034
D7217960130
D1055428
D7118460331
D1132641
D7020602683
D0117380385
D7210430046
D7015180169
D1202019
D1445881
D7114480011
D7119800041
D1250929
D1367855
D0110420473
D7020554741
D7119670422
D7020692954
D7113706514
D1781207
D7118351219
D1316198
D7016390798
D7020554455
D0127480027
D7020090044
D7020602825
D0115030324
D0109870120
D7010170572
D7214590504
D7020557171
D7118460347
D7114090080
D7012270847
D0109770141
D1321089
D7011830418
D2050751
D7013190394
D6246066
D1367727
D1215706
D7216450107
D1197701
D7714330253
D0105620117
D7114601527
D7018801314
D0129620206
D7020601095
D7017570582
D6052776
D0119460024
D0115080012
D1721085

D7112880091
D0115030468
D7015920311
D7020601490
D7119250338
D7020556842
D3575002
D7020260925
D7010260052
D7116410549
D7018890478
D3585926
D7015570244
D2980217
D7020300895
D0107910001
D7020460405
D7015880270
D6247032
D5416410
D6241575
D7119610740
D0109770306
D7211720218
D7119891449
D6665645
D7015592565
D7018655341
D7117100124
D7712650010
D1949217
D7119982567
D1003178
D1017022
D1115383
D1647327
D7018801290
D7119480363
D2190301
D1513491
D6668110
D7138650
D0107950121
D0128760893
D7714330233
D7020361232
D0118673165
D0139880074
D7119520239
D7020595919
D1343314
D7119830940
D7011510729
D7014260011
D4388583
D0124040203
D6466436
D1106711
D7211800091
D7114601906
D7012320599
D7020430921
D7020532624
D7020366418
D1204413
D7720500142
D7217971352
D1472996
D7211750079
D0115570002
D0139960042
D7011460877
D7116410566
D7117170530
D7210430883
D1250923
D4467643
D7217961295
D7020202127
D7020530514
D7014750794
D7015120542
D0115950114
D1719647
D1482245
D7018860399
D7020556890
D1140204
D1112255
D7012230163
D0129660567
D7017570540
D3704059
D7112500002
D0109280006
D7119880213
D7018850139
D7119810498
D7020604289
D0108420517
D7014210714
D7020720291
D7020533104
D1465395
D7210480084
D7020310257
D1006166
D4327185
D7010020375
D1199110
D7213400602
D7113214000
D0104990004
D1105085
D7118030881
D2251106
D1115045
D7020204764
D7011080286
D7019080408
D1196891
D7020430078
D1041088
D7114500052
D7013050023
D7211730107
D7114880049
D7012340035
D7211760247
D1752966
D5080400612
D7114490389
D0108420021
D1341999
D7114490492
D7020550444
D7118350236
D4788147
D7117160134
D7013161009
D1055897
D7015592624
D7018700311
D0112250065
D1392714
D7111590054
D7119910437
D7020580555
D7117171139
D7020260127
D7010200038
D0124080037
D7020431715
D0107200030
D4569325
D0138280143
D5946922
D1456746
D7020410286
D7130053
D7020595873
D7011460769
D7117170846
D7018762801
D7011060167
D0129720190
D0139960060
D1115049
D0139900044
D7020470167
D7214130019
D1446062
D7016000540
D7119560686
D1025983
D7013460335

D1115186
D7017530026
D1465164
D7020693100
D6244634
D1305276
D7016382232
D1361313
D7020160771
D1200751
D7126867
D6053428
D7020300665
D11181177
D1301721
D0107050081
D7215660013
D7020554796
D1655160
D7110920557
D1087918
D7020300826
D7017802040
D7116410212
D7020300626
D7216050069
D1446364
D7562870
D6236015
D7714330894
D7218100494
D6052223
D7010130053
D7012620610
D0127480051
D7718920112
D1066548
D7119831375
D7114430248
D0125230163
D7559485
D7020260049
D7212090738
D7119911606
D0112790558
D0138060014
D7415490002
D7212090455
D6245724
D4793541
D2187999
D0109070014
D7070707164
D7119880822
D7020554225
D7020260074
D7119985496
D7568141
D7013050043
D7412610165
D7020594623
D1000435
D970595
D7112900761
D6510071
D7017690514
D1748737
D7118210561
D7020601073
D0109070016
D2227670
D5976645
D7119851658
D6239020
D7119430089
D7117390126
D6057962
D7013831644
D2207175
D0118670931
D11649928
D7015150424
D7013941088
D0109750322
D7020550361
D11186728
D7119831614
D4789521
D7011420036
D7119930025
D7020690639
D11186570
D7131704
D3341632
D7211750048
D7020430916
D7020555646
D1182395
D7013161733
D0127500075
D7020601729
D7713040332
D7013060563
D0111070004
D1600084
D7018810537
D7020260516
D0107830098
D6850475
D7020410331
D7117160133
D1344711
D7020530880
D4795849
D7715560092
D7020470170
D7020410092
D7119561561
D0138240166
D11181162
D5662494
D7119760001
D0110420381
D6170988
D0115530251
D7214590243
D7012740387
D6902356
D7118340033
D1655186
D7214910070
D7017690322
D2151598
D7212090188
D0129660662
D7110920909
D7510791464
D1016151
D7112897497
D7018890475
D3353952
D7012270454
D0127500151
D0105480023
D1162185
D7018880317
D7015820166
D1118741
D7119760168
D7020300901
D7020540716
D7210710040
D1736230
D7020692963
D7018611264
D7119831548
D7112900117
D5152469
D7571114
D1446056
D6244592
D7013050149
D3644489
D1278077
D1205793
D0128680003
D7020555007
D7111550215
D7020090009
D7020721884
D1301572
D7017471295
D1759854
D7714331157
D0128761075
D7020160112

D0107710075
D1182541
D7020410068
D1018656
D7020553394
D7114090976
D7120000015
D7020580469
D7010120302
D6667166
D7013460642
D7111640078
D7116680021
D0109870002
D7020531728
D7020610415
D3354067
D7020527401
D4048999
D7559621
D7020721499
D7119480917
D7119610067
D1217021
D1298433
D7138597
D7415490027
D3931984
D1121291
D7020431182
D7117320001
D7013860064
D1357990
D1112049
D8840403
D7020556765
D0129663830
D7012701685
D7013460684
D0127430013
D7112893443
D7211330009
D7217973665
D0116700101
D7212090494
D6465783
D7119680270
D7119844595
D7131390
D0109160009
D1340284
D7210430140
D6309252
D0125228776
D7020531664
D7119631170
D11186080
D1180029
D7114830617
D7020430986
D6918360
D7012270400
D7119831087
D1169443
D7114090484
D7019081246
D1368556
D2103980
D1289884
D0107000006
D7561273
D7218310376
D7010000456
D7117170050
D7020490804
D7510792676
D1182926
D2049285
D0127500110
D7020580394
D0104640076
D7013050128
D7139382
D7119980358
D7013870349
D7013110120
D7020510086
D7020532223
D7212090128
D7119831534
D11186640
D7013891433
D7111630079
D4795217
D0105670106
D3391515
D0125210466
D7018890422
D7118351765
D7010620158
D7119700290
D7119832936
D4341866
D7139140
D7015121506
D7211790185
D7118061773
D1901307
D1112183
D0107100006
D7013050513
D7563011
D1112622
D7715430001
D7013070393
D1019028
D1059643
D7112901145
D1456172
D2195805
D7217180010
D1975651
D0127443455
D7020530721
D7010600144
D7119630717
D7020553133
D0111000904
D6057977
D0103960009
D1655157
D7020300908
D3463413
D7119561835
D7119812862
D7020533342
D1767247
D7010000252
D7113701111
D6492540
D7111620026
D7413930375
D0107210247
D7119830135
D5662480
D7119670162
D7020520701
D7013870050
D7569591
D6426512
D7011100545
D5506333
D7119880548
D6056605
D7020210102
D7413120704
D0109770183
D1445750
D1048435
D0125340290
D7714330216
D7016000819
D7117940527
D7011510809
D1209746
D6667002
D7020603478
D6849463
D7020593478
D7119900024
D1113725
D7116410736
D7018860401
D7020721804
D7119250329
D0107640062

D7020260909	D7020460064	D7020440001	D0105010043
D1109192	D1183594	D7020460439	D7563124
D7118560563	D4034690	D1006819	D1043081
D7133031	D7111550054	D7214590433	D0109750297
D7011100011	D7020350013	D7020410066	D7015880214
D7131769	D1151224	D7119930093	D7713930015
D7117940018	D7117320239	D7714331189	D7018860528
D7540176	D7110950102	D7117330209	D1159499
D7018810575	D7413920240	D11649874	D3463847
D0107490008	D7113232454	D7114100137	D0127460234
D7211230310	D2192716	D0118460010	D0217330027
D4434692	D1176671	D7211190030	D7714780010
D1418510	D5031877	D1120021	D7020430828
D7020410384	D7010240403	D7119730912	D7213750200
D7213330079	D1103199	D0115090289	D7020556137
D7020431965	D7119300148	D7020141069	D0119470058
D7020532648	D3708752	D2630914	D7213330021
D1782049	D7559978	D1295275	D0103960032
D7015010011	D1088044	D7020361170	D7118080191
D7020370042	D6053541	D7119630569	D7020604015
D2042879	D7020532232	D7020526860	D7114601339
D7210430839	D4044659	D2630893	D7112905871
D6466645	D7214170045	D6236556	D7012270971
D1904680	D1614786	D1216168	D7014711041
D7119880131	D1031521	D1455343	D2837427
D7015920580	D0138160026	D7213750083	D7120000036
D7212030026	D7011460915	D0116100020	D4434506
D7117170957	D7118510215	D1220755	D7020431954
D9078753	D7292439	D1433832	D7119630627
D5914049	D7018611189	D7110300005	D7119480558
D6246319	D7010240087	D7119891475	D7210430742
D7210770072	D1514169	D6236070	D6236044
D1033418	D7020532618	D7720500049	D4787242
D6466775	D0115540623	D1182369	D0115950698
D6921833	D1290554	D1301183	D7119230011
D7119471541	D7013830419	D7015571527	D4801369
D7020601265	D7213390029	D1372400	D6052605
D7119911097	D1437694	D1114574	D1639536
D7020682554	D7119691622	D0111370137	D1059746
D1348904	D7216690061	D7211690065	D7214560046
D4795484	D1450969	D5429045	D7020691677
D1769053	D0124100015	D7015900001	D1006208
D0105500031	D7020593401	D1000131	D7119680296
D3462673	D7016000908	D7020672156	D7113260360

D1016829
D0115090263
D7020550490
D7119831966
D3485516
D7119880066
D7020600040
D1058701
D7020530786
D7118560220
D7020180185
D0112842107
D1055921
D7020601212
D1098960
D7214590217
D1907226
D0115520513
D6299600
D7119880222
D7126289
D0107780022
D7110390048
D6236679
D0109370393
D0103680027
D7212960003
D7020300409
D1003161
D7215660005
D7020554240
D0118640079
D0107510027
D3145910
D6057560
D7118510271
D0125160217
D7020603929
D0128760724
D7020580529
D7212970038
D4195337
D7010240001
D7011460438
D7010280588
D7119850620
D7017560122
D3644621
D7118150810
D7015900161
D7020350381
D5778902
D7012180032
D6327893
D7016000555
D7020601062
D7210540052
D7111590003
D3620042
D0125800099
D1317180
D7017570225
D7714330036
D7016310764
D0127460455
D7013941172
D7016390486
D7020526951
D7114011366
D7015910111
D4802504
D7119880216
D1108734
D3461694
D7014320079
D4787302
D3677935
D7211730626
D6922920
D1459041
D7111630015
D7119870046
D7210740146
D6244378
D11186053
D1748183
D0129620310
D7119692049
D1311314
D4788284
D1344702
D7020526889
D1446052
D4797645
D7111630138
D1196297
D2127982
D7114601036
D0109420221
D7562958
D7020260769
D4788359
D0127500157
D7020593448
D0103930006
D7214190878
D7119520724
D7018991841
D7011510426
D5000640734
D7112890152
D1739826
D7014400446
D7139119
D0112590007
D7020720144
D2129101
D0139030143
D7211790385
D7213340089
D1094720
D7020530063
D1045436
D3851315
D7012620998
D7110950792
D7020340102
D2115422
D7020431667
D7111640042
D7020554972
D7008499
D7119460473
D7020470058
D7118351759
D7118340479
D4043576
D5946928
D7020663785
D0109420183
D7020460353
D7014670870
D0115540631
D7015500159
D5270560025
D7020470605
D7119692349
D7117160114
D1178891
D1010432
D7020470352
D0109420002
D1114922
D1195418
D7020721585
D1755602
D7018704069
D2934055
D7020363931
D0110830233
D0111150003
D0107880013
D7119988686
D7020531705
D7018770808
D0110420213
D5791609
D7119390113
D1617944
D7119880113
D1366593
D7119560892
D7020530856
D3489298
D1046027
D7020556789

D1112030
D1113705
D0109780002
D1773894
D7110921010
D7116580541
D0109200016
D7214590401
D7116410436
D7119870102
D0111070146
D7127635
D0105850172
D7013890553
D7111640162
D7210480348
D7020721647
D1468677
D7020141329
D7119986301
D4676992
D2908664
D7114890186
D1369991
D0103420267
D7112908339
D7217930100
D7015860209
D6667735
D1182612
D7111630374
D7013940884
D7011930076
D7211790471
D7011520005
D5728989
D7020340077
D7211760105
D0108480036
D6239870
D7020370049
D7715460550
D7114830521
D1348946
D1517662
D7016000616
D0105620399
D7110952486
D7020532780
D0115950217
D7017550360
D7113706011
D7020598014
D7128959
D0129720145
D1304174
D6299505
D0112800067
D1901321
D7113231954
D1484745
D11180510
D1463339
D7211760349
D5010333
D1111924
D3513246
D1118905
D7211690112
D7020720672
D7211760328
D7119800079
D7020720651
D7018800734
D7211790052
D2630870
D7215400013
D1903198
D1112135
D7110940406
D7126681
D1993883
D0115540499
D3644671
D0128760343
D7119860139
D7020720936
D0125160275
D6052652
D7214190913
D1618995
D1720542
D1098517
D7020070812
D7020470126
D7010000399
D0123380218
D7114490542
D7213330261
D7210800105
D7110950905
D7211810336
D1647320
D7115790041
D7013540741
D7020160785
D1908023
D7020160637
D7017760706
D7211730194
D7013830941
D0105280005
D7020618606
D0108850003
D7011000017
D1090193
D7020601016
D0129490018
D7119880393
D7013831400
D0107070115
D7112894395
D1149524
D7117840579
D5066406
D7015570496
D6667937
D7014320306
D6246939
D1646419
D7013910003
D1347306
D1365114
D6237492
D7112905567
D7214540080
D1097946
D0103970184
D7020431855
D7018811856
D7011510735
D7217300240
D1112302
D7016310261
D1444435
D7020554752
D3015489
D0139990017
D7211790084
D1056509
D7213330029
D7020532367
D7018801398
D7118070222
D0104900061
D7135256
D7011460972
D0127430218
D7020553506
D7018630359
D1562215
D7415480325
D1183333
D7020300133
D1056710
D7211730356
D7113630074
D7020550110
D7110950560
D0139910053
D7017690218
D7112897803
D1501942
D1655168
D7119630734
D7020260202

D7013940808
D7570805
D7020300598
D7013460278
D7714330042
D7013460038
D7011960134
D0117381020
D7014210459
D1191936
D1159729
D7211740029
D7119911261
D6665801
D7020410118
D7119642080
D7020710002
D0138140038
D7116460058
D7119860207
D7216930014
D1807395
D6051004
D7012720029
D7020160330
D7119832133
D7110950773
D7211731120
D7119850784
D7117320175
D1676639
D7020470277
D7020350521
D8916333
D4482403
D7011450014
D4790541
D7020553517
D7016362010
D7119860032
D7020390515
D7212090736
D0105620171
D1365492
D7211720204
D0125290508
D5918060
D6248972
D7010120731
D0117380082
D7020527357
D7015580101
D7117390619
D1705454
D7013831572
D3698725
D1901610
D7015920272
D0103970050
D7600172
D7020691349
D7116990046
D6329862
D7111640001
D7013990065
D1032290
D3355777
D7216420049
D7119200004
D7126879
D2113996
D1682234
D3628756
D7119760180
D1033383
D0115030332
D7216044944
D0139960017
D7020542473
D7020721559
D7014791127
D0115520597
D7020533803
D0129720448
D7020430822
D7020721709
D7020431998
D7011110305
D4005684
D1309897
D3485669
D1112582
D5974518
D7010250205
D5110070287
D2235743
D6069526
D7013191266
D7013910010
D0115030042
D7015550285
D7110950232
D7006898
D5008415
D1514513
D1294041
D7020380058
D3511227
D6463668
D3924002
D7127138
D7010270020
D1436707
D7119730796
D1828567
D0107620052
D7213390054
D7012740415
D0129661010
D7119880175
D7020160429
D1146540
D1347840
D7020556651
D0117940008
D0149430001
D1003219
D7543451
D7119851571
D7013170354
D0107180006
D7570657
D7117370210
D3459400
D1033331
D7020596722
D7116460007
D7020690091
D7119641327
D7128105
D7018800964
D4469309
D1351246
D7014670725
D7560736
D7119985091
D7020554283
D6465769
D1434695
D0107070148
D7119832051
D7416250185
D7020554979
D1217725
D1101683
D6465875
D6299481
D7119680050
D7119620468
D7020204743
D5980429
D0104820145
D7020527422
D1037140
D7020490234
D7012701563
D6246638
D7011460567
D7020520294
D7018850276
D7119851641
D7510370099
D7020430619
D0104970100
D7020557015
D7116410443

D0115540526
D7016260157
D7118350759
D7020530106
D0109450003
D11186706
D7012790068
D1445721
D7014610526
D7020590569
D7020554898
D7011940322
D7119891272
D7015860117
D1365633
D4050430
D7114050100
D7013070317
D6463696
D7117940490
D7018660016
D0109780015
D7020590828
D7217971284
D0125226296
D7012270088
D4347300
D1033356
D0117470004
D7020350382
D7117840934
D7020430761
D7119880363
D1587179
D7570645
D1003339
D1347610
D6850250
D2206373
D0118671579
D1467649
D7117940704
D1036396
D0109080069
D1522504
D7110952649
D7020720178
D7020602728
D7118020035
D0104220006
D0104910353
D1433848
D7119831448
D3644579
D7011090026
D7020202035
D7116060019
D7114520016
D7012701728
D0127460494
D7010650151
D1763397
D7014750596
D7715560126
D6328397
D7119831397
D7010170628
D0109370086
D1121286
D7113214856
D7020555101
D7020557118
D7110940302
D7020601871
D7017430179
D7571167
D6057123
D0127460496
D7563415
D7214910031
D7020380040
D7020602918
D7133193
D1072923
D7217970087
D7012130195
D1559697
D0107320358
D6057054
D7020530840
D7017550249
D7020532695
D7111640175
D3463443
D6299392
D7210800203
D0109070101
D7117840502
D7012690080
D1507075
D7211730849
D7114471161
D3461329
D7020556816
D7013010696
D0129580033
D0108890574
D1287068
D0115030546
D0107760141
D7020532644
D1446100
D1513355
D0104000018
D7210800299
D1060187
D1098379
D3463553
D7015880237
D7214590568
D7212580023
D7210510138
D9078839
D7110920811
D1295725
D7020310195
D7119986110
D3684014
D7020552990
D7210440130
D7114880046
D7015120568
D7119610873
D7017480797
D7113213405
D970069
D7720500045
D6135746
D0111290005
D7012320105
D1209324
D7014610600
D7114830285
D1287431
D7211750040
D7018971608
D7110940296
D7012730073
D7214920003
D6849452
D7210450027
D7018770796
D7119891534
D7135550
D2125319
D7117940536
D6156142
D0115080075
D7010980047
D0116270019
D1086134
D7119691660
D7211270043
D7211390033
D7119760209
D7020691034
D7714330858
D1007051
D7011870015
D7013940114
D6466606
D6641961
D1305493
D7712640012
D7114900586
D7014210585

D2113886
D7015101926
D1114177
D7117231057
D1309853
D3461474
D2125390
D1096638
D7020580513
D7014250220
D1007112
D3851307
D0127460546
D7018660030
D1754042
D0125160125
D5976654
D7010170426
D0103540311
D7019081232
D7215260007
D7113220446
D7013540797
D7016270003
D7717710031
D4791802
D7012340051
D7412660330
D7015170035
D7114510028
D7212970050
D7013540394
D0108890187
D7020350457
D0111150079
D7020649722
D7020070444
D7013160901
D7020553561
D1060191
D0127442976
D0107210038
D4209442
D1043481
D7013831445
D0108420285
D6328393
D7119851506
D7014790314
D6667602
D0125290142
D7111610062
D7118240255
D0115950307
D7119620722
D0127441771
D7135338
D1045880
D7217970722
D7010120436
D7020554393
D7561267
D7020590038
D3463616
D7018850982
D7014320664
D1345840
D6309368
D3823738
D4061505
D7118070130
D5000150636
D1109168
D7111630484
D7015030115
D6244555
D7012701678
D7010210041
D7213330445
D7216044579
D6055231
D7016240119
D3644585
D7020533271
D7020090021
D7119130280
D6466526
D7119791818
D7014350009
D7014320137
D7119830871
D7020580313
D7010280576
D7019081252
D7011060239
D7020160441
D7015010688
D1086884
D7114960054
D7020592227
D7128127
D7114601733
D1363027
D7020720366
D7117840993
D1113711
D1780202
D7018652342
D7116190011
D7119832602
D7217180008
D7020540016
D7020261058
D7114900630
D1763411
D1002493
D1112057
D7011100235
D3458775
D1772678
D5815950
D7119250096
D7114850362
D7119850566
D7119520721
D7017520076
D7020430015
D7014000191
D2271846
D7020556815
D7012340422
D7013540166
D7111550059
D7020690449
D4434866
D7020532267
D7010170588
D7110950728
D7020432163
D2181725
D0115950341
D7115740066
D1500446
D7017442167
D1184335
D0116300078
D7114551272
D7020370063
D7020602867
D5946923
D7117231036
D7020553914
D2849686
D7013831010
D4434876
D0109070050
D7415480218
D5759246
D2222686
D7017760306
D7018800838
D3355827
D7020533365
D7020431712
D2049819
D7567092
D3706284
D7118600412
D0118670523
D1761333
D7017440277
D1313838
D1038632
D7020602980
D0109370574
D1025936

D1094727
D7119983236
D7115780015
D2832932
D7119560856
D7216670184
D7020204772
D7008882
D7211710079
D7117170416
D7020542800
D7213400102
D7013890402
D7214590115
D1002795
D1307480
D7214190747
D1073071
D0108890228
D7020090331
D7212580246
D4434700
D0138230430
D7020533354
D0111370009
D7018860255
D7714330878
D1287042
D7114880047
D1754395
D1172488
D7119850795
D7020555596
D7020720848
D0107620667
D7014750648
D7016233021
D7116460004
D7020721087
D7717710049
D7563344
D7010000388
D7013940741
D0128761441
D7211730574
D7412300030
D7013191307
D7210741776
D0109160022
D1182808
D7013460065
D7211730832
D6236543
D7018611177
D7118210193
D0115540519
D7016260144
D7020554121
D4792534
D1182377
D7114010855
D7119880149
D1363312
D7015500035
D1119664
D7011420079
D1114695
D1014688
D7018800768
D7114090645
D7013050178
D1901275
D7413930345
D6056471
D7009573
D7118350264
D7118030724
D7020594865
D7013830123
D7119520550
D3644562
D7213780072
D6667537
D7015511020
D4795231
D7012260011
D7015510519
D0112250059
D7020527340
D1047044
D1435424
D0113630004
D2061701
D1690070
D7013460024
D1594323
D5411399
D7559529
D7020340101
D7213400338
D7012701751
D0107640033
D7014650234
D7020556807
D6052966
D1181364
D4015950
D7013191651
D7119832958
D6242491
D7413900047
D7118070207
D7119850740
D7013860004
D0117380120
D1287929
D4794887
D1112058
D7214540242
D6244429
D7119691322
D7211730462
D7011960194
D0108610005
D7020720426
D7214190348
D7017471083
D7020553877
D7013060748
D1190271
D6668276
D0139900020
D7020610959
D7715470027
D1111667
D7020070804
D1004440
D0110420416
D7571089
D6238781
D0108810002
D6057876
D2153749
D7211790454
D2251119
D7569548
D7020361426
D1842351
D7020090187
D7211810082
D7210540015
D7117171049
D5302356
D7014650185
D7116940071
D7115740105
D7020601846
D7119870161
D7020532849
D7020390359
D0108420297
D7114470755
D7020530495
D7020532455
D1358326
D7119911129
D0123070001
D7114601704
D2115177
D1358439
D1046056
D7020721728
D7216044384
D7218190016
D7714331020
D1003956

D7020531638
D7211750074
D7214540029
D7110920569
D7020554228
D6246948
D0105850472
D7011830358
D7016760002
D7116410139
D7715530439
D7114040091
D7013831261
D1203977
D3644598
D7013050538
D7211750054
D4787287
D7119880909
D1127725
D7118260712
D7013870352
D7119910866
D7214190314
D3969331
D7561245
D5413061
D7013911272
D7013060066
D7118062515
D0123690003
D1115616
D7118060362
D1435421
D7111630045
D7020553545
D7119560792
D7116568366
D0139960046
D7020531579
D7115790035
D7012701896
D7020556951
D1112567

D7020531572
D1306963
D0115540684
D5290450164
D7119480554
D1114170
D0109080036
D1433901
D7013070168
D1468546
D7562127
D1107129
D7119200006
D7020533358
D1105777
D7010620219
D7020380047
D7119480774
D7214160170
D0107780055
D0129660681
D7014750998
D7015870036
D7216690038
D7015450954
D7011420007
D5025180
D7211680028
D1498468
D9958109
D2125406
D1901285
D7016000265
D7020390136
D6922313
D7119631517
D7118020005
D7020533154
D7119832279
D1621273
D7414620770
D7118600454
D1792957
D0103540403

D7020556271
D0125350077
D6923214
D7013050617
D7138625
D0114360004
D7216571006
D1302879
D6327796
D7012701186
D7212580308
D7114490458
D7011830090
D7013911760
D7020531432
D0129590035
D7117090775
D7016400585
D7020401752
D7020602843
D11640596
D7119892034
D4053121
D6248842
D7119880046
D7119610100
D7117170527
D7013050583
D0107620462
D6240083
D1499628
D7119790006
D7110940476
D1321461
D0110420421
D7010120213
D7118560589
D4022606
D7559771
D7117160093
D7714300127
D7020230032
D2226951
D4435007

D6053393
D6248890
D7119985800
D1446194
D4799225
D7010040023
D7016000028
D1005307
D3358660
D7020602364
D7020160546
D7559661
D1109844
D0109350014
D7210430492
D2089531
D2125399
D7014000196
D7214910149
D4788604
D7113230179
D7015590972
D7014210360
D0107620905
D7211790482
D1279513
D7119988858
D1090351
D4801400
D7212090102
D7013460001
D1113421
D7118330155
D1158290
D7016233512
D7011460420
D6595003
D1159778
D7216440106
D7016381616
D7020556808
D7019081197
D1468867
D1746635

D1348893
D7020260319
D0125340054
D1038939
D7116411138
D5963277
D7012250073
D1763845
D7016000586
D7571022
D7118060157
D1090408
D7017741779
D1328450
D0105860038
D7119730221
D7114090730
D2931551
D2471798
D7119620110
D7013170388
D7119692235
D7014750619
D1345075
D7212570134
D7020527927
D3460195
D7020556931
D7210430175
D1118404
D1115355
D7020160674
D7020721142
D1120071
D7412300084
D7714290071
D970321
D7116581135
D7112901174
D0116460005
D1089793
D7014320213
D0109770684
D7119930013
D987241
D3326063
D1048762
D7714230335
D7020721742
D7010381
D7020593563
D7020596232
D7117010078
D7211190047
D7018670005
D6465572
D1306746
D7135515
D7020555860
D7138681
D7562890
D7115750001
D0104970092
D7020126834
D1347652
D7013191073
D1000590
D7119780039
D7019081214
D7510792813
D0116150009
D7135306
D7114471508
D7119860133
D7015500277
D7129550
D7119830821
D7211730281
D6882507
D7212060088
D7017570100
D1074561
D7211730799
D7020300557
D7018890044
D9078861
D7020092885
D6237888
D7010280597
D7020720212
D0111370072
D7119300021
D1157650
D0107210097
D7020553362
D1446045
D7020160739
D7559757
D7020527465
D7211730623
D0109050024
D7110950147
D6052843
D1097102
D7119692265
D0105670014
D6668059
D7014750584
D7119891526
D4795915
D7014750758
D7020596414
D7119880632
D1309373
D1780514
D6247970
D1087258
D6001154
D6668544
D3579822
D1515171
D6329876
D0116730119
D5152471
D7020531141
D1581158
D1108203
D1358144
D1108264
D7020721470
D6177472
D7212580344
D3173460
D0115540040
D1149847
D6238066
D0115540496
D7013010837
D11761024
D7020527840
D0105470023
D1048145
D7013911789
D6238799
D7561277
D7210430057
D7012780030
D7111610058
D1201984
D7114490378
D7016350021
D7018810583
D7216430011
D6594963
D2198487
D6666900
D0107930667
D7020410154
D7211730856
D7119631492
D7014000174
D7018810768
D7020240363
D7212060143
D0111910126
D7020556901
D6595402
D7020361153
D5662478
D7216441251
D1732979
D6923468
D7119380125
D4792235
D7115670010
D7114830578

D7011110218	D7218000034	D7020556303	D0108490030
D0111690010	D7020556188	D0117940011	D1001397
D7119520580	D1515201	D7020710115	D0128680194
D7020550611	D1301201	D0133110023	D7014791028
D7020260173	D3507232	D1016262	D7563042
D6056772	D7020596519	D7020532786	D7111630125
D7119930285	D7553290	D7211130015	D7017440042
D1148703	D6465825	D4795966	D7020570008
D0115090108	D2125322	D7020430682	D6244315
D0129520046	D7113231405	D6922927	D7214590035
D7213330701	D1182972	D1113552	D1033294
D7013590220	D1737188	D6298162	D1290241
D7212090270	D5104188	D7020555927	D7216660620
D7010170240	D7119851749	D2630856	D1038705
D7568688	D7015960335	D7013890823	D7020531682
D0109230068	D7210480014	D7020431843	D7020410054
D4828221	D6595413	D1121204	D7113706013
D2961682	D7013830239	D7112471149	D1284676
D0109790044	D7118070211	D1051649	D2848333
D7020617739	D7013170339	D7119831524	D0138080058
D6665641	D7020557009	D4794836	D7114090621
D7214540165	D1328924	D6051765	D7020530103
D1112054	D7119800115	D7117171374	D6327426
D7210740619	D7018770948	D1284555	D3677775
D7210430861	D7119985621	D7014660335	D7119280076
D0110420076	D1157533	D7020556142	D0117392506
D7112900437	D7020430707	D7020200146	D7017430429
D7114510196	D7008361	D4014054	D7020532715
D7017550189	D7118062120	D0129620023	D7020291380
D7212930033	D7013060499	D1071790	D7018800721
D0107210049	D1060411	D7114090816	D7117840567
D1344759	D7014320643	D7015410192	D7013190556
D7011960179	D7014751080	D7015880246	D0117380042
D1191021	D7119851722	D7012240177	D4200200
D7010010084	D7020603884	D7212060019	D7216950034
D7119850826	D7111640223	D7020300791	D4347283
D7019081220	D1509903	D0108440160	D7020430741
D0128760738	D7020160407	D0129661053	D7111550327
D1275613	D7211720758	D7117160193	D7016270336
D1673071	D7020300690	D6594998	D1748950
D7018670064	D1033309	D7113660005	D1446122
D0116730071	D4801468	D1304400	D7010120994
D1090520	D1618796	D7110920913	D7216044456
D7012701513	D7211810340	D7119920703	D1156754

D7020721579
D6243835
D0109200084
D7112892241
D7020532983
D6052069
D3109741
D1392629
D7020591848
D7114551873
D1183019
D7119811109
D4434529
D7012790117
D7114420371
D0127460013
D7119831613
D7119792034
D7011830435
D7139139
D7217970024
D0109420307
D1146031
D7119960032
D7014320480
D7020431907
D7020470133
D7010010066
D7113708145
D7216690191
D0129620179
D7014750455
D7012690002
D3367439
D7114830387
D7020553737
D7128116
D7119984510
D5980474
D7020290661
D7119750091
D3358246
D1669617
D7210741768
D7114512536
D1602640
D0107840501
D7118410556
D1344369
D7020542479
D7412610238
D6241937
D1182920
D1157503
D7119760030
D7112906211
D11186690
D7018801371
D7020682471
D7114830611
D7217970918
D1048249
D7010150183
D0125228962
D7119891628
D2132572
D0116550098
D7020594661
D1145686
D7216470001
D7020721020
D7010200016
D0127500014
D1048872
D7114601726
D7214560090
D1287024
D7020070406
D7714330279
D7016000458
D1655085
D7013911426
D1303911
D7013010086
D0107780031
D0104640423
D2084248
D7012340195
D7020604255
D1042062
D7119630140
D7115740059
D7020531052
D7019050218
D1282578
D2192119
D1115375
D0104210067
D1182901
D6053807
D7020556389
D7561196
D0118670686
D7018770790
D2227343
D7020601680
D7119981778
D1331031
D7012120409
D0115540165
D7010150189
D7216690220
D7119430078
D7119860050
D9958098
D0149430052
D5005244
D7018670069
D1284255
D1078435
D1342110
D3460806
D1005148
D2125429
D7110950155
D7020260832
D7020721030
D7115740049
D7020553015
D0109370351
D7128103
D7020331590
D7110950140
D3687683
D7119987581
D1446135
D7015970170
D7119572559
D6327999
D1097331
D1016097
D7138711
D7110950267
D1445686
D7129791
D7020410163
D7119680373
D1000418
D7111630716
D3461243
D7119250339
D5662501
D0127441873
D7214590510
D3996475
D7117840930
D6641945
D1071978
D7217972463
D7117080053
D0217270029
D0108890385
D1815598
D7020331516
D7113706466
D7114880033
D4043755
D1212929
D3463165
D1056966
D7119891799
D7718920098
D7110950165
D0107620083
D7114830172
D7020070474

D5030230216
D7020693650
D7210430244
D4434970
D0115120472
D1901271
D1133683
D1287093
D7119160203
D7119920308
D1149856
D7213330258
D7412660441
D0109870018
D7216420056
D0115520408
D7014610715
D7119832956
D7020261090
D1562204
D7020720903
D0124070076
D0111160050
D7119691598
D4500861
D7020431825
D7020070251
D1038869
D7210430331
D1000881
D0217270009
D7013050155
D0118672952
D1105734
D0105910419
D5662448
D7020430787
D7013910733
D1059088
D7119641102
D4434774
D2125463
D1196928
D7110950146
D7217974181
D1100396
D7020531748
D7020410209
D1059949
D3279961
D0109160021
D7714330012
D7111631056
D1347480
D7119850030
D7569889
D7020601534
D1045556
D7119480005
D7020557014
D7018630619
D7020595867
D3627924
D7010120729
D1901254
D1451136
D6052747
D0119470073
D7013060308
D7211790034
D7119760047
D1652921
D7211731020
D7135163
D0127443323
D9080326
D1347499
D1437669
D7012340197
D7211740005
D7020532430
D0115060005
D7714330243
D7114830259
D1446257
D7720190043
D7020602470
D7015070214
D1114811
D0115090882
D7119920707
D0112040004
D5442625
D0127460171
D7716340028
D7020430642
D0109070106
D0117940082
D3988679
D7112410375
D2690646
D7212090245
D7006166
D9958111
D7015592680
D6923308
D1999259
D7011060540
D1033412
D0115540507
D7715560150
D1033342
D7011940497
D7216571066
D1514184
D2044067
D7014710328
D7020580217
D1370072
D7119910462
D7020556980
D7119920561
D7018810565
D3548985
D7020556034
D0125228655
D7119980468
D0115030485
D1033374
D7213330225
D5506340
D6246645
D7212010054
D7020556693
D7112880108
D7559803
D1862979
D7018890464
D1000208
D6238082
D0107880032
D7118460616
D2215353
D7013830326
D1526878
D0128630028
D7020470316
D7571011
D7020532471
D0104640433
D7010150245
D1157842
D7016382638
D5429030
D7119760116
D1339138
D7019052383
D7020550009
D7020721450
D7570327
D7013460673
D1392672
D7020390163
D4351632
D0110420089
D6299039
D7119760130
D6922933
D7020620301
D7570596
D7020366450
D7013060456
D0107770090
D2487391
D1983905
D7019050006

D1298443
D7020554361
D7118460339
D7010260186
D7112908595
D7110920928
D7119880079
D7560302
D5230690013
D7020720005
D7414620734
D7211790546
D0115950149
D7020470381
D7013010911
D7217970135
D11180516
D0113430022
D7119812601
D7119720060
D7020520835
D7217973577
D6052893
D4466016
D7119982626
D3627947
D3680927
D7110950163
D1014719
D7020470107
D7112430273
D7014210205
D1178887
D7017534402
D1441703
D0127460009
D6055235
D7020401500
D1445969
D7217974298
D1292879
D7565492
D7014250449
D7118520005
D7214590036
D2128624
D7114510220
D1300821
D1113482
D7011060295
D1783631
D1293506
D7114601908
D7211750052
D6923164
D1325758
D7119831385
D7020720641
D0111000880
D7015101892
D7014760352
D4797145
D6246223
D1001275
D1290257
D1799671
D4215072
D7016790163
D1056460
D7133054
D7020553714
D1559678
D3460092
D7020430750
D7119250018
D1000020
D6236042
D7213750131
D7020663809
D7016261116
D7013460710
D11640537
D7130435
D7020591099
D7014211285
D1038843
D7118260616
D0103440049
D1110390
D0105860001
D1043528
D1105377
D2205766
D0128680141
D7020521127
D7119620746
D3485515
D1757048
D1114140
D7014211646
D0129663828
D1576163
D7118070194
D0105850482
D7016381204
D7566289
D0115030737
D1277166
D7212930026
D1044381
D7214540085
D1473284
D7020556896
D7116460017
D1047624
D7018720166
D7718920091
D1817443
D7212090297
D7020555931
D6246204
D7020580563
D7020540302
D7015102306
D7015870214
D7020591185
D5946935
D7010120101
D7570029
D7017580355
D0116470227
D1002561
D7110950103
D7510420108
D7013830813
D1292460
D7012120309
D7020141300
D6052598
D7017570155
D7118351938
D1793902
D970065
D0104800824
D7133177
D0115090426
D7115710048
D0129720076
D7210480017
D7216571485
D1436618
D0113430047
D0109750316
D7119791962
D0108480089
D0125240485
D7212570200
D0138090013
D7014791193
D0104820191
D7114890214
D7119851726
D1113775
D7111630117
D7011520013
D1048832
D1469920
D7128313
D6328469
D3339908
D0107120065
D0104980002
D7012740485
D7020721476
D7119843174
D7114880058

D7013070009
D1726988
D7010000189
D7118340143
D7020542578
D0105860014
D1148097
D7119790016
D7112905897
D0116250001
D9080301
D6244334
D7013910030
D7214160173
D7013910972
D7014710493
D1499803
D1309138
D7558355
D7010150263
D0107050032
D1196280
D981962
D6327609
D7016000595
D7020602733
D7216690463
D1446160
D0103550010
D7020601854
D7119920713
D7211760201
D0123400638
D7216090301
D7213780045
D0108470233
D4247826
D2425690
D7015170111
D7119680235
D7014320710
D7020601252
D0109850018
D1090383
D1499341
D0129600106
D1445470
D0138270184
D4787926
D6052908
D7119630161
D7715530397
D7020596582
D7119611167
D7119910100
D7015121455
D7565344
D1655179
D7012210143
D1740260
D7213400420
D3513080
D7012160034
D7110921075
D6236049
D0110490098
D7118060099
D9078801
D0107930428
D7216530032
D7113230503
D7014750440
D1303364
D1177535
D0109360004
D0119560022
D7714330130
D9080296
D7012320112
D7561885
D7215630075
D7118070202
D7211790468
D1302342
D7115390025
D1760731
D6668253
D7562451
D7020533943
D7211760058
D7015580089
D7012701883
D1787906
D1016769
D7217970299
D7211720311
D7020204735
D1119393
D1783964
D1344638
D7013060069
D7112420236
D7118560933
D3680493
D7118460485
D1603634
D7118340031
D7020430632
D5662476
D7012701754
D7218101487
D7119960123
D6242495
D7011060302
D7018731147
D7015970117
D0117940043
D3355676
D7213330250
D7213750048
D7119880918
D7114810029
D1303954
D7119560624
D7211730839
D0110510004
D4802723
D6243702
D2245193
D7013910651
D7020432028
D7018990610
D7211800096
D7117830146
D1089761
D4347308
D0107210289
D2033089
D0107320377
D994185
D7118030744
D7020520667
D0127443404
D7020531115
D7020556304
D0103980059
D1014494
D1320620
D1178589
D7017720021
D7119680109
D6241121
D7114830255
D7020432101
D1715962
D7006169
D7119341357
D7119850345
D7217974313
D0109450008
D0128680220
D1735822
D7119981679
D7015990038
D0109250098
D7020593820
D1481486
D1098360
D1196238
D1519300
D7211730815
D1446177
D7118510241
D7020070759
D7020601869
D7015550369

D7128987
D7119988031
D6056205
D7119920522
D6328459
D0115030065
D1001469
D4246106
D1446275
D7020532818
D7020556917
D0127440718
D6238073
D7510420117
D4434545
D7119571919
D1920000
D6641957
D0115520515
D6249100
D7119750057
D7020350509
D1179264
D2215351
D7216571670
D11182100
D7211390194
D7016260339
D7014790268
D7012620362
D7020554668
D7020350745
D7213330285
D7020542774
D2030883
D1515086
D7020490212
D7016362202
D1056568
D1392686
D1326686
D7119680356
D5662466
D1108323
D0109750237
D0109370524
D3121137
D6242858
D0127500008
D7020350044
D7139387
D1115001
D7119641621
D0104550344
D7020720352
D1205786
D7013830828
D7020070103
D2203170
D7116410361
D3887236
D7016280020
D7119850756
D7020410412
D7020554644
D1304925
D3711031
D1901602
D0107320206
D1112062
D7215300002
D7217973853
D7020533138
D1101390
D7020555657
D7010121031
D7013870123
D1642434
D1901596
D6923140
D7020533586
D1179729
D6052706
D1183247
D7215650009
D1623321
D7119692078
D7018720100
D7212090894
D7113670324
D1001704
D7559668
D6667624
D1760319
D6053775
D7114550432
D7119880622
D0125200003
D7118351068
D7210750018
D7571166
D3463695
D7013820033
D6666701
D7117830067
D7011940466
D1186277
D7212930029
D7119880158
D6242078
D1482323
D7211810124
D0124040155
D6328831
D7118351694
D7010413
D1114487
D3644298
D0127442284
D7020260576
D7119692483
D1101956
D3462239
D7010610046
D1113556
D1362863
D7116460080
D7211720523
D1107972
D1368750
D7117160101
D7119831928
D0107070155
D7211760162
D0129720574
D9079173
D7020533646
D0125228632
D7020710062
D7110940316
D7117170170
D4800942
D7714300136
D4434526
D7110950694
D7116480053
D0109450050
D7020721363
D7112420128
D7114090596
D7110952717
D11186090
D7020252442
D1001311
D1031267
D7212970004
D3488424
D1003393
D1191455
D7110950132
D7020030010
D1042427
D7011960160
D0109150023
D1301469
D1755129
D7214190107
D7114470318
D1661317
D7213400599
D7116150135
D7010431
D1120227
D7114010450
D5060610058
D7210710009

D1038757
D1113434
D7110840344
D1121657
D7118330049
D1756090
D4970668
D1056507
D1309450
D1369622
D1149622
D1332767
D7110952718
D1257574
D0109080030
D1103253
D6665933
D7214540234
D4030884
D7114830471
D1367111
D6666748
D7020390364
D7717490010
D0109790065
D0103370287
D7011100075
D7018611242
D7113270072
D1434314
D7020533600
D7013170374
D7212580060
D7112420115
D7217930095
D7714720074
D1157582
D0119470075
D7012260175
D7020556177
D7010506
D6052799
D4795260
D7010210016
D7011040004
D3892196
D7114090130
D7020554701
D1287020
D7017580133
D7012260200
D7018970004
D7119691879
D0115030004
D7013070194
D7017530064
D2630889
D3687819
D1125129
D7016233998
D0112325636
D0104920003
D7020530482
D1445718
D4802531
D7113213096
D7012701733
D7116980191
D1309443
D7020533384
D0110420336
D2049177
D1264360
D7118410022
D7113706044
D6668410
D7020556440
D7119691959
D7016000792
D7119680302
D7210820038
D7715560107
D6667170
D7020553402
D7019002410
D1044950
D6248295
D7018930028
D7012130163
D1874188
D1011644
D3477126
D7135110
D7020530465
D0117400019
D7413120127
D0125240706
D7119480761
D7119380010
D0128390040
D7020554405
D7012260014
D0125230030
D1089955
D4195678
D7013050121
D0104270072
D7012120204
D7020557137
D1264630
D6246703
D7013831134
D7119470663
D6666546
D7020402333
D1498528
D7016350066
D1113894
D7020694245
D7412610221
D1301189
D0109200020
D6466372
D7012740258
D1097291
D7020557112
D4800000
D7119610059
D7214910055
D2134295
D0107760144
D7119471077
D1446036
D6238155
D1045967
D0125240650
D7020430680
D7118510012
D0105850153
D1714620
D0108890276
D0123750181
D4061515
D1233016
D1099043
D7119520015
D1014496
D1011777
D0110420382
D7014260282
D7119850251
D7011470001
D7020557093
D7020600059
D0125240243
D7020556800
D1085636
D1145937
D1105463
D7119730369
D1494019
D1000668
D7018630033
D0129620145
D6666186
D1466373
D1012426
D7113610008
D7010120134
D1043478
D7213330489
D7011510771
D5625455
D1493938
D7135513
D7020673294

D3514744
D7015011029
D1341334
D7018630567
D7020431859
D0110520014
D7020592594
D7020554820
D7119630901
D7020260700
D7216690307
D1186379
D0109380019
D7216660950
D3519138
D5254114
D7012780031
D7020141028
D7013941153
D7020490233
D7015450940
D7015520012
D7020531676
D7119850369
D7211750017
D1287054
D7561581
D7217974998
D5414863
D0113630043
D7018890470
D4802692
D7018912701
D7014750657
D7020300112
D1446020
D0115950739
D0111000891
D2192912
D7020532775
D4211864
D6236615
D7119891281
D1446143
D2125337
D7014210292
D2484583
D4790374
D1709566
D7119561354
D0103550171
D7559455
D7211760065
D7119890392
D7020160489
D7018655636
D7118060639
D7020160086
D7020260350
D7563121
D1196805
D7020532912
D7016870143
D7212580287
D1436623
D7216910004
D7112896885
D0113430150
D7114420159
D7114430181
D7129548
D7218310431
D7011090019
D0115540239
D7215630112
D4396070
D7113221609
D7113701133
D7015011033
D7018870002
D7119760136
D7117400014
D7139137
D7510350028
D7559525
D3675873
D7119987390
D7211720422
D1358177
D7212090708
D7020410332
D1056360
D7113230762
D1359680
D0127460025
D0105820046
D7213330165
D7015571958
D1199061
D1302965
D7119630534
D7014750567
D4787229
D7020556970
D1086026
D3137279
D6057184
D7119360403
D7118350396
D7020682281
D7016361095
D7119461546
D7020420384
D7562079
D7117850637
D7020520453
D7216660597
D4790147
D7113260288
D3462807
D1060301
D7020030005
D1150553
D1358118
D7119851661
D7020430853
D1484709
D1481192
D1112105
D7016000724
D7020533214
D7116980017
D7020431757
D6050987
D7012702439
D1167913
D1446084
D0111370110
D7020553461
D7510780066
D0118650014
D7117830140
D7020721203
D7015870224
D7119880841
D1149684
D7119791964
D7014751205
D7019050067
D5152568
D7014261102
D7112490039
D0115030251
D7420672
D7020720683
D0139900041
D7114980017
D7720500740
D1527384
D7013460017
D1109436
D7011080081
D3500833
D7216050832
D0104940041
D7114420553
D7020682207
D1736244
D7211790094
D1797013
D1084728
D7020140993
D3694094
D7015550371
D7217961276
D7119691381

D7216530132	D1112265	D1106598	D7119981306
D0138210081	D7013170331	D1290289	D7020520071
D7110952614	D0128760133	D7018611215	D7119988821
D1025940	D7139235	D7013990087	D0128761464
D7020532153	D1499012	D7020721798	D7559667
D1287408	D7010640289	D1830484	D6243884
D7017570111	D7018880171	D11186102	D0110490073
D7020530545	D7119870053	D6641981	D7018630898
D7020060003	D1113849	D1110293	D7020141695
D7119845362	D6050978	D0118671417	D7214590450
D7218310605	D0103970045	D7006904	D7119620036
D1056581	D1107506	D7020598028	D7114830680
D0139940090	D1646212	D7012180030	D7714230363
D1218654	D7118060076	D7014750290	D7110950829
D0103420239	D1032296	D7210740397	D7016270274
D1003140	D1772806	D4319899	D0115540416
D7020593536	D7020604306	D7014750015	D0129663963
D7212090667	D0115540665	D7020721545	D7114011407
D6466361	D4389223	D7020594009	D6248730
D7119561147	D7011420186	D7133045	D7020592274
D7013990325	D0115950198	D7559459	D3185418
D2848334	D0129600112	D1155732	D7113260422
D0105470011	D1042190	D7114510215	D6246250
D1392704	D7012700772	D1047753	D6056194
D7212090468	D2125460	D3680686	D7119831944
D7119930072	D7214190349	D1891753	D1392659
D7210800082	D0149860019	D7020361145	D7010120264
D7119642310	D0108380014	D0108560457	D0107550044
D1115199	D0109160389	D6329852	D7119981369
D1313819	D7016780061	D1358474	D7114450001
D7216661111	D0125228649	D9079122	D5051530190
D1133495	D2030894	D6466037	D1446048
D7118330033	D0129720294	D2523251	D7211790299
D7119560623	D6238989	D7011460613	D6248225
D7020293025	D7119984980	D7013940078	D0139930093
D7119891671	D7561504	D1148767	D7133203
D7138713	D7016232230	D1596866	D7011450008
D971596	D1863053	D7015870046	D7020340020
D1111918	D1732805	D7118061608	D1055925
D7118330004	D7012740378	D7013460066	D7212090909
D7119692117	D6923177	D7212570190	D7020361149
D1290457	D7713420021	D0103350039	D7113260424
D7114090872	D5466692	D0127442784	D1178883
D1903602	D1347401	D1006066	D1403084

D7561891
D0115080026
D7020693936
D7013990271
D7020410120
D7217970190
D7133050
D7218380035
D0111000907
D7012620871
D1015117
D7119880343
D7213330209
D6923042
D7114960302
D7217961256
D6052570
D7020460461
D7017560505
D4162669
D1361401
D7216660861
D7020470055
D7212090391
D1047743
D7119880366
D1014700
D0103560033
D0124040229
D7015180206
D1709289
D7020460061
D1156756
D6667823
D7117160032
D7110920713
D7216050731
D7112460006
D7012701099
D7020531461
D6465796
D7110940357
D7014750502
D0115090008
D7014260769
D0112170025
D7119691318
D7020070626
D7020556810
D7714331000
D7215770043
D7018771398
D7020410288
D7212950003
D7119800102
D7015150111
D1071511
D1605476
D10354460
D1188445
D2842272
D1138318
D1182999
D7020530681
D6052853
D0109290031
D7118510317
D7210770014
D7020556341
D7020618334
D7010160127
D7212090784
D7217982289
D7019081243
D7117090810
D0109070020
D7020530890
D7217970984
D7016350090
D1055971
D1133442
D1217687
D7117170504
D7114040458
D7119520530
D7013060731
D0125211141
D1322882
D0129610015
D7212090179
D7010260183
D7020553370
D7125985
D0109390051
D1392627
D7013050405
D7010210052
D1570163
D7217970873
D7010590324
D3463051
D7020410028
D7016270027
D1523278
D1114536
D0125350065
D7011500124
D7012120133
D1445646
D2976842
D1110641
D7020685740
D7119620114
D0104010040
D7015160137
D0112842172
D7018611178
D1109176
D1265432
D1001471
D4801375
D0112200114
D1106494
D1182424
D0108560048
D7113231470
D7020591057
D7212090536
D7218101441
D1033832
D7216530037
D1099923
D7010120960
D7016380360
D6243700
D3851346
D7119832153
D7020520757
D7119641155
D0109350101
D7119831605
D7215630004
D7211760430
D6641905
D0125160262
D7019050066
D0109770189
D6328497
D0115130009
D0127500080
D7014211375
D7112894235
D0123750837
D3671757
D1444405
D1313975
D1002308
D0117390759
D7116411570
D0103350129
D0104560194
D6248779
D7510792191
D7217961282
D7119980645
D0124080031
D7015870179
D7013161805
D2847625
D0115940004
D1362067
D7020431268
D7216950009
D1170727
D7017690624
D7012700990

D7138719
D2166582
D11640469
D6641980
D1144846
D1572651
D7020090246
D7016000541
D1366349
D7119620350
D7020554301
D0104910369
D1763880
D1264492
D7118460375
D4802610
D3644612
D7216660331
D7018890467
D0117380127
D1098148
D7119830679
D7013891464
D7119640316
D0109390067
D7211130017
D5914048
D1772130
D7016210988
D7014671045
D1329463
D7020532371
D3459940
D1305571
D1056484
D7016350121
D1524689
D0103540297
D7212090670
D0129620113
D7213400594
D1312841
D7714330041
D7117840564
D7510792124
D7211750021
D1060103
D7015020076
D7020363933
D7020555652
D7014750030
D7216661152
D6595030
D7014390006
D7020160787
D7012260039
D7014400464
D4216887
D1789111
D7020684401
D1117929
D0129620112
D7020432010
D7111640092
D1762951
D4802158
D7018766337
D7119920697
D7020556115
D7212090584
D1310244
D7713040674
D7112420081
D7011940439
D7020350113
D7011960142
D3823744
D7214910167
D7020160236
D7010610054
D7214170059
D7117160181
D6057549
D0105651001
D7111640186
D1033867
D4787223
D7020470638
D7020460379
D1089714
D7020721205
D988020
D1104252
D7211790434
D7018850984
D7020090206
D7020595876
D1158045
D7011460531
D7122657
D1329908
D0103540334
D5043973
D7119891649
D2201888
D7115710012
D0111134041
D7020720204
D7218190003
D7020533547
D9959425
D7114850246
D1344338
D6057211
D7113230479
D7114551766
D7018800871
D0105620307
D7117330087
D7016233988
D7018670042
D1677638
D7114420476
D0127460189
D7117170285
D7018630470
D7017581397
D7114830812
D0139960049
D4248377
D7020470559
D7012120350
D0115030591
D7118030719
D7013830159
D7012740129
D7119812691
D1728527
D7011060210
D7020070674
D7020300182
D6239079
D7119982003
D7014211246
D7011520017
D7217930072
D7113260051
D7010670074
D7111630152
D1114776
D1003369
D0120630033
D7016390879
D7119680042
D6668046
D7214580040
D7020300002
D7133194
D7020521037
D7020531779
D7112410427
D7020431903
D0115520350
D7020390416
D7020532132
D7212090331
D1086719
D3460532
D1005338
D7119880249
D7216044423
D7213330011
D7212580360
D7119850357
D1037214
D0128660098

D6327956
D7020531160
D7539932
D7212580368
D7020141046
D1536147
D7119891918
D1011817
D0107610009
D7020260283
D7111630503
D7112420356
D7018710860
D1006061
D7020556722
D7020533382
D7210730016
D0118650040
D7112905828
D7114830164
D7217982244
D0128680172
D7116580429
D7216515011
D0128680094
D1708928
D0128660168
D7717710025
D7020556293
D7112905050
D7020432063
D1049560
D0103550169
D0109160313
D7118350222
D1000218
D7020532174
D1592307
D0109400043
D7020601041
D1111768
D10639124
D0109850005
D1461349
D7210430595
D0125226316
D1711850
D6624346
D7014320184
D0115030285
D6936263
D7010121055
D0127441810
D0115030890
D0107110005
D7013990247
D4793839
D7011060547
D7118240114
D4403464
D2270454
D7020532919
D7212580152
D7114550347
D1078712
D7012270470
D11182101
D1202862
D7020480312
D7015580003
D7119681611
D1113364
D1151363
D0104820267
D7013530088
D7561925
D7712640085
D0108890640
D0118670464
D7010280050
D7114420343
D1031220
D7216450141
D1001288
D7213390055
D7119960101
D7020532891
D7011460954
D7020542808
D3080958
D5012950
D7020556545
D0111070336
D1437729
D7011060257
D1748905
D6052633
D2212581
D7015450917
D8630900
D7016780288
D11640627
D7211230182
D1651168
D7014320109
D7563023
D7013160180
D7211230154
D1113305
D7015920159
D7211760332
D7020331257
D0111050010
D7012120355
D7116110204
D7211810067
D1364598
D5042480
D7116080072
D7018930018
D7020692613
D0103230773
D1445729
D1367653
D7018990033
D7011460234
D7013170412
D1745039
D1136109
D7216440942
D1468523
D7210430421
D7020431660
D7119910983
D7113214859
D7211810337
D1446234
D7020556968
D7119520727
D7116140021
D7020260762
D7020432148
D1300752
D7020721663
D7119850123
D1832245
D1392748
D7214540168
D7119880607
D7119691853
D1370508
D1333342
D7070707137
D7018694188
D1039084
D7213750197
D7017550213
D7116980048
D7018811844
D7011520015
D7013831296
D7020557061
D7020532041
D7020541969
D1477057
D0104170039
D7119760223
D7020160013
D1305167
D7118340512
D0112240054
D1304087
D4045960
D1043217
D7013060533
D4577025

D7119831720	D7567843	D1033408	D7020594019
D7013590595	D1499841	D7119880456	D0105920049
D7510420039	D7114830136	D7006900	D1342085
D7020721394	D7012270297	D7213390021	D7020490038
D0108810013	D7011880098	D7113220806	D1001669
D7110940472	D7118280065	D0149430033	D7112471095
D7119561777	D7128967	D7110920923	D0119470014
D7112880006	D7020556050	D7119692377	D1783138
D7114090956	D7018801347	D7014750528	D0112820049
D1033464	D7214191000	D1115116	D7014750460
D7016232667	D0139960057	D1110246	D7020602719
D1099727	D7015020121	D7020390506	D1182370
D7020252427	D7119930223	D7011460524	D7114830142
D2027799	D7016261109	D1156998	D7018710865
D7014250206	D5071687	D7014740321	D7211290003
D7020596143	D0108420493	D6052587	D7114010376
D7013820051	D1043638	D1393838	D0108610020
D7020602378	D7216690049	D1033296	D7214190274
D7210480033	D7017534391	D7110952626	D2264007
D7119520101	D3342557	D7020420381	D1344619
D4802698	D0113430003	D7112460005	D7010130001
D1156727	D7020556898	D9958136	D7114020019
D1006999	D0104640108	D0138120053	D7114850385
D1183129	D7119692410	D7020600994	D1196319
D1869488	D7013831051	D1106545	D7011940672
D6240735	D0139020049	D1568469	D7020070398
D7210750026	D7020260184	D0112640025	D7510792660
D7113230585	D7010620006	D1468763	D7119791873
D7213330382	D5973789	D3041346	D7212090087
D6057854	D0129660974	D1870727	D0128660175
D1036165	D7210430618	D0127442339	D7013161775
D0108420006	D0104800830	D7110940336	D1749838
D1182350	D6465892	D7013190937	D7216640242
D7020510116	D7020380053	D1371058	D7020070351
D1445993	D7020554433	D7010280518	D0110420398
D7417849	D7019050087	D0105820623	D1105302
D11186665	D7012270313	D7010410	D7213770033
D0108480049	D4195314	D7214590447	D7016260113
D7020530781	D0107760157	D7010170198	D7560012
D7116411489	D7017440262	D7216440488	D1006517
D7010240489	D7011040025	D7212090378	D7110950090
D0109770362	D7714330903	D7013891839	D1879783
D7211730705	D7013940769	D7118240076	D7020592437
D1102893	D7014211226	D4798185	D7211140039

D7119670002
D7018990182
D7011000010
D7413960441
D4797381
D7119980460
D7020593374
D7570660
D7012270294
D1780187
D7714330182
D1509653
D7017656042
D7020160456
D7020020685
D0104550347
D1647373
D7011010095
D1295056
D7214191086
D1113717
D1109068
D7018959628
D1016265
D7013540925
D0113430020
D2254213
D9080661
D7714230383
D7119630187
D7116940029
D7211730619
D7018800860
D7018860377
D1232759
D7714331003
D7510400006
D7216690366
D7018770752
D7715420033
D1737019
D7020553195
D1048836
D7020596062
D7116110099
D6465806
D7018800782
D6466641
D7020410389
D7212010641
D3658433
D7119470903
D7020141042
D2930156
D1256822
D0138290018
D7118470018
D7117090050
D7020601470
D7118280391
D7210540008
D7020721098
D7118350251
D1305385
D2202425
D1001716
D7114601524
D7014390002
D1313984
D7118061293
D7020556549
D7213400600
D3999966
D1000913
D1183097
D7211730866
D9080315
D1348401
D6668440
D7216050104
D7216051118
D7015160033
D7117170013
D7010040026
D1150833
D7114010777
D7017470565
D7020610286
D1068802
D7214590072
D1136797
D7018670039
D7713930012
D7020160334
D1500183
D7211720644
D7114010472
D7020160529
D7715430039
D7216044241
D7138599
D2848680
D7020691486
D7211720835
D7114520020
D7020430975
D7020554729
D7113707717
D1105128
D7020160666
D7020556026
D4794976
D7014790480
D7116980001
D7020554710
D1059783
D1573900
D0116470066
D6328564
D7020470214
D7020672634
D2368006
D7016270054
D0103580015
D7119811982
D1113768
D7117020072
D7118292019
D3884500
D0108320008
D0124120003
D6329830
D4347281
D1000679
D7018630466
D0115030243
D7018771054
D7012780014
D7119670056
D3742121
D7114960119
D1216942
D1824549
D7012701524
D7216660371
D1111209
D7119390014
D7014670527
D1594274
D7012320551
D2630930
D7010090117
D7020556242
D7139211
D1322850
D6246966
D1112316
D1196878
D7119851720
D1002325
D1090322
D7020361171
D7014250490
D7119520737
D1099034
D7117200228
D7130444
D7119642129
D2973467
D6466238
D7018771229
D7211230089
D7020331200
D0108560562
D7012270452
D6466430

D7020370083
D7119880327
D1312628
D11186710
D7020651722
D2125369
D7213330057
D0111460009
D1800916
D7020552471
D7139185
D7020556729
D7117230917
D7018655037
D7013070022
D1148519
D7111631066
D7010980007
D0114360007
D1433677
D7118062283
D7020721179
D7020527434
D7020554007
D6213521
D9080233
D1190266
D7018810807
D7110920694
D7217970700
D7715460057
D7116110253
D1687303
D7020555966
D7119930110
D1287910
D7563278
D1001536
D7020533323
D7020557169
D7216930008
D7020553961
D7118210177
D6237258
D7212580237
D7216690289
D0138270043
D7020460241
D7559788
D1660935
D7713369
D6329866
D7119750078
D7510792696
D7118560455
D1757880
D7020720465
D7712650123
D7113690006
D7569948
D0104240005
D7013050007
D7013460312
D7119560059
D2932616
D7020553246
D0103080016
D7020554896
D7119631418
D7119850018
D7216540206
D7012340314
D7118330055
D7213400591
D7570803
D6237513
D6328094
D6238033
D7020531952
D4801827
D7119870073
D5270360110
D1562009
D7118350349
D7017470135
D0129660909
D7118240065
D1083894
D0129660927
D7119230008
D3651715
D7018811880
D7114830236
D7112430307
D7020431354
D1672668
D7559147
D9080287
D0103420145
D7510791741
D1440535
D10052652
D7012260153
D1757298
D7020527404
D0107340032
D7020601068
D7212930004
D7211310003
D4248482
D7114601359
D7020410006
D1433616
D7118410142
D0124140037
D7212090104
D1468517
D7217971745
D7018990062
D0115090046
D7119930218
D7011060205
D7012790005
D0124120060
D7017520052
D0105920050
D6327612
D7013050321
D7012620572
D9080279
D1148763
D7009633
D7119760042
D6240742
D7411540071
D7216420063
D7010480
D1187962
D0127443117
D7018801348
D1112121
D3517098
D0103420024
D0110800054
D2190390
D7714230021
D7013161778
D7010240191
D7114830226
D7114091002
D7018670071
D7015571386
D7112904918
D7212990014
D7110950310
D1307638
D7119880377
D7413180348
D6426501
D5110070368
D0127460534
D7020601714
D7111610050
D1148035
D0104500040
D7115210004
D1202557
D4798027
D7212090195
D7020555063
D2033111
D7214540091
D7110950042
D7010170236
D7413180292
D1366347

D7020603912	D7410330186	D1047787	D7013170416
D7116680014	D7114471948	D1779696	D7018810822
D7210430433	D7119980504	D7010020510	D2333847
D7210730035	D0138910029	D0107790024	D7714330228
D7119151184	D7119832948	D6327863	D0138210059
D7119250023	D0115950594	D7117840490	D7020580570
D4312445	D1112131	D7110950021	D7714331028
D7119800032	D3696454	D7116940033	D0105870043
D1720934	D7214590436	D7217300166	D6057902
D7018801217	D7119830424	D6856370	D1121282
D7115740155	D3486619	D1121495	D7119791998
D1558705	D7116557961	D7116130013	D7014750021
D5110070056	D7010610062	D0115950290	D7114551536
D0129663759	D1371748	D7011520014	D1057339
D6051795	D7114601858	D7119983121	D1095009
D6466639	D1302122	D7118350308	D4787259
D7119642540	D7211730558	D1436658	D7020470313
D1055899	D7714330281	D0139890023	D7114601072
D1113536	D6871433	D7119480616	D0118720069
D7113230219	D7018811890	D7014710542	D7118350245
D7020201564	D0105900002	D7013460265	D7119870120
D1090425	D7559774	D7013191265	D1792678
D0109390070	D7020555633	D0127442108	D1446207
D1044472	D1089638	D3341069	D0123400142
D7110940270	D7113230540	D1985806	D0111770013
D7218310534	D0129720506	D6849455	D4791195
D0127443382	D7212010197	D7020430648	D7012620888
D7020550594	D1033389	D7119341294	D1060376
D7214910157	D7020432111	D7020550082	D7115740043
D0115080086	D4435155	D7020390157	D7714780016
D7119561053	D1513656	D1445996	D7211970025
D7212570149	D7013060626	D0129720685	D7118350035
D2958058	D7012620594	D7119250090	D7410900004
D1014795	D7138653	D1196268	D7118061092
D0113630005	D1050803	D3340223	D7013830113
D7116940098	D4347279	D7017770114	D7020401724
D3692928	D6667253	D7015592295	D1162809
D1436868	D7216571105	D7012120142	D1243167
D1675146	D1327752	D1392493	D7571100
D7010250021	D0111910131	D1038927	D3449082
D0129620353	D7020527893	D4495686	D7111620118
D1298076	D7119681080	D1305618	D7111590005
D7020603050	D7010640355	D0127460056	D1036393
D5724268	D0109790040	D7010000324	D7114600375

D7117160183
D7017560775
D7020721285
D1499705
D6052863
D7114830550
D7013060697
D0117390752
D7111630451
D7116990031
D1341264
D1114268
D7014380112
D7013050262
D7413920033
D7020554260
D7010090081
D1581193
D7020620328
D2234787
D3463318
D7117840529
D1140196
D5980513
D0125370019
D7411540117
D7016350296
D6052809
D1708161
D7020510068
D6667810
D7019081239
D7020390429
D1705022
D7020530842
D7020260441
D0129720127
D7013910916
D7014750093
D1446256
D7510370012
D7020260135
D7020600010
D7020591293
D7020520496
D1557114
D7020390448
D7013170283
D7115690216
D4436931
D7214910216
D7118330013
D7020693919
D7212090415
D7014320002
D1038739
D1303976
D7020260560
D0110490015
D1218304
D7119330006
D3128833
D7217961281
D7020370067
D7117350136
D4052991
D6239324
D7020556379
D7016000492
D0103620049
D1736801
D7112900914
D6298579
D7214590050
D0139030014
D2082707
D0111780284
D7119800056
D7020533921
D7118600472
D7119850653
D1145362
D6244552
D7012620879
D7020201603
D7020402327
D0109370636
D7216450015
D1437163
D4435080
D7020532782
D1196942
D7012790039
D7013060484
D1293873
D1001133
D1305419
D7114490436
D7012701000
D1088343
D7211730955
D1327229
D6985181
D1056575
D7020532033
D1529657
D7119832616
D7119831406
D0109420069
D7214590098
D3485519
D7119740003
D7020527378
D1161011
D7017660051
D5047586
D970456
D7013060670
D7114601777
D1197835
D6057544
D7211730694
D7020431702
D7020260014
D0125800071
D7020490075
D1446140
D5110070244
D6299201
D7020470366
D3644680
D7020556433
D6239820
D2084619
D7015920153
D7014750187
D1218035
D7111630481
D0115030041
D7020531725
D4799178
D0123400528
D7011000020
D3644573
D1133308
D2215644
D7211270025
D7119300098
D3462681
D0108440157
D7212010572
D7214910210
D5662479
D9080305
D0107210017
D7217961244
D7116410437
D7015520011
D7010467
D7210742115
D1073148
D7214540104
D4802812
D6667231
D7119981494
D7015570974
D3689807
D7119983654
D3354093
D7020721588
D7011500028
D1347329
D7018801406
D7114090626
D6053420
D1392742

D7015011039
D6923371
D1120082
D9080294
D6238146
D7013070062
D1044939
D7012700793
D1005063
D0108420260
D1182563
D1396851
D0108420495
D7559515
D7020410197
D1096212
D1149523
D0112325707
D7214590376
D0118671803
D2142912
D7119390032
D2848748
D1654872
D4061536
D1505986
D5980466
D6081641
D7211680035
D7119987450
D0127442281
D7020654131
D7014350008
D0110490075
D1790462
D11186660
D7114470031
D0117890056
D7018890415
D7115740047
D7119870047
D7013830784
D1112579
D7119988982
D7018771287
D7020533659
D1217720
D1435445
D7018930044
D1065499
D0108890231
D7017452044
D1186250
D7020671842
D7018700297
D7216570930
D1150019
D7020402292
D1199277
D7020530512
D7011830393
D0111170146
D4247982
D7020350026
D1151593
D1500670
D2125338
D7018959865
D1772780
D0109870050
D1198727
D7114471431
D0105820664
D7020596165
D1467502
D7110952462
D3706073
D7214931620
D0149760027
D7119290194
D1000912
D7119880056
D1748024
D7015910005
D0113430116
D1434662
D7214942759
D1038007
D1182421
D6300148
D7210740526
D7020070494
D7119630834
D7112897713
D7020531025
D3700098
D0118670077
D7012621069
D7126002
D7020331252
D7020610297
D7119390021
D7217971872
D1655165
D1001007
D1758898
D7213330360
D7020260654
D11186523
D1113401
D1149626
D1034313
D7119880054
D7211750011
D7114090882
D7211760075
D7211790003
D7114600992
D7011510778
D0104990013
D7114040244
D7017470337
D1181571
D7114600805
D1115071
D7119831713
D7213770007
D7119982279
D7114830008
D7111610002
D4038490
D1977565
D3358535
D7211810401
D7020410387
D7017760707
D7570343
D1102838
D6247141
D7720190057
D7020530661
D6238098
D1107135
D1274637
D7115740179
D0125340105
D0139030085
D6243390
D7217970247
D4479292
D0117380125
D1033648
D7020617637
D0112820057
D0104820378
D1761784
D7214590144
D1136787
D7020604270
D7017560395
D7569522
D1446133
D1005758
D7211720543
D7116411157
D7019081225
D0109770106
D7115740034
D7018800709
D0128660155
D7570357
D7018801356
D7542325
D4791898
D7559841
D6057976

D7016350147
D7012690055
D1772156
D7020580632
D7020530757
D7211730776
D7020141432
D7218010703
D7720190223
D7217970664
D7213400669
D7119880727
D1604336
D7714330293
D7016232152
D1069117
D7119611107
D7020252426
D1315039
D1102669
D7119810337
D7119850136
D4794411
D7126249
D2128576
D1112236
D7119831458
D7020430652
D7119880880
D7020556686
D7015410205
D4252263
D1688390
D7216513332
D7015101785
D7010080054
D7012790010
D1768519
D7217221014
D7119681561
D7413180141
D7129748
D1218539
D7111580214
D1786487
D7020531627
D4434507
D7010150200
D7215700135
D7714331185
D7020432130
D7012320082
D1287462
D7218101579
D7014750302
D7714330980
D7020410158
D7110920002
D7117830048
D7020526901
D7020180279
D7211730836
D7017470545
D7119561884
D0109850003
D2216667
D1098715
D7112892152
D1985248
D0115630016
D7020691606
D0129590009
D6668214
D7116590379
D7010121065
D1239304
D7016382160
D7126575
D6668397
D0125340094
D7018771071
D1655077
D3511028
D7018890424
D7119832431
D7112420024
D7010000154
D1655146
D7010130015
D7119760217
D7218310616
D1655031
D7214590180
D0114090027
D3462157
D7012701364
D1107231
D0103960016
D6239400
D7010461
D2193482
D7211390148
D7115710010
D1045943
D0129680031
D7015150118
D7139033
D7011500020
D1098467
D1292458
D7114090828
D7212090420
D7211750003
D6923446
D1691939
D7020470060
D1506916
D4788690
D7114520025
D7114890203
D4535632
D7018771138
D7111630010
D1048723
D11180518
D7212090479
D7018771243
D1305927
D7214590327
D1605642
D7212590035
D7119860202
D1043247
D7011460874
D5973187
D0129661063
D7119740031
D6052798
D1556853
D2156457
D7020602982
D7020721466
D7012620018
D7128098
D7010280376
D0105670001
D7013831303
D7020553432
D7012320320
D1655078
D7214590174
D7114830847
D1123570
D7020720973
D1113439
D7015570038
D7215630153
D1071636
D7119812851
D7133036
D7116110114
D7015920277
D7212060121
D0109280046
D7114511756
D7020530016
D7210480063
D7133112
D0115030560
D7114960287
D7010170272
D2125384
D7116972170
D0129530001
D2612057
D7118351677

D7020680857
D7011100070
D7216620005
D1086833
D0125190014
D7117090696
D6239866
D2630843
D7214590498
D0123750924
D3463197
D5110090053
D7114600155
D7216441044
D7013050359
D1642411
D7211300075
D7020555067
D7114980005
D7114490471
D1500469
D7018680454
D7017440253
D1446063
D1345091
D0129660666
D2193439
D7571171
D1200354
D7016233147
D7214140157
D7119760071
D7214910261
D7016270295
D7412610237
D7217300223
D0107620459
D7020600735
D7714330900
D7119930114
D7211720826
D7119891264
D7014750576
D7119832623
D0138070034
D7114090983
D0107930442
D7714330862
D1305646
D6327214
D6053532
D7020618414
D4798661
D7015970017
D3669619
D7119561586
D7018822399
D1001761
D1691687
D6246623
D1043516
D7212530001
D1350371
D7020533075
D7119770965
D7019051205
D7118340509
D1882905
D7020331318
D0109160239
D7119630827
D7119760122
D1446262
D2380265
D11185966
D7013940432
D1002299
D7010080041
D7119450017
D6782717
D7210430586
D7015170109
D7119341367
D7012620513
D7020410200
D11181188
D1446089
D1113939
D1192728
D7015580056
D7015160135
D1111923
D1159784
D7216440004
D0109770765
D1059177
D7020554676
D7214590280
D7013010887
D7216690111
D1182686
D7115710035
D7114830828
D7016390842
D7114490416
D1328918
D7118280144
D7126643
D0125240442
D4794769
D7135174
D1482437
D7010120369
D7117840716
D7020720695
D0112400073
D1089966
D8916328
D7212580028
D0115950174
D1112287
D7013160777
D7211330028
D7116460018
D7018771475
D7013830040
D7218310562
D7020554943
D1056772
D7119692070
D4840209
D0105660528
D1091046
D7011460327
D6246060
D7413120162
D7012620672
D7113232349
D1198887
D7013830647
D7119760148
D7126187
D1097346
D6328859
D7012270730
D7020460336
D0105710031
D7015570012
D4127023
D1282856
D7014740324
D7212970040
D7013050167
D6236084
D7014320407
D6048283
D7020600744
D7010270065
D7018800520
D7114600534
D7015570122
D2318624
D7119220019
D7110940255
D1359333
D7006760
D6244102
D7119880128
D7114490530
D3555953
D0125340085
D7018811914
D4787271
D7020534017
D7119790121
D7214540024

D7020720709
D7020556194
D7119891376
D7018800819
D7113270192
D5482017
D1344799
D7018654879
D6466540
D5040980
D7715560194
D1560248
D0129710031
D7113670343
D1261719
D2630910
D7020691411
D7017680666
D7119480586
D7018990180
D1754010
D7118351769
D1035779
D1191233
D7014320112
D1127911
D7016380482
D7012700917
D1110325
D1197705
D1705372
D0114350084
D7119160017
D1863600
D0125228070
D7119730322
D1095054
D0109420316
D6329869
D7714300112
D7119988627
D1098333
D7020532088
D3462676
D7119641996
D7717710001
D7013860087
D7214190743
D6052891
D7216661079
D7010462
D7020480015
D7020540294
D0107620980
D7211730193
D7020580499
D1772784
D7020556019
D1361083
D7020252451
D7212090560
D7135215
D7020390258
D1897367
D7015450926
D7020580471
D7119930096
D7119812647
D6463872
D1182531
D7012260126
D1790216
D7017440581
D1232677
D7017570264
D7020310173
D7110920985
D7012120011
D7119152646
D1182647
D7119810652
D7012740190
D0128761098
D1484733
D6057898
D1001474
D1106282
D7118062228
D7017520077
D11181163
D7119520553
D7111640141
D7011830084
D6666095
D7015410079
D7010000297
D1034298
D0128680226
D7119812513
D7217970083
D0127442328
D7020260112
D7119730638
D2125368

S5 Reference

1. S. Yun, M. Jeong, R. Kim, J. Kang and H. J. Kim, *Advances in neural information processing systems*, 2019, **32**.
2. A. Vaswani, *Advances in Neural Information Processing Systems*, 2017.
3. L. A. Bratholm, W. Gerrard, B. Anderson, S. Bai, S. Choi, L. Dang, P. Hanchar, A. Howard, S. Kim and Z. Kolter, *Plos one*, 2021, **16**, e0253612.
4. A. Radford, *arXiv preprint arXiv:1511.06434*, 2015.
5. Y. You, J. Li, S. Reddi, J. Hseu, S. Kumar, S. Bhojanapalli, X. Song, J. Demmel, K. Keutzer and C.-J. Hsieh, *arXiv preprint arXiv:1904.00962*, 2019.
6. K. He, X. Zhang, S. Ren and J. Sun, 2016.
7. Gaussian 09, Revision D.01, M. J. Frisch, G. W. Trucks, H. B. Schlegel, G. E. Scuseria, M. A. Robb, J. R. Cheeseman, G. Scalmani, V. Barone, G. A. Petersson, H. Nakatsuji, X. Li, M. Caricato, A. Marenich, J. Bloino, B. G. Janesko, R. Gomperts, B. Mennucci, H. P. Hratchian, J. V. Ortiz, A. F. Izmaylov, J. L. Sonnenberg, D. Williams-Young, F. Ding, F. Lipparini, F. Egidi, J. Goings, B. Peng, A. Petrone, T. Henderson, D. Ranasinghe, V. G. Zakrzewski, J. Gao, N. Rega, G. Zheng, W. Liang, M. Hada, M. Ehara, K. Toyota, R. Fukuda, J. Hasegawa, M. Ishida, T. Nakajima, Y. Honda, O. Kitao, H. Nakai, T. Vreven, K. Throssell, J. A. Montgomery, Jr., J. E. Peralta, F. Ogliaro, M. Bearpark, J. J. Heyd, E. Brothers, K. N. Kudin, V. N. Staroverov, T. Keith, R. Kobayashi, J. Normand, K. Raghavachari, A. Rendell, J. C. Burant, S. S. Iyengar, J. Tomasi, M. Cossi, J. M. Millam, M. Klene, C. Adamo, R. Cammi, J. W. Ochterski, R. L. Martin, K. Morokuma, O. Farkas, J. B. Foresman, and D. J. Fox, Gaussian, Inc., Wallingford CT, 2016.
8. C. Adamo and V. Barone , *J. Chem. Phys.*, 1998, **108** , 664-675
9. A. McLean and G. Chandler , *J. Chem. Phys.*, 1980, **72** , 5639 - 5648
10. R. Krishnan , J. S. Binkley , R. Seeger and J. A. Pople, *J. Chem. Phys.*, 1980, **72**, 650-654
11. J.-D. Chai and M. Head-Gordon , *J. Chem. Phys.*, 2008, **128** , 084106
12. W. Deng , J. R. Cheeseman and M. J. Frisch , *J. Chem. Theory Comput.*, 2006, **2** , 1028-1037.
13. G. Landrum, *Greg Landrum*, 2013, **8**, 5281.
14. T. A. Halgren, *J. Comput. Chem.*, 1996, **17**, 490-519.
15. C. Bannwarth, S. Ehlert and S. Grimme, *Journal of chemical theory and computation*, 2019, **15**, 1652-1671.
16. P. Gao, X. Wang and H. Yu, *Advanced Theory and Simulations*, 2019, **2**, 1800148.
17. P. Gao, J. Zhang and H. Chen, *International journal of quantum chemistry*, 2021, **121**, e26482.
18. W. Gerrard, L. A. Bratholm, M. J. Packer, A. J. Mulholland, D. R. Glowacki and C. P. Butts, *Chemical science*, 2020, **11**, 508-515.
19. Y. Guan, S. S. Sowndarya, L. C. Gallegos, P. C. S. John and R. S. Paton, *Chemical Science*, 2021, **12**, 12012-12026.
20. C.Dickson, PhD Thesis, University of Bristol, 2018 https://hdl.handle.net/1983/7f93852e-3a5e-45b2-b8b3-23dc0694b601
